# Supplementary material for: SIRT5-mediated desuccinylation of PPA2 enhances HIF-1alpha-dependent adaptation to hypoxic stress and colorectal cancer metastasis
Source: EMBO J. 2025 Mar 31;44(9):2514–40. doi: 10.1038/s44318-025-00416-1 (PMC12048626; doi:10.1038/s44318-025-00416-1)
Supplement: Supplementary file 1 — Table EV1 [file 44318_2025_416_MOESM1_ESM.docx]

**Table EV1. STR profiles of cell lines**

|  | **DLD1** | **SW1116** | **HEK293T** |
| --- | --- | --- | --- |
| **STR loci** |  |  |  |
| Amelogenin | X, Y | X, Y | X, X |
| TH01 | 7, 9.3 | 6, 6 | 7, 9.3 |
| D5S818 | 13, 13 | 11, 12 | 8, 9 |
| D13S317 | 8, 11 | 11, 14 | 12, 14 |
| D7S820 | 10, 12 | 12, 12 | 11, 11 |
| D16S539 | 12, 13 | 9, 12 | 9, 13 |
| CSFIPO | 11, 12 | 10, 11 | 11, 12 |
| vWA | 18, 19 | 14, 19 | 16, 19 |
| TPOX | 8, 11 | 8, 11 | 11, 11 |
