# Supplementary material for: SIRT5-mediated desuccinylation of PPA2 enhances HIF-1alpha-dependent adaptation to hypoxic stress and colorectal cancer metastasis
Source: EMBO J. 2025 Mar 31;44(9):2514–40. doi: 10.1038/s44318-025-00416-1 (PMC12048626; doi:10.1038/s44318-025-00416-1)
Supplement: Supplementary file 2 — Table EV2 [file 44318_2025_416_MOESM2_ESM.docx]

**Table EV2. Oligonucleotide sequences**

| **Name** | **Sequences (5'-3')** | |
| --- | --- | --- |
| **qPCR primers for human** | **Forward (5'-3')** | **Reverse (5'-3')** |
| 18S | GTAACCCGTTGAACCCCATT | CCATCCAATCGGTAGTAGCG |
| β-actin | CATGTACGTTGCTATCCAGGC | CTCCTTAATGTCACGCACGAT |
| PPA2 | AAGAAGTTCAAACCGGGTTACC | GCAACGGAAAGGGCTATCAGA |
| LOX | CGGCGGAGGAAAACTGTCT | TCGGCTGGGTAAGAAATCTGA |
| LOXL1 | GGCTGCTATGACACCTACAATG | GTAGTGAATGTTGCATCTCACCA |
| SNAI1 | TCGGAAGCCTAACTACAGCGA | AGATGAGCATTGGCAGCGAG |
| ALDOA | ATGCCCTACCAATATCCAGCA | GCTCCCAGTGGACTCATCTG |
| ENO1 | AAAGCTGGTGCCGTTGAGAA | GGTTGTGGTAAACCTCTGCTC |
| LDHA | ATGGCAACTCTAAAGGATCAGC | CCAACCCCAACAACTGTAATCT |
| PFKP | CGCCTACCTCAACGTGGTG | ACCTCCAGAACGAAGGTCCTC |
| PKM | ATGTCGAAGCCCCATAGTGAA | TGGGTGGTGAATCAATGTCCA |
| HIF-1α | GAACGTCGAAAAGAAAAGTCTCG | CCTTATCAAGATGCGAACTCACA |
| SIRT5 | GCCATAGCCGAGTGTGAGAC | CAACTCCACAAGAGGTACATCG |
| SIRT7 | GACCTGGTAACGGAGCTGC | CGACCAAGTATTTGGCGTTCC |
| ELOVL6 | GCACCCGAACTAGGAGATACA | CCCCGGCAACCATGTCTTT |
| DLAT | CGGAACTCCACGAGTGACC | CCCCGCCATACCCTGTAGT |
| CRYAB | CCTGAGTCCCTTCTACCTTCG | CACATCTCCCAACACCTTAACTT |
| LITAF | ATGTCGGTTCCAGGACCTTAC | TACGAAGGAGGATTCATGCCC |
| NEDD4 | CAGGCCCTCAATCACAAGC | AGGCCCTAGATCATTGGAAGT |
| NEDD4L | GACATGGAGCATGGATGGGAA | GTTCGGCCTAAATTGTCCACT |
| PPIL2 | GCCAAACAGGACCCGTCTTAT | AGCTGCTTCATGTGTGGTCTC |
| SYVN1 | AACCCCTGGGACAACAAGG | GCGAGACATGATGGCATCTG |
| SNAI1 (promoter) | GTGCTCTTGGCTAGCTG | GAGAGCGTGGCATTGAC |
| LOX (promoter) | GGGCTGGTGACCTAATAGC | GACACACTGGCTTAATCTGG |
| LDHA (promoter) | GACTCGAGATGAGATGCC | CTCTGCTGCTAAGCCTAC |
| ENO1 (promoter) | GCTCACCGGTCCTATCTG | CATTCGTCGGGAGAGC |
| **qPCR primers for mouse** | **Forward (5'-3')** | **Reverse (5'-3')** |
| LOX | CAGCCACATAGATCGCATGGT | GCCGTATCCAGGTCGGTTC |
| LOXL1 | CGCCCTTCGTAAACCAGTATG | CACCACGGTAGTACACGTAGC |
| SNAI1 | CACACGCTGCCTTGTGTCT | GGTCAGCAAAAGCACGGTT |
| ALDOA | CGTGTGAATCCCTGCATTGG | CAGCCCCTGGGTAGTTGTC |
| ENO1 | TGCGTCCACTGGCATCTAC | CAGAGCAGGCGCAATAGTTTTA |
| LDHA | CAAAGACTACTGTGTAACTGCGA | TGGACTGTACTTGACAATGTTGG |
| PFKP | CGCCTATCCGAAGTACCTGGA | CCCCGTGTAGATTCCCATGC |
| PKM | CGCCTGGACATTGACTCTG | GAAATTCAGCCGAGCCACATT |
| β-actin | GGCTGTATTCCCCTCCATCG | CCAGTTGGTAACAATGCCATGT |
| **siRNA, shRNA, sgRNA** |  | |
| siPPA2#1 | GCCUCUUCUUUAAGAAUGUAA | |
| siPPA2#2 | CCUAUGAAGAAAGCACGAAAU | |
| siELOVL6#1 | CCAGUAAGUUUAUGAUCCUUU | |
| siELOVL6#2 | CCUAAUGAAUAAACGAGCAAA | |
| siDLAT#1 | CCAUACCUCAUUAUUACCUUU | |
| siDLAT#2 | GCAGAGGUUGAAACUGAUAAA | |
| siCRYAB | CCAUUACUUCAUCCCUGUCAU | |
| siLITAF | GCACACAGAUUCACUUUAAUU | |
| siPPIL2 | UCUCAAUUCUUCAUCACGUUU | |
| siSYVN1 | UGAAUGCUUAAUCCCGGGAAA | |
| siNEDD4 | CGCCUUGACUUACCUCCAUAU | |
| siWWP2 | CCUCACCUACUUUCGCUUUAU | |
| siNEDD4L | CGCCUUGACUUACCUCCAUAU | |
| siSIRT5 | CGUCCACACGAAACCAGAUUU | |
| siSIRT7 | GUCCAGCCUGAAGGUUCUAAA | |
| shPPA2#1 | GCCTCTTCTTTAAGAATGTAA | |
| shPPA2#2 | CCTATGAAGAAAGCACGAAAT | |
| shNEDD4 | CGCCTTGACTTACCTCCATAT | |
| shSIRT5 | CGTCCACACGAAACCAGATTT | |
| shVHL | TATCACACTGCCAGTGTATAC | |
| sgHIF-1α | AACCATAACAAAACCATCCA | |
