# Supplementary material for: SIRT5-mediated desuccinylation of PPA2 enhances HIF-1alpha-dependent adaptation to hypoxic stress and colorectal cancer metastasis
Source: EMBO J. 2025 Mar 31;44(9):2514–40. doi: 10.1038/s44318-025-00416-1 (PMC12048626; doi:10.1038/s44318-025-00416-1)
Supplement: Supplementary file 3 — Table EV3 [file 44318_2025_416_MOESM3_ESM.docx]

**Table EV3. CRLM burden score**

|  |  | score |
| --- | --- | --- |
| The number of liver metastasis | 1-3 | 1 |
|  | 4-6 | 2 |
|  | 7-9 | 3 |
|  | 10-12 | 5 |
|  | 13-15 | 7 |
|  | 16-18 | 9 |
|  | 19-21 | 11 |
|  | >21 | 14 |
| Maximum tumor diameter | >2.5mm | 3 |
|  | >5mm | 6 |
|  | >10mm | 11 |

Note. CRLM burden score = score (the number of liver metastasis) + score (maximum tumor diameter)
