# Supplementary material for: SIRT5-mediated desuccinylation of PPA2 enhances HIF-1alpha-dependent adaptation to hypoxic stress and colorectal cancer metastasis
Source: EMBO J. 2025 Mar 31;44(9):2514–40. doi: 10.1038/s44318-025-00416-1 (PMC12048626; doi:10.1038/s44318-025-00416-1)
Supplement: Supplementary file 4 — Appendix [file 44318_2025_416_MOESM4_ESM.pdf]

Appendix

SIRT5-mediated desuccinylation of PPA2 enhances HIF-1alpha-dependent adaptation to hypoxic stress and colorectal cancer metastasis

Xiang Zhang, Yuqin Di, Youpeng Wang, Jiale Qin, Lvlan Ye, Xiangqiong Wen, Zunfu Ke, Ziyang Wang, Weiling He

The file includes:

Appendix Table S1..2

Appendix Table S1. Product information and CAS number of metabolite standards

Compounds	Product information	CAS
D-Glucose-6-phosphate (G6P)	#ZTR-G595338, TRC	56-73-5
D-Fructose-6-phosphate (F6P)	#ZG-23130, Glycosci	26177-86-9
Dihydroxyacetone-phosphate (DHAP)	#51269, Sigma	20915
Glyceraldehyde-3-phosphate (G3P)	#39705, Sigma	591-57-1
3-phosphoglycerate (3-PGA)	#P8877, Sigma	820-11-1
2-Phospho-D-glyceric acid (2-PGA)	#ZTR-P358000, TRC	3443-57-0
Phosphoenolpyruvic-acid (PEP)	#ZTR-P360870, Sigma	138-08-9
Pyruvic-acid (PA)	#RO10854, Sigma	138-08-9
Lactate	#L1750, Sigma	50-21-5
Cis-Aconitic-acid (CAA)	#ZC-66443, Zzstandard	585-84-2
Succinic-Acid (SA)	#ZL-158003, Zzstandard	2387-71-5
Fumaric-acid (FA)	#F110741, Aladdin	110-17-8
L-Glutamic-acid (Glu)	#ZC-45481, Zzstandard	56-86-0
Glutamine (Gln)	#CDAA-281494, ANPEL	56-85-9
