# Supplementary figures and images for: SIRT5-mediated desuccinylation of PPA2 enhances HIF-1alpha-dependent adaptation to hypoxic stress and colorectal cancer metastasis (part 1 of 5)
Source: EMBO J. 2025 Mar 31;44(9):2514–40. doi: 10.1038/s44318-025-00416-1 (PMC12048626; doi:10.1038/s44318-025-00416-1)

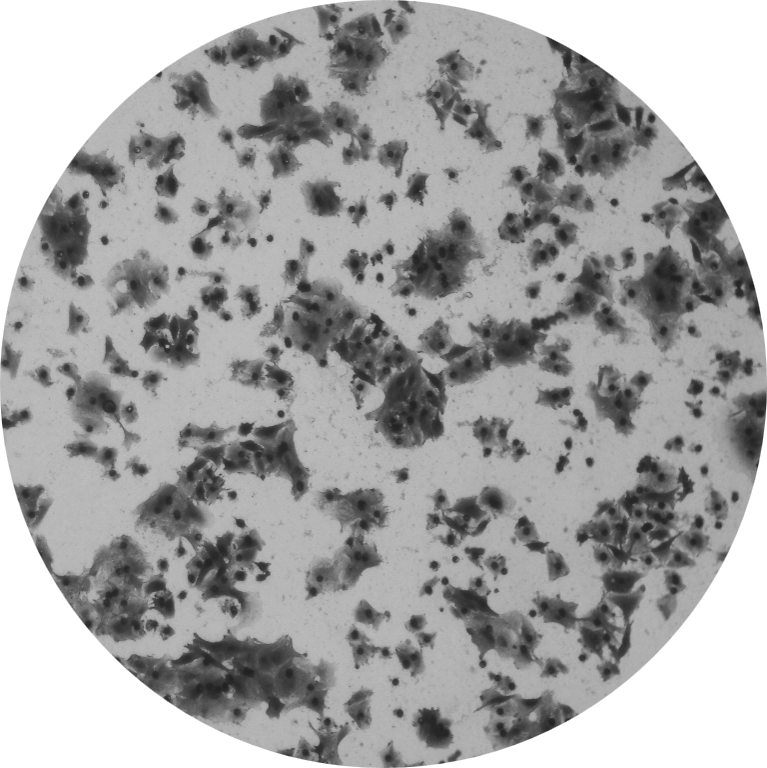

Supplement: Supplementary file 9 — Source data Fig. 1 [file 44318_2025_416_MOESM9_ESM.zip › EMBOJ-2024-119243R_SourceDataForFigure 1/1B/siDLAT.tif]

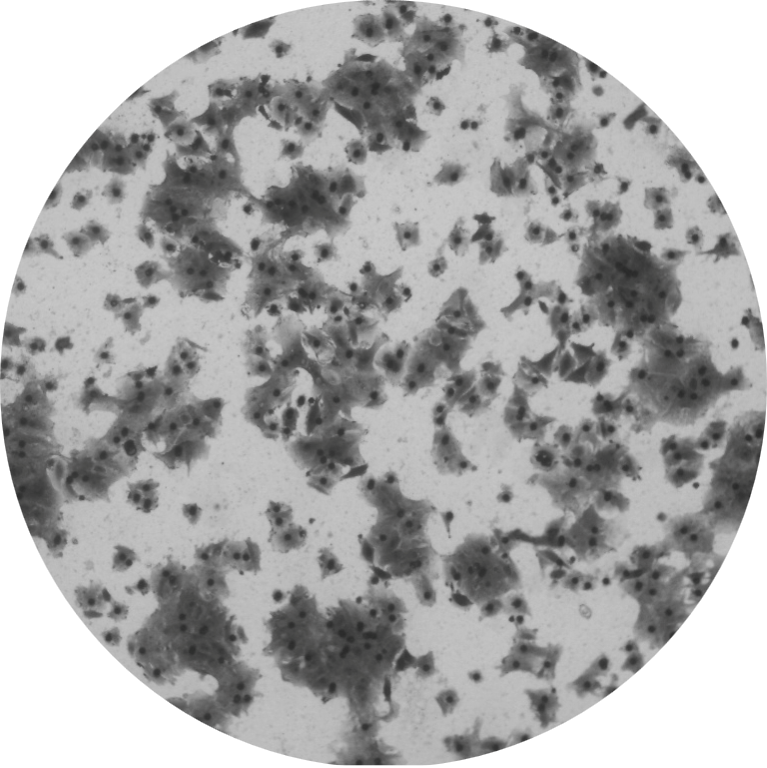

Supplement: Supplementary file 9 — Source data Fig. 1 [file 44318_2025_416_MOESM9_ESM.zip › EMBOJ-2024-119243R_SourceDataForFigure 1/1B/siELOVL6.tif]

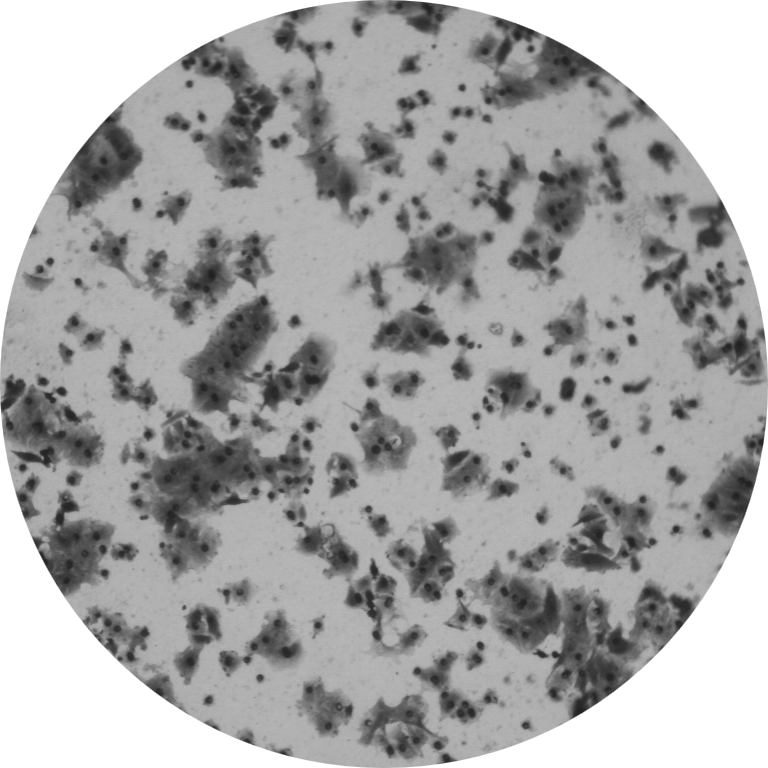

Supplement: Supplementary file 9 — Source data Fig. 1 [file 44318_2025_416_MOESM9_ESM.zip › EMBOJ-2024-119243R_SourceDataForFigure 1/1B/siNC.tif]

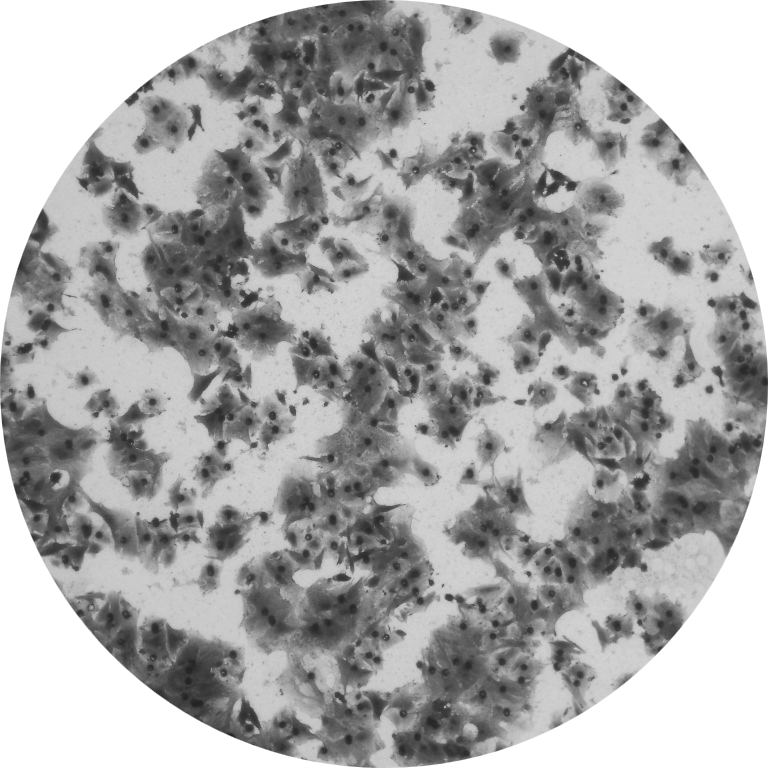

Supplement: Supplementary file 9 — Source data Fig. 1 [file 44318_2025_416_MOESM9_ESM.zip › EMBOJ-2024-119243R_SourceDataForFigure 1/1B/siPPA2.tif]

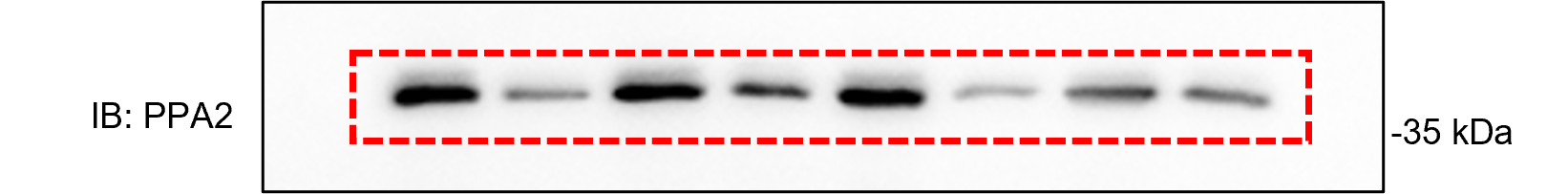

Supplement: Supplementary file 9 — Source data Fig. 1 [file 44318_2025_416_MOESM9_ESM.zip › EMBOJ-2024-119243R_SourceDataForFigure 1/1F/PPA2-1.tif]

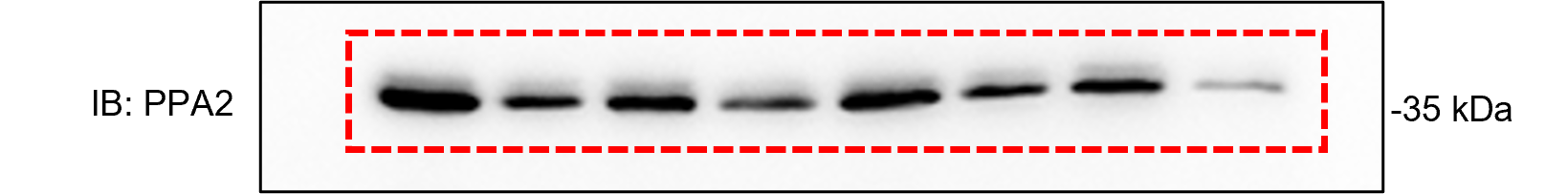

Supplement: Supplementary file 9 — Source data Fig. 1 [file 44318_2025_416_MOESM9_ESM.zip › EMBOJ-2024-119243R_SourceDataForFigure 1/1F/PPA2-2.tif]

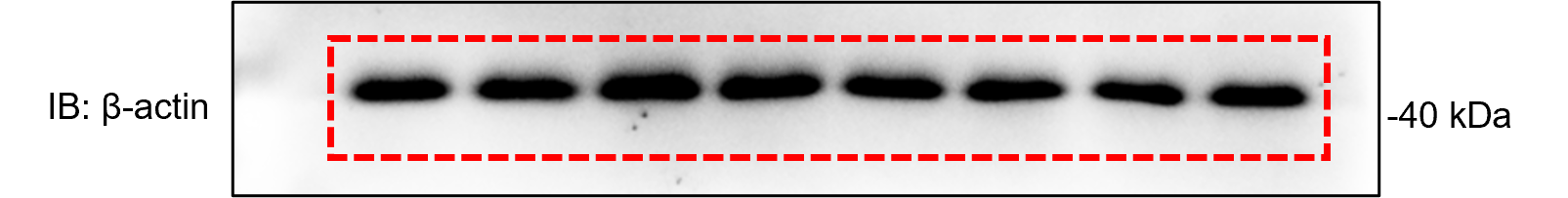

Supplement: Supplementary file 9 — Source data Fig. 1 [file 44318_2025_416_MOESM9_ESM.zip › EMBOJ-2024-119243R_SourceDataForFigure 1/1F/β-actin-1.tif]

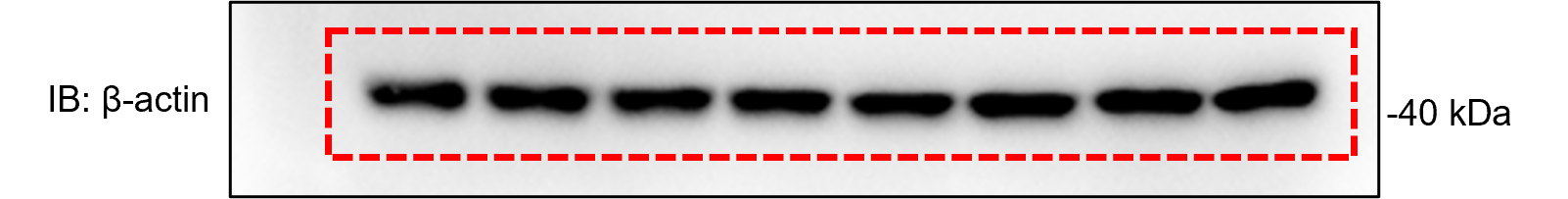

Supplement: Supplementary file 9 — Source data Fig. 1 [file 44318_2025_416_MOESM9_ESM.zip › EMBOJ-2024-119243R_SourceDataForFigure 1/1F/β-actin-2.tif]

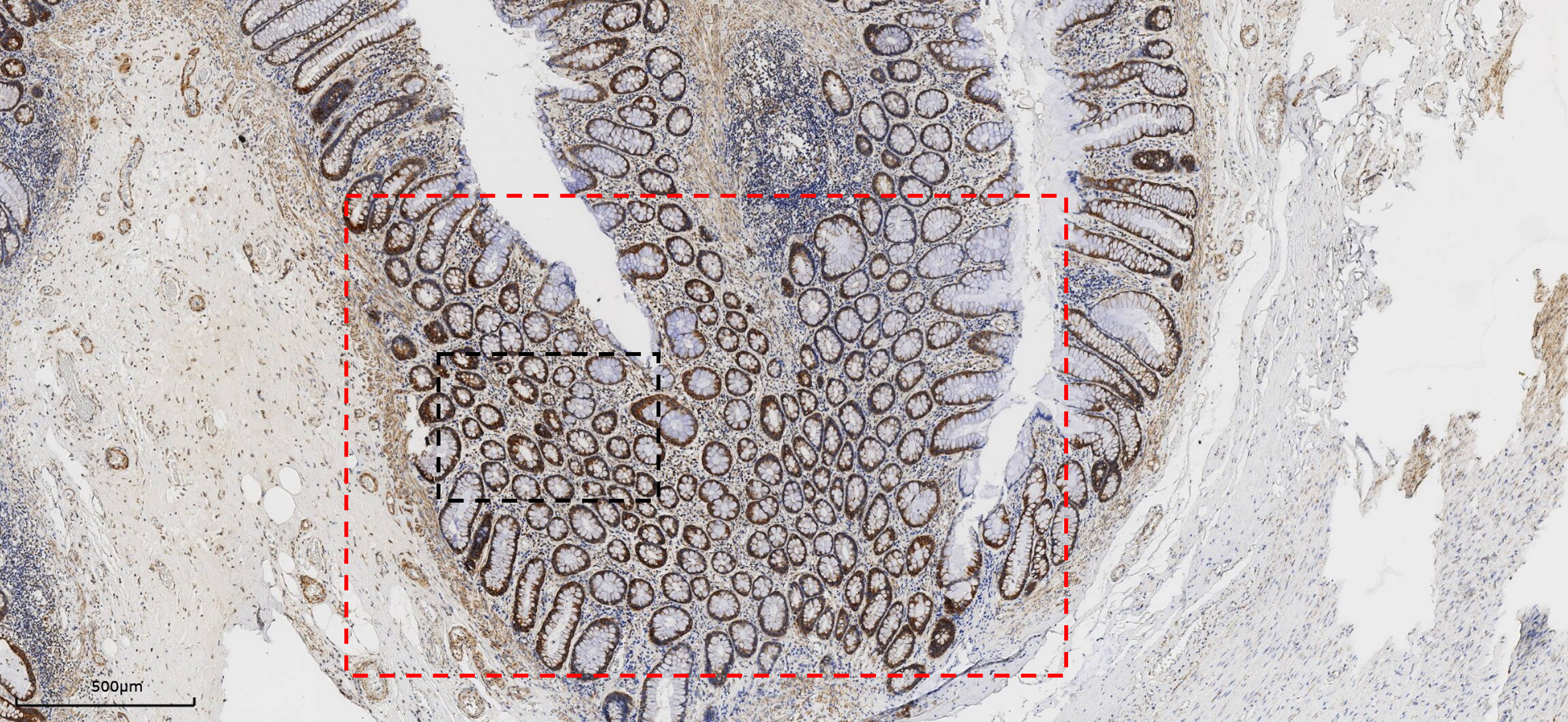

Supplement: Supplementary file 9 — Source data Fig. 1 [file 44318_2025_416_MOESM9_ESM.zip › EMBOJ-2024-119243R_SourceDataForFigure 1/1G/Peritumoral.tif]

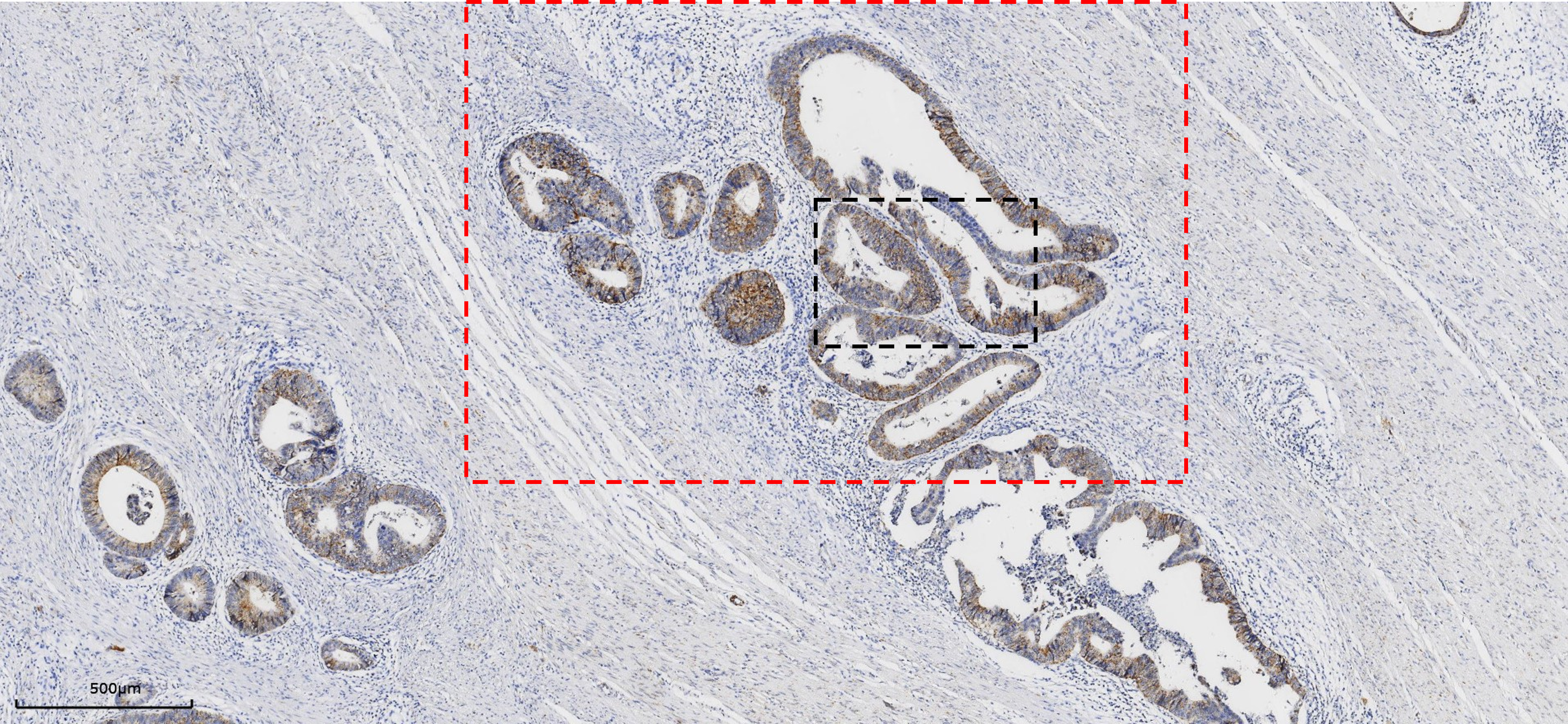

Supplement: Supplementary file 9 — Source data Fig. 1 [file 44318_2025_416_MOESM9_ESM.zip › EMBOJ-2024-119243R_SourceDataForFigure 1/1G/Tumor.tif]

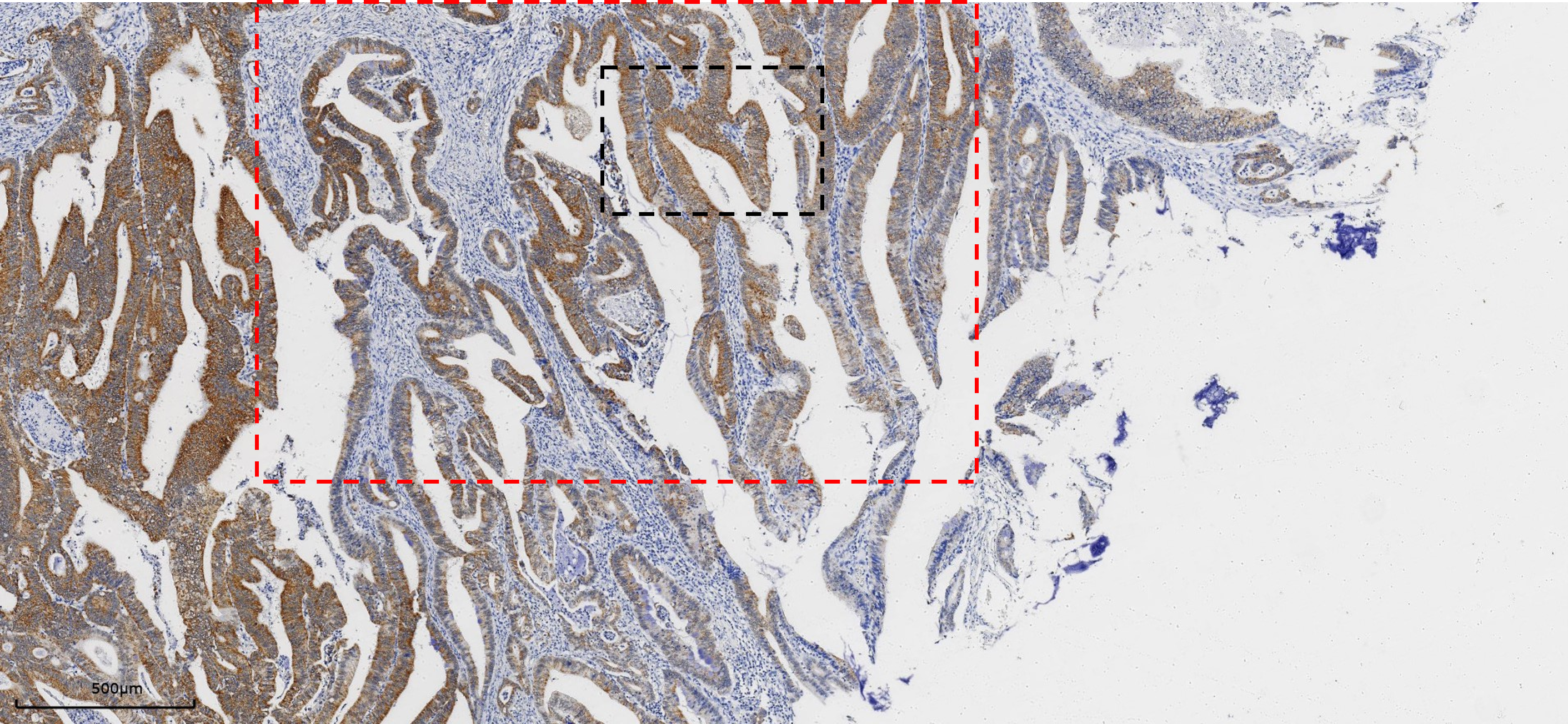

Supplement: Supplementary file 9 — Source data Fig. 1 [file 44318_2025_416_MOESM9_ESM.zip › EMBOJ-2024-119243R_SourceDataForFigure 1/1H/stage I.tif]

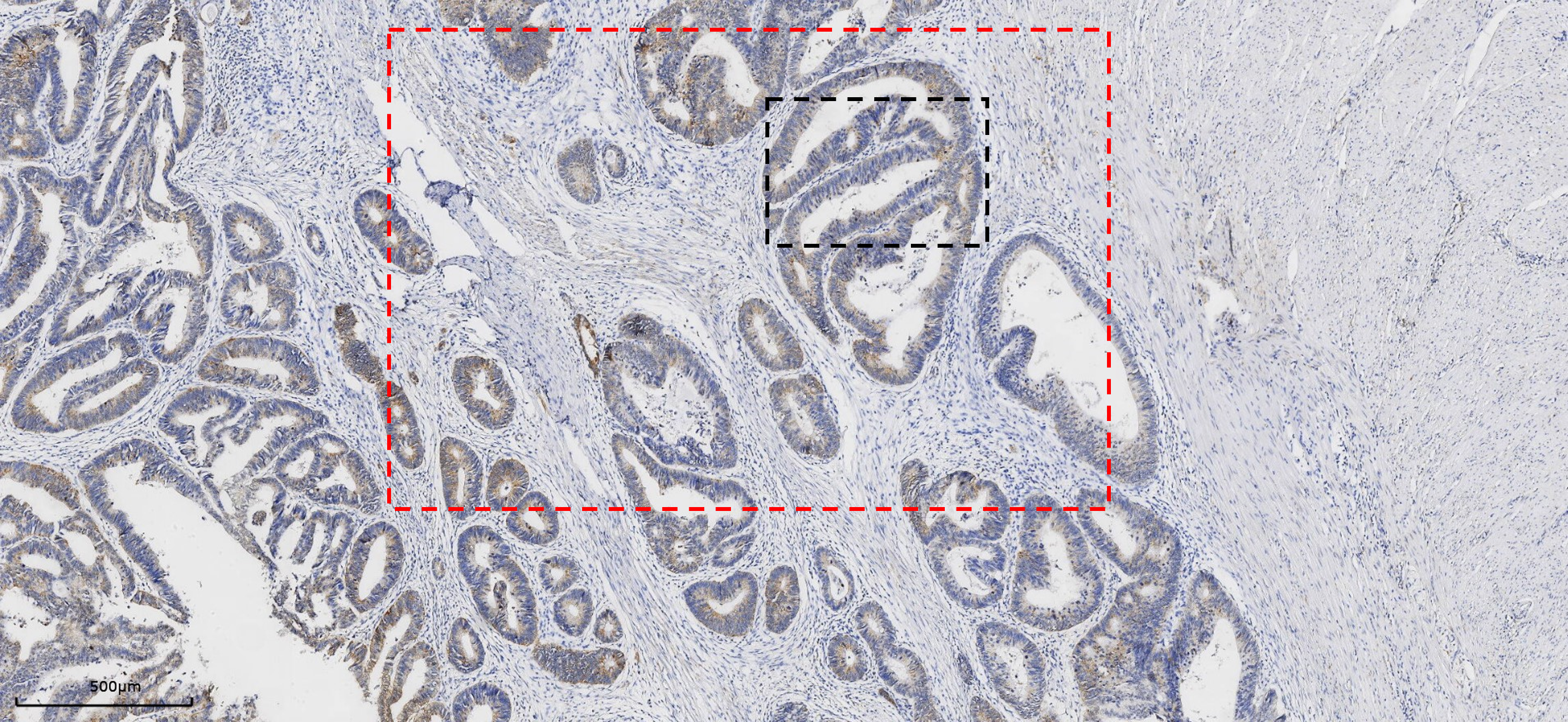

Supplement: Supplementary file 9 — Source data Fig. 1 [file 44318_2025_416_MOESM9_ESM.zip › EMBOJ-2024-119243R_SourceDataForFigure 1/1H/stage IV.tif]

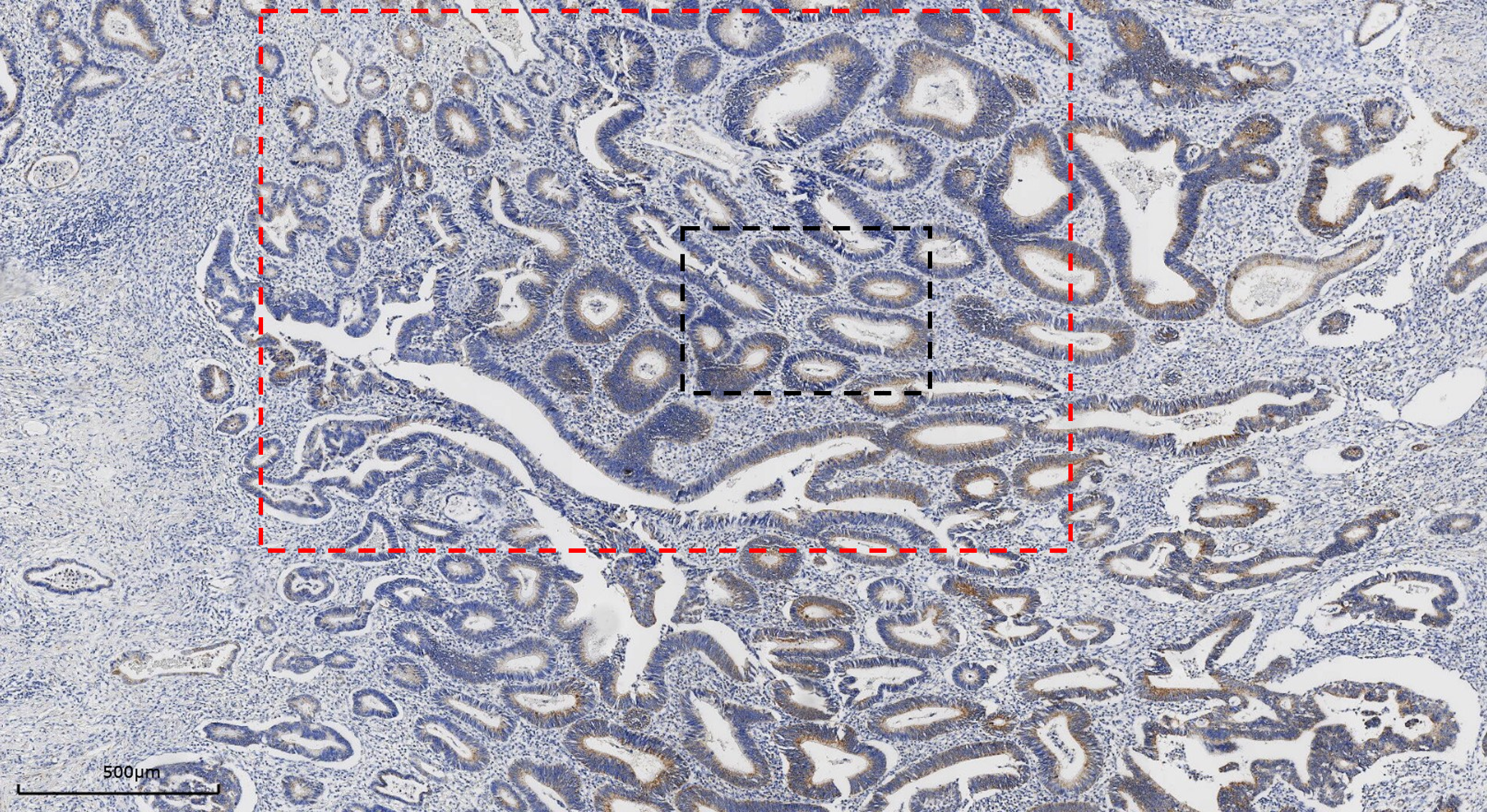

Supplement: Supplementary file 9 — Source data Fig. 1 [file 44318_2025_416_MOESM9_ESM.zip › EMBOJ-2024-119243R_SourceDataForFigure 1/1I/Metastatic.tif]

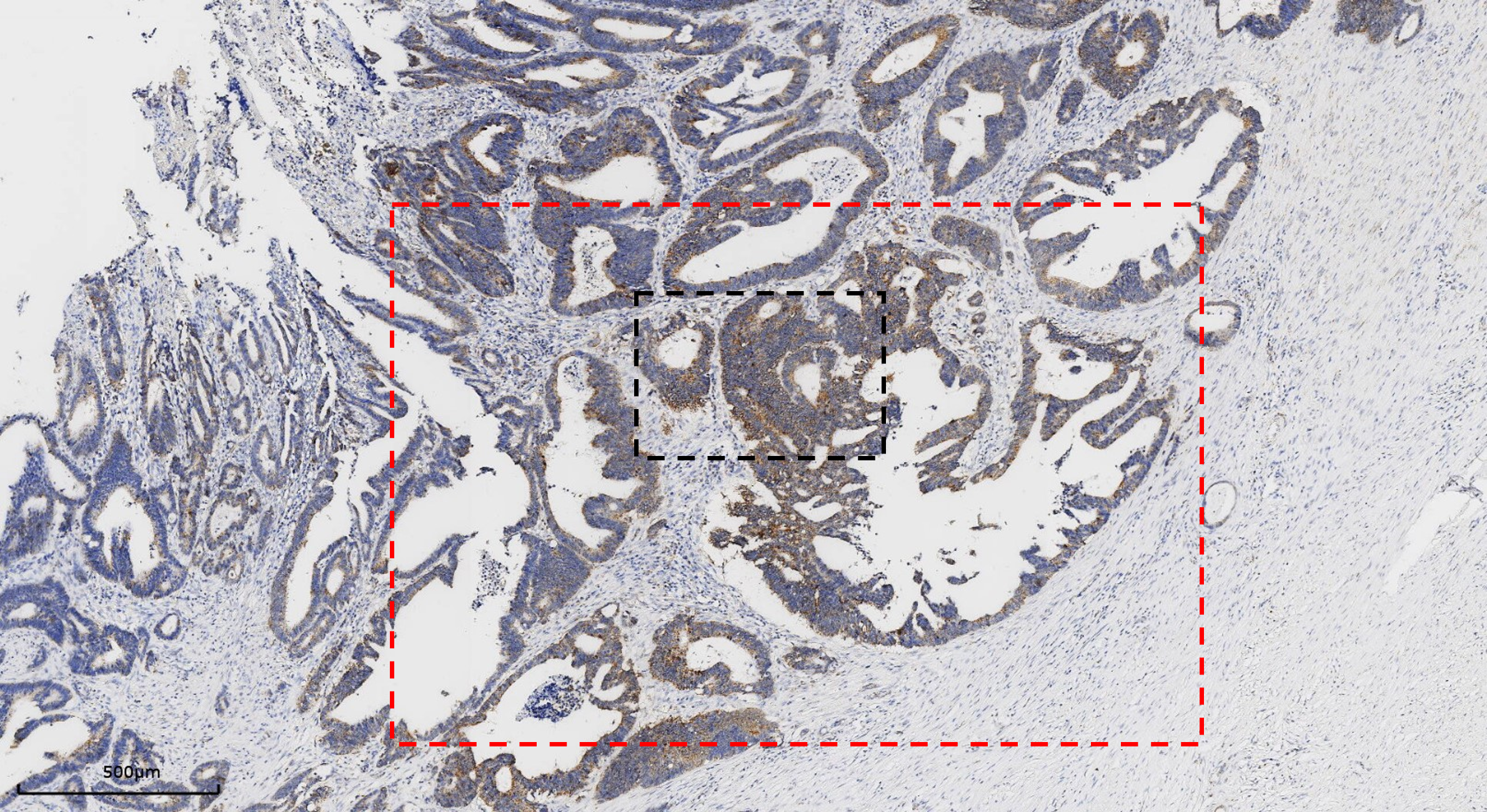

Supplement: Supplementary file 9 — Source data Fig. 1 [file 44318_2025_416_MOESM9_ESM.zip › EMBOJ-2024-119243R_SourceDataForFigure 1/1I/No metastatic.tif]

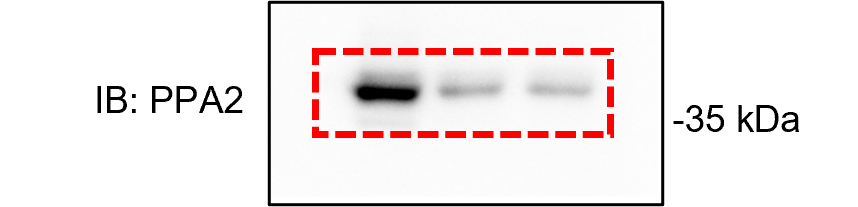

Supplement: Supplementary file 10 — Source data Fig. 2 [file 44318_2025_416_MOESM10_ESM.zip › EMBOJ-2024-119243R_SourceDataForFigure 2/2A/DLD1-PPA2.tif]

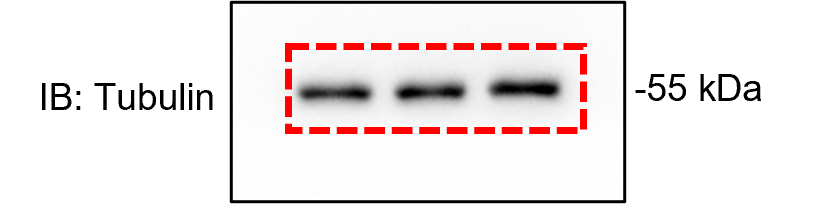

Supplement: Supplementary file 10 — Source data Fig. 2 [file 44318_2025_416_MOESM10_ESM.zip › EMBOJ-2024-119243R_SourceDataForFigure 2/2A/DLD1-Tubulin.tif]

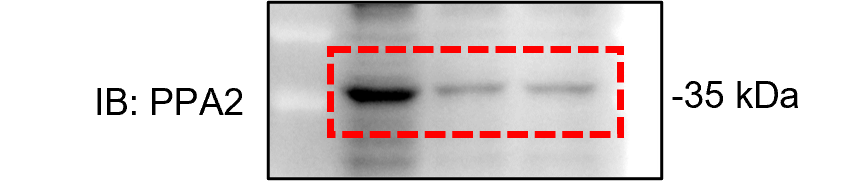

Supplement: Supplementary file 10 — Source data Fig. 2 [file 44318_2025_416_MOESM10_ESM.zip › EMBOJ-2024-119243R_SourceDataForFigure 2/2A/SW1116-PPA2.tif]

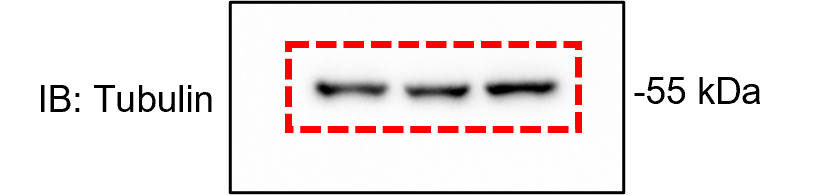

Supplement: Supplementary file 10 — Source data Fig. 2 [file 44318_2025_416_MOESM10_ESM.zip › EMBOJ-2024-119243R_SourceDataForFigure 2/2A/SW1116-Tubulin.tif]

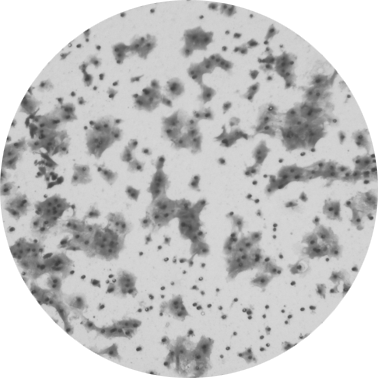

Supplement: Supplementary file 10 — Source data Fig. 2 [file 44318_2025_416_MOESM10_ESM.zip › EMBOJ-2024-119243R_SourceDataForFigure 2/2B/DLD1-HO-shNT-migration.tif]

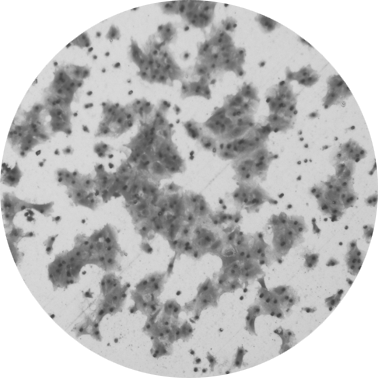

Supplement: Supplementary file 10 — Source data Fig. 2 [file 44318_2025_416_MOESM10_ESM.zip › EMBOJ-2024-119243R_SourceDataForFigure 2/2B/DLD1-HO-shPPA2#1-migration.tif]

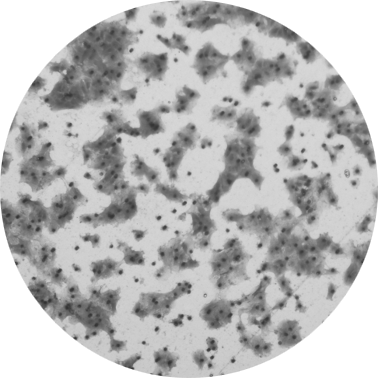

Supplement: Supplementary file 10 — Source data Fig. 2 [file 44318_2025_416_MOESM10_ESM.zip › EMBOJ-2024-119243R_SourceDataForFigure 2/2B/DLD1-HO-shPPA2#2-migration.tif]

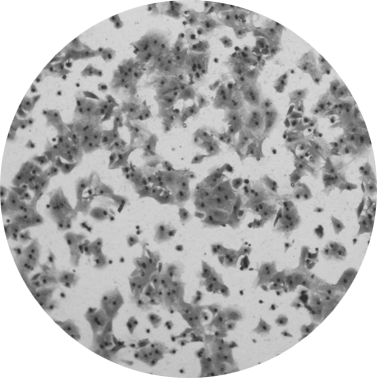

Supplement: Supplementary file 10 — Source data Fig. 2 [file 44318_2025_416_MOESM10_ESM.zip › EMBOJ-2024-119243R_SourceDataForFigure 2/2B/DLD1-LO-shNT-migration.tif]

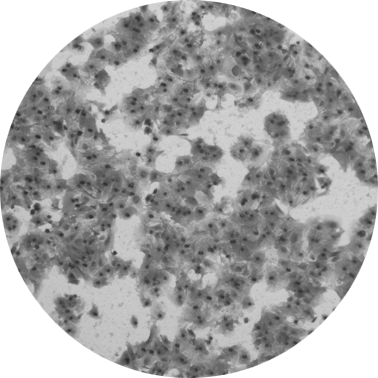

Supplement: Supplementary file 10 — Source data Fig. 2 [file 44318_2025_416_MOESM10_ESM.zip › EMBOJ-2024-119243R_SourceDataForFigure 2/2B/DLD1-LO-shPPA2#1-migration.tif]

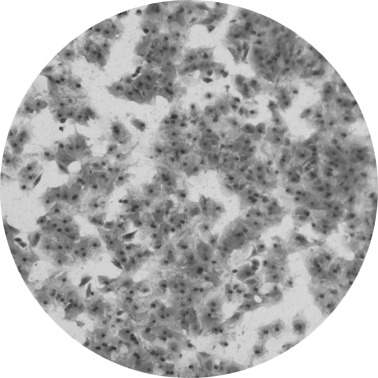

Supplement: Supplementary file 10 — Source data Fig. 2 [file 44318_2025_416_MOESM10_ESM.zip › EMBOJ-2024-119243R_SourceDataForFigure 2/2B/DLD1-LO-shPPA2#2-migration.tif]

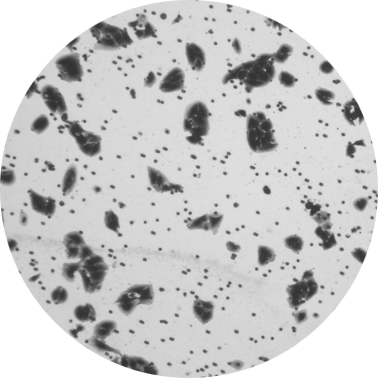

Supplement: Supplementary file 10 — Source data Fig. 2 [file 44318_2025_416_MOESM10_ESM.zip › EMBOJ-2024-119243R_SourceDataForFigure 2/2B/SW1116-HO-shNT-migration.tif]

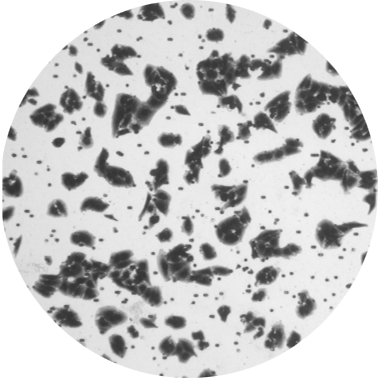

Supplement: Supplementary file 10 — Source data Fig. 2 [file 44318_2025_416_MOESM10_ESM.zip › EMBOJ-2024-119243R_SourceDataForFigure 2/2B/SW1116-HO-shPPA2#1-migration.tif]

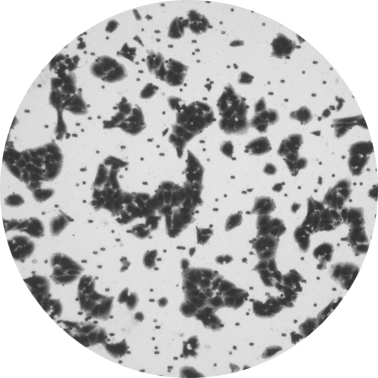

Supplement: Supplementary file 10 — Source data Fig. 2 [file 44318_2025_416_MOESM10_ESM.zip › EMBOJ-2024-119243R_SourceDataForFigure 2/2B/SW1116-HO-shPPA2#2-migration.tif]

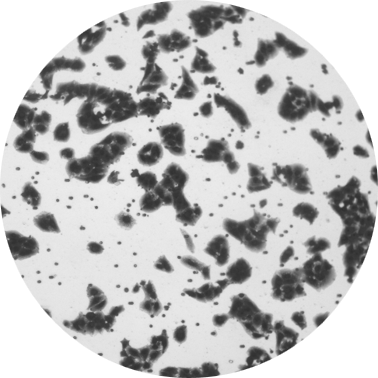

Supplement: Supplementary file 10 — Source data Fig. 2 [file 44318_2025_416_MOESM10_ESM.zip › EMBOJ-2024-119243R_SourceDataForFigure 2/2B/SW1116-LO-shNT-migration.tif]

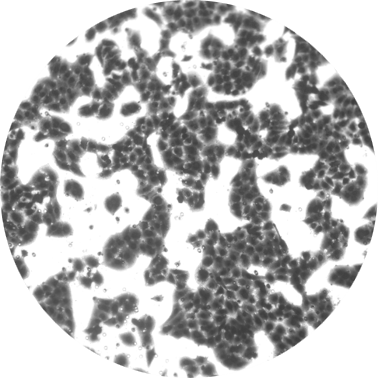

Supplement: Supplementary file 10 — Source data Fig. 2 [file 44318_2025_416_MOESM10_ESM.zip › EMBOJ-2024-119243R_SourceDataForFigure 2/2B/SW1116-LO-shPPA2#1-migration.tif]

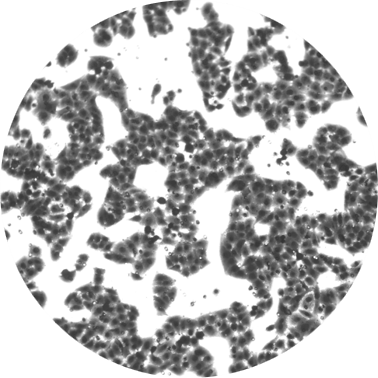

Supplement: Supplementary file 10 — Source data Fig. 2 [file 44318_2025_416_MOESM10_ESM.zip › EMBOJ-2024-119243R_SourceDataForFigure 2/2B/SW1116-LO-shPPA2#2-migration.tif]

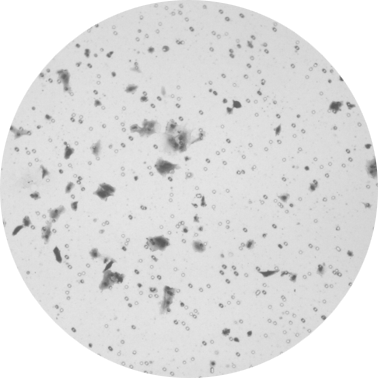

Supplement: Supplementary file 10 — Source data Fig. 2 [file 44318_2025_416_MOESM10_ESM.zip › EMBOJ-2024-119243R_SourceDataForFigure 2/2C/DLD1-HO-shNT-INVASION.tif]

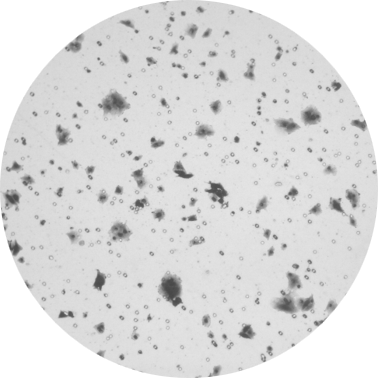

Supplement: Supplementary file 10 — Source data Fig. 2 [file 44318_2025_416_MOESM10_ESM.zip › EMBOJ-2024-119243R_SourceDataForFigure 2/2C/DLD1-HO-shPPA2#1-INVASION.tif]

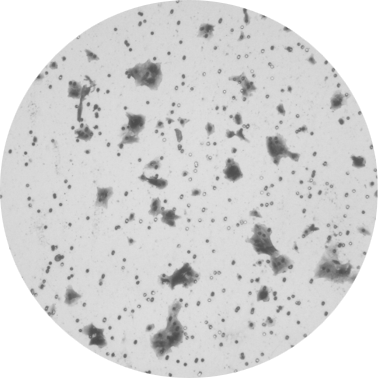

Supplement: Supplementary file 10 — Source data Fig. 2 [file 44318_2025_416_MOESM10_ESM.zip › EMBOJ-2024-119243R_SourceDataForFigure 2/2C/DLD1-HO-shPPA2#2-INVASION.tif]

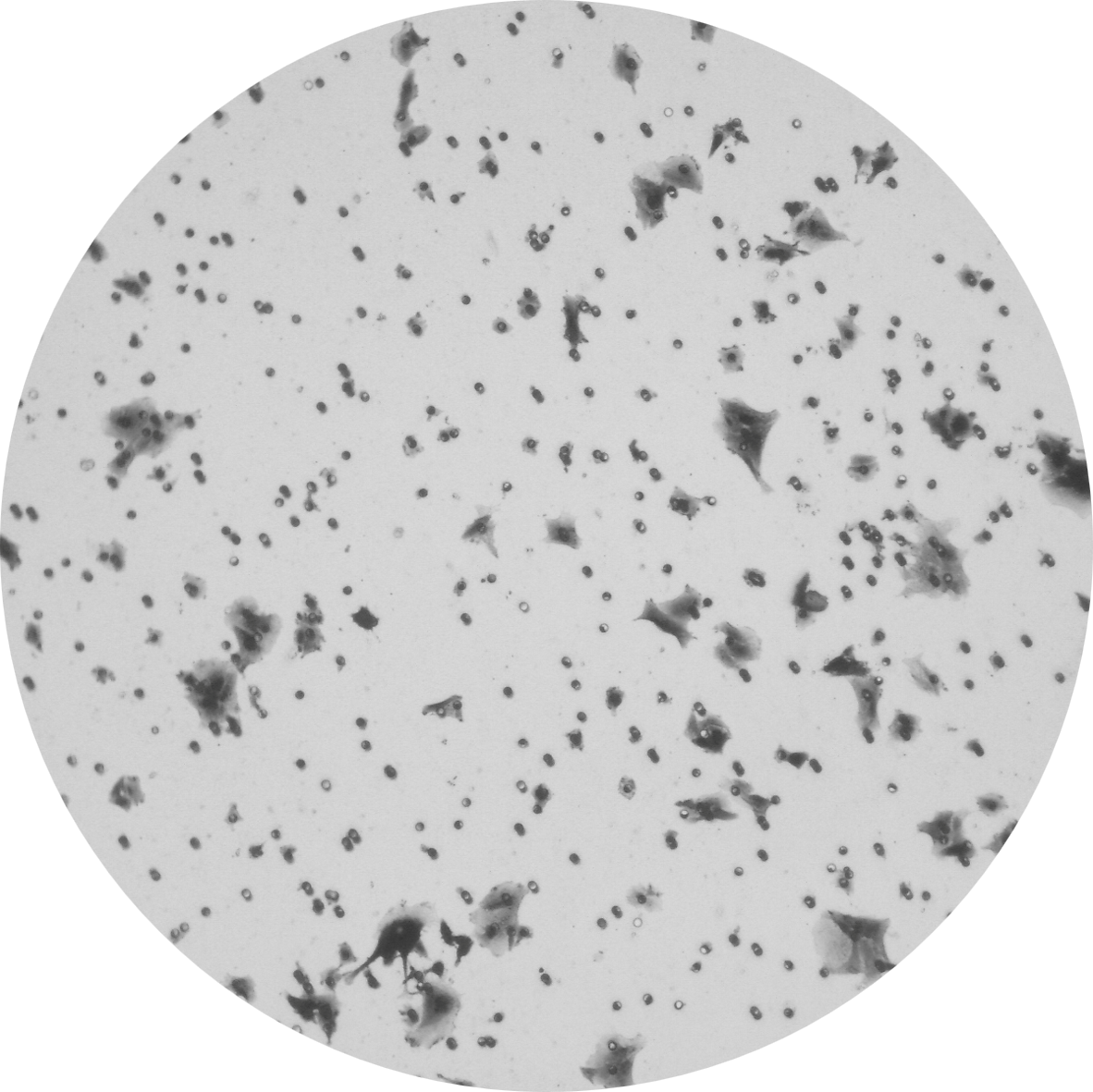

Supplement: Supplementary file 10 — Source data Fig. 2 [file 44318_2025_416_MOESM10_ESM.zip › EMBOJ-2024-119243R_SourceDataForFigure 2/2C/DLD1-LO-shNT-INVASION.tif]

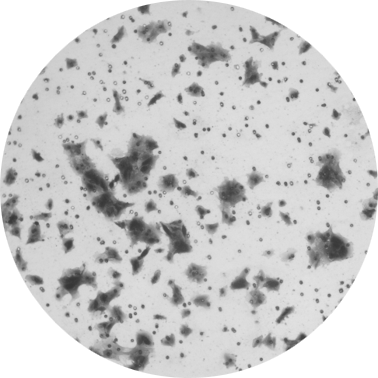

Supplement: Supplementary file 10 — Source data Fig. 2 [file 44318_2025_416_MOESM10_ESM.zip › EMBOJ-2024-119243R_SourceDataForFigure 2/2C/DLD1-LO-shPPA2#1-INVASION.tif]

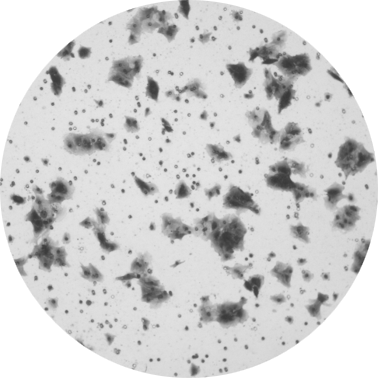

Supplement: Supplementary file 10 — Source data Fig. 2 [file 44318_2025_416_MOESM10_ESM.zip › EMBOJ-2024-119243R_SourceDataForFigure 2/2C/DLD1-LO-shPPA2#2-INVASION.tif]

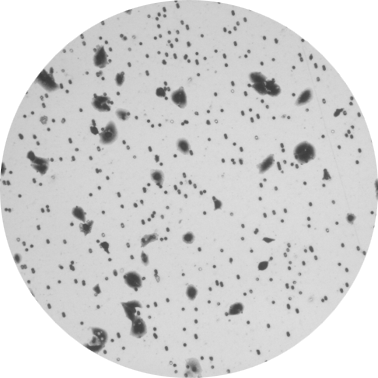

Supplement: Supplementary file 10 — Source data Fig. 2 [file 44318_2025_416_MOESM10_ESM.zip › EMBOJ-2024-119243R_SourceDataForFigure 2/2C/SW1116-HO-shNT-INVASION.tif]

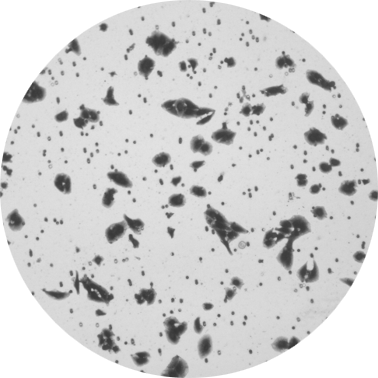

Supplement: Supplementary file 10 — Source data Fig. 2 [file 44318_2025_416_MOESM10_ESM.zip › EMBOJ-2024-119243R_SourceDataForFigure 2/2C/SW1116-HO-shPPA2#1-INVASION.tif]

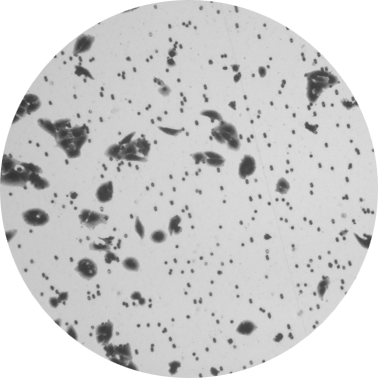

Supplement: Supplementary file 10 — Source data Fig. 2 [file 44318_2025_416_MOESM10_ESM.zip › EMBOJ-2024-119243R_SourceDataForFigure 2/2C/SW1116-HO-shPPA2#2-INVASION.tif]

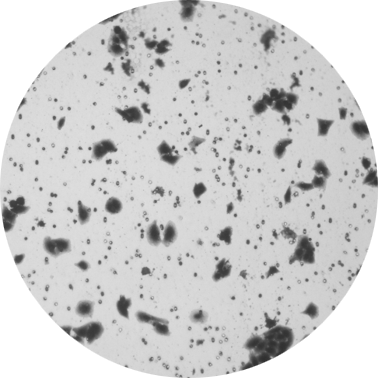

Supplement: Supplementary file 10 — Source data Fig. 2 [file 44318_2025_416_MOESM10_ESM.zip › EMBOJ-2024-119243R_SourceDataForFigure 2/2C/SW1116-LO-shNT-INVASION.tif]

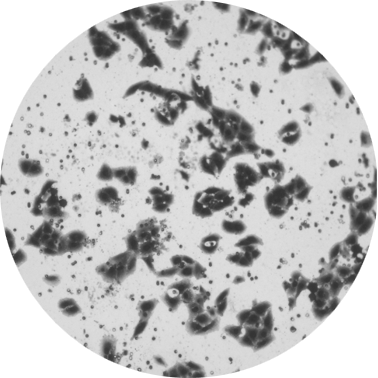

Supplement: Supplementary file 10 — Source data Fig. 2 [file 44318_2025_416_MOESM10_ESM.zip › EMBOJ-2024-119243R_SourceDataForFigure 2/2C/SW1116-LO-shPPA2#1-INVASION.tif]

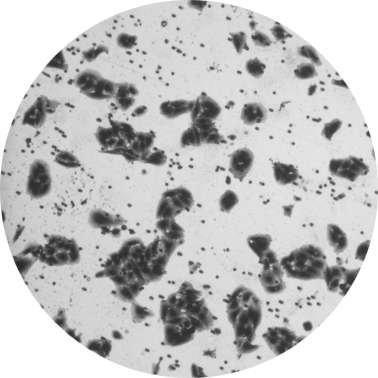

Supplement: Supplementary file 10 — Source data Fig. 2 [file 44318_2025_416_MOESM10_ESM.zip › EMBOJ-2024-119243R_SourceDataForFigure 2/2C/SW1116-LO-shPPA2#2-INVASION.tif]

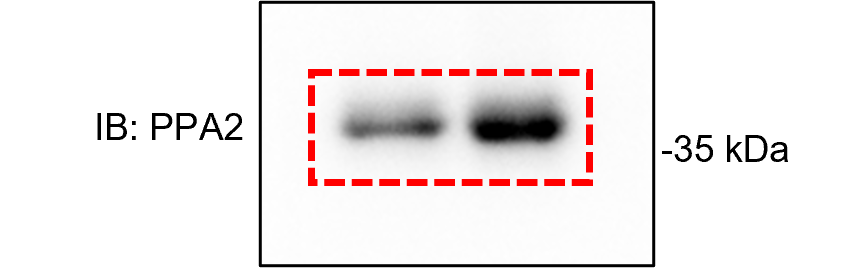

Supplement: Supplementary file 10 — Source data Fig. 2 [file 44318_2025_416_MOESM10_ESM.zip › EMBOJ-2024-119243R_SourceDataForFigure 2/2F/DLD1-PPA2.tif]

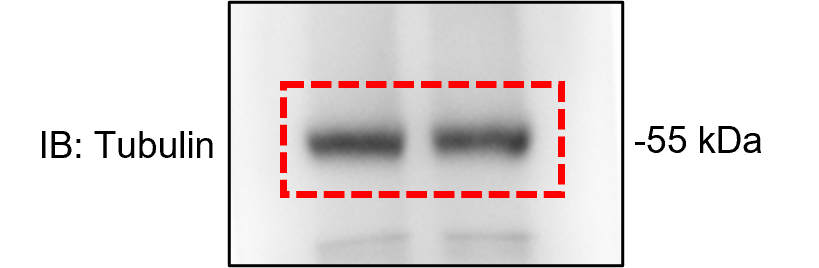

Supplement: Supplementary file 10 — Source data Fig. 2 [file 44318_2025_416_MOESM10_ESM.zip › EMBOJ-2024-119243R_SourceDataForFigure 2/2F/DLD1-Tubulin.tif]

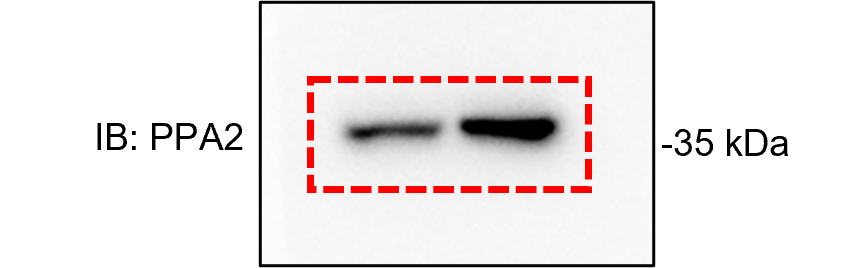

Supplement: Supplementary file 10 — Source data Fig. 2 [file 44318_2025_416_MOESM10_ESM.zip › EMBOJ-2024-119243R_SourceDataForFigure 2/2F/SW1116-PPA2.tif]

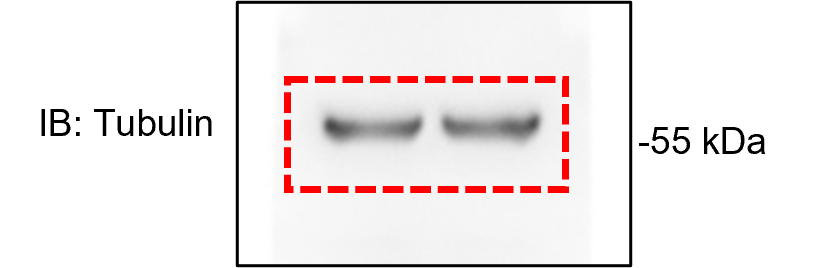

Supplement: Supplementary file 10 — Source data Fig. 2 [file 44318_2025_416_MOESM10_ESM.zip › EMBOJ-2024-119243R_SourceDataForFigure 2/2F/SW1116-Tubulin.tif]

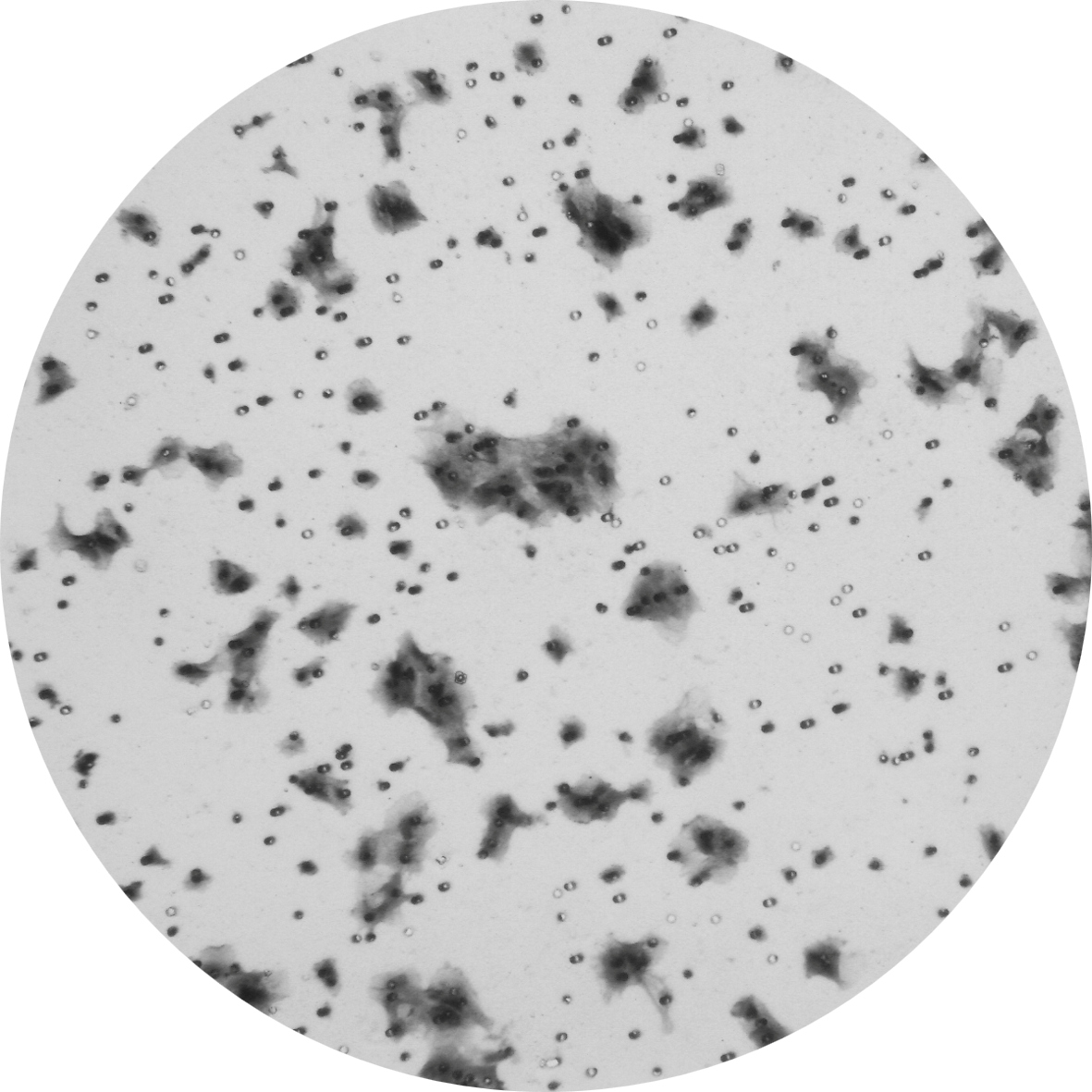

Supplement: Supplementary file 10 — Source data Fig. 2 [file 44318_2025_416_MOESM10_ESM.zip › EMBOJ-2024-119243R_SourceDataForFigure 2/2G/DLD1-HO-PPA2-migration.tif]

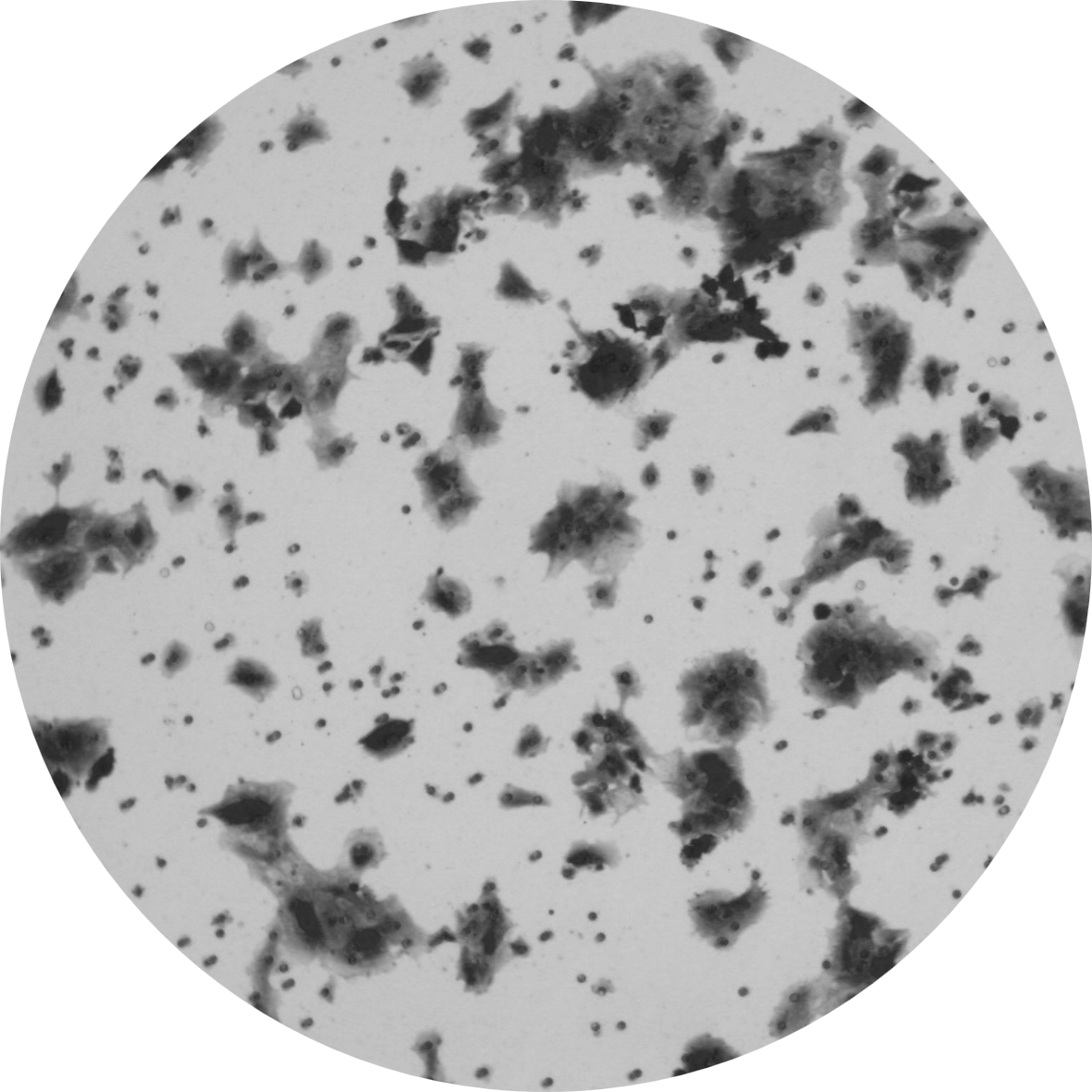

Supplement: Supplementary file 10 — Source data Fig. 2 [file 44318_2025_416_MOESM10_ESM.zip › EMBOJ-2024-119243R_SourceDataForFigure 2/2G/DLD1-HO-Vector-migration.tif]

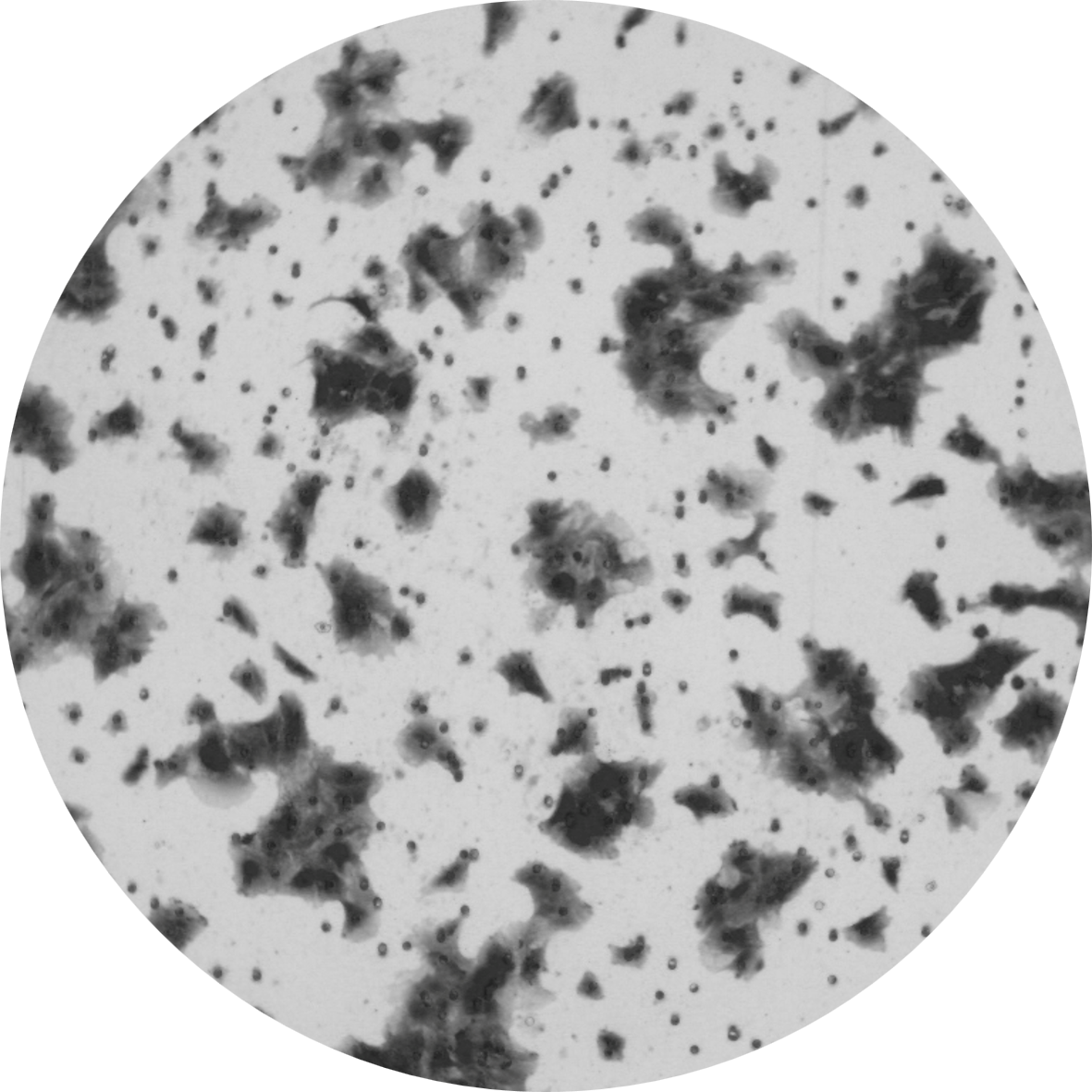

Supplement: Supplementary file 10 — Source data Fig. 2 [file 44318_2025_416_MOESM10_ESM.zip › EMBOJ-2024-119243R_SourceDataForFigure 2/2G/DLD1-LO-PPA2-migration.tif]

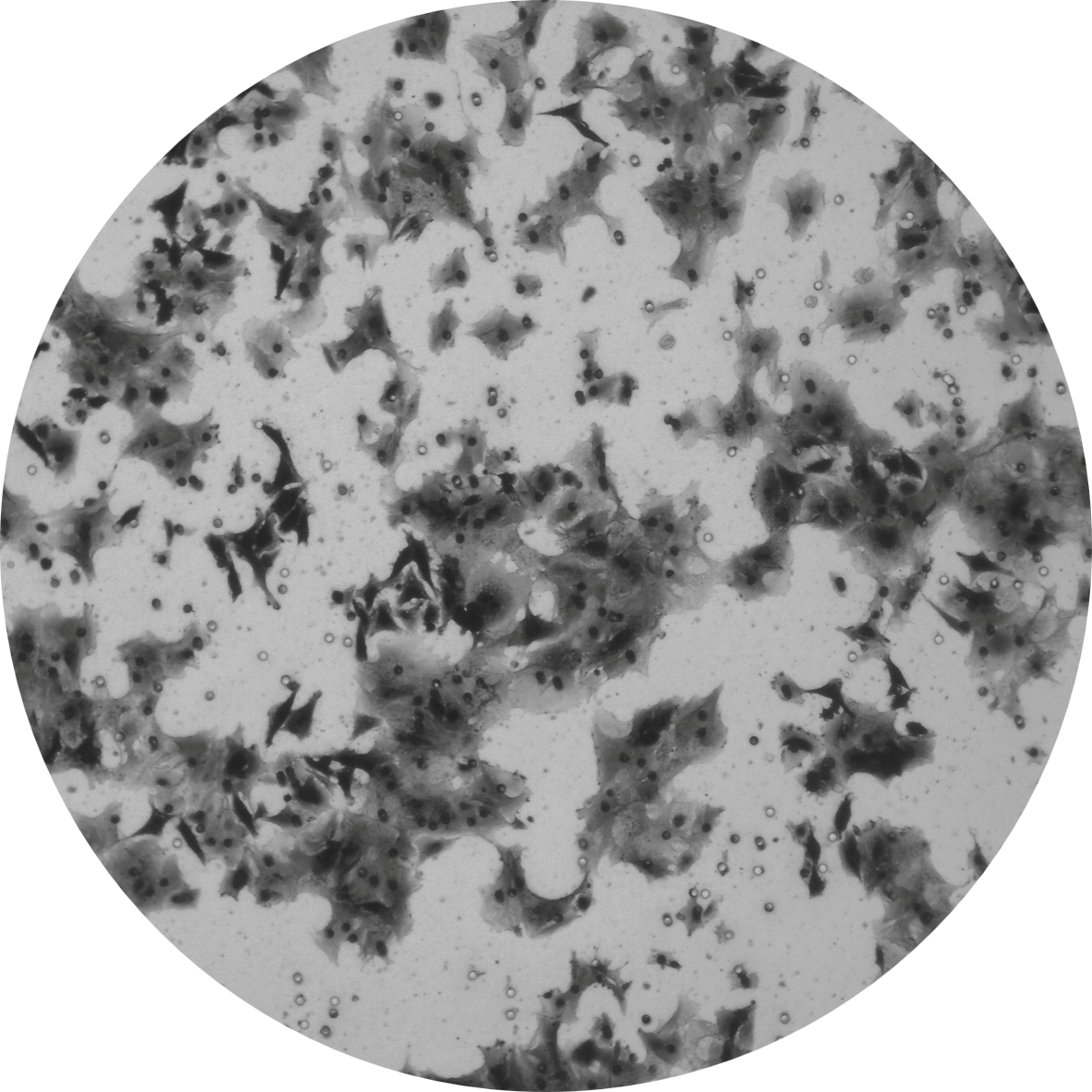

Supplement: Supplementary file 10 — Source data Fig. 2 [file 44318_2025_416_MOESM10_ESM.zip › EMBOJ-2024-119243R_SourceDataForFigure 2/2G/DLD1-LO-Vector-migration.tif]

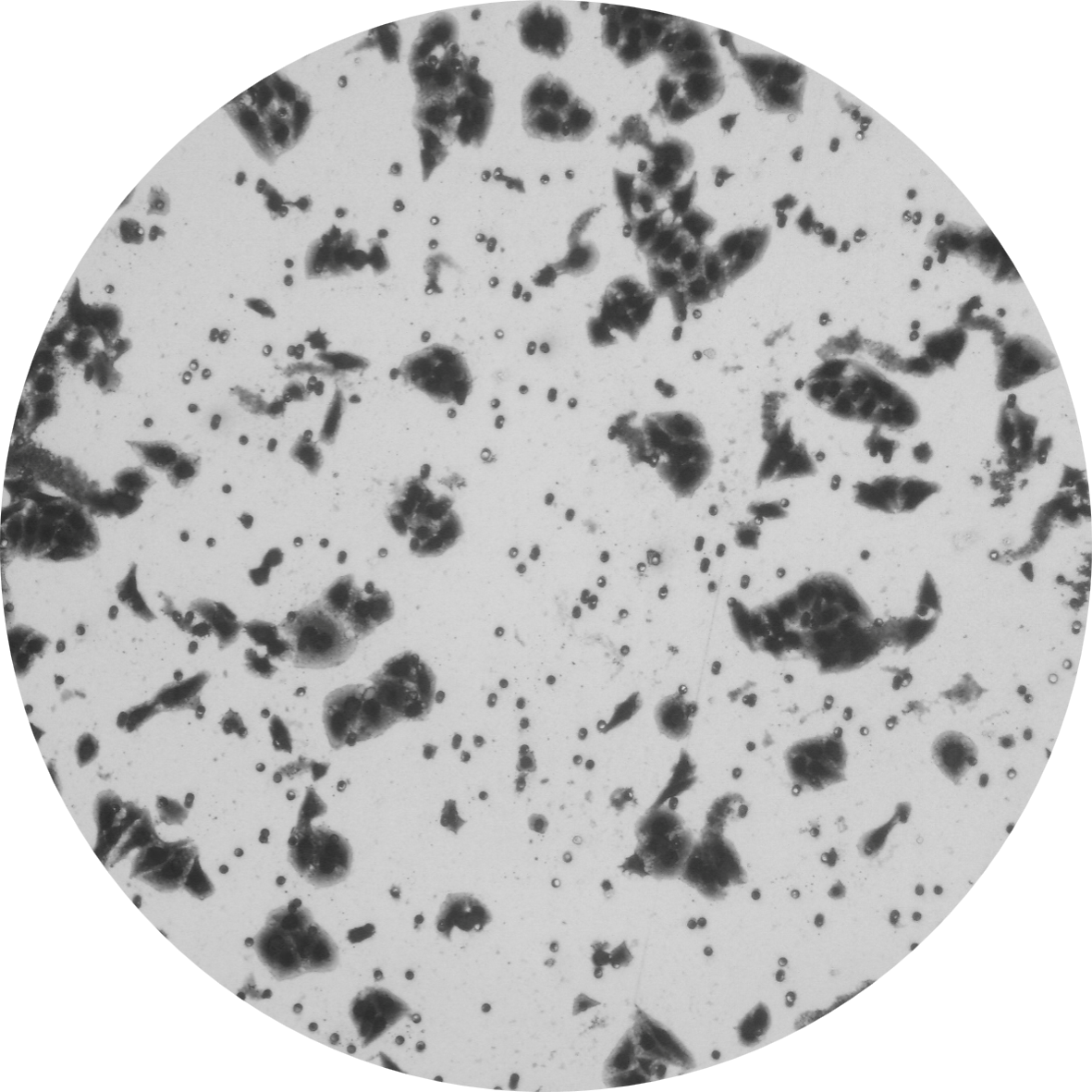

Supplement: Supplementary file 10 — Source data Fig. 2 [file 44318_2025_416_MOESM10_ESM.zip › EMBOJ-2024-119243R_SourceDataForFigure 2/2G/SW1116-HO-PPA2-migration.tif]

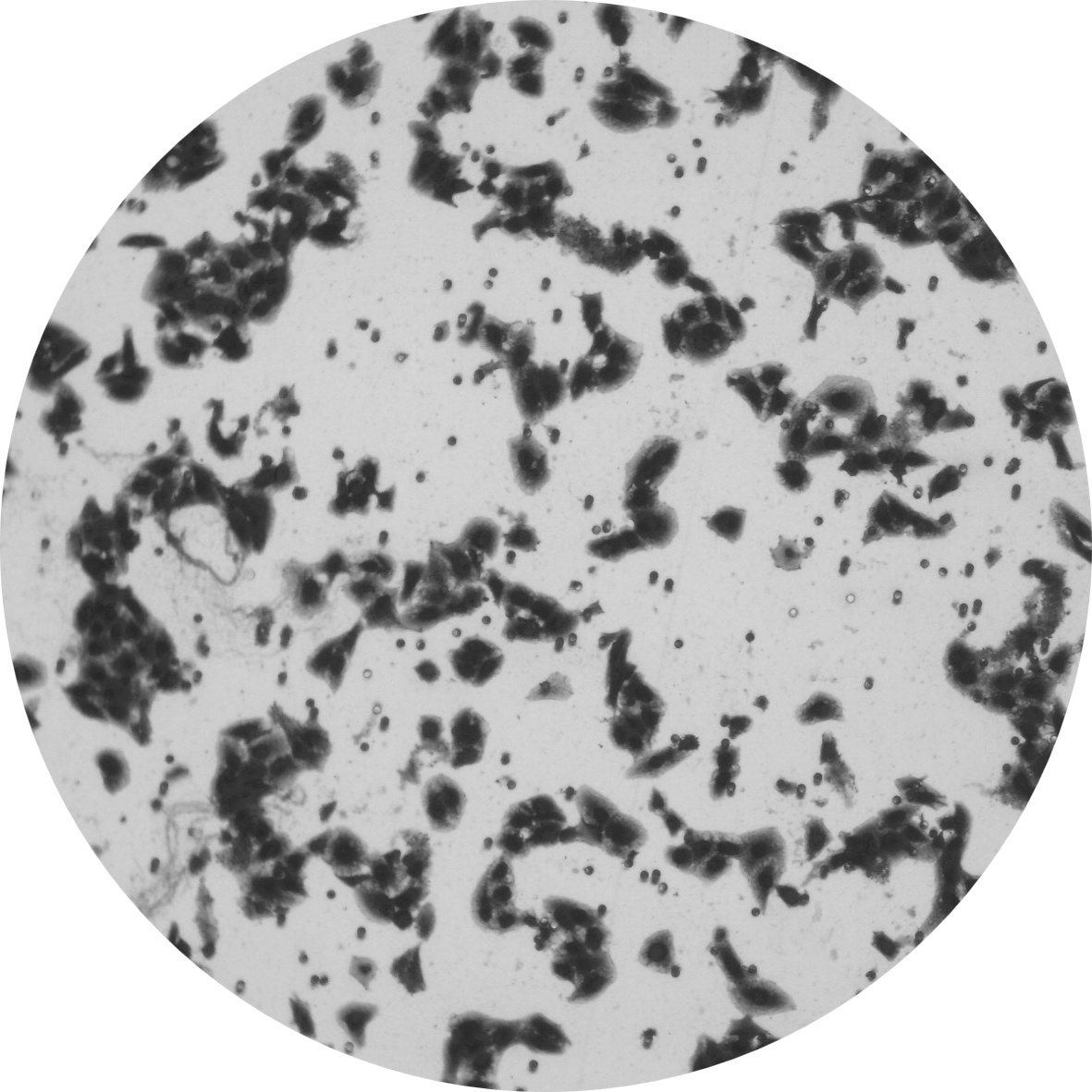

Supplement: Supplementary file 10 — Source data Fig. 2 [file 44318_2025_416_MOESM10_ESM.zip › EMBOJ-2024-119243R_SourceDataForFigure 2/2G/SW1116-HO-Vector-migration.tif]

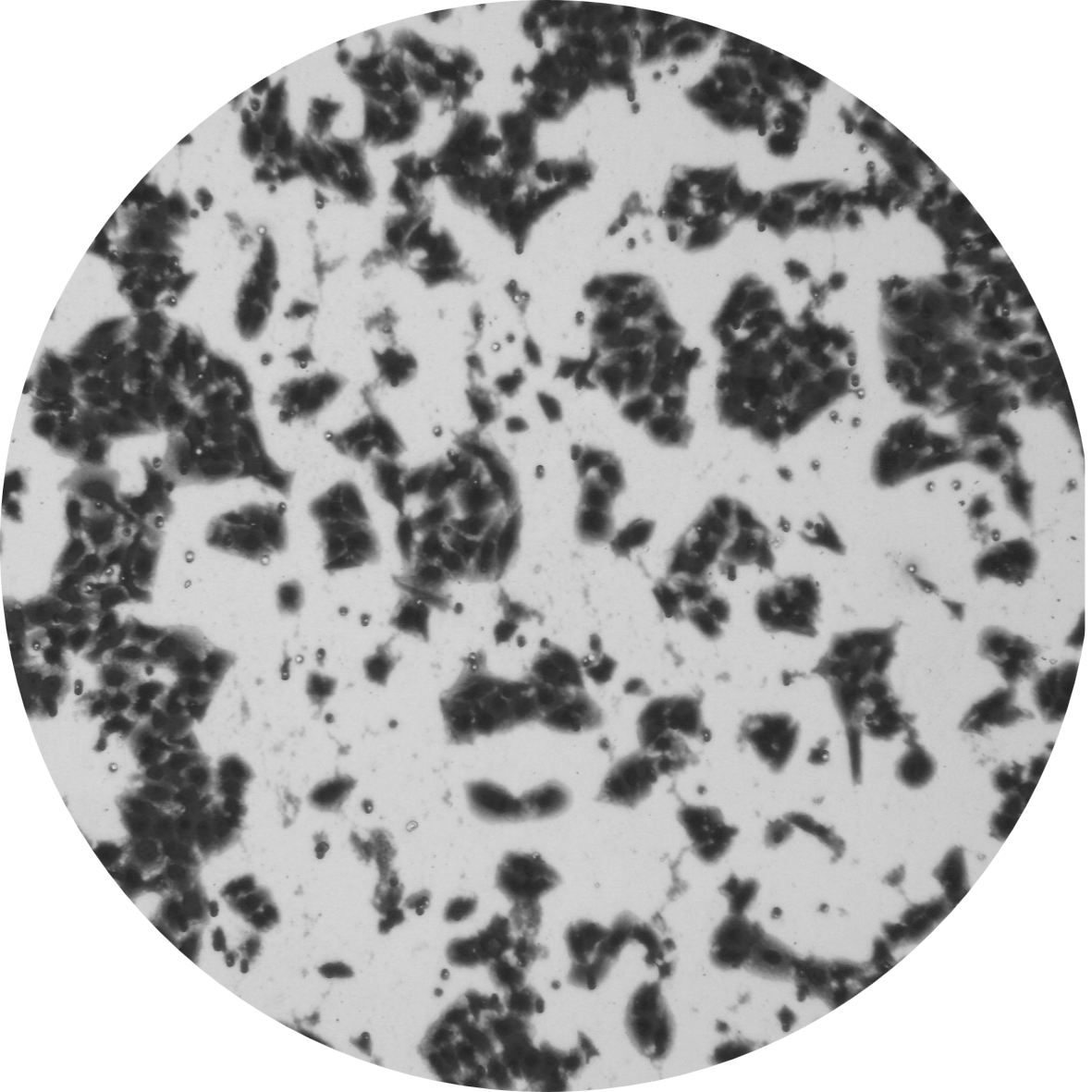

Supplement: Supplementary file 10 — Source data Fig. 2 [file 44318_2025_416_MOESM10_ESM.zip › EMBOJ-2024-119243R_SourceDataForFigure 2/2G/SW1116-LO-PPA2-migration.tif]

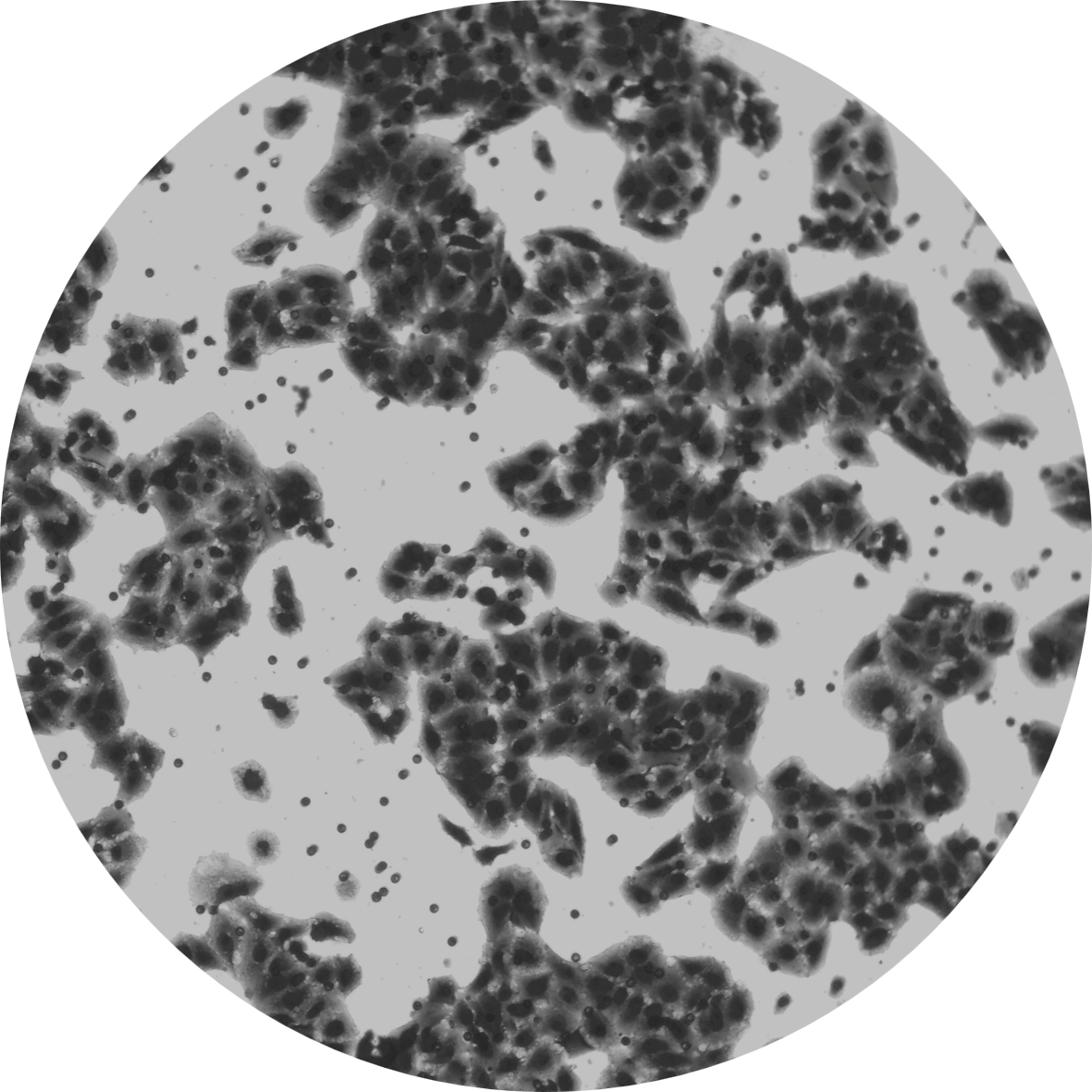

Supplement: Supplementary file 10 — Source data Fig. 2 [file 44318_2025_416_MOESM10_ESM.zip › EMBOJ-2024-119243R_SourceDataForFigure 2/2G/SW1116-LO-Vector-migration.tif]

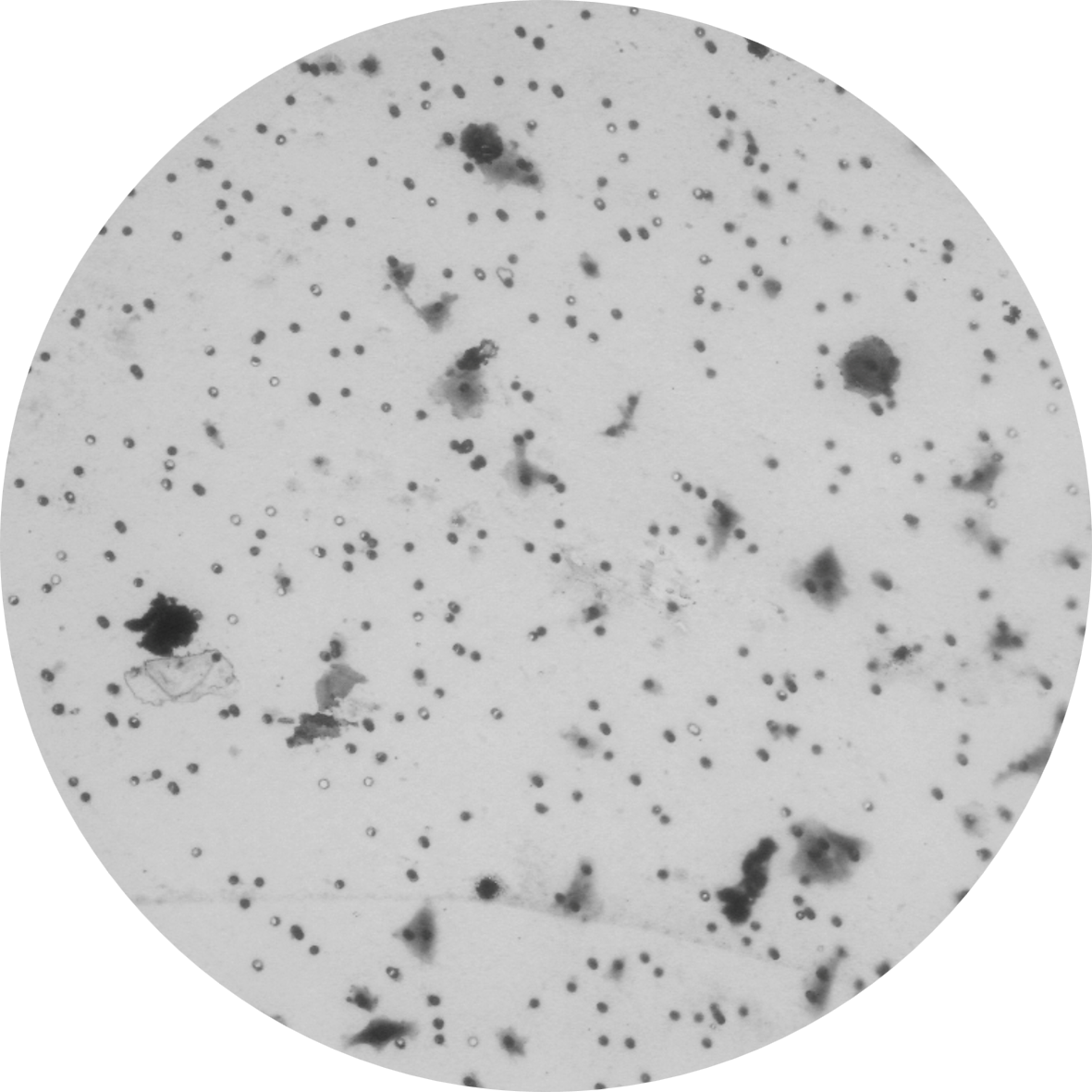

Supplement: Supplementary file 10 — Source data Fig. 2 [file 44318_2025_416_MOESM10_ESM.zip › EMBOJ-2024-119243R_SourceDataForFigure 2/2H/DLD1-HO-PPA2-invasion.tif]

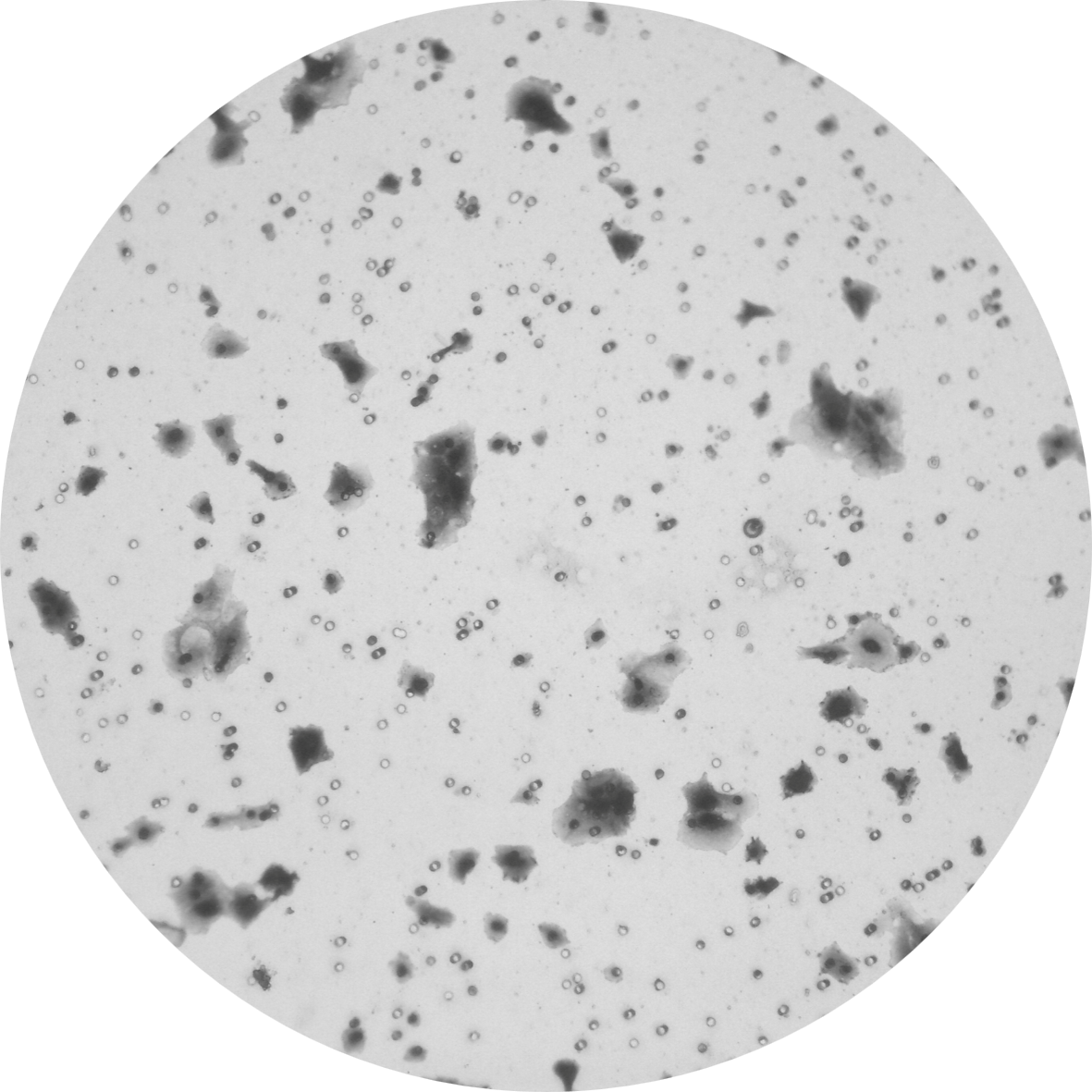

Supplement: Supplementary file 10 — Source data Fig. 2 [file 44318_2025_416_MOESM10_ESM.zip › EMBOJ-2024-119243R_SourceDataForFigure 2/2H/DLD1-HO-Vector-invasion.tif]

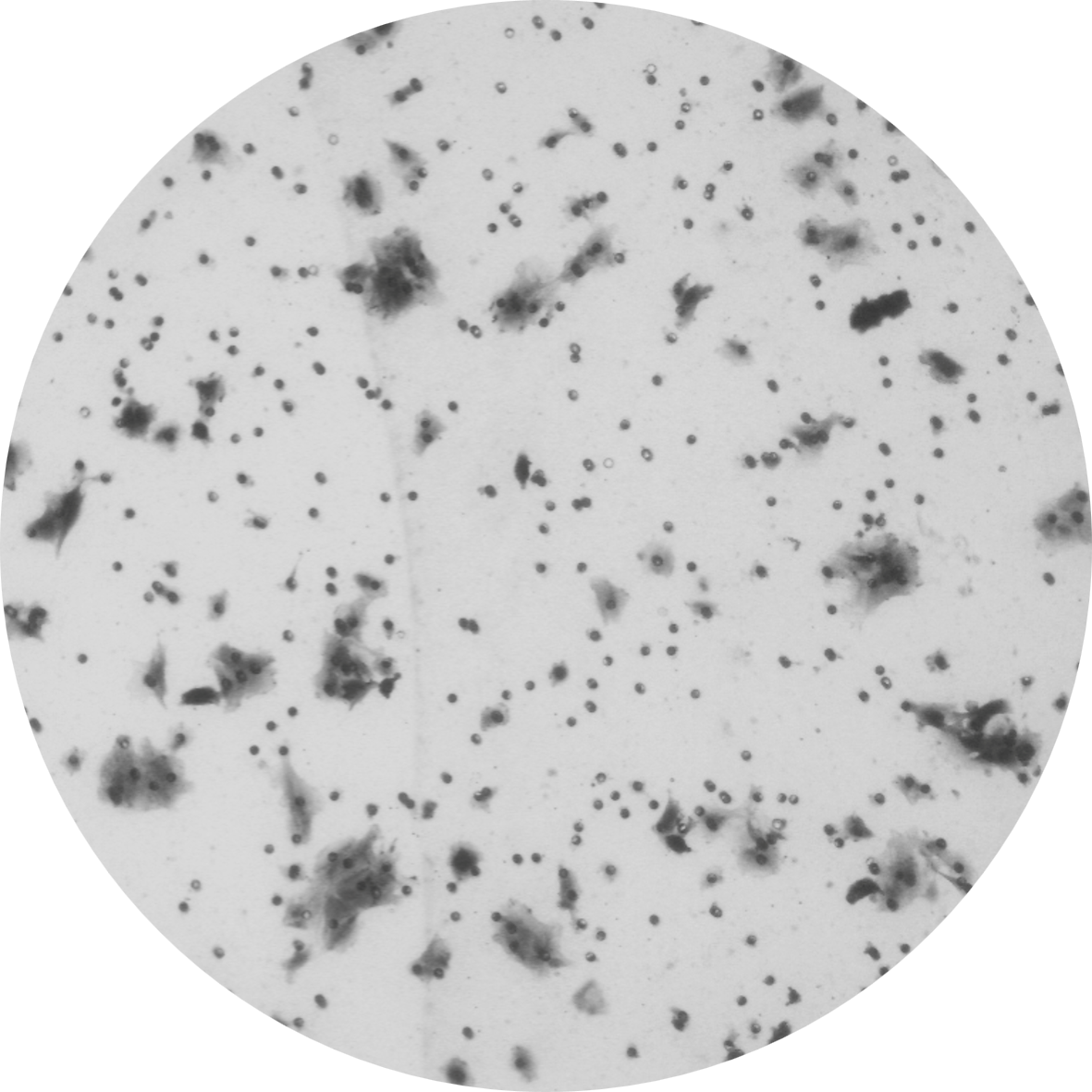

Supplement: Supplementary file 10 — Source data Fig. 2 [file 44318_2025_416_MOESM10_ESM.zip › EMBOJ-2024-119243R_SourceDataForFigure 2/2H/DLD1-LO-PPA2-invasion.tif]

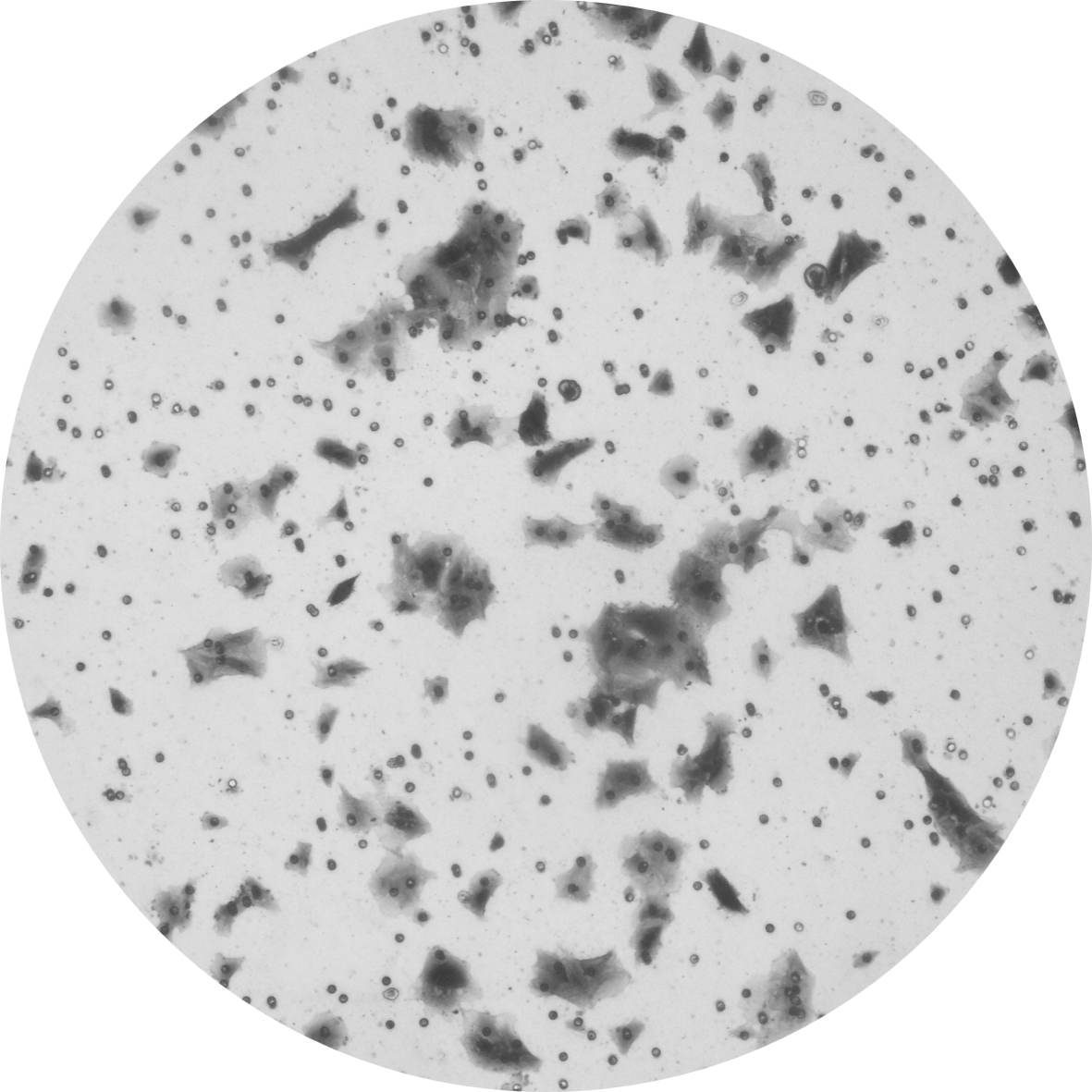

Supplement: Supplementary file 10 — Source data Fig. 2 [file 44318_2025_416_MOESM10_ESM.zip › EMBOJ-2024-119243R_SourceDataForFigure 2/2H/DLD1-LO-Vector-invasion.tif]

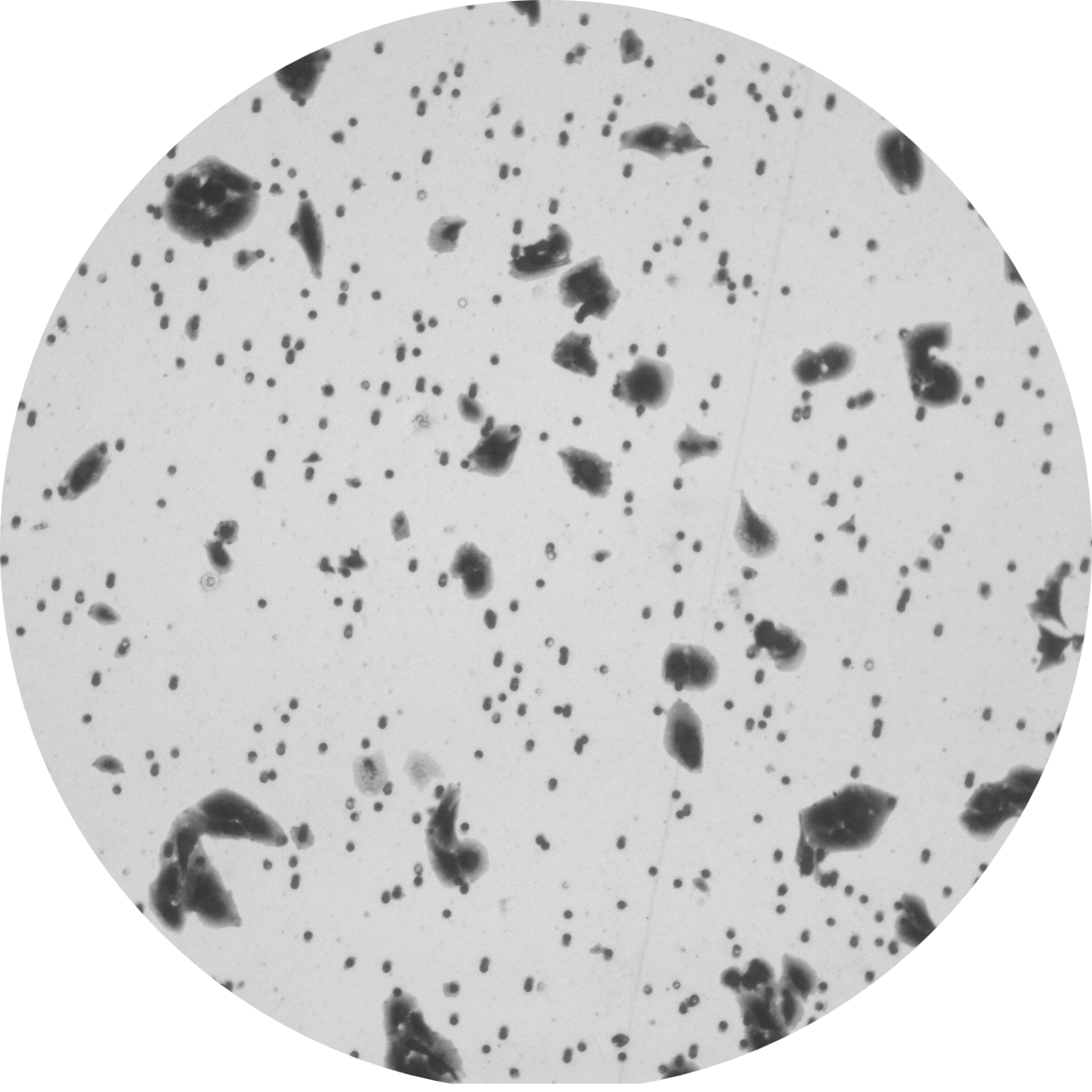

Supplement: Supplementary file 10 — Source data Fig. 2 [file 44318_2025_416_MOESM10_ESM.zip › EMBOJ-2024-119243R_SourceDataForFigure 2/2H/SW1116-HO-PPA2-invasion.tif]

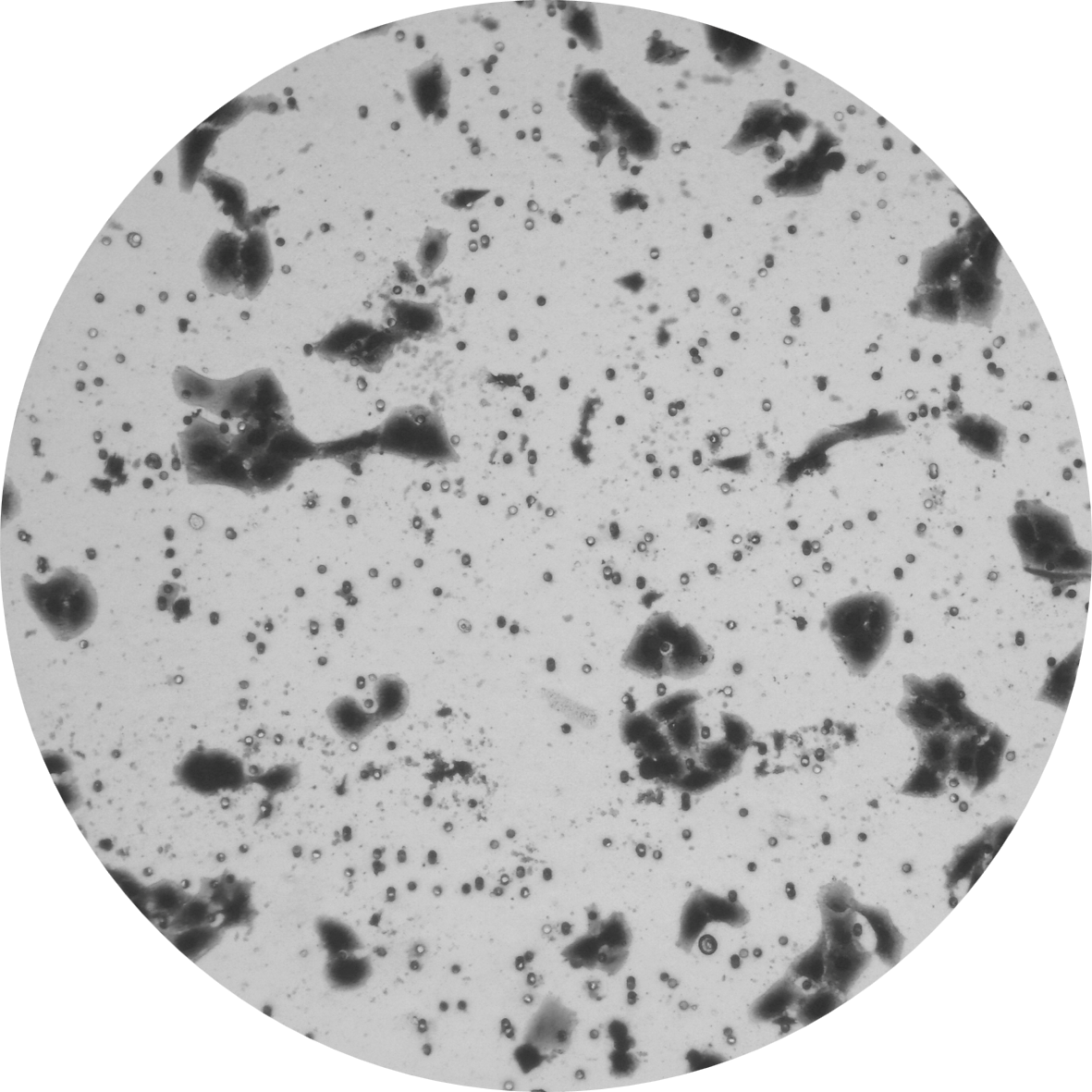

Supplement: Supplementary file 10 — Source data Fig. 2 [file 44318_2025_416_MOESM10_ESM.zip › EMBOJ-2024-119243R_SourceDataForFigure 2/2H/SW1116-HO-Vector-invasion.tif]

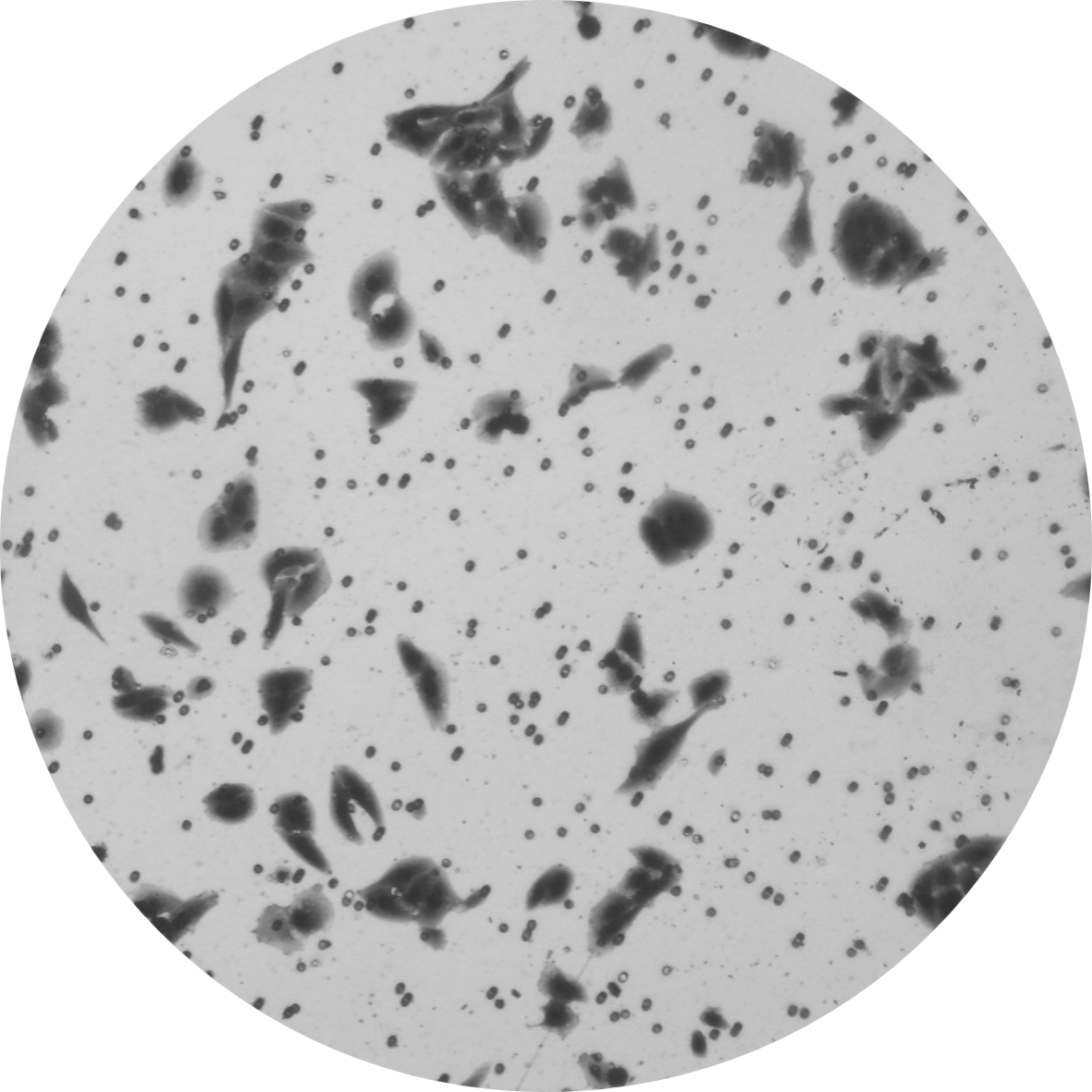

Supplement: Supplementary file 10 — Source data Fig. 2 [file 44318_2025_416_MOESM10_ESM.zip › EMBOJ-2024-119243R_SourceDataForFigure 2/2H/SW1116-LO-PPA2-invasion.tif]

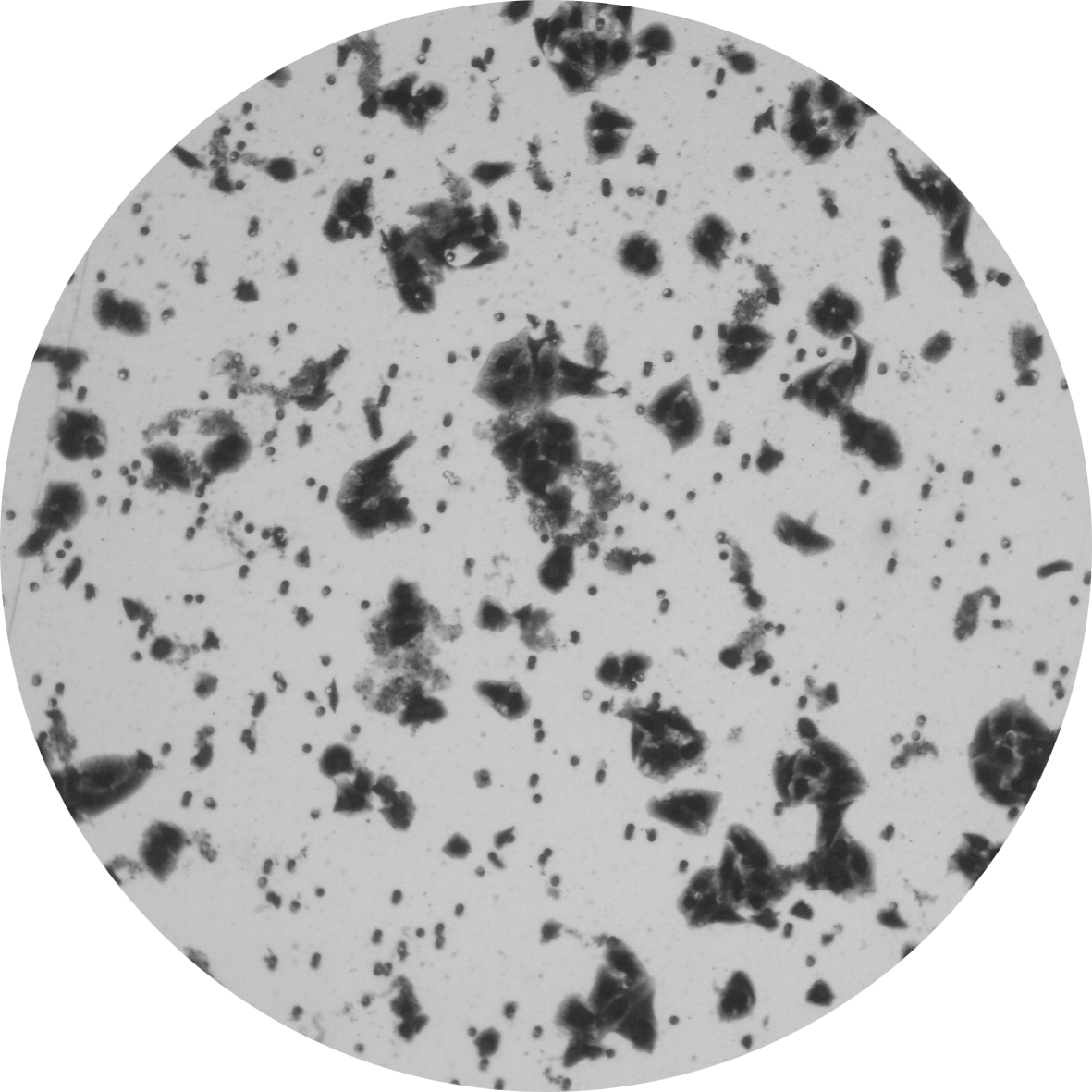

Supplement: Supplementary file 10 — Source data Fig. 2 [file 44318_2025_416_MOESM10_ESM.zip › EMBOJ-2024-119243R_SourceDataForFigure 2/2H/SW1116-LO-Vector-invasion.tif]

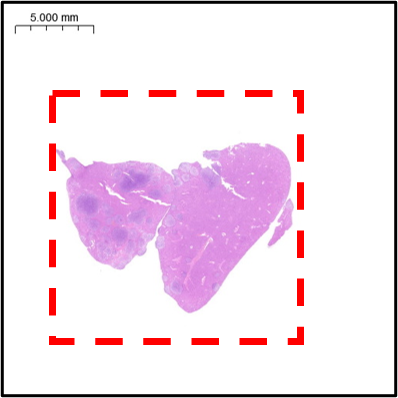

Supplement: Supplementary file 10 — Source data Fig. 2 [file 44318_2025_416_MOESM10_ESM.zip › EMBOJ-2024-119243R_SourceDataForFigure 2/2M/shNT-HE.tif]

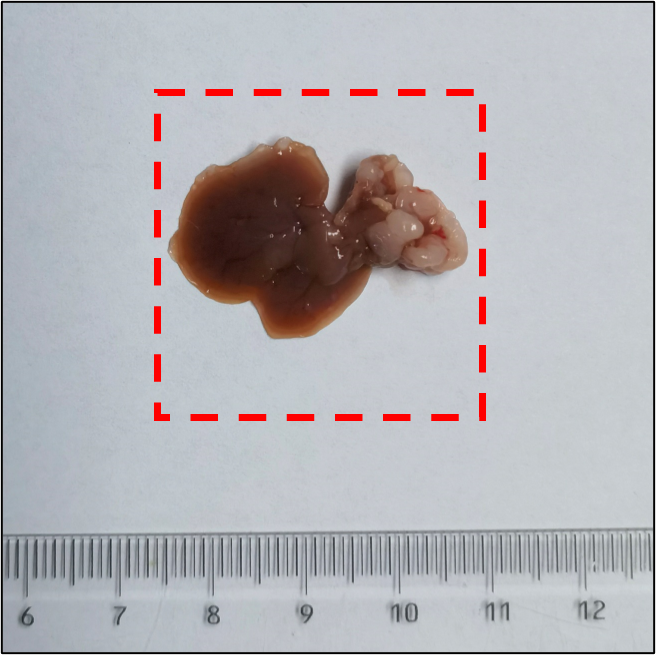

Supplement: Supplementary file 10 — Source data Fig. 2 [file 44318_2025_416_MOESM10_ESM.zip › EMBOJ-2024-119243R_SourceDataForFigure 2/2M/shNT.tif]

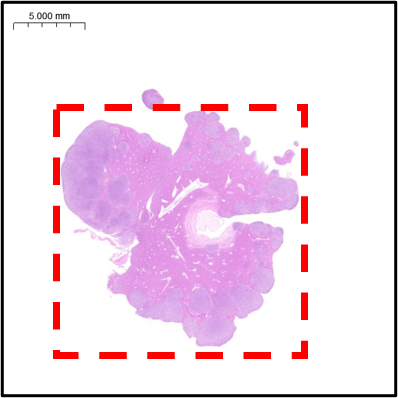

Supplement: Supplementary file 10 — Source data Fig. 2 [file 44318_2025_416_MOESM10_ESM.zip › EMBOJ-2024-119243R_SourceDataForFigure 2/2M/shPPA2#1-HE.tif]

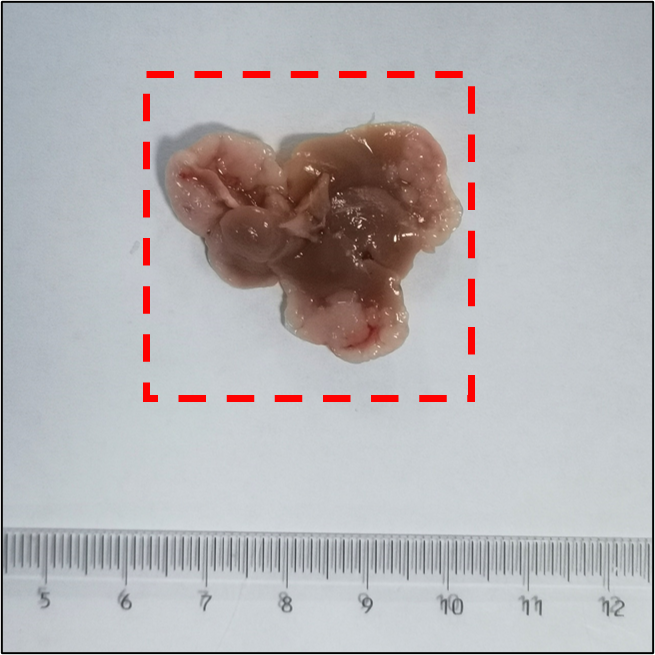

Supplement: Supplementary file 10 — Source data Fig. 2 [file 44318_2025_416_MOESM10_ESM.zip › EMBOJ-2024-119243R_SourceDataForFigure 2/2M/shPPA2#1.tif]

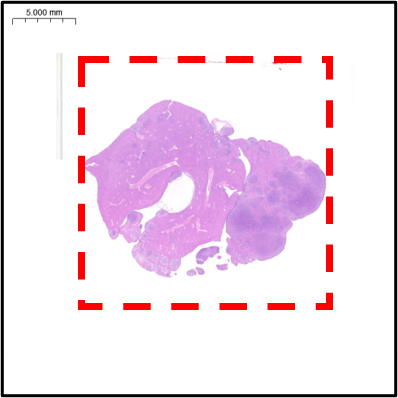

Supplement: Supplementary file 10 — Source data Fig. 2 [file 44318_2025_416_MOESM10_ESM.zip › EMBOJ-2024-119243R_SourceDataForFigure 2/2M/shPPA2#2-HE.tif]

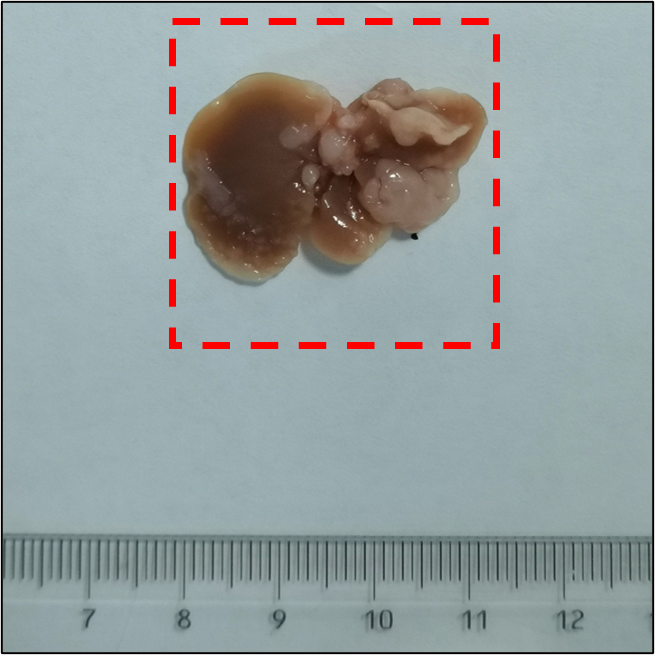

Supplement: Supplementary file 10 — Source data Fig. 2 [file 44318_2025_416_MOESM10_ESM.zip › EMBOJ-2024-119243R_SourceDataForFigure 2/2M/shPPA2#2.tif]

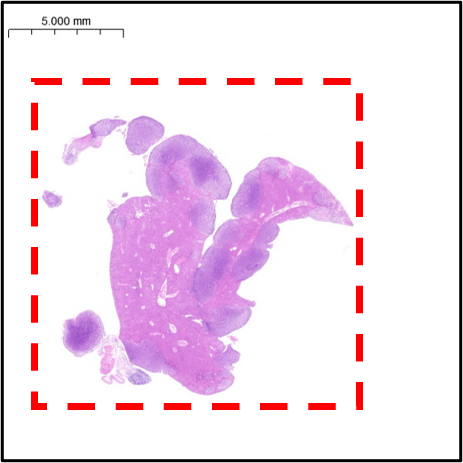

Supplement: Supplementary file 10 — Source data Fig. 2 [file 44318_2025_416_MOESM10_ESM.zip › EMBOJ-2024-119243R_SourceDataForFigure 2/2N/PPA2-ED-HE.tif]

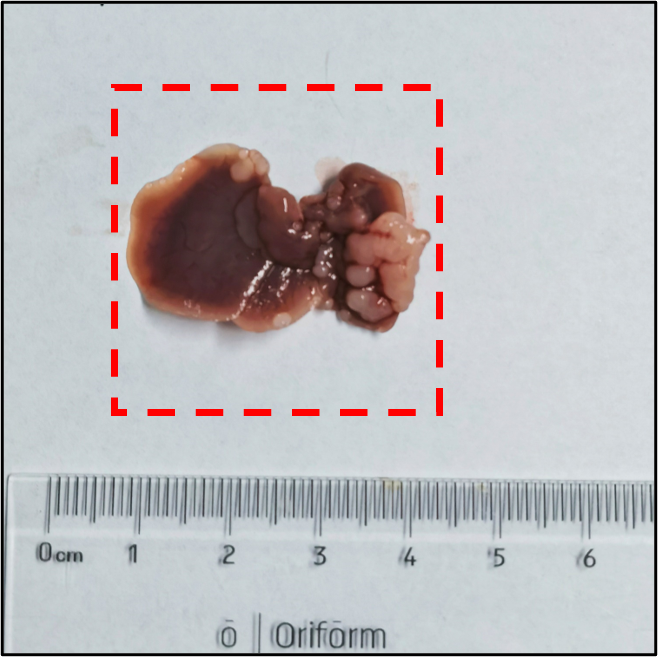

Supplement: Supplementary file 10 — Source data Fig. 2 [file 44318_2025_416_MOESM10_ESM.zip › EMBOJ-2024-119243R_SourceDataForFigure 2/2N/PPA2-ED.tif]

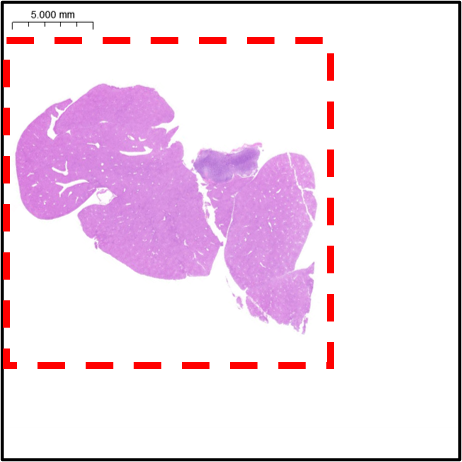

Supplement: Supplementary file 10 — Source data Fig. 2 [file 44318_2025_416_MOESM10_ESM.zip › EMBOJ-2024-119243R_SourceDataForFigure 2/2N/PPA2-WT-HE.tif]

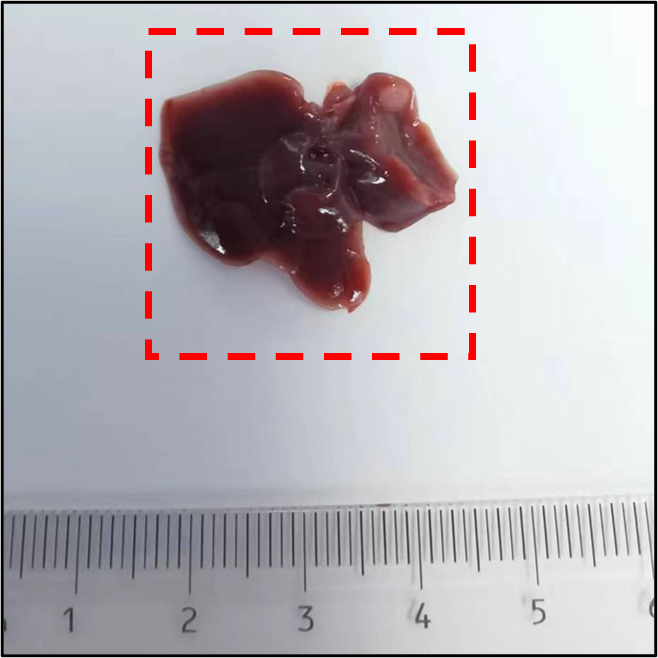

Supplement: Supplementary file 10 — Source data Fig. 2 [file 44318_2025_416_MOESM10_ESM.zip › EMBOJ-2024-119243R_SourceDataForFigure 2/2N/PPA2-WT.tif]

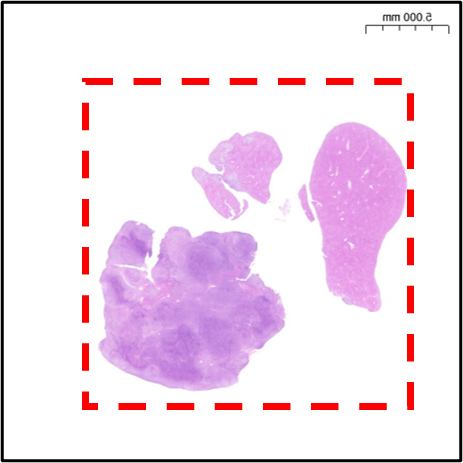

Supplement: Supplementary file 10 — Source data Fig. 2 [file 44318_2025_416_MOESM10_ESM.zip › EMBOJ-2024-119243R_SourceDataForFigure 2/2N/Vector-HE.tif]

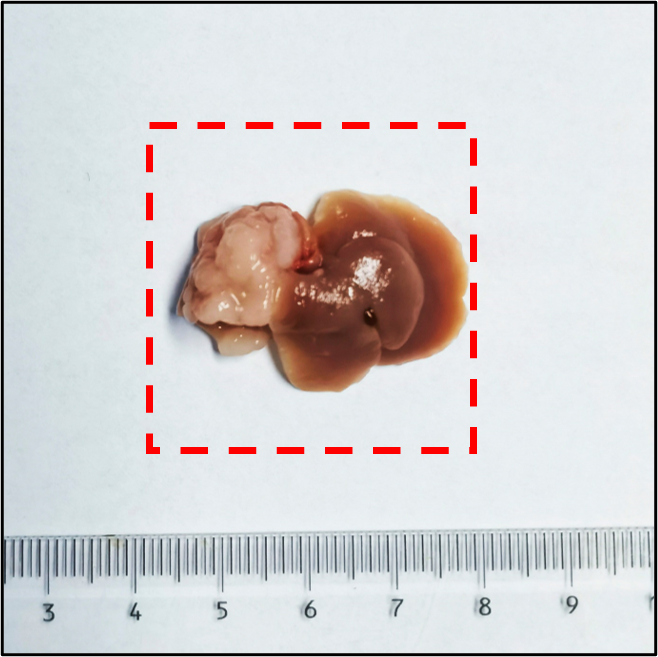

Supplement: Supplementary file 10 — Source data Fig. 2 [file 44318_2025_416_MOESM10_ESM.zip › EMBOJ-2024-119243R_SourceDataForFigure 2/2N/Vector.tif]

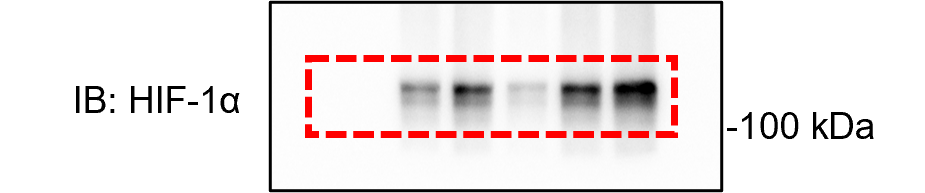

Supplement: Supplementary file 11 — Source data Fig. 3 [file 44318_2025_416_MOESM11_ESM.zip › EMBOJ-2024-119243R_SourceDataForFigure 3/3K/DLD1-HIF-1α.tif]

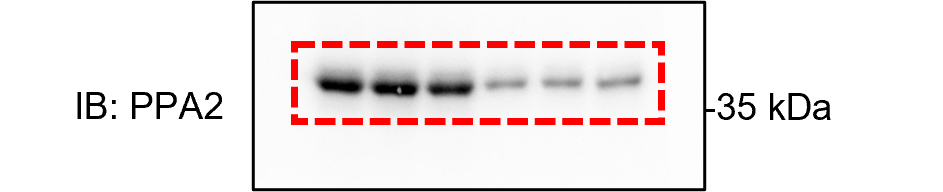

Supplement: Supplementary file 11 — Source data Fig. 3 [file 44318_2025_416_MOESM11_ESM.zip › EMBOJ-2024-119243R_SourceDataForFigure 3/3K/DLD1-PPA2.tif]

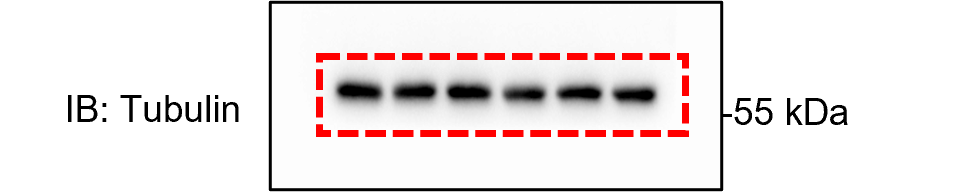

Supplement: Supplementary file 11 — Source data Fig. 3 [file 44318_2025_416_MOESM11_ESM.zip › EMBOJ-2024-119243R_SourceDataForFigure 3/3K/DLD1-Tubulin.tif]

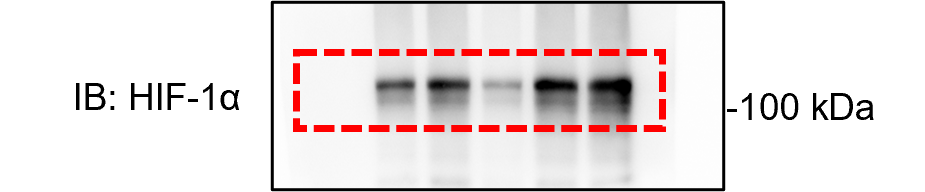

Supplement: Supplementary file 11 — Source data Fig. 3 [file 44318_2025_416_MOESM11_ESM.zip › EMBOJ-2024-119243R_SourceDataForFigure 3/3K/SW1116-HIF-1α.tif]

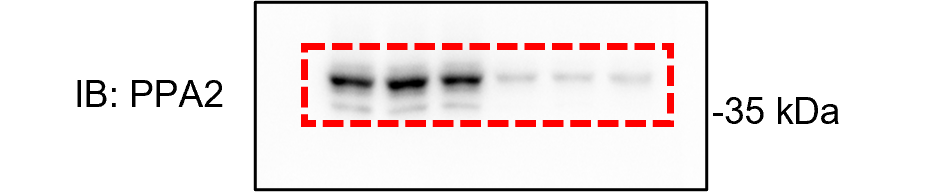

Supplement: Supplementary file 11 — Source data Fig. 3 [file 44318_2025_416_MOESM11_ESM.zip › EMBOJ-2024-119243R_SourceDataForFigure 3/3K/SW1116-PPA2.tif]

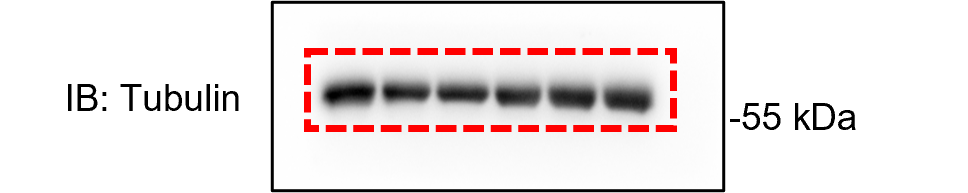

Supplement: Supplementary file 11 — Source data Fig. 3 [file 44318_2025_416_MOESM11_ESM.zip › EMBOJ-2024-119243R_SourceDataForFigure 3/3K/SW1116-Tubulin.tif]

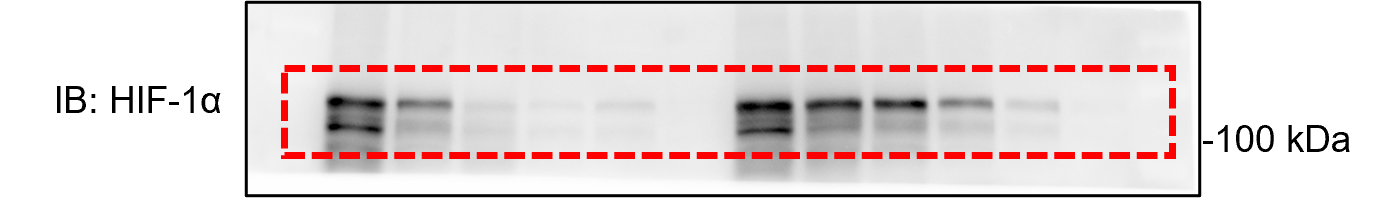

Supplement: Supplementary file 11 — Source data Fig. 3 [file 44318_2025_416_MOESM11_ESM.zip › EMBOJ-2024-119243R_SourceDataForFigure 3/3L/DLD1-HIF-1α.tif]

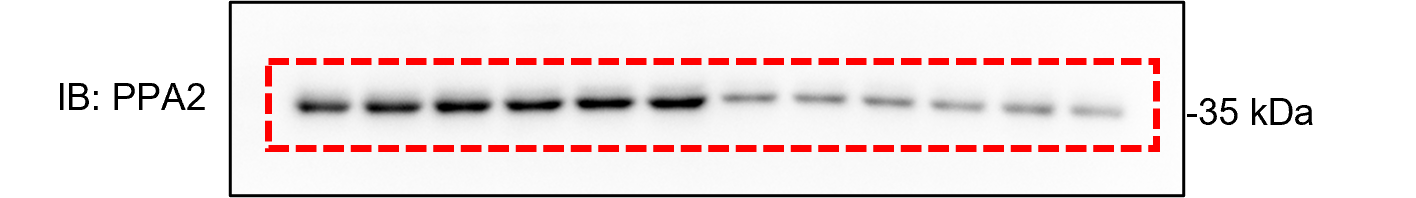

Supplement: Supplementary file 11 — Source data Fig. 3 [file 44318_2025_416_MOESM11_ESM.zip › EMBOJ-2024-119243R_SourceDataForFigure 3/3L/DLD1-PPA2.tif]

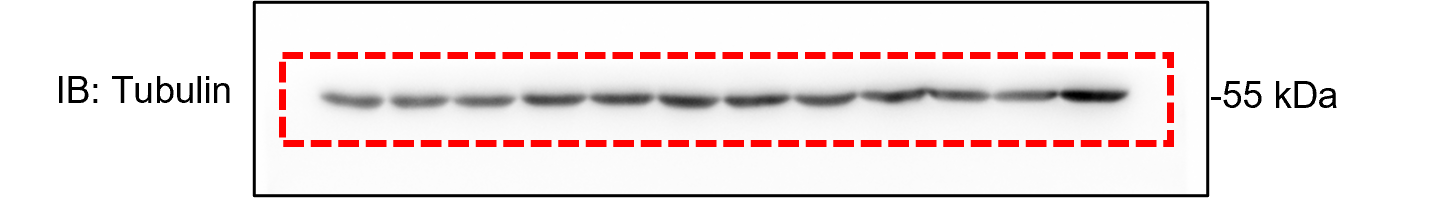

Supplement: Supplementary file 11 — Source data Fig. 3 [file 44318_2025_416_MOESM11_ESM.zip › EMBOJ-2024-119243R_SourceDataForFigure 3/3L/DLD1-Tubulin.tif]

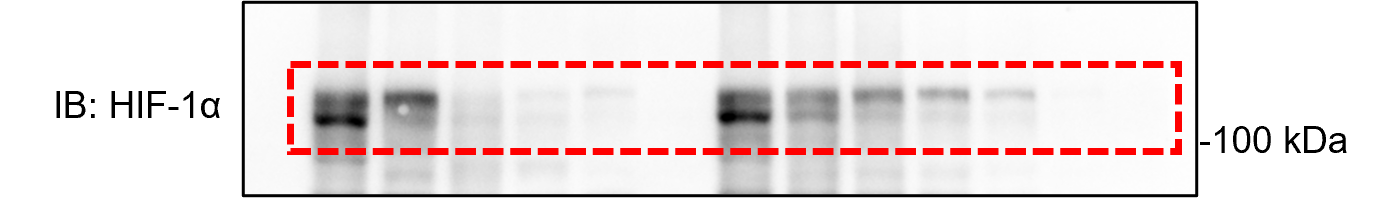

Supplement: Supplementary file 11 — Source data Fig. 3 [file 44318_2025_416_MOESM11_ESM.zip › EMBOJ-2024-119243R_SourceDataForFigure 3/3L/SW1116-HIF-1α.tif]

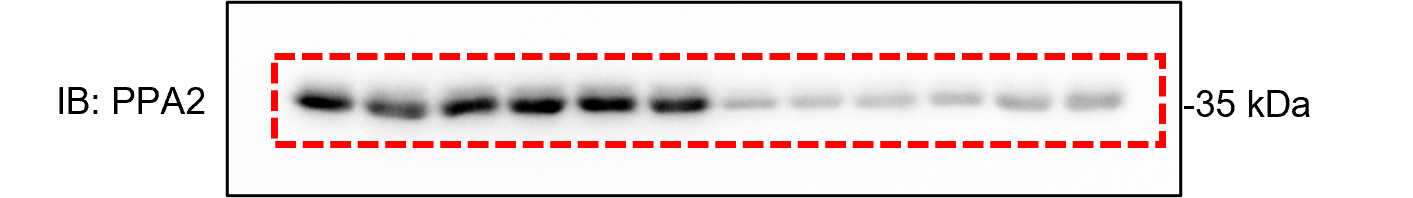

Supplement: Supplementary file 11 — Source data Fig. 3 [file 44318_2025_416_MOESM11_ESM.zip › EMBOJ-2024-119243R_SourceDataForFigure 3/3L/SW1116-PPA2.tif]

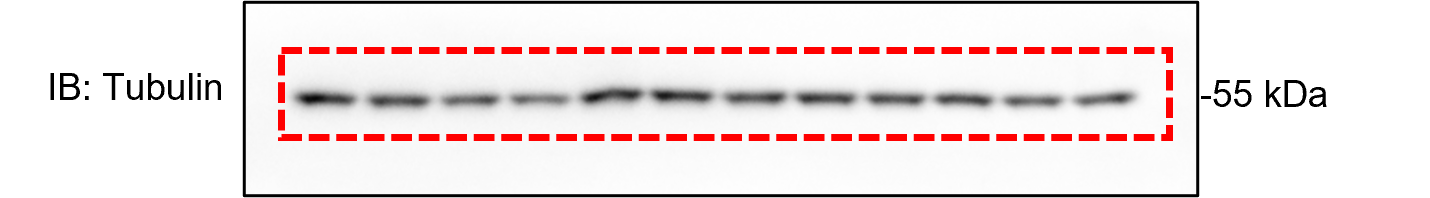

Supplement: Supplementary file 11 — Source data Fig. 3 [file 44318_2025_416_MOESM11_ESM.zip › EMBOJ-2024-119243R_SourceDataForFigure 3/3L/SW1116-Tubulin.tif]

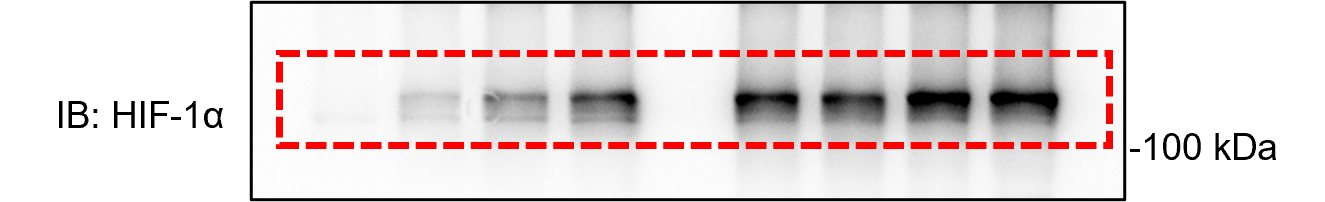

Supplement: Supplementary file 11 — Source data Fig. 3 [file 44318_2025_416_MOESM11_ESM.zip › EMBOJ-2024-119243R_SourceDataForFigure 3/3M/DLD1-HIF-1α.tif]

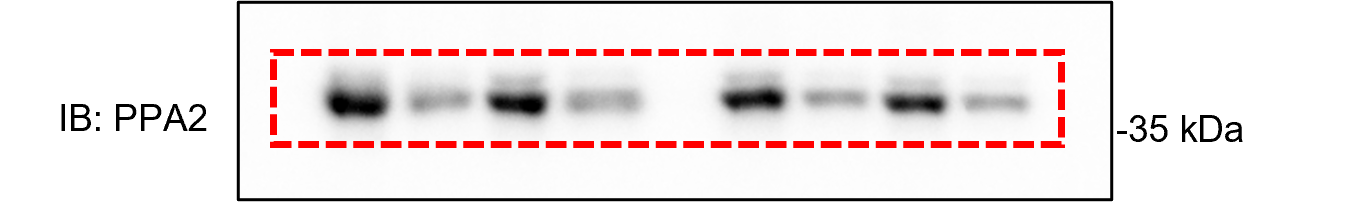

Supplement: Supplementary file 11 — Source data Fig. 3 [file 44318_2025_416_MOESM11_ESM.zip › EMBOJ-2024-119243R_SourceDataForFigure 3/3M/DLD1-PPA2.tif]

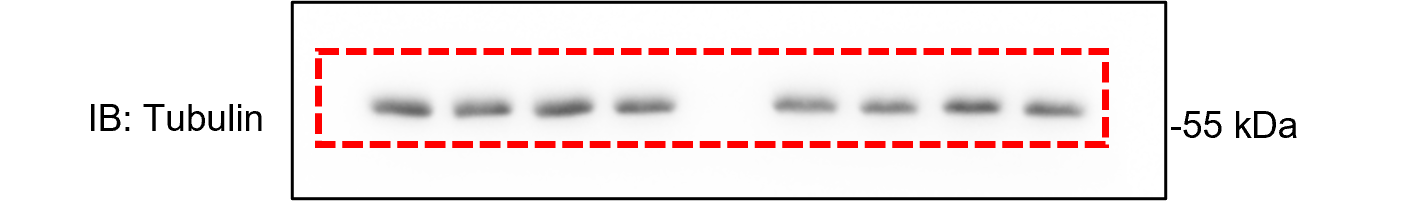

Supplement: Supplementary file 11 — Source data Fig. 3 [file 44318_2025_416_MOESM11_ESM.zip › EMBOJ-2024-119243R_SourceDataForFigure 3/3M/DLD1-Tubulin.tif]

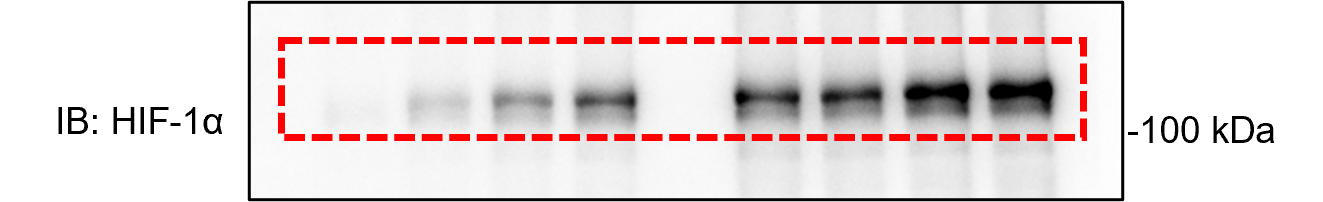

Supplement: Supplementary file 11 — Source data Fig. 3 [file 44318_2025_416_MOESM11_ESM.zip › EMBOJ-2024-119243R_SourceDataForFigure 3/3M/SW1116-HIF-1α.tif]

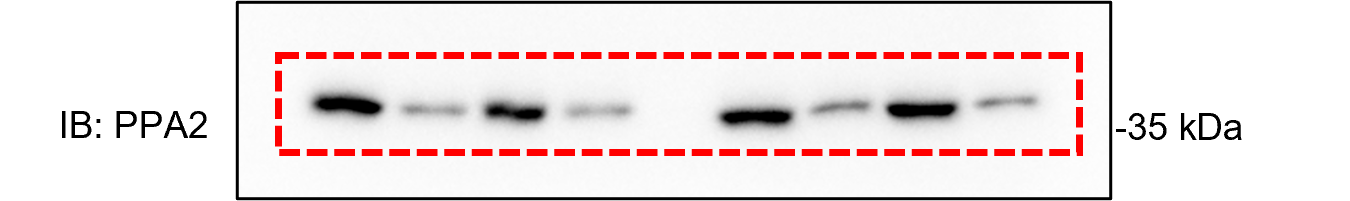

Supplement: Supplementary file 11 — Source data Fig. 3 [file 44318_2025_416_MOESM11_ESM.zip › EMBOJ-2024-119243R_SourceDataForFigure 3/3M/SW1116-PPA2.tif]

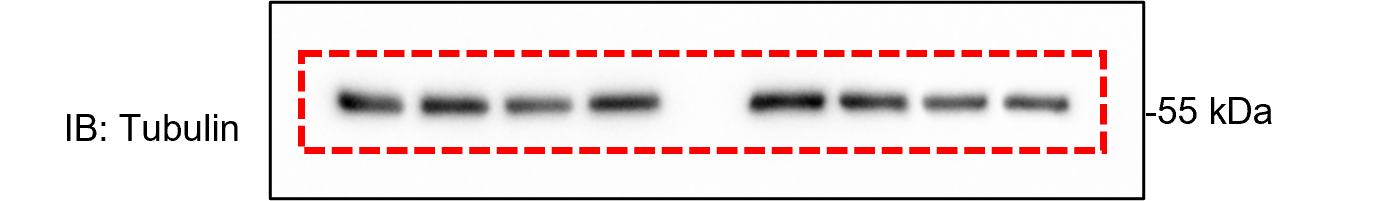

Supplement: Supplementary file 11 — Source data Fig. 3 [file 44318_2025_416_MOESM11_ESM.zip › EMBOJ-2024-119243R_SourceDataForFigure 3/3M/SW1116-Tubulin.tif]

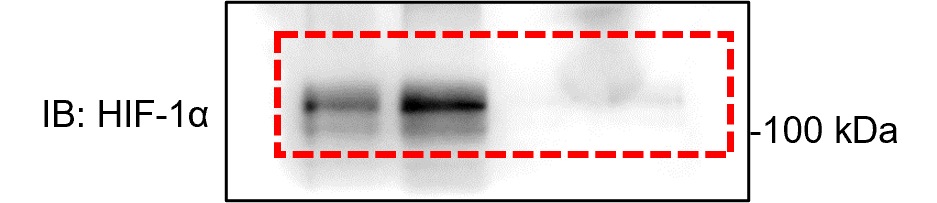

Supplement: Supplementary file 11 — Source data Fig. 3 [file 44318_2025_416_MOESM11_ESM.zip › EMBOJ-2024-119243R_SourceDataForFigure 3/3O/DLD1-HIF-1α.tif]

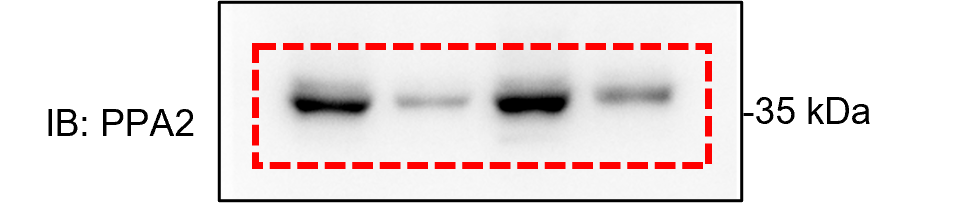

Supplement: Supplementary file 11 — Source data Fig. 3 [file 44318_2025_416_MOESM11_ESM.zip › EMBOJ-2024-119243R_SourceDataForFigure 3/3O/DLD1-PPA2.tif]

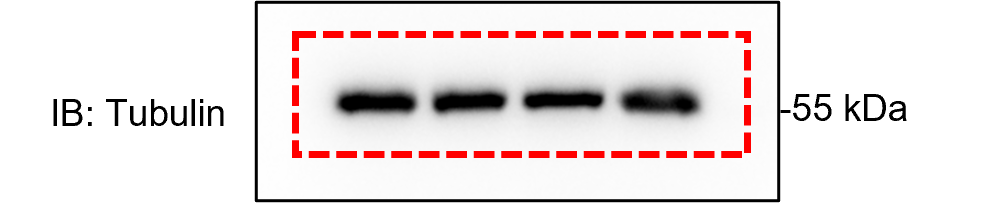

Supplement: Supplementary file 11 — Source data Fig. 3 [file 44318_2025_416_MOESM11_ESM.zip › EMBOJ-2024-119243R_SourceDataForFigure 3/3O/DLD1-Tubulin.tif]

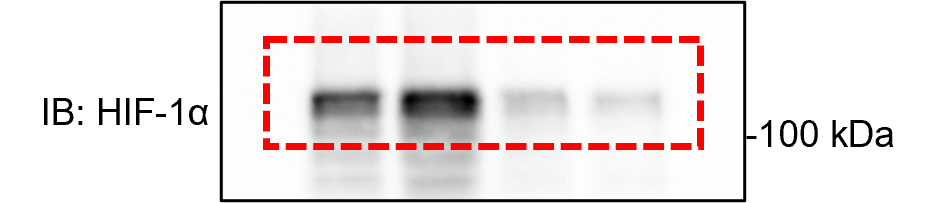

Supplement: Supplementary file 11 — Source data Fig. 3 [file 44318_2025_416_MOESM11_ESM.zip › EMBOJ-2024-119243R_SourceDataForFigure 3/3O/SW1116-HIF-1α.tif]

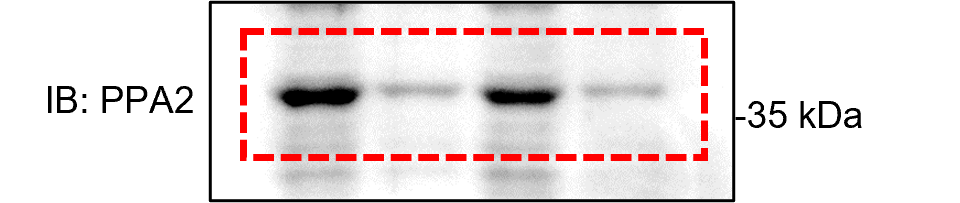

Supplement: Supplementary file 11 — Source data Fig. 3 [file 44318_2025_416_MOESM11_ESM.zip › EMBOJ-2024-119243R_SourceDataForFigure 3/3O/SW1116-PPA2.tif]

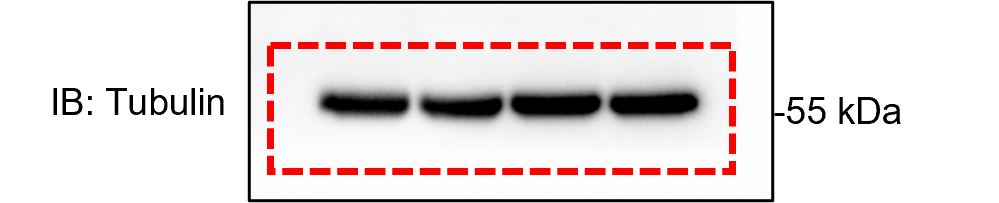

Supplement: Supplementary file 11 — Source data Fig. 3 [file 44318_2025_416_MOESM11_ESM.zip › EMBOJ-2024-119243R_SourceDataForFigure 3/3O/SW1116-Tubulin.tif]

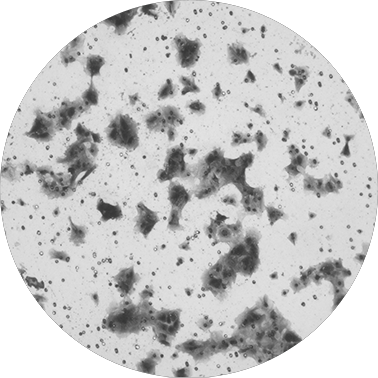

Supplement: Supplementary file 11 — Source data Fig. 3 [file 44318_2025_416_MOESM11_ESM.zip › EMBOJ-2024-119243R_SourceDataForFigure 3/3Q/DLD1-sgCtrl-shNT.tif]

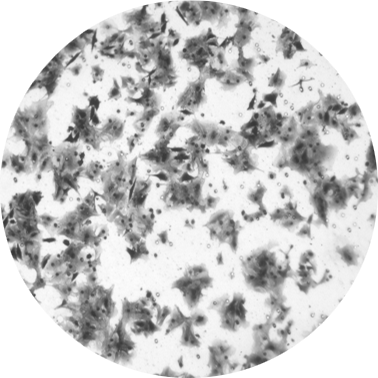

Supplement: Supplementary file 11 — Source data Fig. 3 [file 44318_2025_416_MOESM11_ESM.zip › EMBOJ-2024-119243R_SourceDataForFigure 3/3Q/DLD1-sgCtrl-shPPA2.tif]
