# Supplementary figures and images for: SIRT5-mediated desuccinylation of PPA2 enhances HIF-1alpha-dependent adaptation to hypoxic stress and colorectal cancer metastasis (part 2 of 5)
Source: EMBO J. 2025 Mar 31;44(9):2514–40. doi: 10.1038/s44318-025-00416-1 (PMC12048626; doi:10.1038/s44318-025-00416-1)

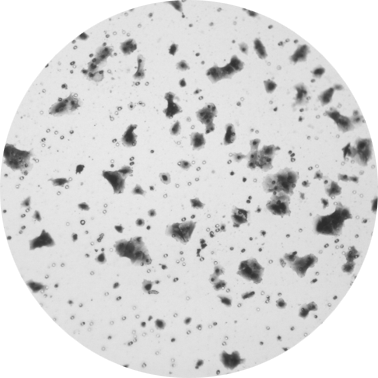

Supplement: Supplementary file 11 — Source data Fig. 3 [file 44318_2025_416_MOESM11_ESM.zip › EMBOJ-2024-119243R_SourceDataForFigure 3/3Q/DLD1-sgHIF-1α-shNT.tif]

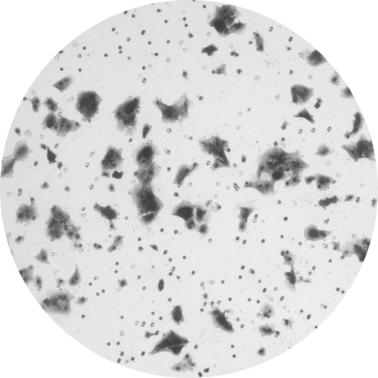

Supplement: Supplementary file 11 — Source data Fig. 3 [file 44318_2025_416_MOESM11_ESM.zip › EMBOJ-2024-119243R_SourceDataForFigure 3/3Q/DLD1-sgHIF-1α-shPPA2.tif]

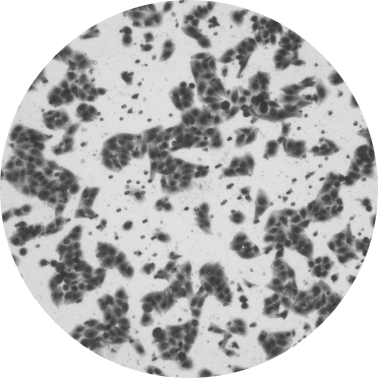

Supplement: Supplementary file 11 — Source data Fig. 3 [file 44318_2025_416_MOESM11_ESM.zip › EMBOJ-2024-119243R_SourceDataForFigure 3/3Q/SW1116-sgCtrl-shNT.tif]

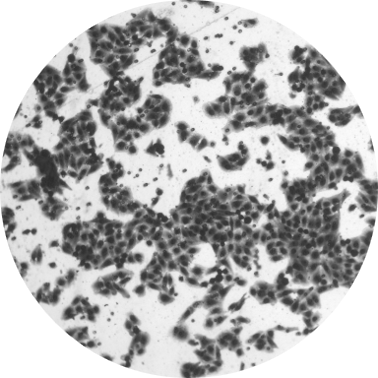

Supplement: Supplementary file 11 — Source data Fig. 3 [file 44318_2025_416_MOESM11_ESM.zip › EMBOJ-2024-119243R_SourceDataForFigure 3/3Q/SW1116-sgCtrl-shPPA2.tif]

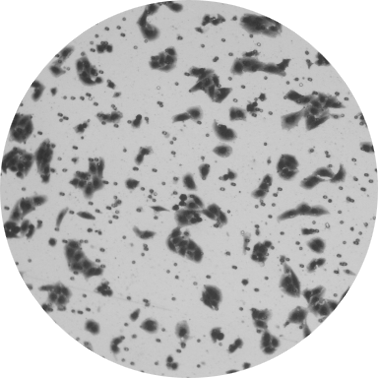

Supplement: Supplementary file 11 — Source data Fig. 3 [file 44318_2025_416_MOESM11_ESM.zip › EMBOJ-2024-119243R_SourceDataForFigure 3/3Q/SW1116-sgHIF-1α-shNT.tif]

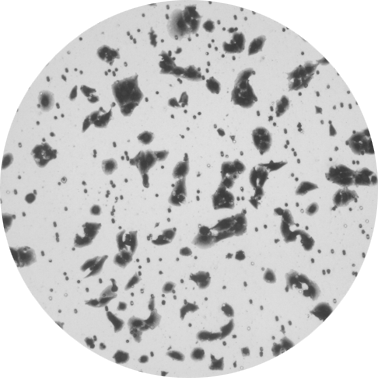

Supplement: Supplementary file 11 — Source data Fig. 3 [file 44318_2025_416_MOESM11_ESM.zip › EMBOJ-2024-119243R_SourceDataForFigure 3/3Q/SW1116-sgHIF-1α-shPPA2.tif]

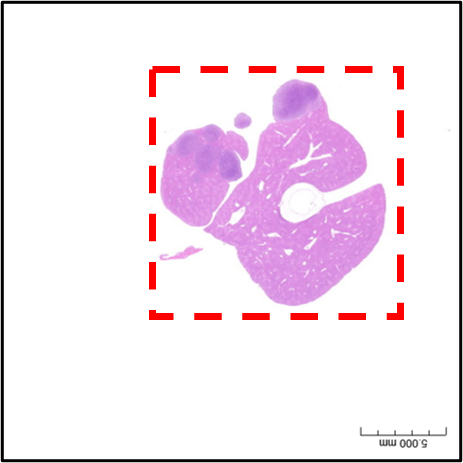

Supplement: Supplementary file 11 — Source data Fig. 3 [file 44318_2025_416_MOESM11_ESM.zip › EMBOJ-2024-119243R_SourceDataForFigure 3/3U/shNT-sgCtrl-HE.tif]

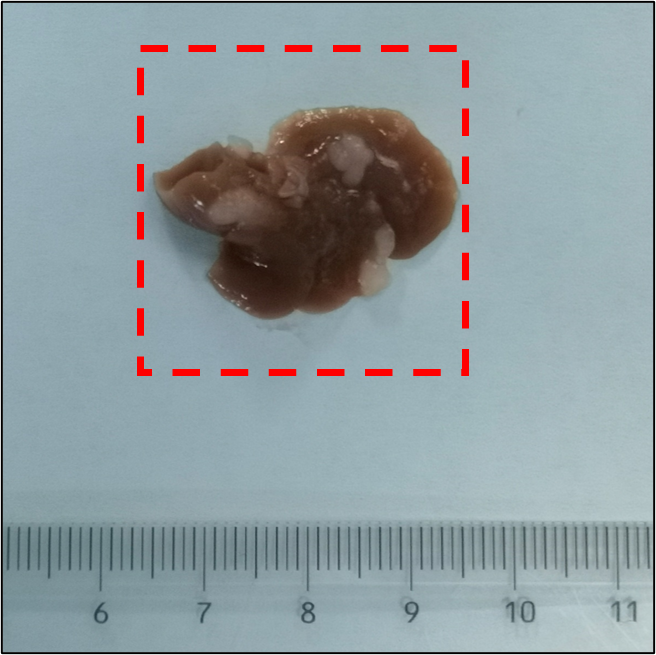

Supplement: Supplementary file 11 — Source data Fig. 3 [file 44318_2025_416_MOESM11_ESM.zip › EMBOJ-2024-119243R_SourceDataForFigure 3/3U/shNT-sgCtrl.tif]

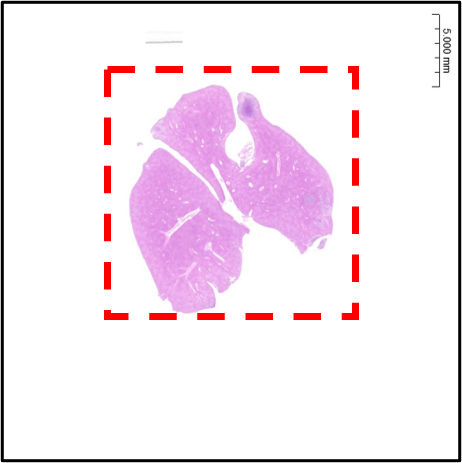

Supplement: Supplementary file 11 — Source data Fig. 3 [file 44318_2025_416_MOESM11_ESM.zip › EMBOJ-2024-119243R_SourceDataForFigure 3/3U/shNT-sgHIF-1α-HE.tif]

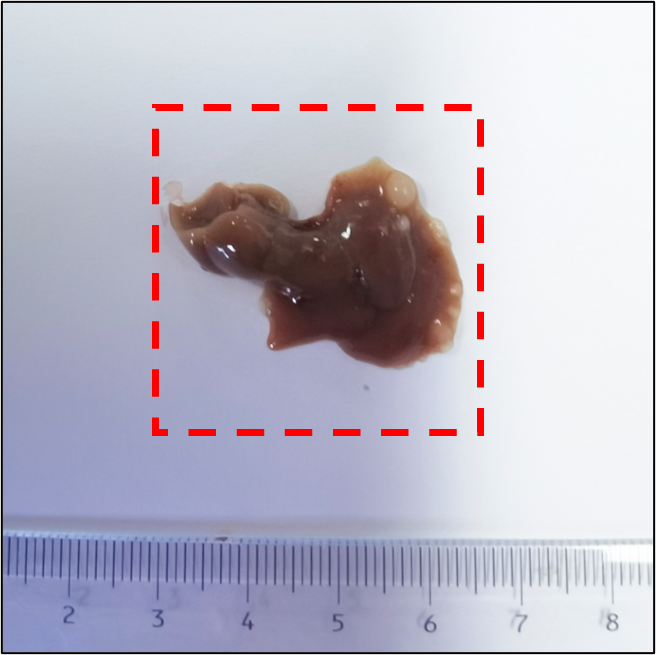

Supplement: Supplementary file 11 — Source data Fig. 3 [file 44318_2025_416_MOESM11_ESM.zip › EMBOJ-2024-119243R_SourceDataForFigure 3/3U/shNT-sgHIF-1α.tif]

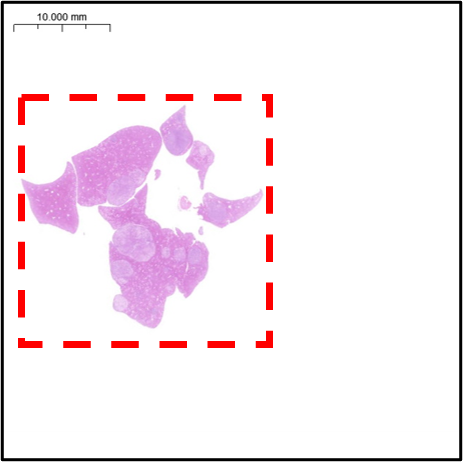

Supplement: Supplementary file 11 — Source data Fig. 3 [file 44318_2025_416_MOESM11_ESM.zip › EMBOJ-2024-119243R_SourceDataForFigure 3/3U/shPPA2-sgCtrl-HE.tif]

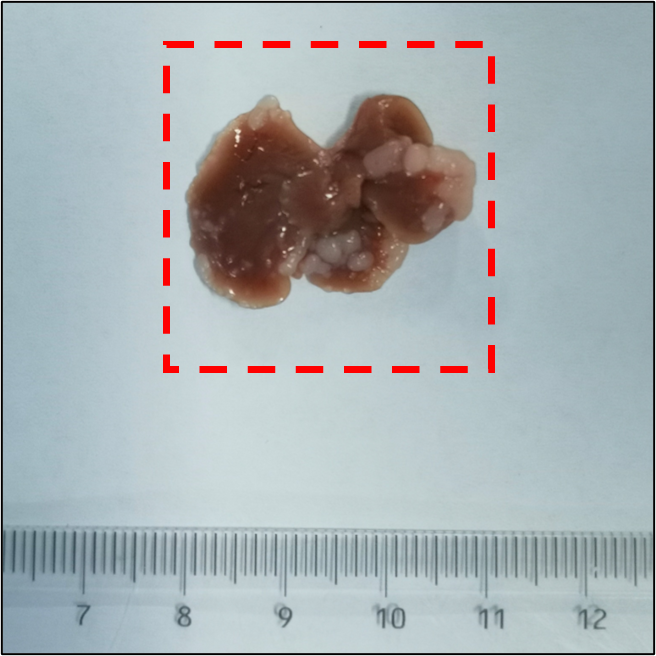

Supplement: Supplementary file 11 — Source data Fig. 3 [file 44318_2025_416_MOESM11_ESM.zip › EMBOJ-2024-119243R_SourceDataForFigure 3/3U/shPPA2-sgCtrl.tif]

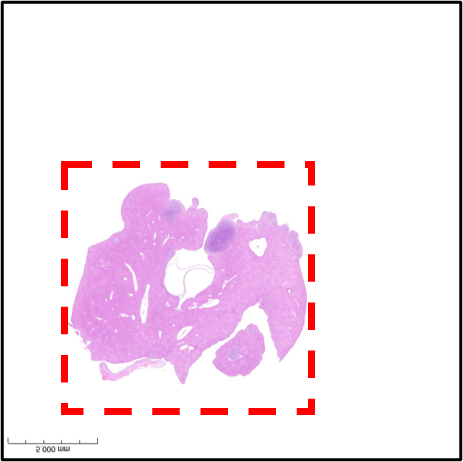

Supplement: Supplementary file 11 — Source data Fig. 3 [file 44318_2025_416_MOESM11_ESM.zip › EMBOJ-2024-119243R_SourceDataForFigure 3/3U/shPPA2-sgHIF-1α-HE.tif]

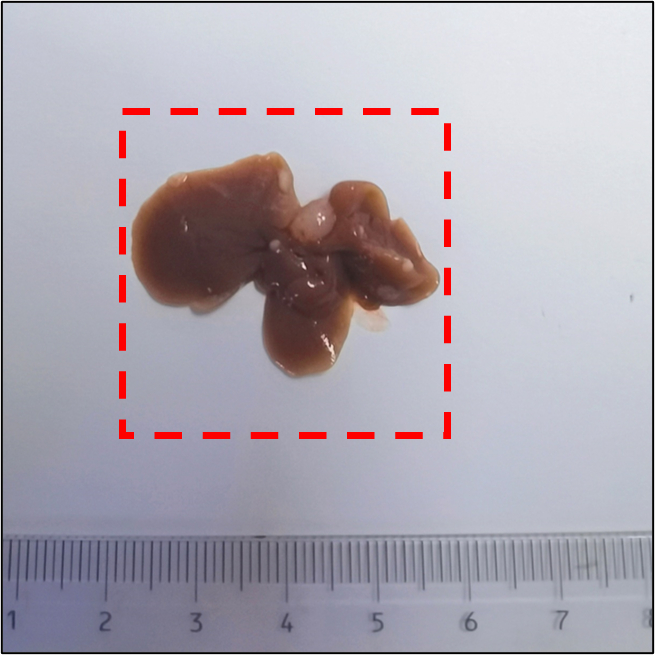

Supplement: Supplementary file 11 — Source data Fig. 3 [file 44318_2025_416_MOESM11_ESM.zip › EMBOJ-2024-119243R_SourceDataForFigure 3/3U/shPPA2-sgHIF-1α.tif]

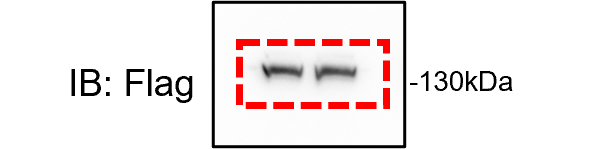

Supplement: Supplementary file 12 — Source data Fig. 4 [file 44318_2025_416_MOESM12_ESM.zip › EMBOJ-2024-119243R_SourceDataForFigure 4/4A/Input-Flag-LO.tif]

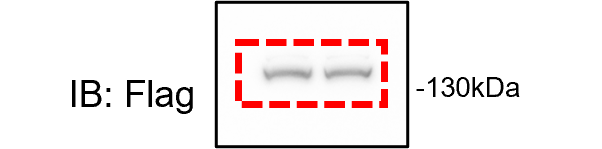

Supplement: Supplementary file 12 — Source data Fig. 4 [file 44318_2025_416_MOESM12_ESM.zip › EMBOJ-2024-119243R_SourceDataForFigure 4/4A/Input-Flag.tif]

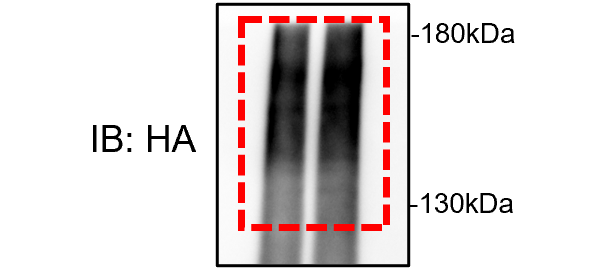

Supplement: Supplementary file 12 — Source data Fig. 4 [file 44318_2025_416_MOESM12_ESM.zip › EMBOJ-2024-119243R_SourceDataForFigure 4/4A/Input-HA-LO.tif]

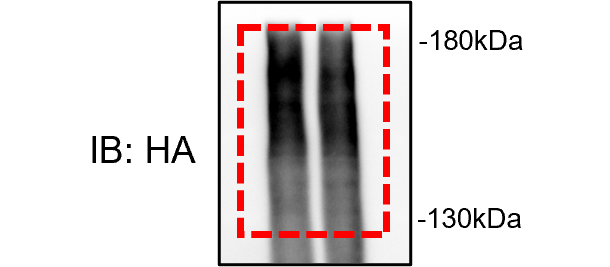

Supplement: Supplementary file 12 — Source data Fig. 4 [file 44318_2025_416_MOESM12_ESM.zip › EMBOJ-2024-119243R_SourceDataForFigure 4/4A/Input-HA.tif]

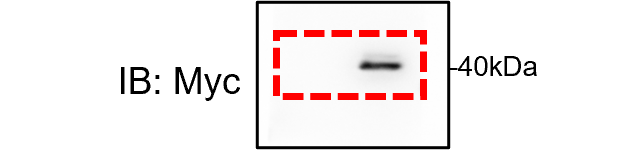

Supplement: Supplementary file 12 — Source data Fig. 4 [file 44318_2025_416_MOESM12_ESM.zip › EMBOJ-2024-119243R_SourceDataForFigure 4/4A/Input-Myc-LO.tif]

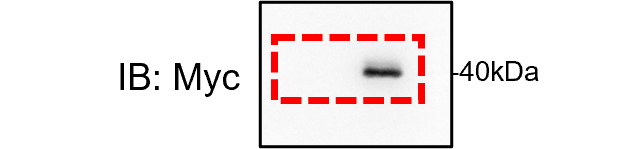

Supplement: Supplementary file 12 — Source data Fig. 4 [file 44318_2025_416_MOESM12_ESM.zip › EMBOJ-2024-119243R_SourceDataForFigure 4/4A/Input-Myc.tif]

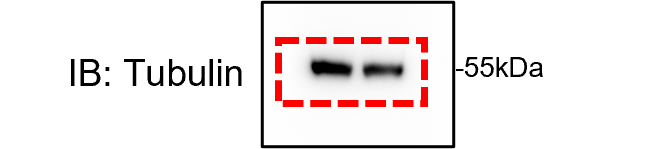

Supplement: Supplementary file 12 — Source data Fig. 4 [file 44318_2025_416_MOESM12_ESM.zip › EMBOJ-2024-119243R_SourceDataForFigure 4/4A/Input-Tubulin-LO.tif]

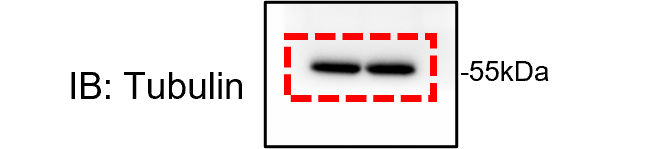

Supplement: Supplementary file 12 — Source data Fig. 4 [file 44318_2025_416_MOESM12_ESM.zip › EMBOJ-2024-119243R_SourceDataForFigure 4/4A/Input-Tubulin.tif]

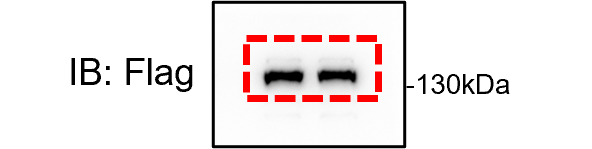

Supplement: Supplementary file 12 — Source data Fig. 4 [file 44318_2025_416_MOESM12_ESM.zip › EMBOJ-2024-119243R_SourceDataForFigure 4/4A/IP-Flag-LO.tif]

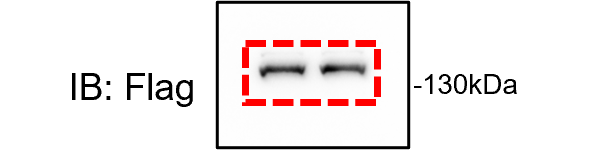

Supplement: Supplementary file 12 — Source data Fig. 4 [file 44318_2025_416_MOESM12_ESM.zip › EMBOJ-2024-119243R_SourceDataForFigure 4/4A/IP-Flag.tif]

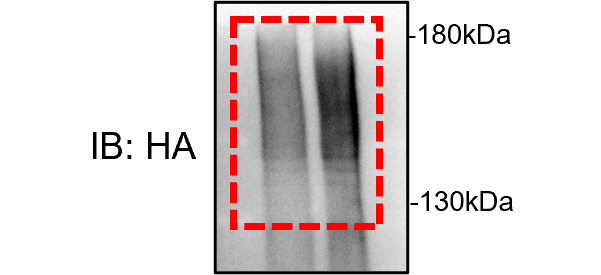

Supplement: Supplementary file 12 — Source data Fig. 4 [file 44318_2025_416_MOESM12_ESM.zip › EMBOJ-2024-119243R_SourceDataForFigure 4/4A/IP-HA-LO-Long exposuer.tif]

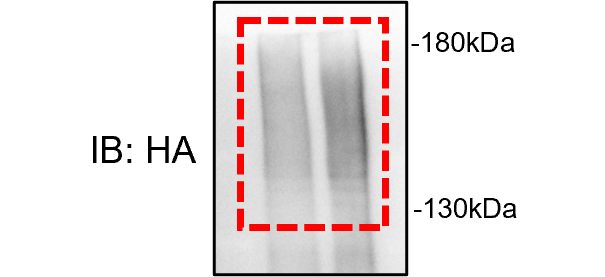

Supplement: Supplementary file 12 — Source data Fig. 4 [file 44318_2025_416_MOESM12_ESM.zip › EMBOJ-2024-119243R_SourceDataForFigure 4/4A/IP-HA-LO.tif]

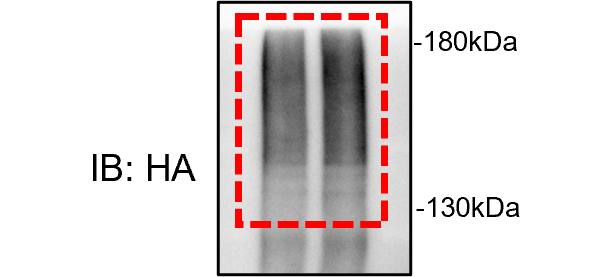

Supplement: Supplementary file 12 — Source data Fig. 4 [file 44318_2025_416_MOESM12_ESM.zip › EMBOJ-2024-119243R_SourceDataForFigure 4/4A/IP-HA.tif]

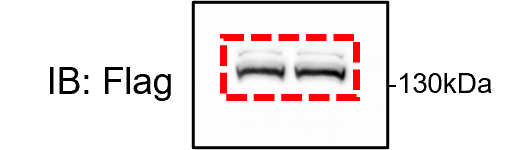

Supplement: Supplementary file 12 — Source data Fig. 4 [file 44318_2025_416_MOESM12_ESM.zip › EMBOJ-2024-119243R_SourceDataForFigure 4/4B/Input-Flag-LO.tif]

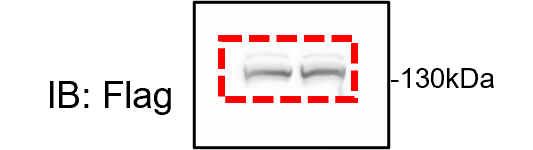

Supplement: Supplementary file 12 — Source data Fig. 4 [file 44318_2025_416_MOESM12_ESM.zip › EMBOJ-2024-119243R_SourceDataForFigure 4/4B/Input-Flag.tif]

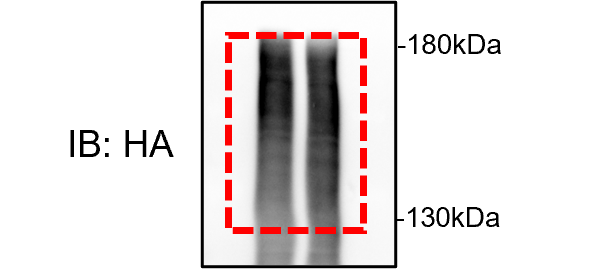

Supplement: Supplementary file 12 — Source data Fig. 4 [file 44318_2025_416_MOESM12_ESM.zip › EMBOJ-2024-119243R_SourceDataForFigure 4/4B/Input-HA-LO.tif]

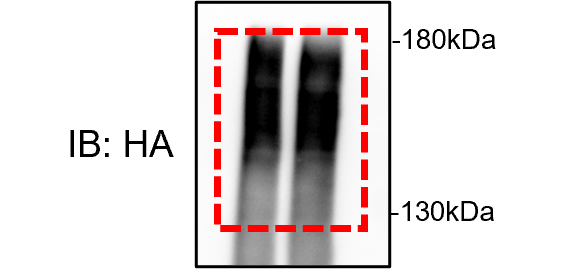

Supplement: Supplementary file 12 — Source data Fig. 4 [file 44318_2025_416_MOESM12_ESM.zip › EMBOJ-2024-119243R_SourceDataForFigure 4/4B/Input-HA.tif]

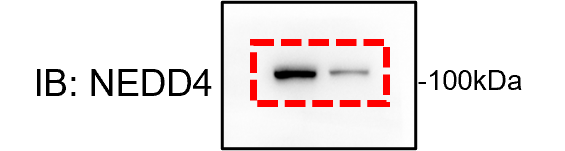

Supplement: Supplementary file 12 — Source data Fig. 4 [file 44318_2025_416_MOESM12_ESM.zip › EMBOJ-2024-119243R_SourceDataForFigure 4/4B/Input-NEDD4-LO.tif]

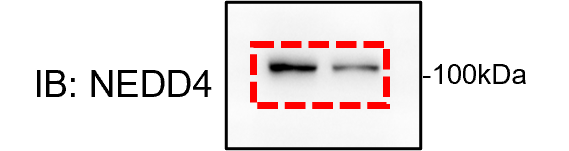

Supplement: Supplementary file 12 — Source data Fig. 4 [file 44318_2025_416_MOESM12_ESM.zip › EMBOJ-2024-119243R_SourceDataForFigure 4/4B/Input-NEDD4.tif]

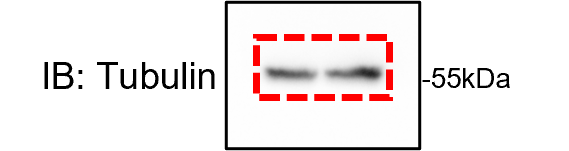

Supplement: Supplementary file 12 — Source data Fig. 4 [file 44318_2025_416_MOESM12_ESM.zip › EMBOJ-2024-119243R_SourceDataForFigure 4/4B/Input-Tubulin-LO.tif]

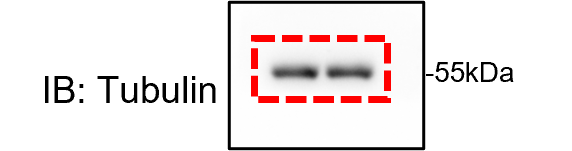

Supplement: Supplementary file 12 — Source data Fig. 4 [file 44318_2025_416_MOESM12_ESM.zip › EMBOJ-2024-119243R_SourceDataForFigure 4/4B/Input-Tubulin.tif]

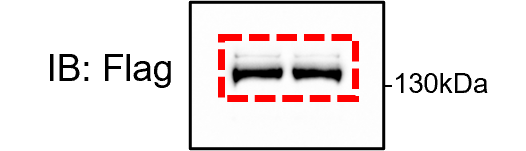

Supplement: Supplementary file 12 — Source data Fig. 4 [file 44318_2025_416_MOESM12_ESM.zip › EMBOJ-2024-119243R_SourceDataForFigure 4/4B/IP-Flag-LO.tif]

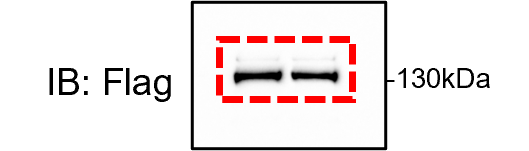

Supplement: Supplementary file 12 — Source data Fig. 4 [file 44318_2025_416_MOESM12_ESM.zip › EMBOJ-2024-119243R_SourceDataForFigure 4/4B/IP-Flag.tif]

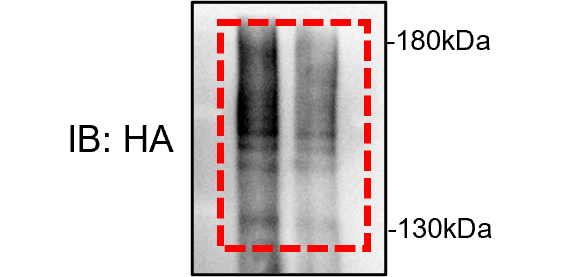

Supplement: Supplementary file 12 — Source data Fig. 4 [file 44318_2025_416_MOESM12_ESM.zip › EMBOJ-2024-119243R_SourceDataForFigure 4/4B/IP-HA-LO-Long exposure.tif]

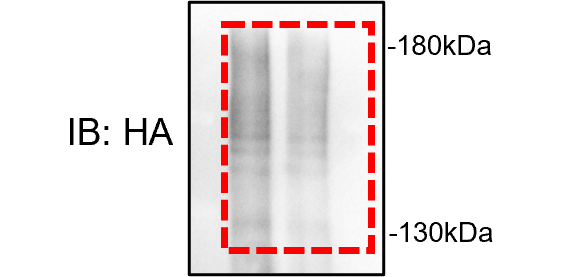

Supplement: Supplementary file 12 — Source data Fig. 4 [file 44318_2025_416_MOESM12_ESM.zip › EMBOJ-2024-119243R_SourceDataForFigure 4/4B/IP-HA-LO.tif]

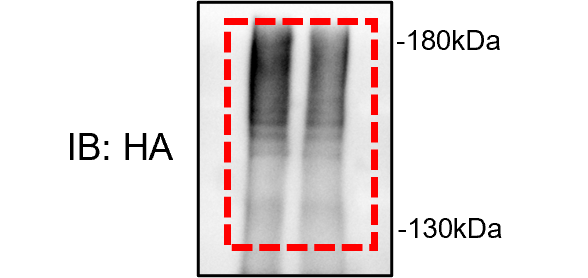

Supplement: Supplementary file 12 — Source data Fig. 4 [file 44318_2025_416_MOESM12_ESM.zip › EMBOJ-2024-119243R_SourceDataForFigure 4/4B/IP-HA.tif]

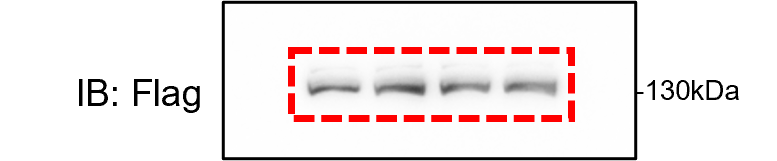

Supplement: Supplementary file 12 — Source data Fig. 4 [file 44318_2025_416_MOESM12_ESM.zip › EMBOJ-2024-119243R_SourceDataForFigure 4/4C/Input-Flag.tif]

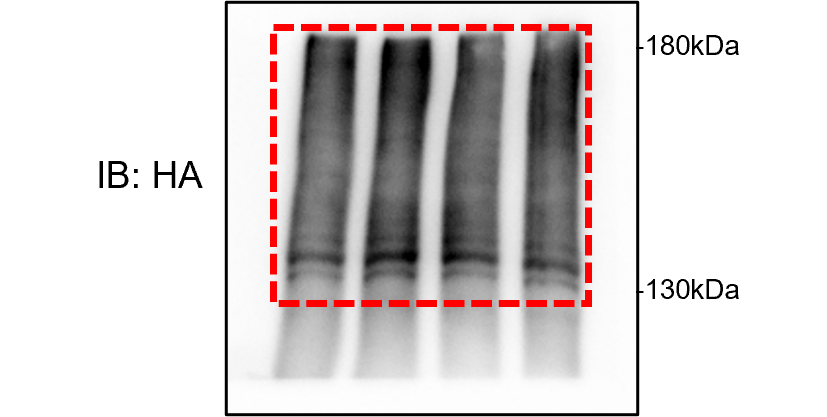

Supplement: Supplementary file 12 — Source data Fig. 4 [file 44318_2025_416_MOESM12_ESM.zip › EMBOJ-2024-119243R_SourceDataForFigure 4/4C/Input-HA.tif]

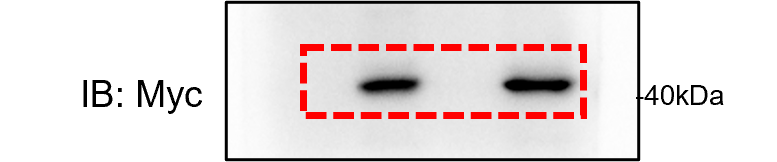

Supplement: Supplementary file 12 — Source data Fig. 4 [file 44318_2025_416_MOESM12_ESM.zip › EMBOJ-2024-119243R_SourceDataForFigure 4/4C/Input-Myc.tif]

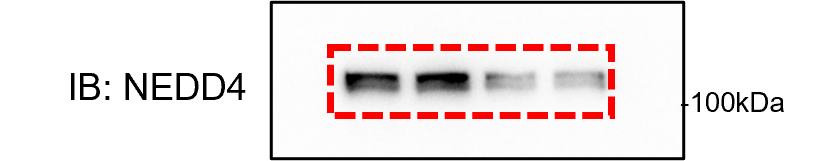

Supplement: Supplementary file 12 — Source data Fig. 4 [file 44318_2025_416_MOESM12_ESM.zip › EMBOJ-2024-119243R_SourceDataForFigure 4/4C/Input-NEDD4.tif]

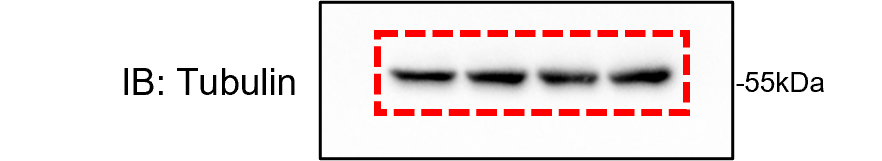

Supplement: Supplementary file 12 — Source data Fig. 4 [file 44318_2025_416_MOESM12_ESM.zip › EMBOJ-2024-119243R_SourceDataForFigure 4/4C/Input-Tubulin.tif]

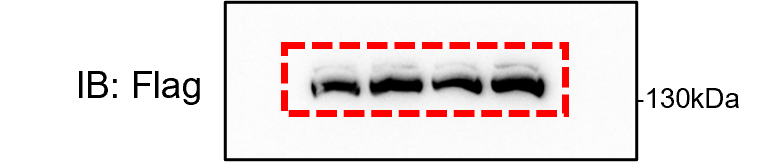

Supplement: Supplementary file 12 — Source data Fig. 4 [file 44318_2025_416_MOESM12_ESM.zip › EMBOJ-2024-119243R_SourceDataForFigure 4/4C/IP-Flag.tif]

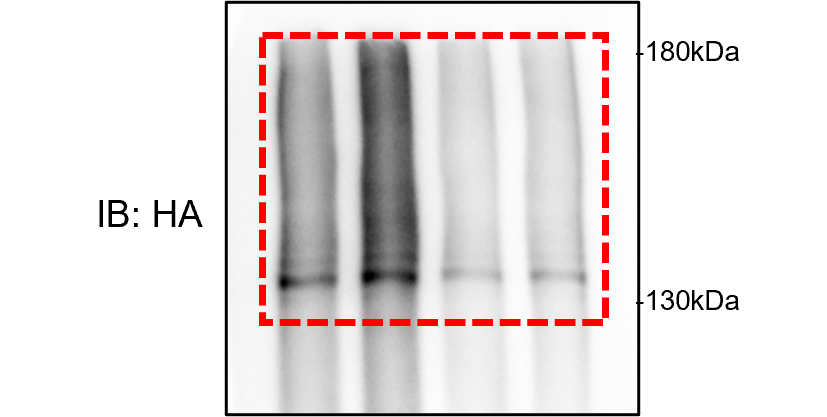

Supplement: Supplementary file 12 — Source data Fig. 4 [file 44318_2025_416_MOESM12_ESM.zip › EMBOJ-2024-119243R_SourceDataForFigure 4/4C/IP-HA.tif]

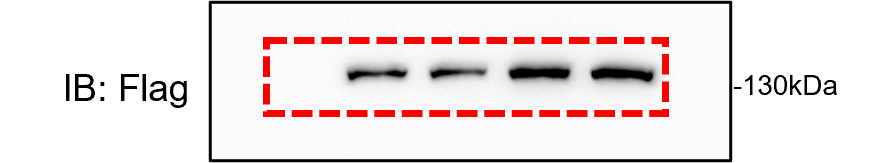

Supplement: Supplementary file 12 — Source data Fig. 4 [file 44318_2025_416_MOESM12_ESM.zip › EMBOJ-2024-119243R_SourceDataForFigure 4/4D/Input-Flag.tif]

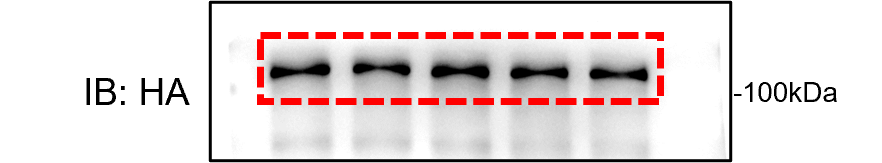

Supplement: Supplementary file 12 — Source data Fig. 4 [file 44318_2025_416_MOESM12_ESM.zip › EMBOJ-2024-119243R_SourceDataForFigure 4/4D/Input-HA.tif]

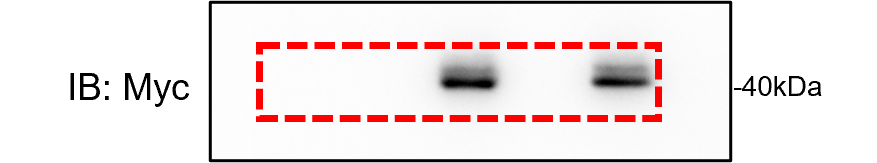

Supplement: Supplementary file 12 — Source data Fig. 4 [file 44318_2025_416_MOESM12_ESM.zip › EMBOJ-2024-119243R_SourceDataForFigure 4/4D/Input-Myc.tif]

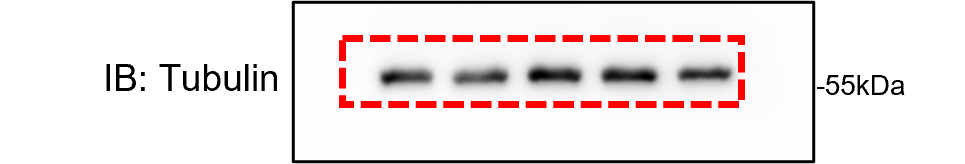

Supplement: Supplementary file 12 — Source data Fig. 4 [file 44318_2025_416_MOESM12_ESM.zip › EMBOJ-2024-119243R_SourceDataForFigure 4/4D/Input-Tubulin.tif]

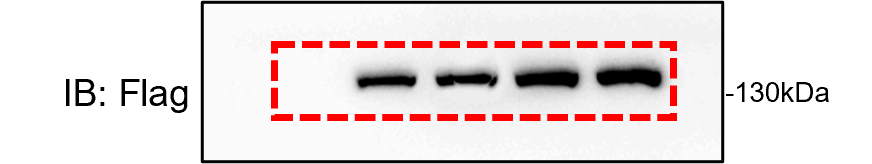

Supplement: Supplementary file 12 — Source data Fig. 4 [file 44318_2025_416_MOESM12_ESM.zip › EMBOJ-2024-119243R_SourceDataForFigure 4/4D/IP-Flag.tif]

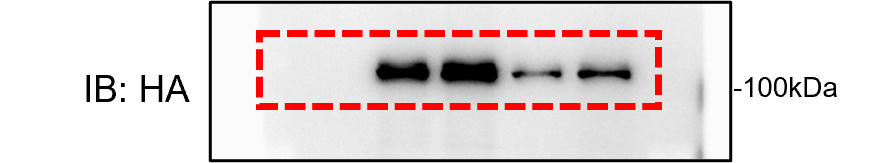

Supplement: Supplementary file 12 — Source data Fig. 4 [file 44318_2025_416_MOESM12_ESM.zip › EMBOJ-2024-119243R_SourceDataForFigure 4/4D/IP-HA-Long exposure.tif]

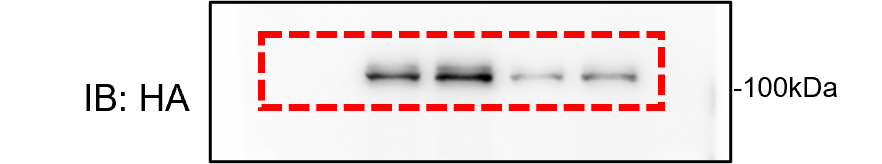

Supplement: Supplementary file 12 — Source data Fig. 4 [file 44318_2025_416_MOESM12_ESM.zip › EMBOJ-2024-119243R_SourceDataForFigure 4/4D/IP-HA-Short exposure.tif]

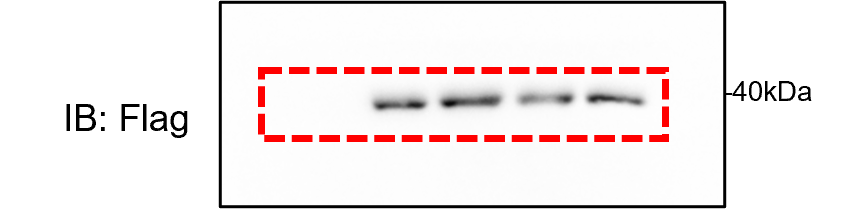

Supplement: Supplementary file 12 — Source data Fig. 4 [file 44318_2025_416_MOESM12_ESM.zip › EMBOJ-2024-119243R_SourceDataForFigure 4/4E/Input-Flag.tif]

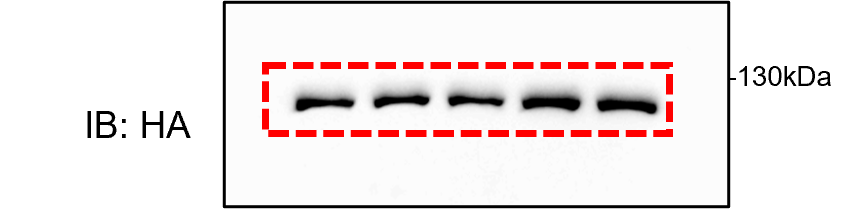

Supplement: Supplementary file 12 — Source data Fig. 4 [file 44318_2025_416_MOESM12_ESM.zip › EMBOJ-2024-119243R_SourceDataForFigure 4/4E/Input-HA.tif]

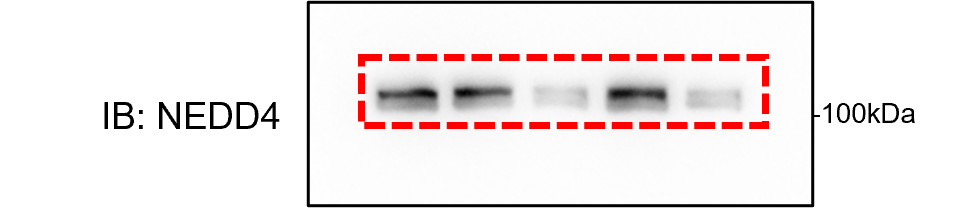

Supplement: Supplementary file 12 — Source data Fig. 4 [file 44318_2025_416_MOESM12_ESM.zip › EMBOJ-2024-119243R_SourceDataForFigure 4/4E/Input-NEDD4.tif]

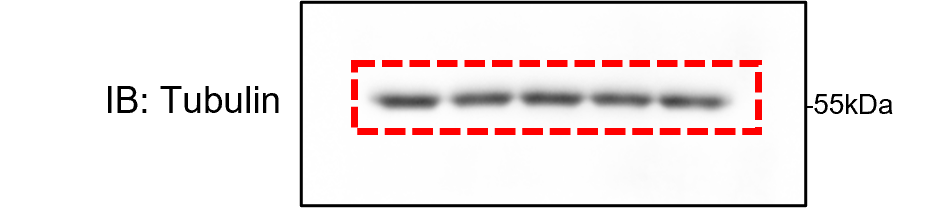

Supplement: Supplementary file 12 — Source data Fig. 4 [file 44318_2025_416_MOESM12_ESM.zip › EMBOJ-2024-119243R_SourceDataForFigure 4/4E/Input-Tubulin.tif]

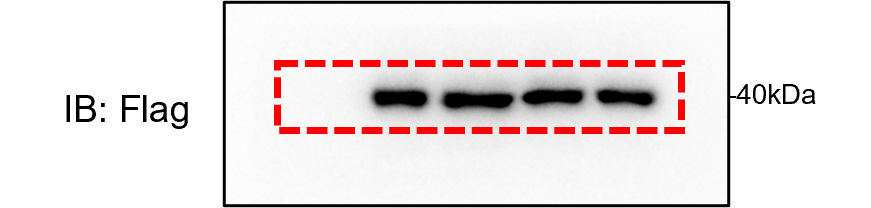

Supplement: Supplementary file 12 — Source data Fig. 4 [file 44318_2025_416_MOESM12_ESM.zip › EMBOJ-2024-119243R_SourceDataForFigure 4/4E/IP-Flag.tif]

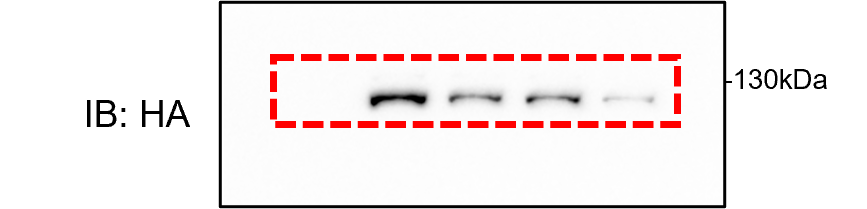

Supplement: Supplementary file 12 — Source data Fig. 4 [file 44318_2025_416_MOESM12_ESM.zip › EMBOJ-2024-119243R_SourceDataForFigure 4/4E/IP-HA.tif]

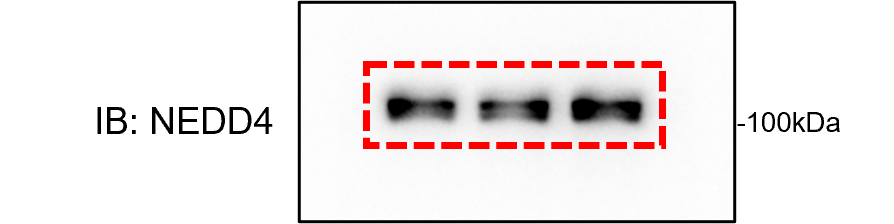

Supplement: Supplementary file 12 — Source data Fig. 4 [file 44318_2025_416_MOESM12_ESM.zip › EMBOJ-2024-119243R_SourceDataForFigure 4/4F/Input-NEDD4.tif]

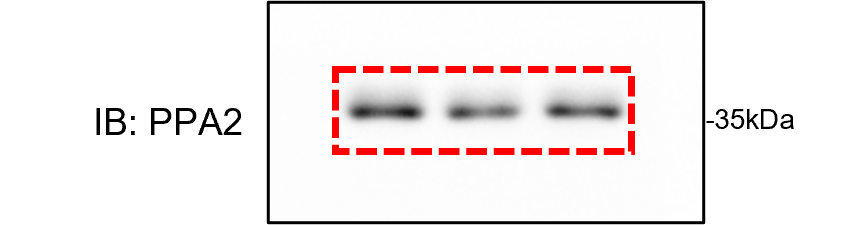

Supplement: Supplementary file 12 — Source data Fig. 4 [file 44318_2025_416_MOESM12_ESM.zip › EMBOJ-2024-119243R_SourceDataForFigure 4/4F/Input-PPA2.tif]

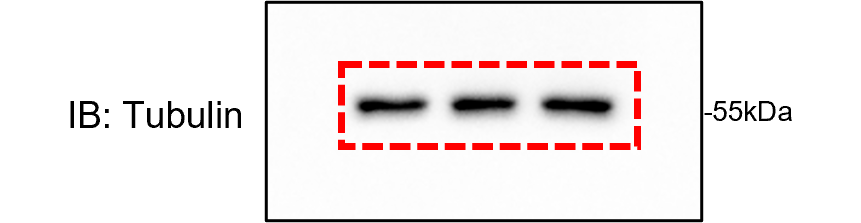

Supplement: Supplementary file 12 — Source data Fig. 4 [file 44318_2025_416_MOESM12_ESM.zip › EMBOJ-2024-119243R_SourceDataForFigure 4/4F/Input-Tubulin.tif]

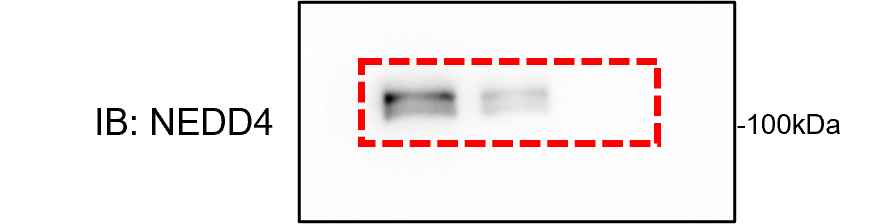

Supplement: Supplementary file 12 — Source data Fig. 4 [file 44318_2025_416_MOESM12_ESM.zip › EMBOJ-2024-119243R_SourceDataForFigure 4/4F/IP-NEDD4.tif]

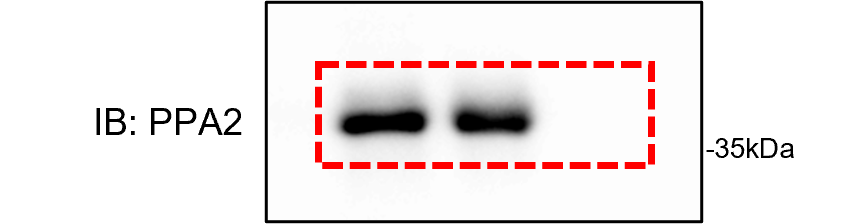

Supplement: Supplementary file 12 — Source data Fig. 4 [file 44318_2025_416_MOESM12_ESM.zip › EMBOJ-2024-119243R_SourceDataForFigure 4/4F/IP-PPA2.tif]

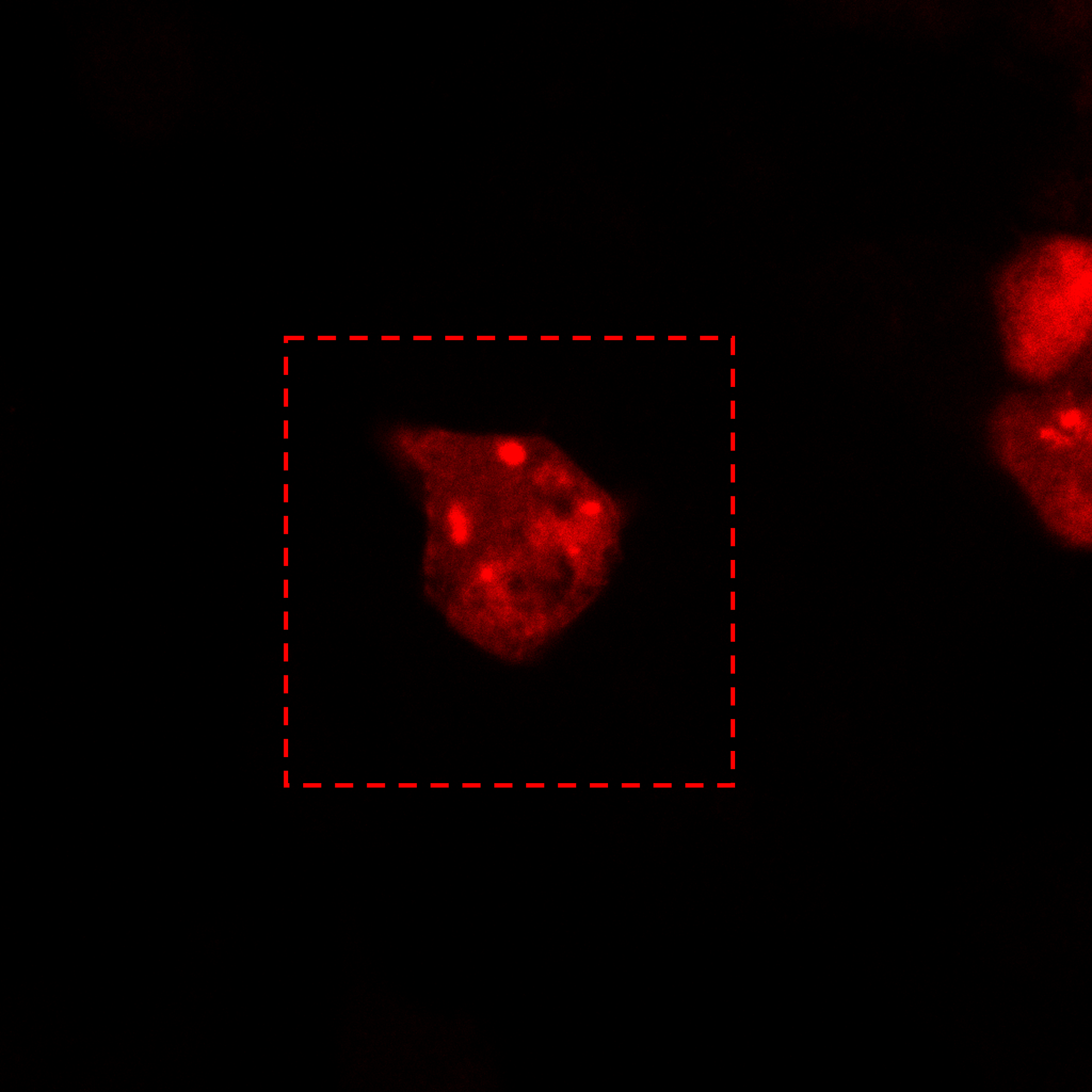

Supplement: Supplementary file 12 — Source data Fig. 4 [file 44318_2025_416_MOESM12_ESM.zip › EMBOJ-2024-119243R_SourceDataForFigure 4/4G/4G-HO-Flag-PPA2.tif]

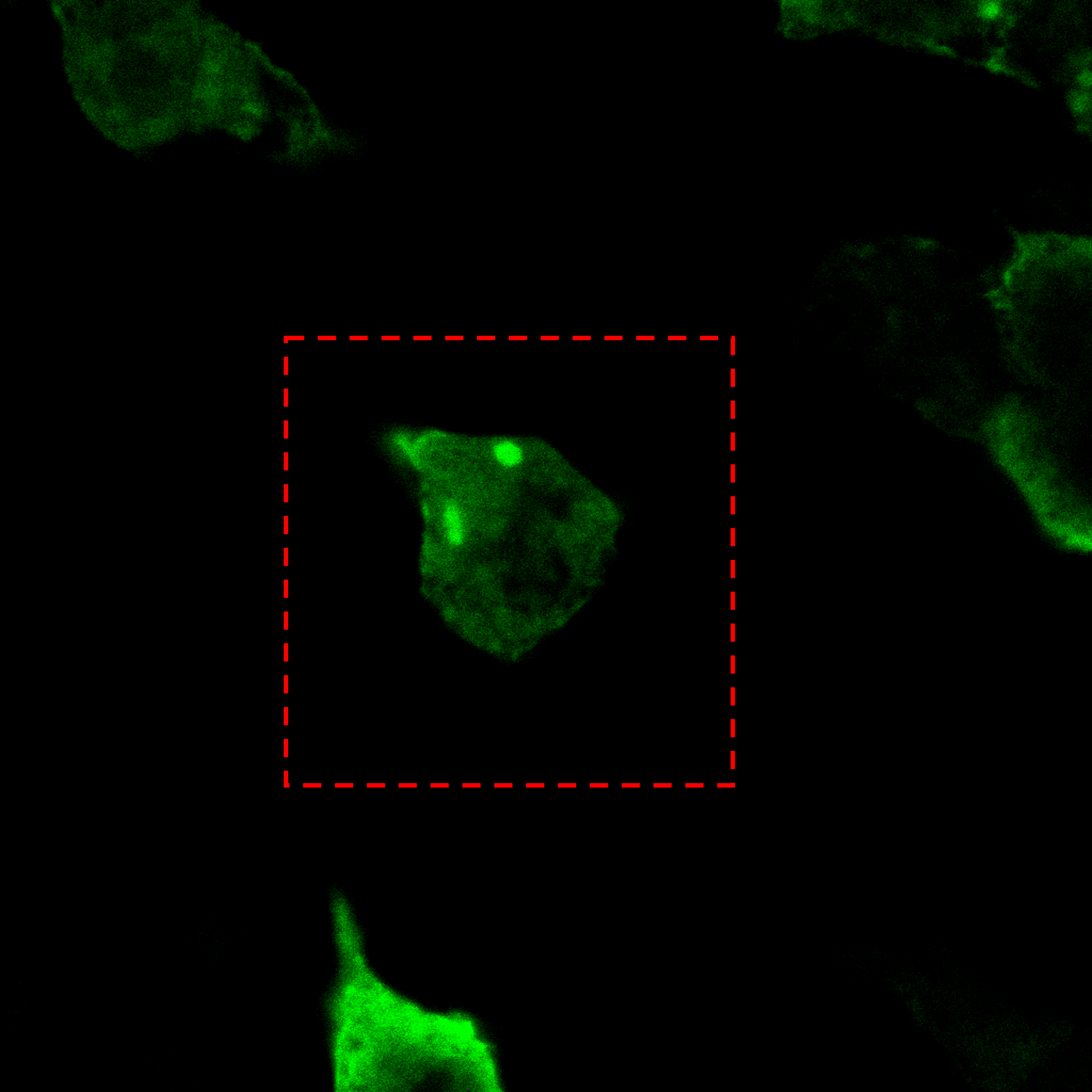

Supplement: Supplementary file 12 — Source data Fig. 4 [file 44318_2025_416_MOESM12_ESM.zip › EMBOJ-2024-119243R_SourceDataForFigure 4/4G/4G-HO-HA-NEDD4.tif]

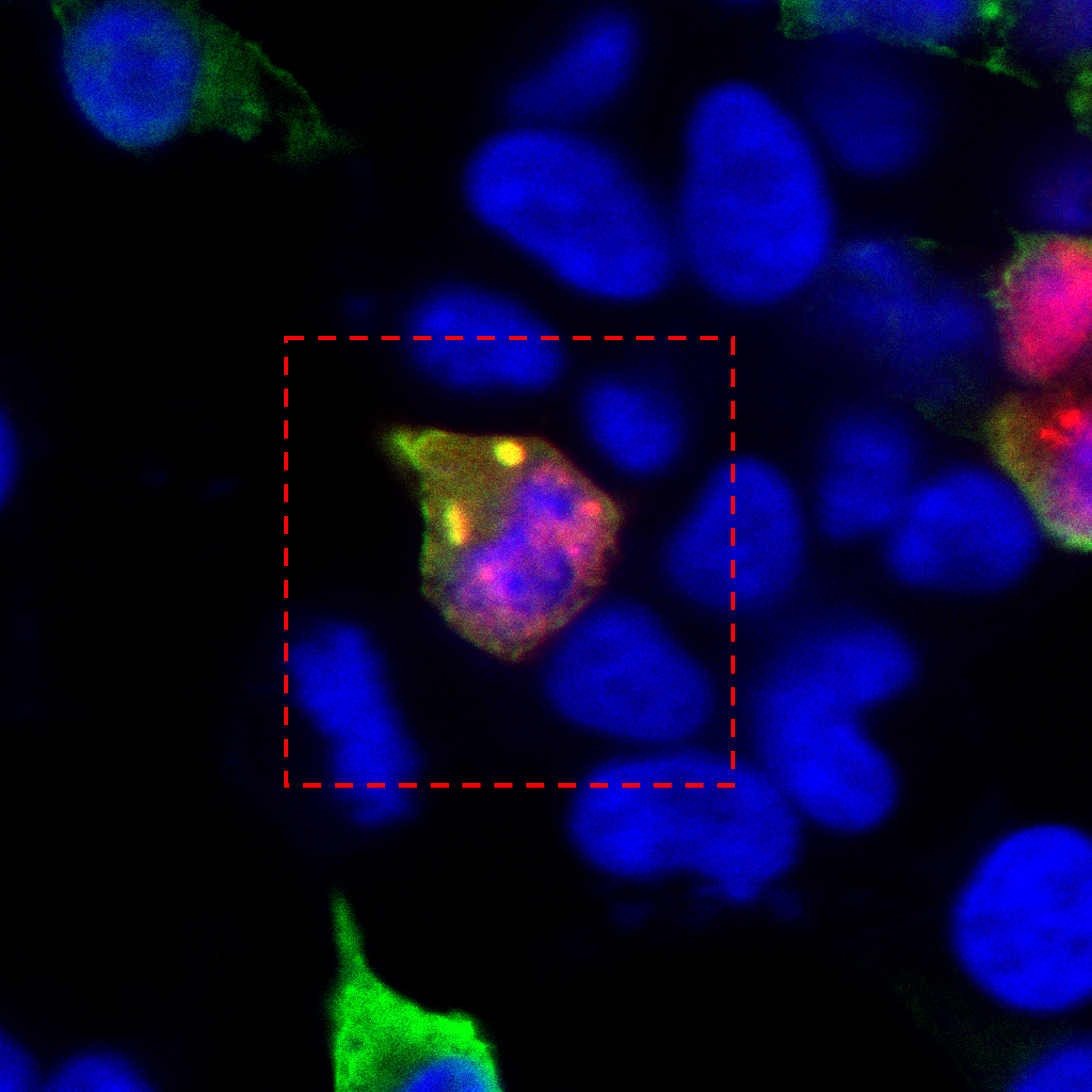

Supplement: Supplementary file 12 — Source data Fig. 4 [file 44318_2025_416_MOESM12_ESM.zip › EMBOJ-2024-119243R_SourceDataForFigure 4/4G/4G-HO-Merge.tif]

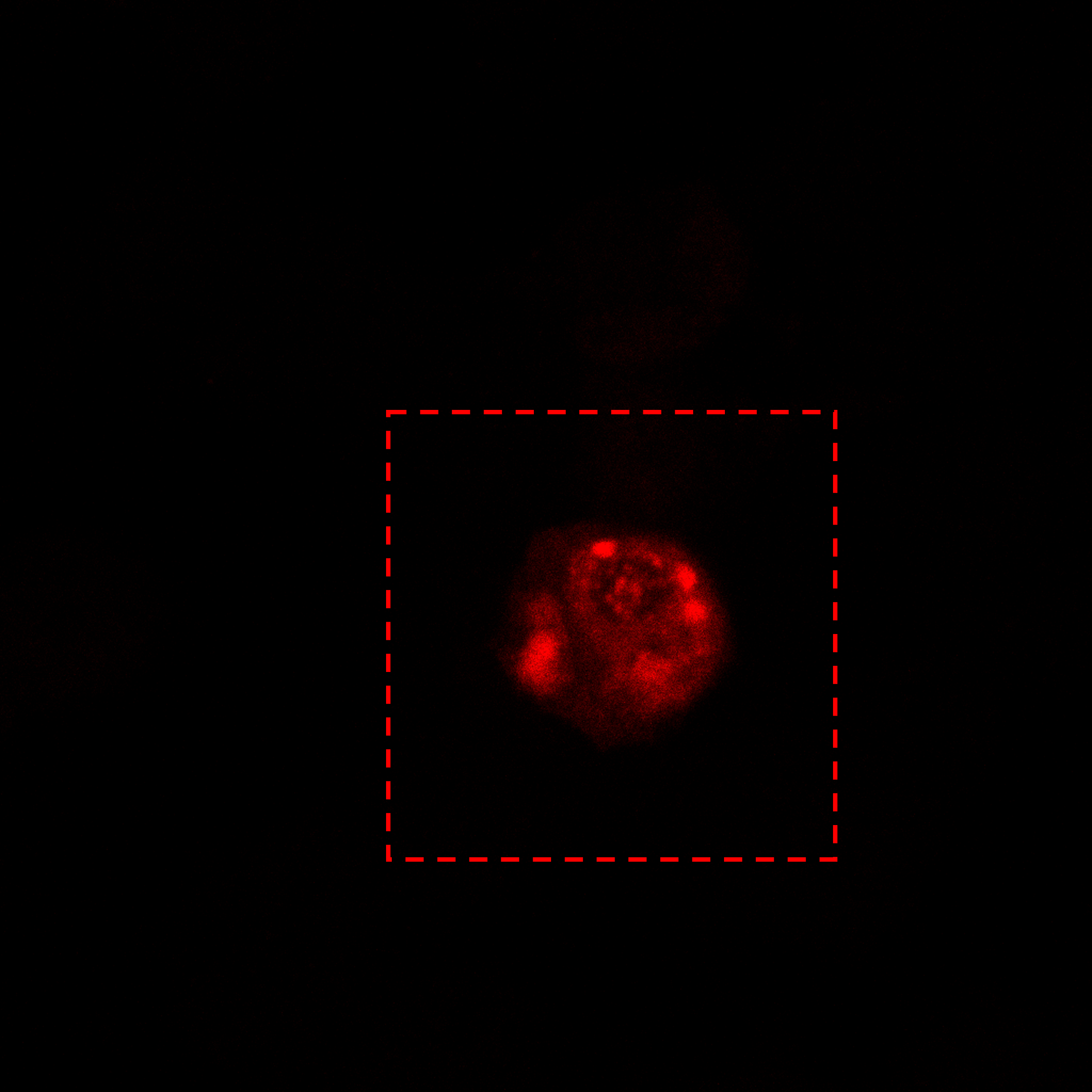

Supplement: Supplementary file 12 — Source data Fig. 4 [file 44318_2025_416_MOESM12_ESM.zip › EMBOJ-2024-119243R_SourceDataForFigure 4/4G/4G-LO-Flag-PPA2.tif]

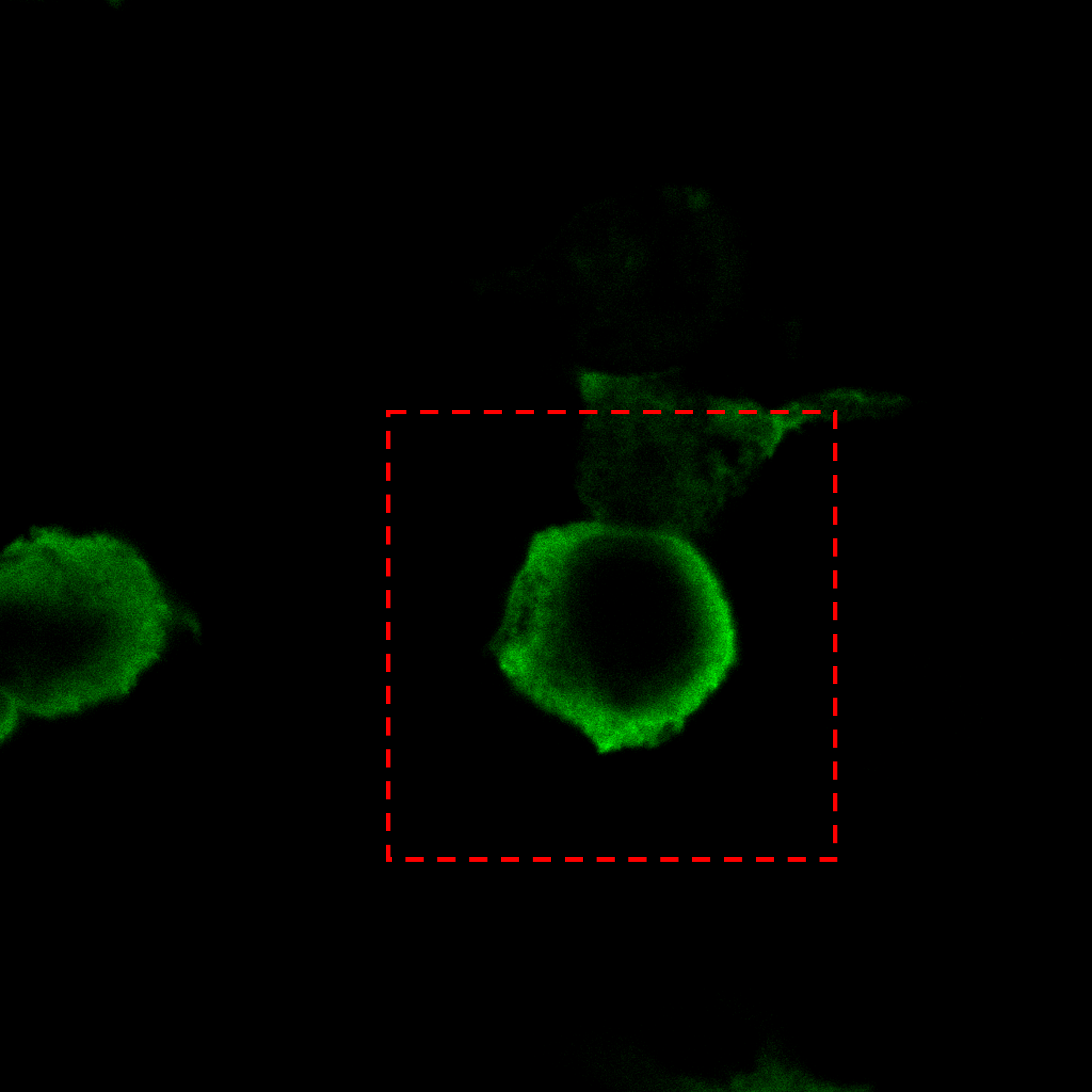

Supplement: Supplementary file 12 — Source data Fig. 4 [file 44318_2025_416_MOESM12_ESM.zip › EMBOJ-2024-119243R_SourceDataForFigure 4/4G/4G-LO-HA-NEDD4.tif]

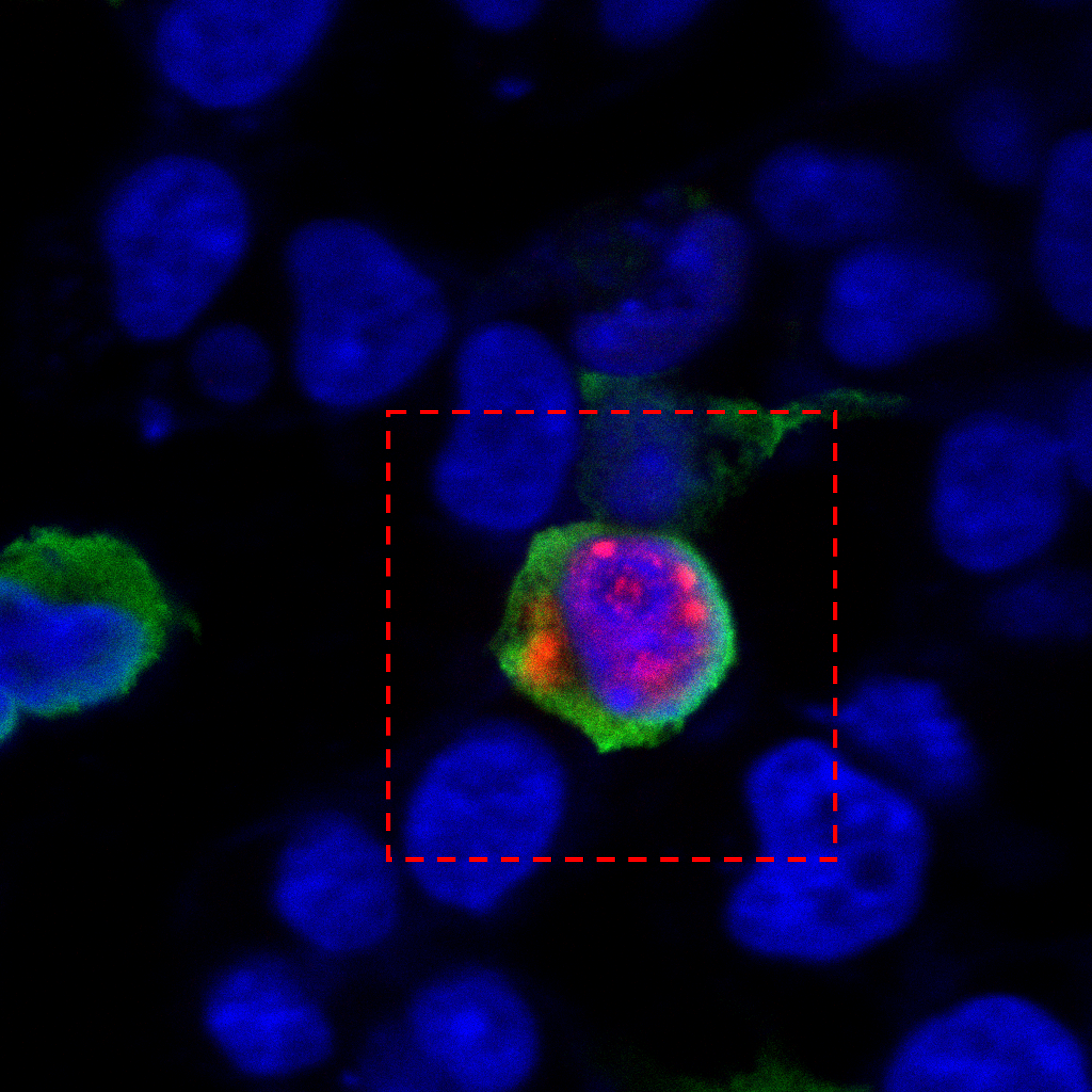

Supplement: Supplementary file 12 — Source data Fig. 4 [file 44318_2025_416_MOESM12_ESM.zip › EMBOJ-2024-119243R_SourceDataForFigure 4/4G/4G-LO-Merge.tif]

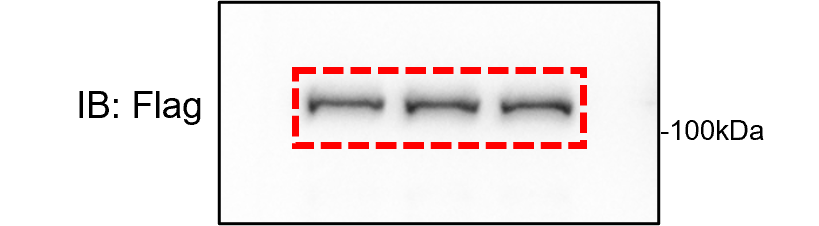

Supplement: Supplementary file 12 — Source data Fig. 4 [file 44318_2025_416_MOESM12_ESM.zip › EMBOJ-2024-119243R_SourceDataForFigure 4/4H/Input-Flag.tif]

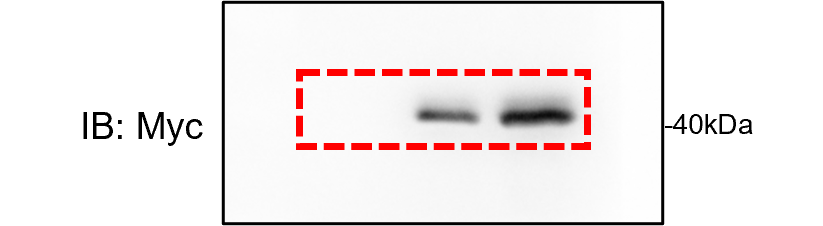

Supplement: Supplementary file 12 — Source data Fig. 4 [file 44318_2025_416_MOESM12_ESM.zip › EMBOJ-2024-119243R_SourceDataForFigure 4/4H/Input-Myc.tif]

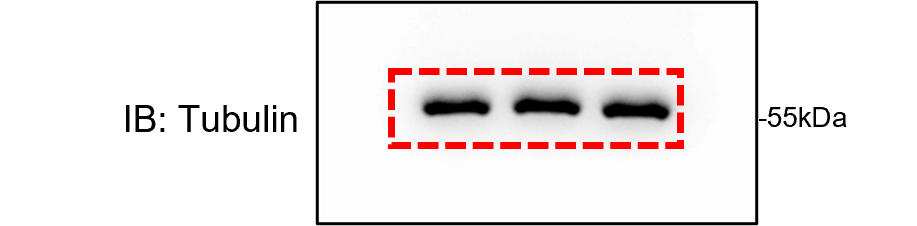

Supplement: Supplementary file 12 — Source data Fig. 4 [file 44318_2025_416_MOESM12_ESM.zip › EMBOJ-2024-119243R_SourceDataForFigure 4/4H/Input-Tubulin.tif]

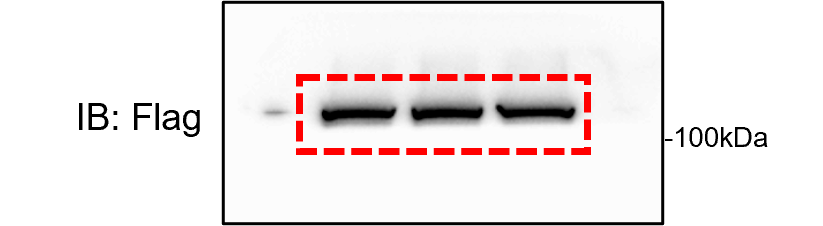

Supplement: Supplementary file 12 — Source data Fig. 4 [file 44318_2025_416_MOESM12_ESM.zip › EMBOJ-2024-119243R_SourceDataForFigure 4/4H/IP-Flag.tif]

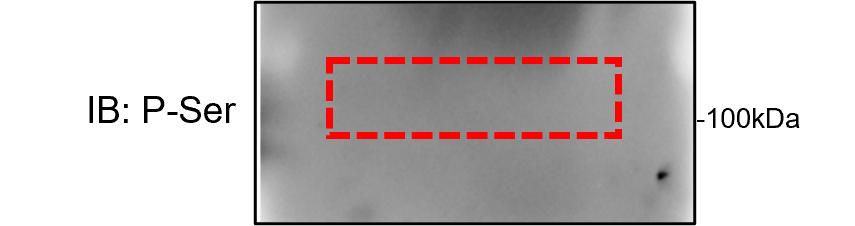

Supplement: Supplementary file 12 — Source data Fig. 4 [file 44318_2025_416_MOESM12_ESM.zip › EMBOJ-2024-119243R_SourceDataForFigure 4/4H/IP-P-Ser.tif]

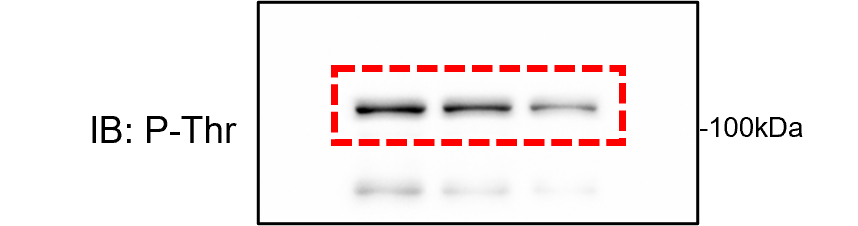

Supplement: Supplementary file 12 — Source data Fig. 4 [file 44318_2025_416_MOESM12_ESM.zip › EMBOJ-2024-119243R_SourceDataForFigure 4/4H/IP-P-Thr.tif]

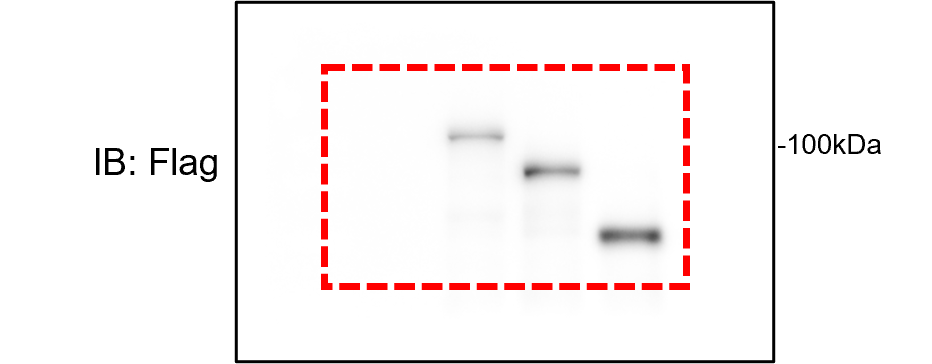

Supplement: Supplementary file 12 — Source data Fig. 4 [file 44318_2025_416_MOESM12_ESM.zip › EMBOJ-2024-119243R_SourceDataForFigure 4/4J/Input-Flag.tif]

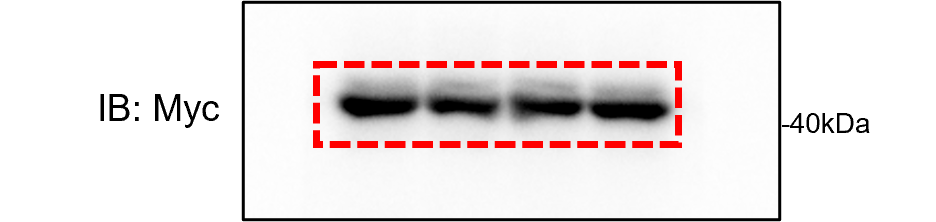

Supplement: Supplementary file 12 — Source data Fig. 4 [file 44318_2025_416_MOESM12_ESM.zip › EMBOJ-2024-119243R_SourceDataForFigure 4/4J/Input-Myc.tif]

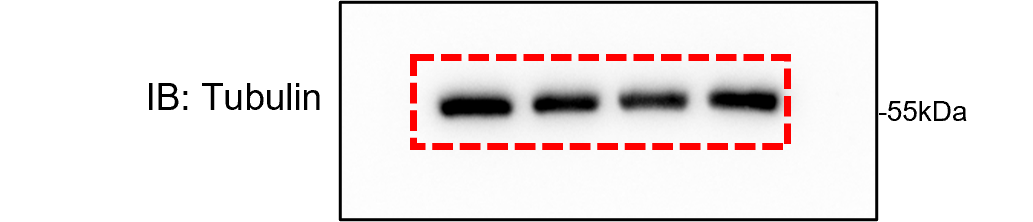

Supplement: Supplementary file 12 — Source data Fig. 4 [file 44318_2025_416_MOESM12_ESM.zip › EMBOJ-2024-119243R_SourceDataForFigure 4/4J/Input-Tubulin.tif]

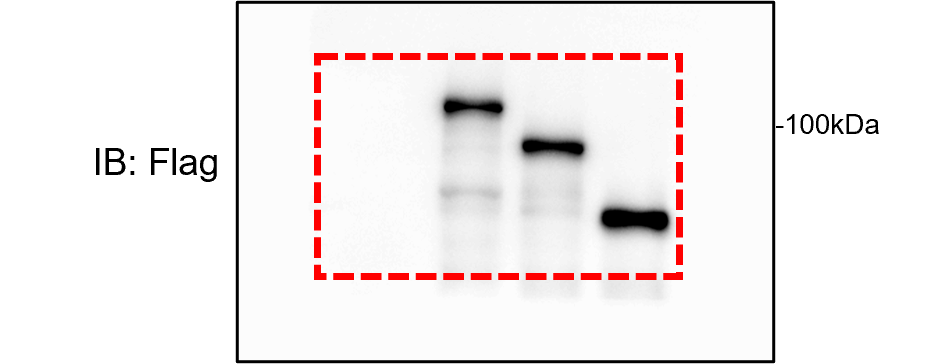

Supplement: Supplementary file 12 — Source data Fig. 4 [file 44318_2025_416_MOESM12_ESM.zip › EMBOJ-2024-119243R_SourceDataForFigure 4/4J/IP-Flag.tif]

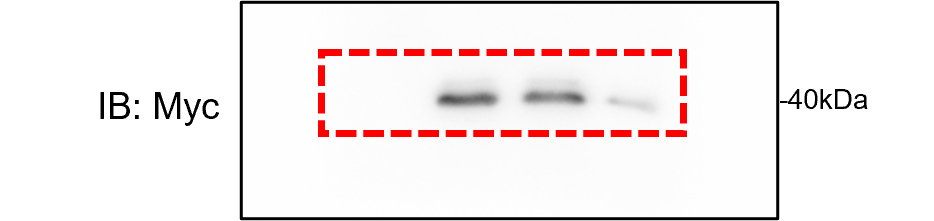

Supplement: Supplementary file 12 — Source data Fig. 4 [file 44318_2025_416_MOESM12_ESM.zip › EMBOJ-2024-119243R_SourceDataForFigure 4/4J/IP-Myc.tif]

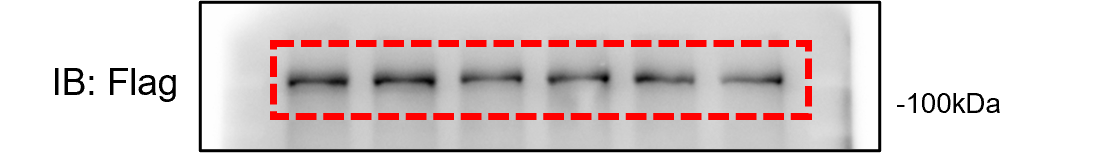

Supplement: Supplementary file 12 — Source data Fig. 4 [file 44318_2025_416_MOESM12_ESM.zip › EMBOJ-2024-119243R_SourceDataForFigure 4/4K/Input-Flag.tif]

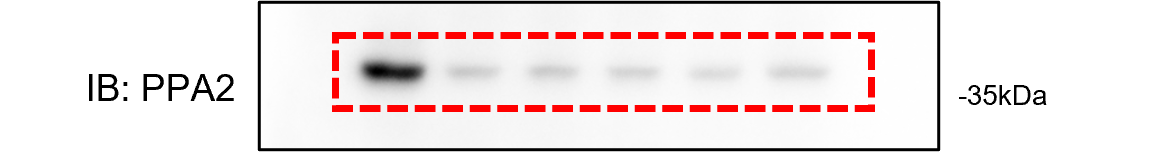

Supplement: Supplementary file 12 — Source data Fig. 4 [file 44318_2025_416_MOESM12_ESM.zip › EMBOJ-2024-119243R_SourceDataForFigure 4/4K/Input-PPA2.tif]

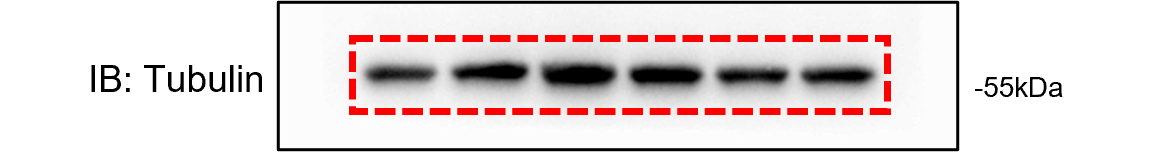

Supplement: Supplementary file 12 — Source data Fig. 4 [file 44318_2025_416_MOESM12_ESM.zip › EMBOJ-2024-119243R_SourceDataForFigure 4/4K/Input-Tubulin.tif]

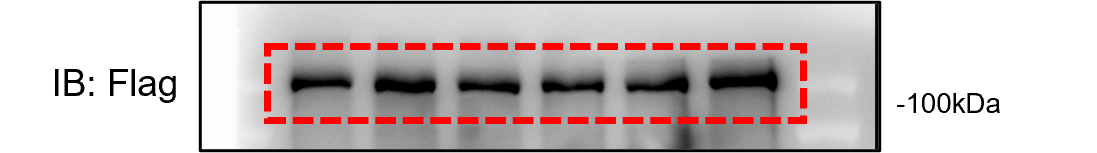

Supplement: Supplementary file 12 — Source data Fig. 4 [file 44318_2025_416_MOESM12_ESM.zip › EMBOJ-2024-119243R_SourceDataForFigure 4/4K/IP-Flag.tif]

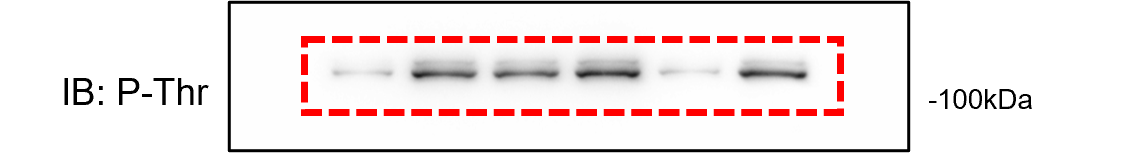

Supplement: Supplementary file 12 — Source data Fig. 4 [file 44318_2025_416_MOESM12_ESM.zip › EMBOJ-2024-119243R_SourceDataForFigure 4/4K/IP-P-Thr.tif]

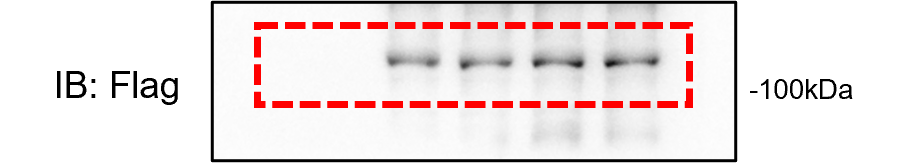

Supplement: Supplementary file 12 — Source data Fig. 4 [file 44318_2025_416_MOESM12_ESM.zip › EMBOJ-2024-119243R_SourceDataForFigure 4/4M/Input-Flag.tif]

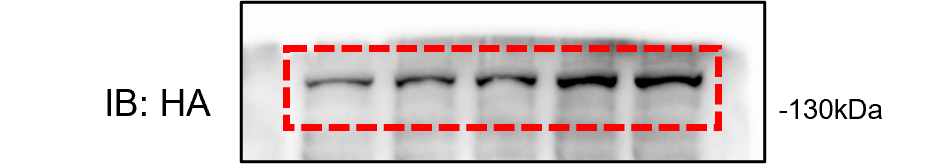

Supplement: Supplementary file 12 — Source data Fig. 4 [file 44318_2025_416_MOESM12_ESM.zip › EMBOJ-2024-119243R_SourceDataForFigure 4/4M/Input-HA.tif]

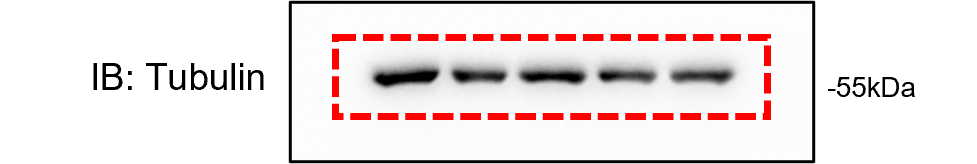

Supplement: Supplementary file 12 — Source data Fig. 4 [file 44318_2025_416_MOESM12_ESM.zip › EMBOJ-2024-119243R_SourceDataForFigure 4/4M/Input-Tubulin.tif]

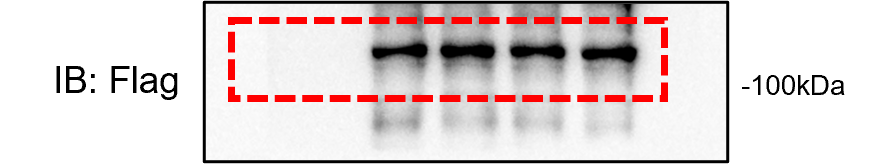

Supplement: Supplementary file 12 — Source data Fig. 4 [file 44318_2025_416_MOESM12_ESM.zip › EMBOJ-2024-119243R_SourceDataForFigure 4/4M/IP-Flag.tif]

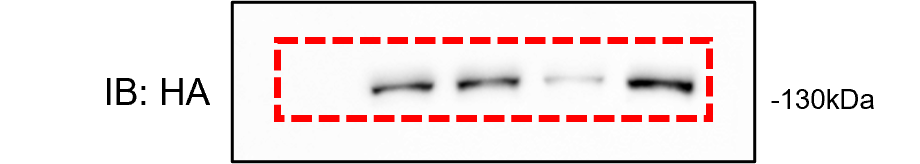

Supplement: Supplementary file 12 — Source data Fig. 4 [file 44318_2025_416_MOESM12_ESM.zip › EMBOJ-2024-119243R_SourceDataForFigure 4/4M/IP-HA.tif]

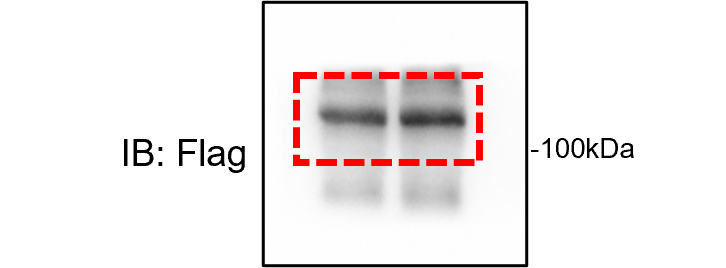

Supplement: Supplementary file 12 — Source data Fig. 4 [file 44318_2025_416_MOESM12_ESM.zip › EMBOJ-2024-119243R_SourceDataForFigure 4/4N/Input-Flag-LO.tif]

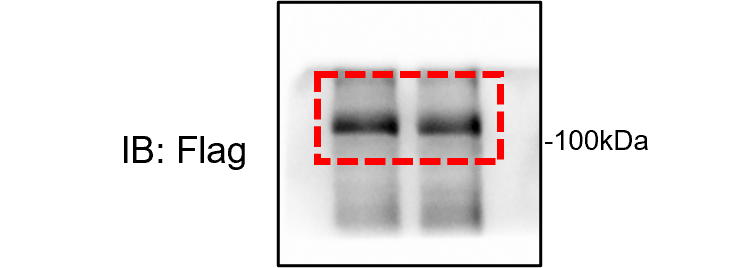

Supplement: Supplementary file 12 — Source data Fig. 4 [file 44318_2025_416_MOESM12_ESM.zip › EMBOJ-2024-119243R_SourceDataForFigure 4/4N/Input-Flag.tif]

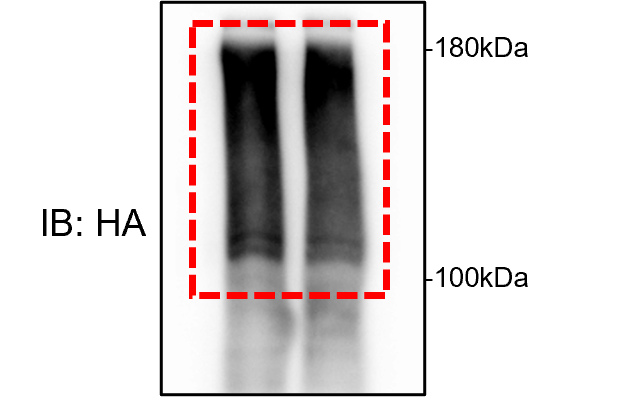

Supplement: Supplementary file 12 — Source data Fig. 4 [file 44318_2025_416_MOESM12_ESM.zip › EMBOJ-2024-119243R_SourceDataForFigure 4/4N/Input-HA-LO.tif]

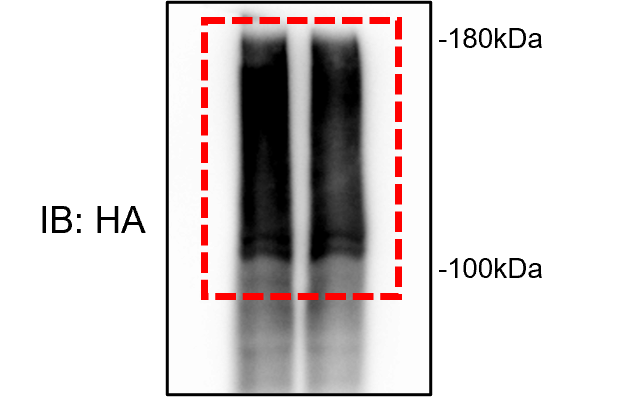

Supplement: Supplementary file 12 — Source data Fig. 4 [file 44318_2025_416_MOESM12_ESM.zip › EMBOJ-2024-119243R_SourceDataForFigure 4/4N/Input-HA.tif]

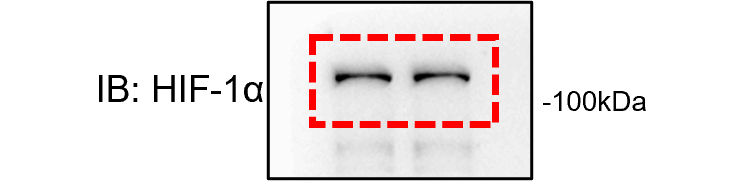

Supplement: Supplementary file 12 — Source data Fig. 4 [file 44318_2025_416_MOESM12_ESM.zip › EMBOJ-2024-119243R_SourceDataForFigure 4/4N/Input-HIF-1α-LO.tif]

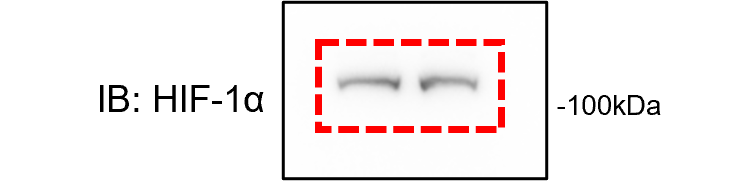

Supplement: Supplementary file 12 — Source data Fig. 4 [file 44318_2025_416_MOESM12_ESM.zip › EMBOJ-2024-119243R_SourceDataForFigure 4/4N/Input-HIF-1α.tif]

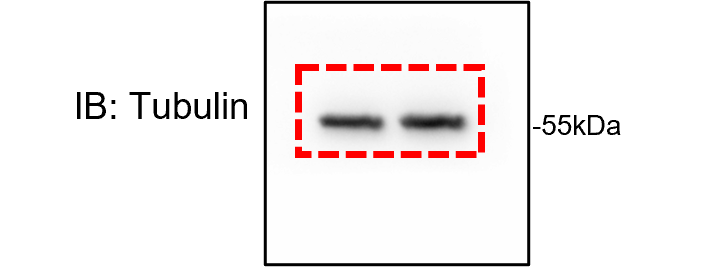

Supplement: Supplementary file 12 — Source data Fig. 4 [file 44318_2025_416_MOESM12_ESM.zip › EMBOJ-2024-119243R_SourceDataForFigure 4/4N/Input-Tubulin-LO.tif]

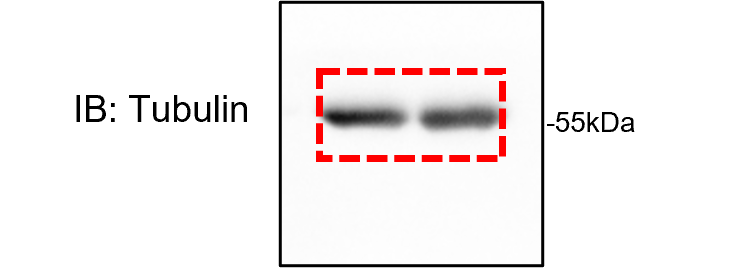

Supplement: Supplementary file 12 — Source data Fig. 4 [file 44318_2025_416_MOESM12_ESM.zip › EMBOJ-2024-119243R_SourceDataForFigure 4/4N/Input-Tubulin.tif]
