# Supplementary figures and images for: SIRT5-mediated desuccinylation of PPA2 enhances HIF-1alpha-dependent adaptation to hypoxic stress and colorectal cancer metastasis (part 3 of 5)
Source: EMBO J. 2025 Mar 31;44(9):2514–40. doi: 10.1038/s44318-025-00416-1 (PMC12048626; doi:10.1038/s44318-025-00416-1)

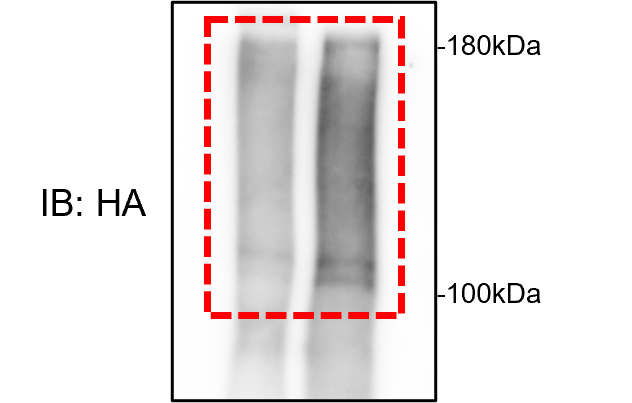

Supplement: Supplementary file 12 — Source data Fig. 4 [file 44318_2025_416_MOESM12_ESM.zip › EMBOJ-2024-119243R_SourceDataForFigure 4/4N/IP-HA-LO.tif]

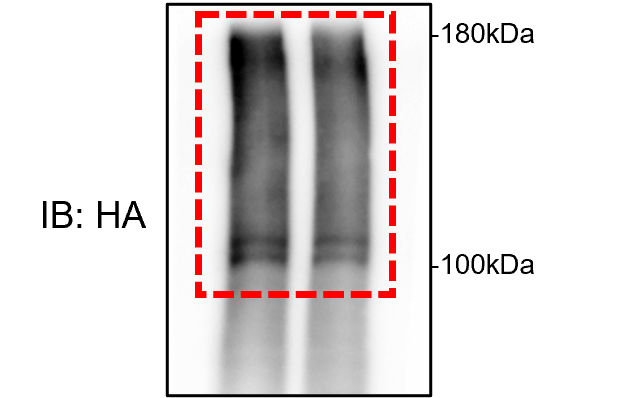

Supplement: Supplementary file 12 — Source data Fig. 4 [file 44318_2025_416_MOESM12_ESM.zip › EMBOJ-2024-119243R_SourceDataForFigure 4/4N/IP-HA.tif]

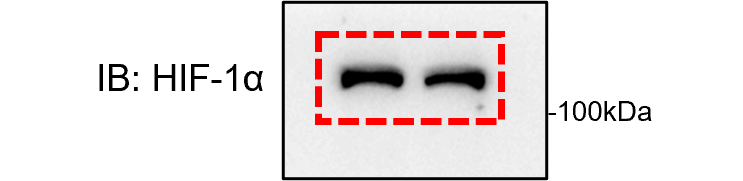

Supplement: Supplementary file 12 — Source data Fig. 4 [file 44318_2025_416_MOESM12_ESM.zip › EMBOJ-2024-119243R_SourceDataForFigure 4/4N/IP-HIF-1α-LO.tif]

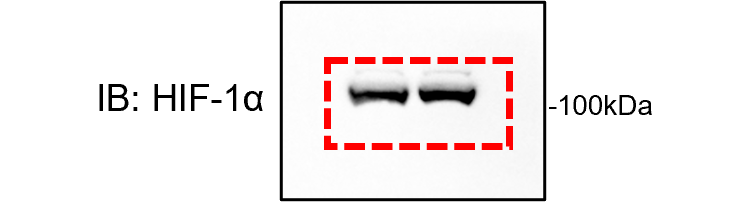

Supplement: Supplementary file 12 — Source data Fig. 4 [file 44318_2025_416_MOESM12_ESM.zip › EMBOJ-2024-119243R_SourceDataForFigure 4/4N/IP-HIF-1α.tif]

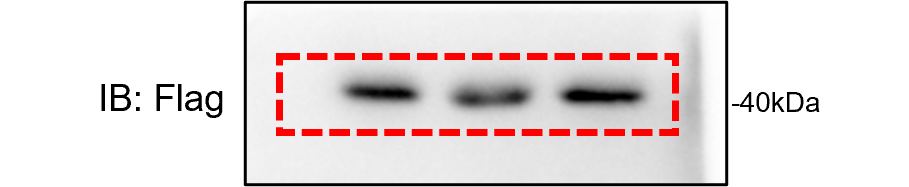

Supplement: Supplementary file 13 — Source data Fig. 5 [file 44318_2025_416_MOESM13_ESM.zip › EMBOJ-2024-119243R_SourceDataForFigure 5/5A/Input-Flag.tif]

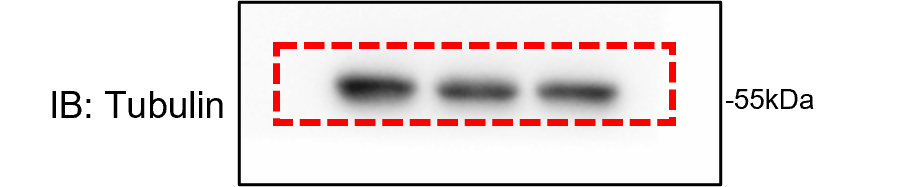

Supplement: Supplementary file 13 — Source data Fig. 5 [file 44318_2025_416_MOESM13_ESM.zip › EMBOJ-2024-119243R_SourceDataForFigure 5/5A/Input-Tubulin.tif]

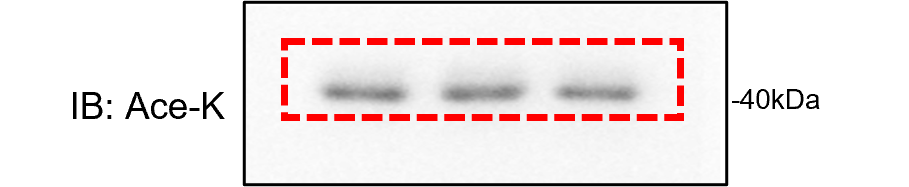

Supplement: Supplementary file 13 — Source data Fig. 5 [file 44318_2025_416_MOESM13_ESM.zip › EMBOJ-2024-119243R_SourceDataForFigure 5/5A/IP-Ace-K.tif]

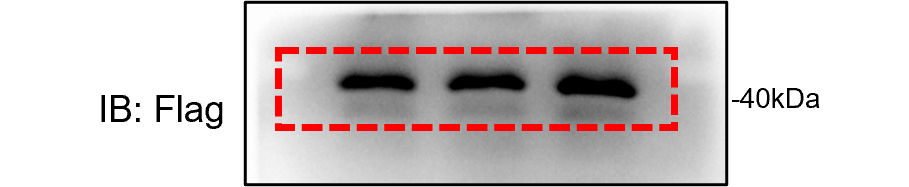

Supplement: Supplementary file 13 — Source data Fig. 5 [file 44318_2025_416_MOESM13_ESM.zip › EMBOJ-2024-119243R_SourceDataForFigure 5/5A/IP-Flag.tif]

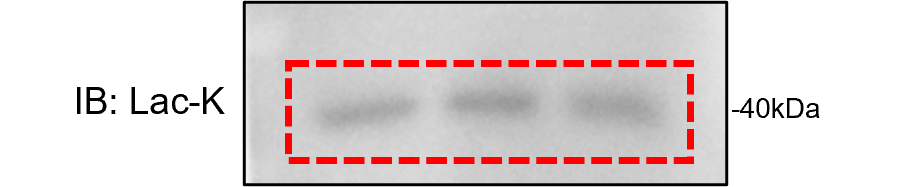

Supplement: Supplementary file 13 — Source data Fig. 5 [file 44318_2025_416_MOESM13_ESM.zip › EMBOJ-2024-119243R_SourceDataForFigure 5/5A/IP-Lac-K.tif]

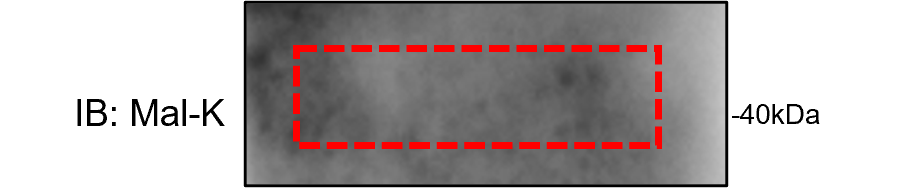

Supplement: Supplementary file 13 — Source data Fig. 5 [file 44318_2025_416_MOESM13_ESM.zip › EMBOJ-2024-119243R_SourceDataForFigure 5/5A/IP-Mal-K.tif]

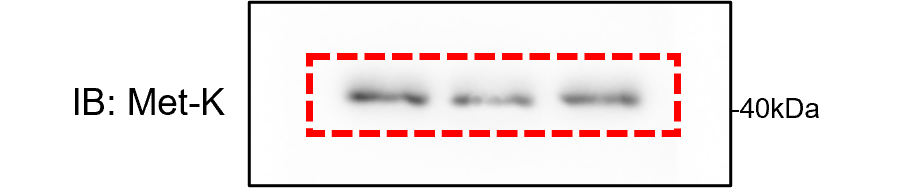

Supplement: Supplementary file 13 — Source data Fig. 5 [file 44318_2025_416_MOESM13_ESM.zip › EMBOJ-2024-119243R_SourceDataForFigure 5/5A/IP-Met-K.tif]

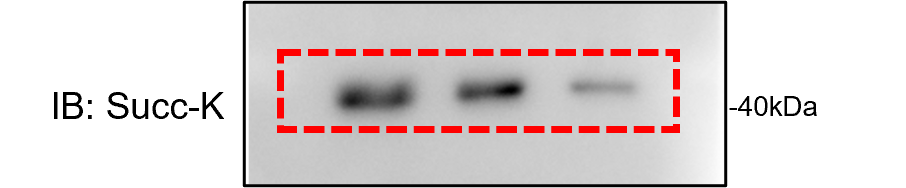

Supplement: Supplementary file 13 — Source data Fig. 5 [file 44318_2025_416_MOESM13_ESM.zip › EMBOJ-2024-119243R_SourceDataForFigure 5/5A/IP-Succ-K.tif]

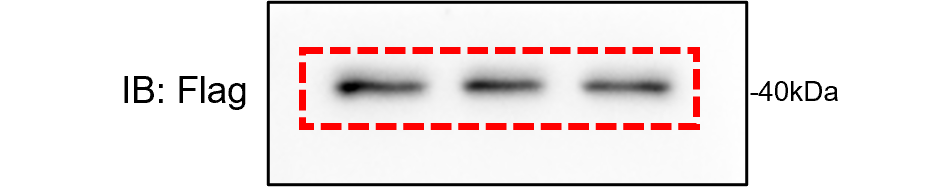

Supplement: Supplementary file 13 — Source data Fig. 5 [file 44318_2025_416_MOESM13_ESM.zip › EMBOJ-2024-119243R_SourceDataForFigure 5/5B/Input-Flag.tif]

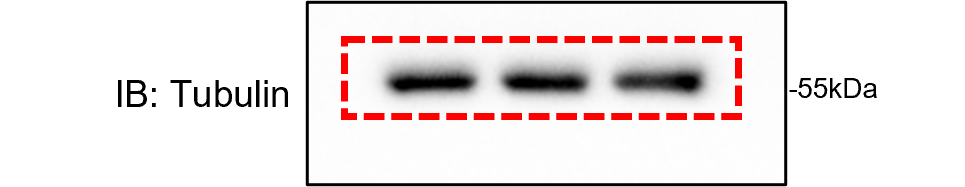

Supplement: Supplementary file 13 — Source data Fig. 5 [file 44318_2025_416_MOESM13_ESM.zip › EMBOJ-2024-119243R_SourceDataForFigure 5/5B/Input-Tubulin.tif]

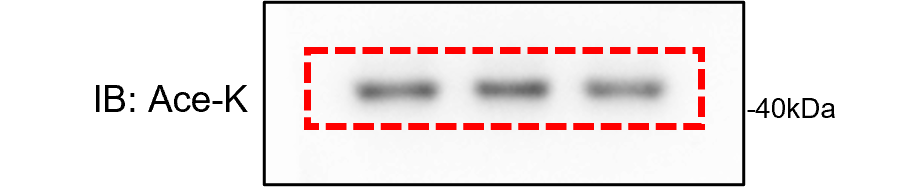

Supplement: Supplementary file 13 — Source data Fig. 5 [file 44318_2025_416_MOESM13_ESM.zip › EMBOJ-2024-119243R_SourceDataForFigure 5/5B/IP-Ace-K.tif]

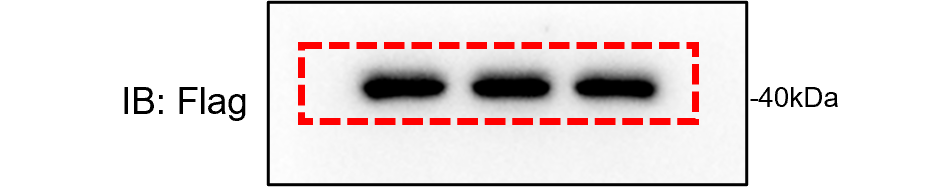

Supplement: Supplementary file 13 — Source data Fig. 5 [file 44318_2025_416_MOESM13_ESM.zip › EMBOJ-2024-119243R_SourceDataForFigure 5/5B/IP-Flag.tif]

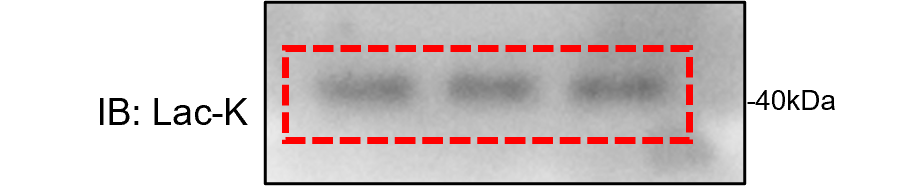

Supplement: Supplementary file 13 — Source data Fig. 5 [file 44318_2025_416_MOESM13_ESM.zip › EMBOJ-2024-119243R_SourceDataForFigure 5/5B/IP-Lac-K.tif]

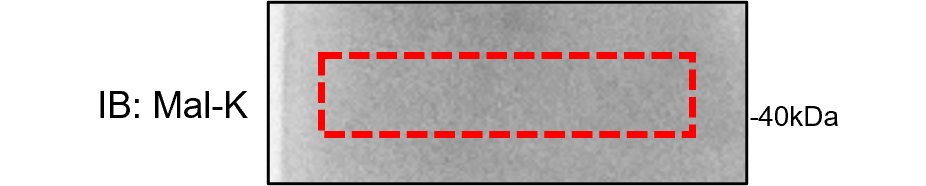

Supplement: Supplementary file 13 — Source data Fig. 5 [file 44318_2025_416_MOESM13_ESM.zip › EMBOJ-2024-119243R_SourceDataForFigure 5/5B/IP-Mal-K.tif]

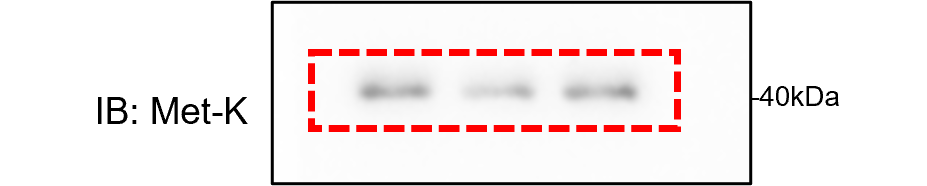

Supplement: Supplementary file 13 — Source data Fig. 5 [file 44318_2025_416_MOESM13_ESM.zip › EMBOJ-2024-119243R_SourceDataForFigure 5/5B/IP-Met-K.tif]

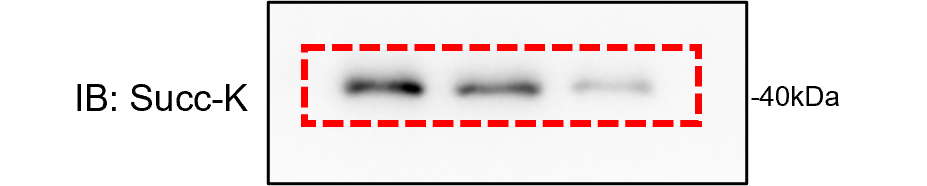

Supplement: Supplementary file 13 — Source data Fig. 5 [file 44318_2025_416_MOESM13_ESM.zip › EMBOJ-2024-119243R_SourceDataForFigure 5/5B/IP-Succ-K.tif]

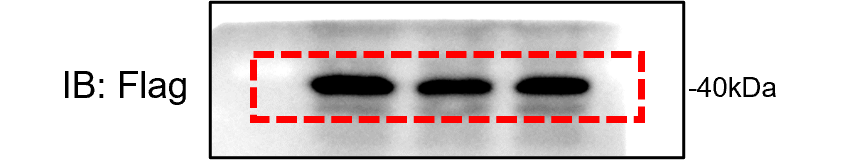

Supplement: Supplementary file 13 — Source data Fig. 5 [file 44318_2025_416_MOESM13_ESM.zip › EMBOJ-2024-119243R_SourceDataForFigure 5/5C/Flag.tif]

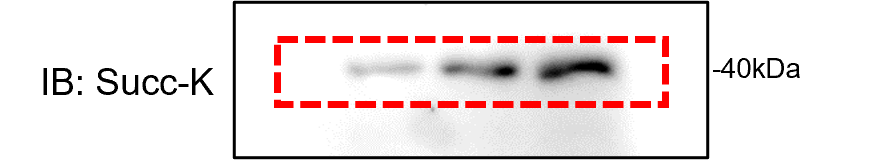

Supplement: Supplementary file 13 — Source data Fig. 5 [file 44318_2025_416_MOESM13_ESM.zip › EMBOJ-2024-119243R_SourceDataForFigure 5/5C/Succ-K.tif]

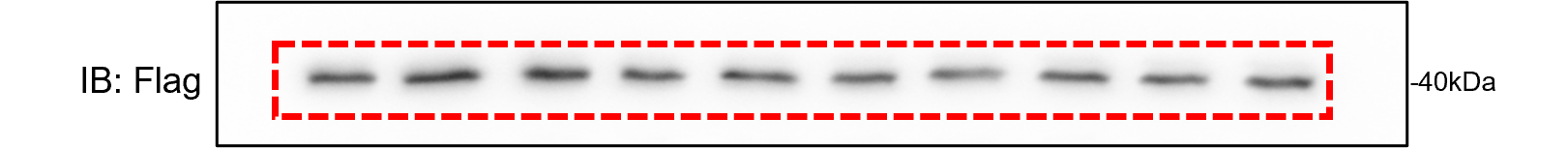

Supplement: Supplementary file 13 — Source data Fig. 5 [file 44318_2025_416_MOESM13_ESM.zip › EMBOJ-2024-119243R_SourceDataForFigure 5/5D/Input-Flag.tif]

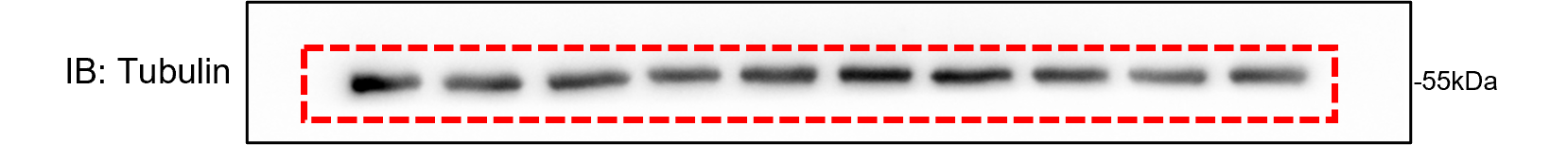

Supplement: Supplementary file 13 — Source data Fig. 5 [file 44318_2025_416_MOESM13_ESM.zip › EMBOJ-2024-119243R_SourceDataForFigure 5/5D/Input-Tubulin.tif]

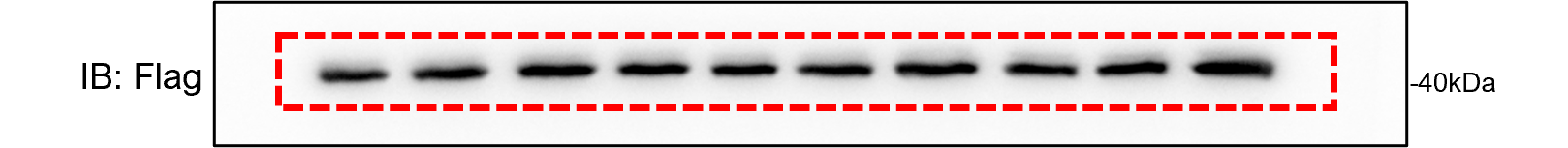

Supplement: Supplementary file 13 — Source data Fig. 5 [file 44318_2025_416_MOESM13_ESM.zip › EMBOJ-2024-119243R_SourceDataForFigure 5/5D/IP-Flag.tif]

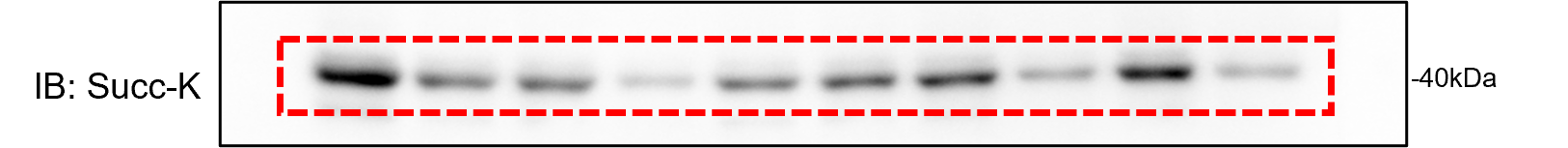

Supplement: Supplementary file 13 — Source data Fig. 5 [file 44318_2025_416_MOESM13_ESM.zip › EMBOJ-2024-119243R_SourceDataForFigure 5/5D/IP-Succ-k.tif]

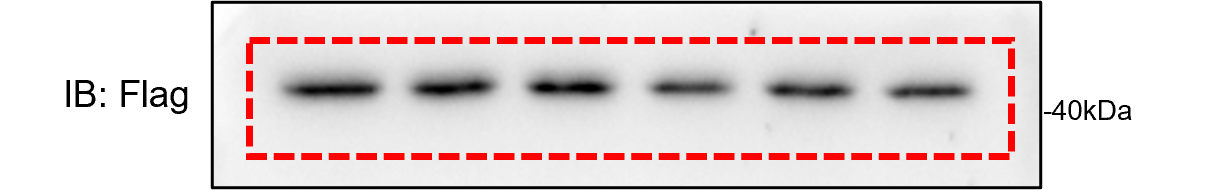

Supplement: Supplementary file 13 — Source data Fig. 5 [file 44318_2025_416_MOESM13_ESM.zip › EMBOJ-2024-119243R_SourceDataForFigure 5/5E/Input-Flag.tif]

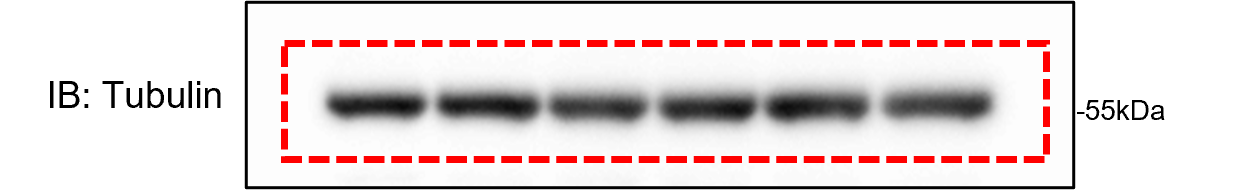

Supplement: Supplementary file 13 — Source data Fig. 5 [file 44318_2025_416_MOESM13_ESM.zip › EMBOJ-2024-119243R_SourceDataForFigure 5/5E/Input-Tubulin.tif]

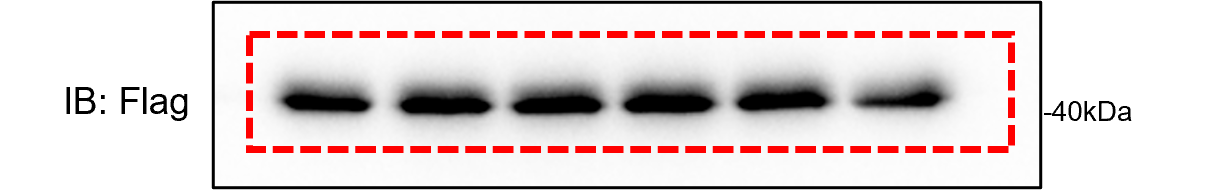

Supplement: Supplementary file 13 — Source data Fig. 5 [file 44318_2025_416_MOESM13_ESM.zip › EMBOJ-2024-119243R_SourceDataForFigure 5/5E/IP-Flag.tif]

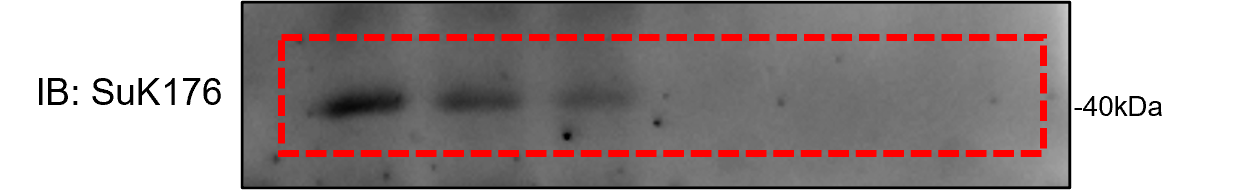

Supplement: Supplementary file 13 — Source data Fig. 5 [file 44318_2025_416_MOESM13_ESM.zip › EMBOJ-2024-119243R_SourceDataForFigure 5/5E/IP-SuK176.tif]

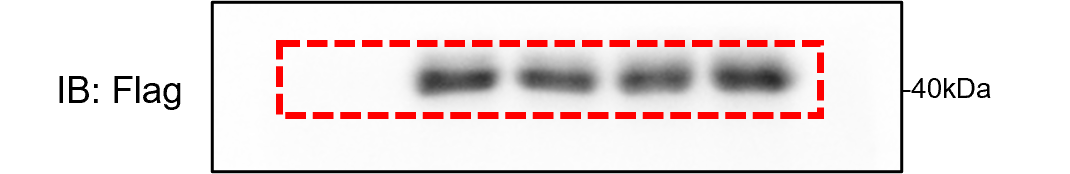

Supplement: Supplementary file 13 — Source data Fig. 5 [file 44318_2025_416_MOESM13_ESM.zip › EMBOJ-2024-119243R_SourceDataForFigure 5/5F/Input-Flag.tif]

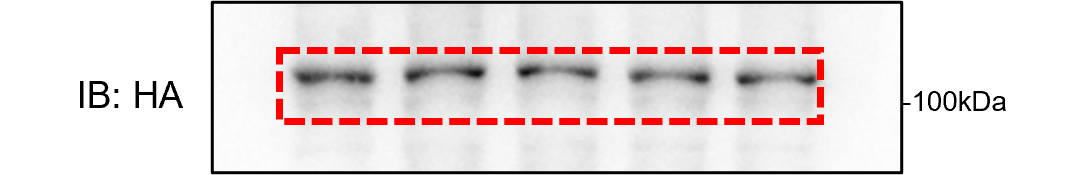

Supplement: Supplementary file 13 — Source data Fig. 5 [file 44318_2025_416_MOESM13_ESM.zip › EMBOJ-2024-119243R_SourceDataForFigure 5/5F/Input-HA.tif]

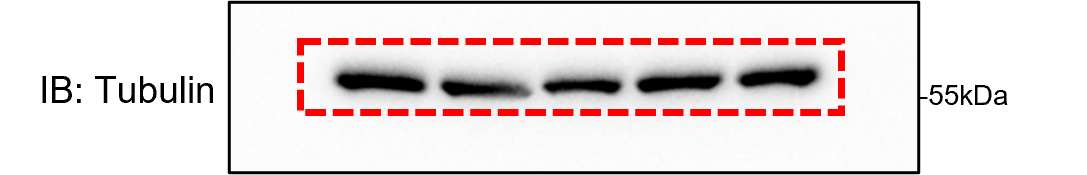

Supplement: Supplementary file 13 — Source data Fig. 5 [file 44318_2025_416_MOESM13_ESM.zip › EMBOJ-2024-119243R_SourceDataForFigure 5/5F/Input-Tubulin.tif]

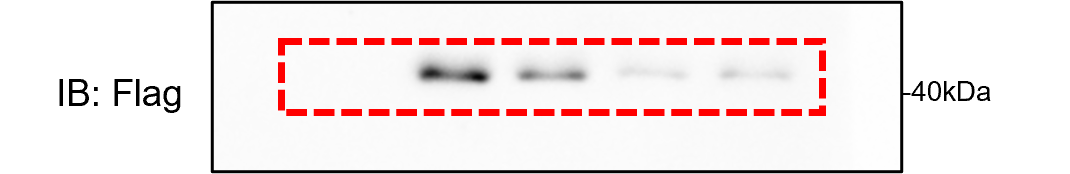

Supplement: Supplementary file 13 — Source data Fig. 5 [file 44318_2025_416_MOESM13_ESM.zip › EMBOJ-2024-119243R_SourceDataForFigure 5/5F/IP-Flag.tif]

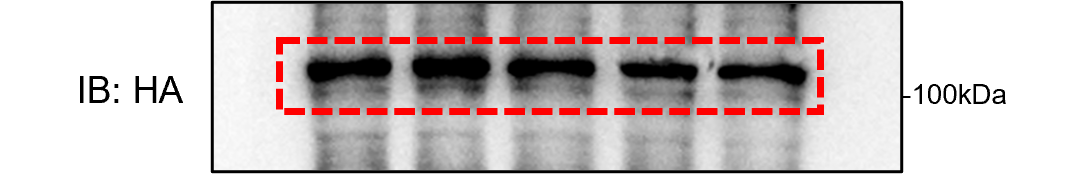

Supplement: Supplementary file 13 — Source data Fig. 5 [file 44318_2025_416_MOESM13_ESM.zip › EMBOJ-2024-119243R_SourceDataForFigure 5/5F/IP-HA.tif]

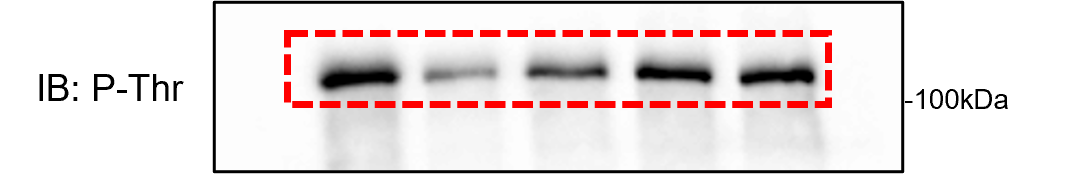

Supplement: Supplementary file 13 — Source data Fig. 5 [file 44318_2025_416_MOESM13_ESM.zip › EMBOJ-2024-119243R_SourceDataForFigure 5/5F/IP-P-Thr.tif]

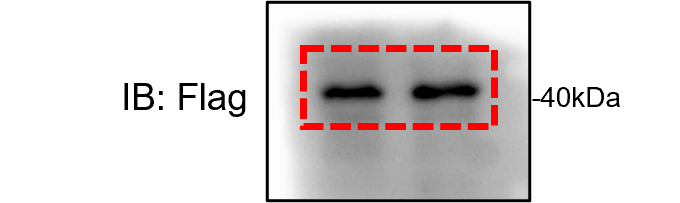

Supplement: Supplementary file 13 — Source data Fig. 5 [file 44318_2025_416_MOESM13_ESM.zip › EMBOJ-2024-119243R_SourceDataForFigure 5/5G/Input-Flag.tif]

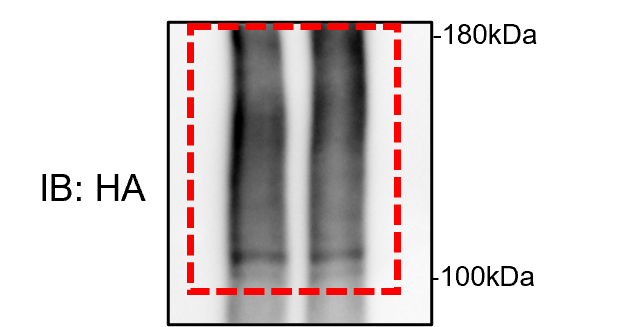

Supplement: Supplementary file 13 — Source data Fig. 5 [file 44318_2025_416_MOESM13_ESM.zip › EMBOJ-2024-119243R_SourceDataForFigure 5/5G/Input-HA.tif]

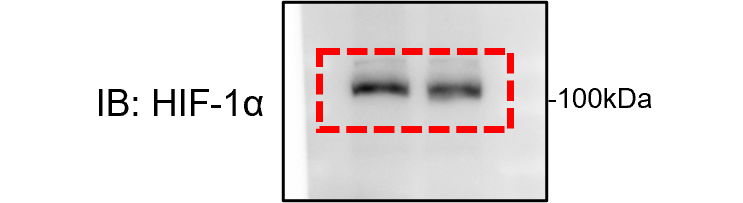

Supplement: Supplementary file 13 — Source data Fig. 5 [file 44318_2025_416_MOESM13_ESM.zip › EMBOJ-2024-119243R_SourceDataForFigure 5/5G/Input-HIF-1α.tif]

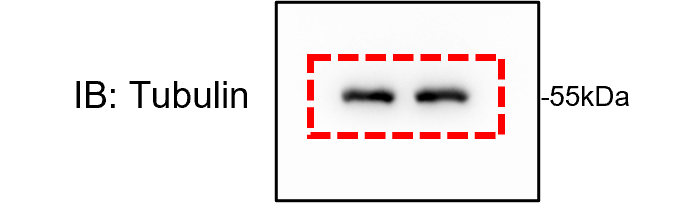

Supplement: Supplementary file 13 — Source data Fig. 5 [file 44318_2025_416_MOESM13_ESM.zip › EMBOJ-2024-119243R_SourceDataForFigure 5/5G/Input-Tubulin.tif]

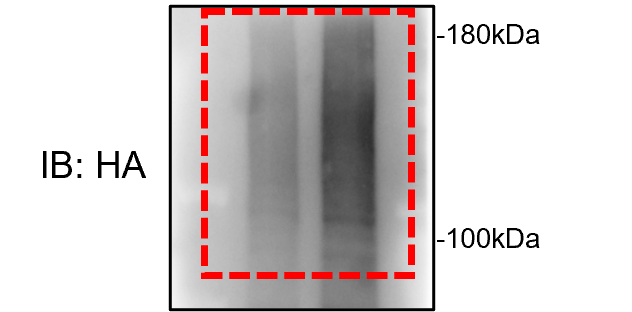

Supplement: Supplementary file 13 — Source data Fig. 5 [file 44318_2025_416_MOESM13_ESM.zip › EMBOJ-2024-119243R_SourceDataForFigure 5/5G/IP-HA.tif]

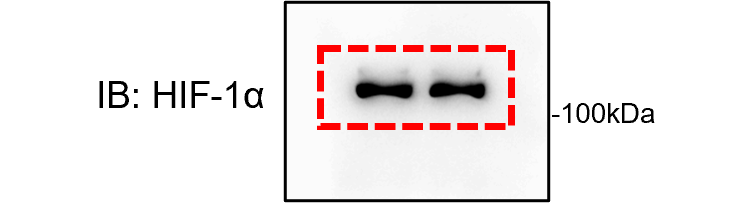

Supplement: Supplementary file 13 — Source data Fig. 5 [file 44318_2025_416_MOESM13_ESM.zip › EMBOJ-2024-119243R_SourceDataForFigure 5/5G/IP-HIF-1α.tif]

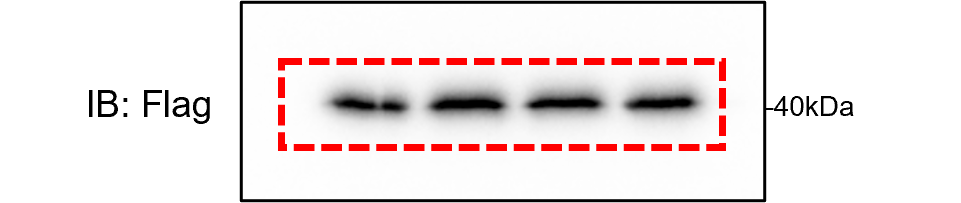

Supplement: Supplementary file 13 — Source data Fig. 5 [file 44318_2025_416_MOESM13_ESM.zip › EMBOJ-2024-119243R_SourceDataForFigure 5/5J/Input-Flag.tif]

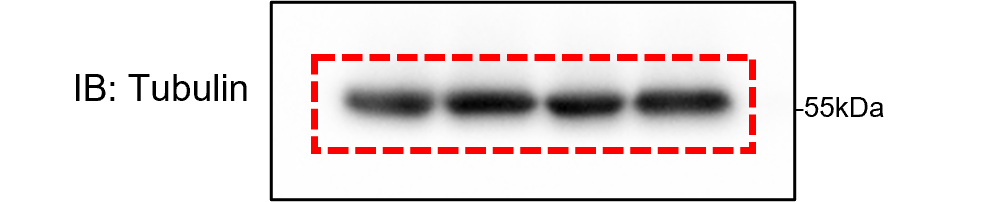

Supplement: Supplementary file 13 — Source data Fig. 5 [file 44318_2025_416_MOESM13_ESM.zip › EMBOJ-2024-119243R_SourceDataForFigure 5/5J/Input-Tubulin.tif]

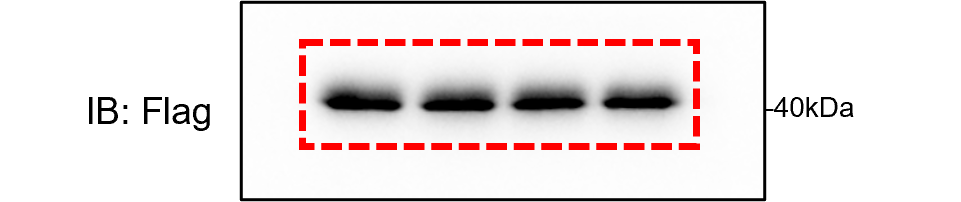

Supplement: Supplementary file 13 — Source data Fig. 5 [file 44318_2025_416_MOESM13_ESM.zip › EMBOJ-2024-119243R_SourceDataForFigure 5/5J/IP-Flag.tif]

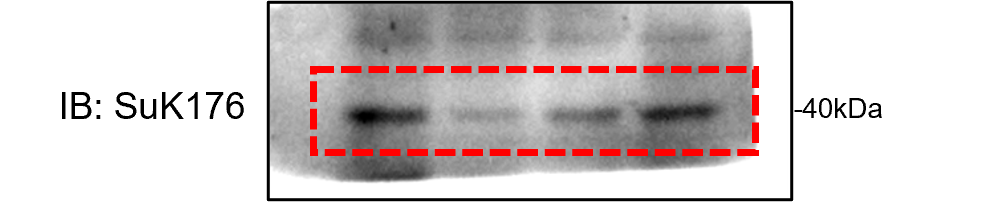

Supplement: Supplementary file 13 — Source data Fig. 5 [file 44318_2025_416_MOESM13_ESM.zip › EMBOJ-2024-119243R_SourceDataForFigure 5/5J/IP-SuK176.tif]

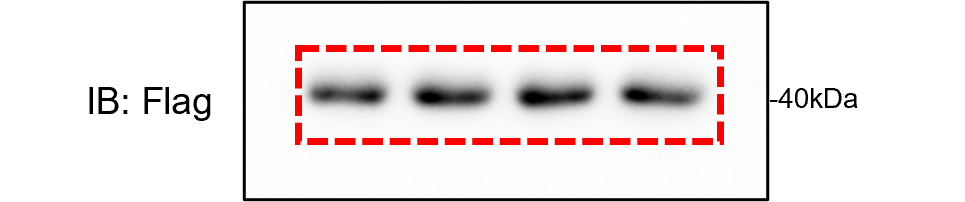

Supplement: Supplementary file 13 — Source data Fig. 5 [file 44318_2025_416_MOESM13_ESM.zip › EMBOJ-2024-119243R_SourceDataForFigure 5/5K/Input-Flag.tif]

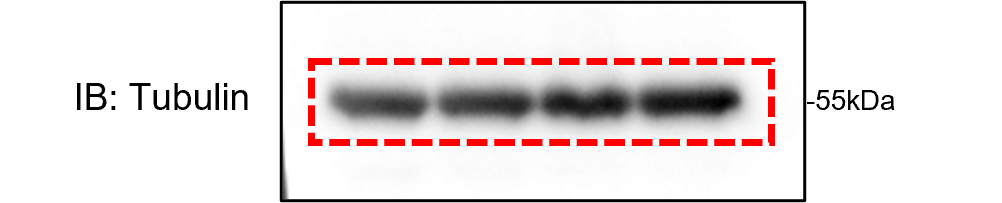

Supplement: Supplementary file 13 — Source data Fig. 5 [file 44318_2025_416_MOESM13_ESM.zip › EMBOJ-2024-119243R_SourceDataForFigure 5/5K/Input-Tubulin.tif]

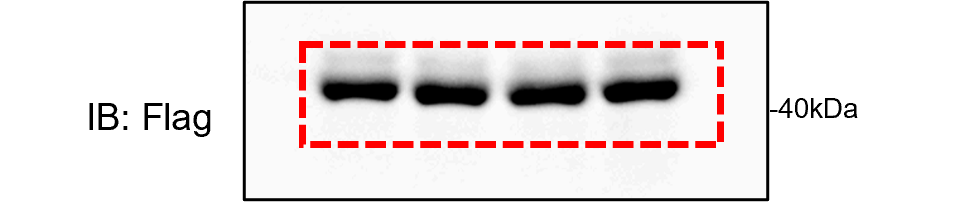

Supplement: Supplementary file 13 — Source data Fig. 5 [file 44318_2025_416_MOESM13_ESM.zip › EMBOJ-2024-119243R_SourceDataForFigure 5/5K/IP-Flag.tif]

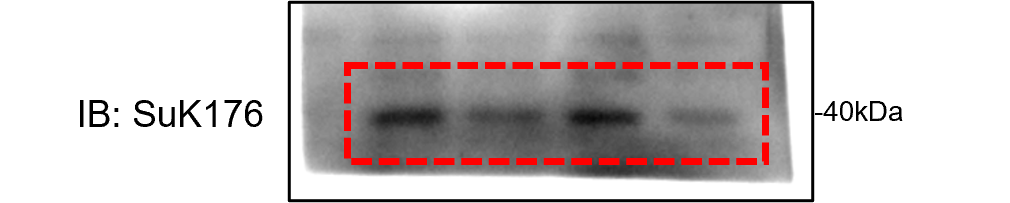

Supplement: Supplementary file 13 — Source data Fig. 5 [file 44318_2025_416_MOESM13_ESM.zip › EMBOJ-2024-119243R_SourceDataForFigure 5/5K/IP-SuK176.tif]

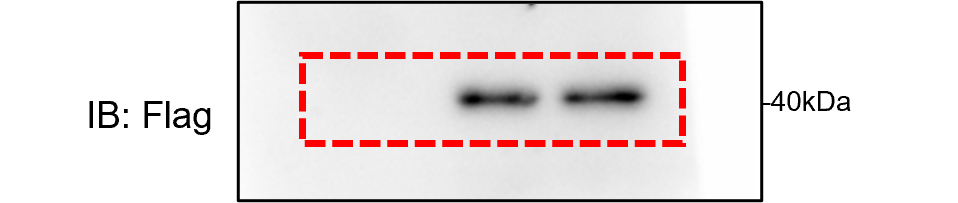

Supplement: Supplementary file 13 — Source data Fig. 5 [file 44318_2025_416_MOESM13_ESM.zip › EMBOJ-2024-119243R_SourceDataForFigure 5/5L/Input-Flag.tif]

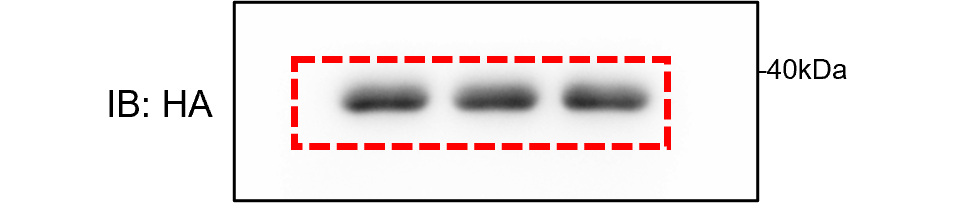

Supplement: Supplementary file 13 — Source data Fig. 5 [file 44318_2025_416_MOESM13_ESM.zip › EMBOJ-2024-119243R_SourceDataForFigure 5/5L/Input-HA.tif]

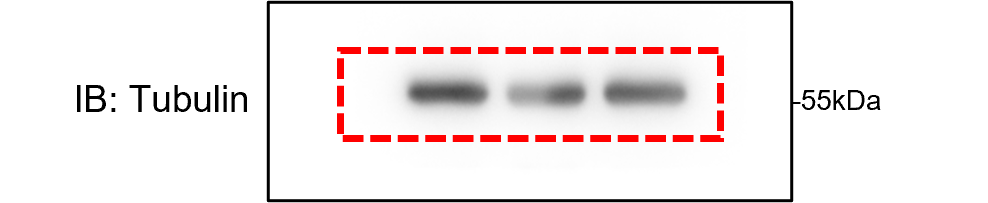

Supplement: Supplementary file 13 — Source data Fig. 5 [file 44318_2025_416_MOESM13_ESM.zip › EMBOJ-2024-119243R_SourceDataForFigure 5/5L/Input-Tubulin.tif]

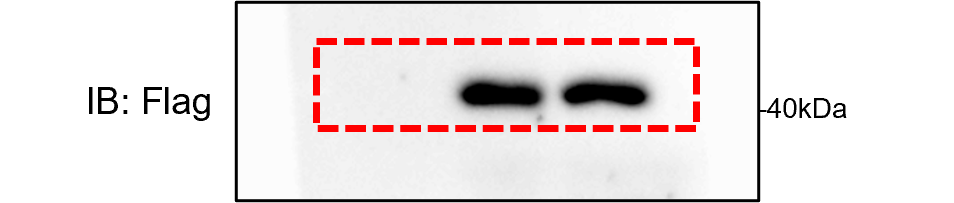

Supplement: Supplementary file 13 — Source data Fig. 5 [file 44318_2025_416_MOESM13_ESM.zip › EMBOJ-2024-119243R_SourceDataForFigure 5/5L/IP-Flag.tif]

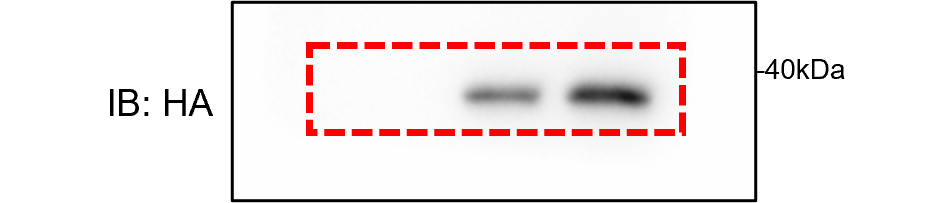

Supplement: Supplementary file 13 — Source data Fig. 5 [file 44318_2025_416_MOESM13_ESM.zip › EMBOJ-2024-119243R_SourceDataForFigure 5/5L/IP-HA.tif]

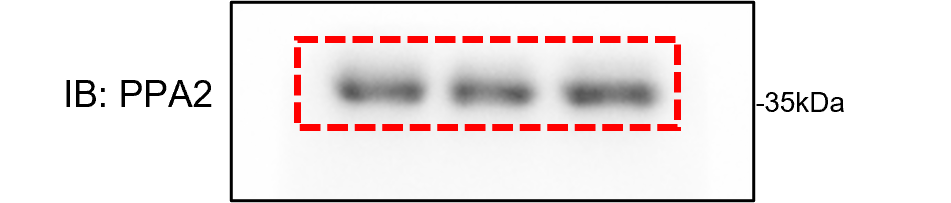

Supplement: Supplementary file 13 — Source data Fig. 5 [file 44318_2025_416_MOESM13_ESM.zip › EMBOJ-2024-119243R_SourceDataForFigure 5/5M/Input-PPA2.tif]

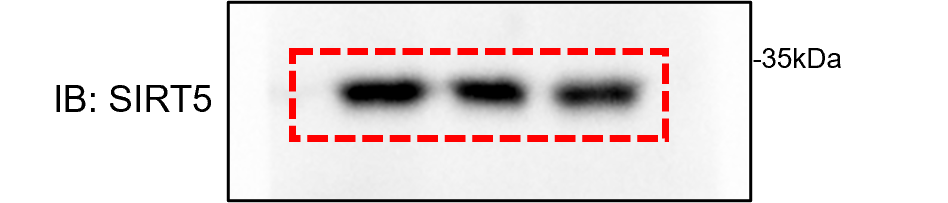

Supplement: Supplementary file 13 — Source data Fig. 5 [file 44318_2025_416_MOESM13_ESM.zip › EMBOJ-2024-119243R_SourceDataForFigure 5/5M/Input-SIRT5.tif]

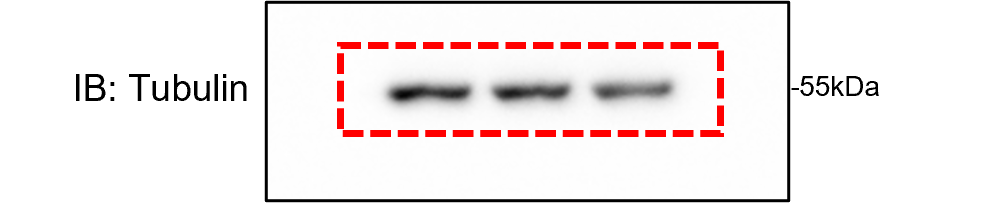

Supplement: Supplementary file 13 — Source data Fig. 5 [file 44318_2025_416_MOESM13_ESM.zip › EMBOJ-2024-119243R_SourceDataForFigure 5/5M/Input-Tubulin.tif]

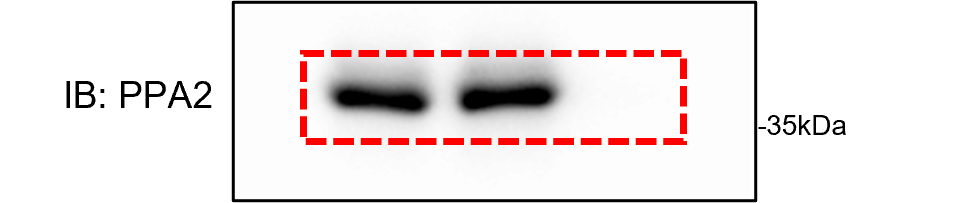

Supplement: Supplementary file 13 — Source data Fig. 5 [file 44318_2025_416_MOESM13_ESM.zip › EMBOJ-2024-119243R_SourceDataForFigure 5/5M/IP-PPA2.tif]

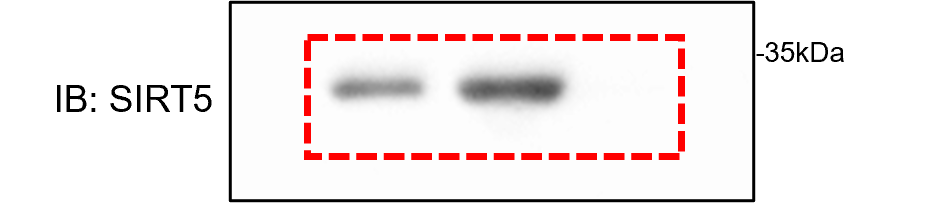

Supplement: Supplementary file 13 — Source data Fig. 5 [file 44318_2025_416_MOESM13_ESM.zip › EMBOJ-2024-119243R_SourceDataForFigure 5/5M/IP-SIRT5.tif]

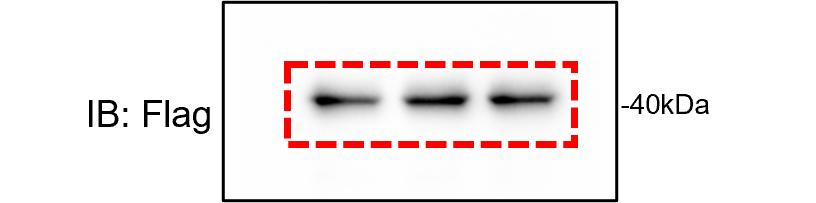

Supplement: Supplementary file 13 — Source data Fig. 5 [file 44318_2025_416_MOESM13_ESM.zip › EMBOJ-2024-119243R_SourceDataForFigure 5/5N/Input-Flag.tif]

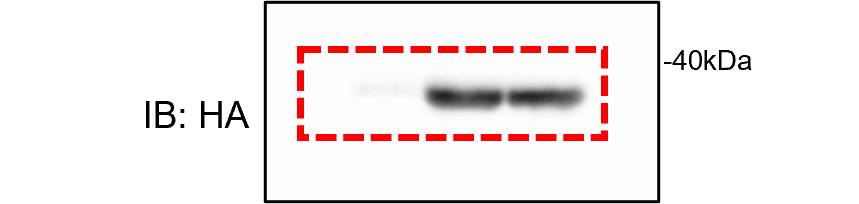

Supplement: Supplementary file 13 — Source data Fig. 5 [file 44318_2025_416_MOESM13_ESM.zip › EMBOJ-2024-119243R_SourceDataForFigure 5/5N/Input-HA.tif]

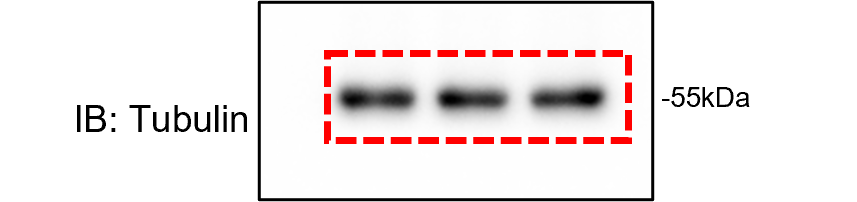

Supplement: Supplementary file 13 — Source data Fig. 5 [file 44318_2025_416_MOESM13_ESM.zip › EMBOJ-2024-119243R_SourceDataForFigure 5/5N/Input-Tubulin.tif]

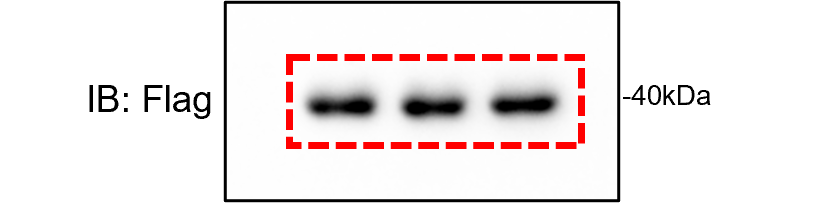

Supplement: Supplementary file 13 — Source data Fig. 5 [file 44318_2025_416_MOESM13_ESM.zip › EMBOJ-2024-119243R_SourceDataForFigure 5/5N/IP-Flag.tif]

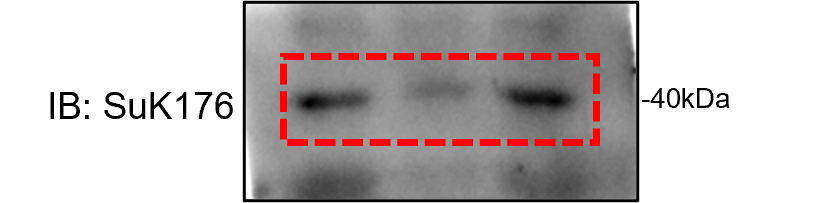

Supplement: Supplementary file 13 — Source data Fig. 5 [file 44318_2025_416_MOESM13_ESM.zip › EMBOJ-2024-119243R_SourceDataForFigure 5/5N/IP-SuK176.tif]

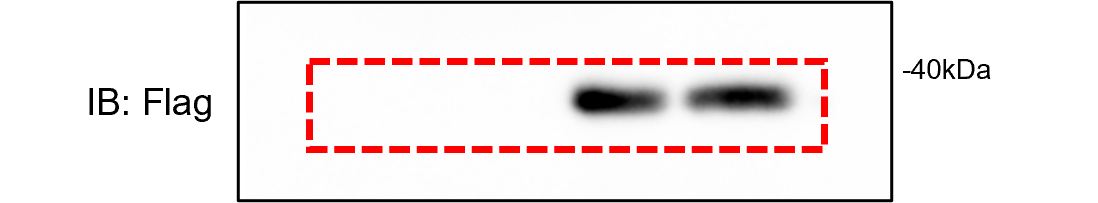

Supplement: Supplementary file 13 — Source data Fig. 5 [file 44318_2025_416_MOESM13_ESM.zip › EMBOJ-2024-119243R_SourceDataForFigure 5/5O/Input-Flag.tif]

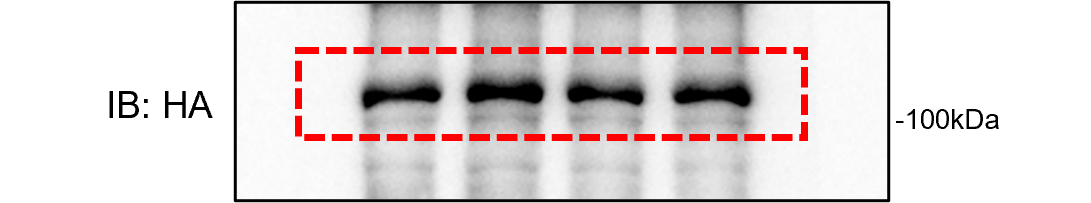

Supplement: Supplementary file 13 — Source data Fig. 5 [file 44318_2025_416_MOESM13_ESM.zip › EMBOJ-2024-119243R_SourceDataForFigure 5/5O/Input-HA.tif]

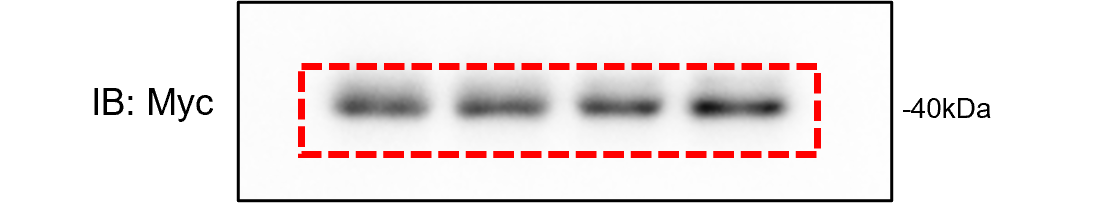

Supplement: Supplementary file 13 — Source data Fig. 5 [file 44318_2025_416_MOESM13_ESM.zip › EMBOJ-2024-119243R_SourceDataForFigure 5/5O/Input-Myc.tif]

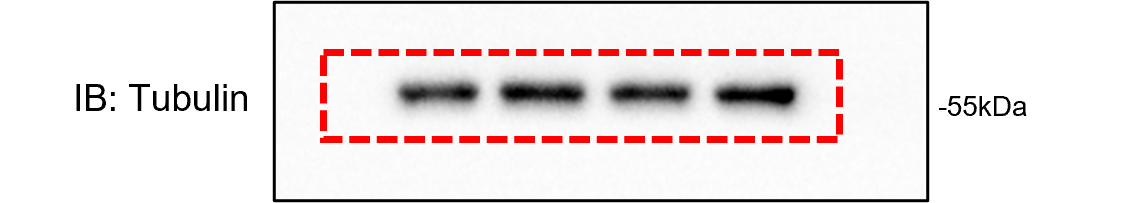

Supplement: Supplementary file 13 — Source data Fig. 5 [file 44318_2025_416_MOESM13_ESM.zip › EMBOJ-2024-119243R_SourceDataForFigure 5/5O/Input-Tubulin.tif]

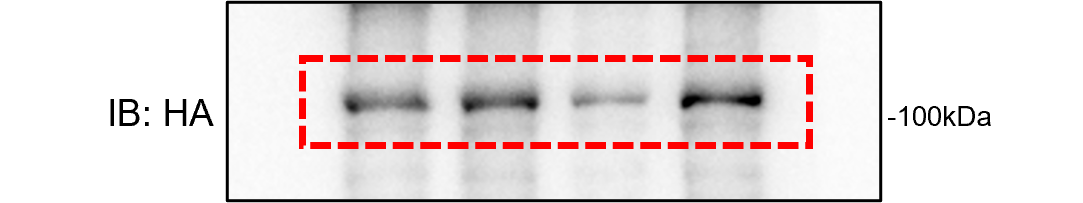

Supplement: Supplementary file 13 — Source data Fig. 5 [file 44318_2025_416_MOESM13_ESM.zip › EMBOJ-2024-119243R_SourceDataForFigure 5/5O/IP-HA.tif]

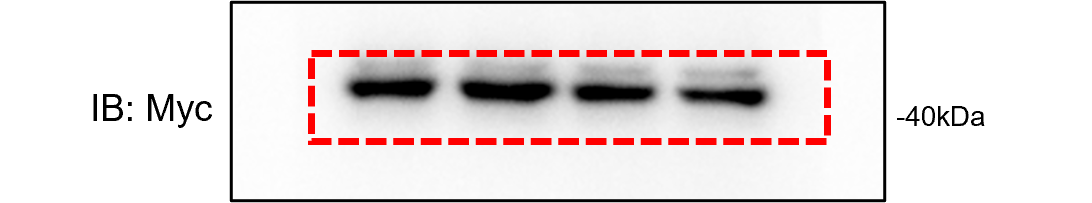

Supplement: Supplementary file 13 — Source data Fig. 5 [file 44318_2025_416_MOESM13_ESM.zip › EMBOJ-2024-119243R_SourceDataForFigure 5/5O/IP-Myc.tif]

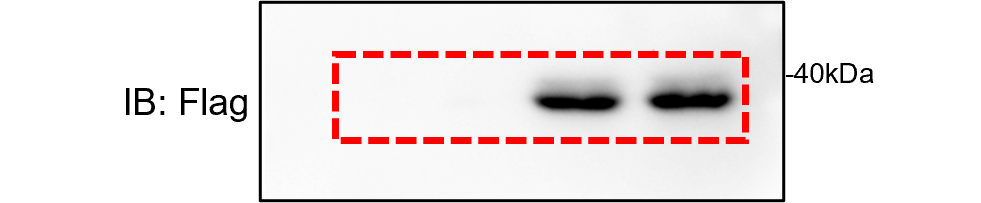

Supplement: Supplementary file 13 — Source data Fig. 5 [file 44318_2025_416_MOESM13_ESM.zip › EMBOJ-2024-119243R_SourceDataForFigure 5/5P/Input-Flag.tif]

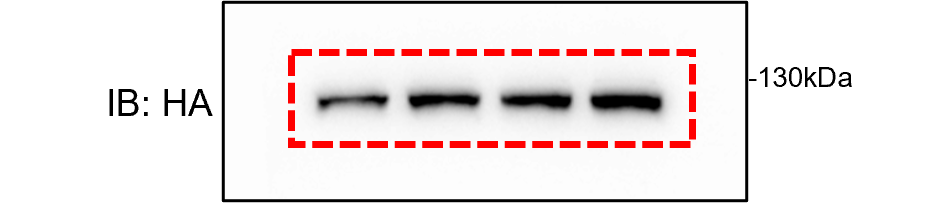

Supplement: Supplementary file 13 — Source data Fig. 5 [file 44318_2025_416_MOESM13_ESM.zip › EMBOJ-2024-119243R_SourceDataForFigure 5/5P/Input-HA.tif]

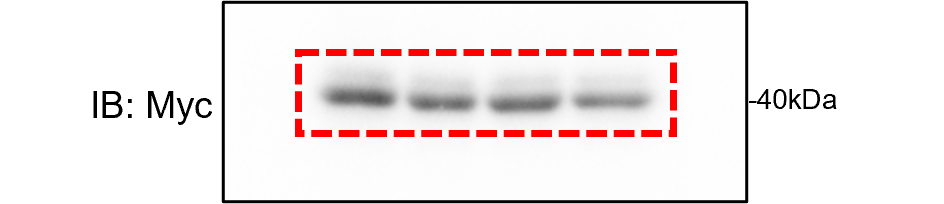

Supplement: Supplementary file 13 — Source data Fig. 5 [file 44318_2025_416_MOESM13_ESM.zip › EMBOJ-2024-119243R_SourceDataForFigure 5/5P/Input-Myc.tif]

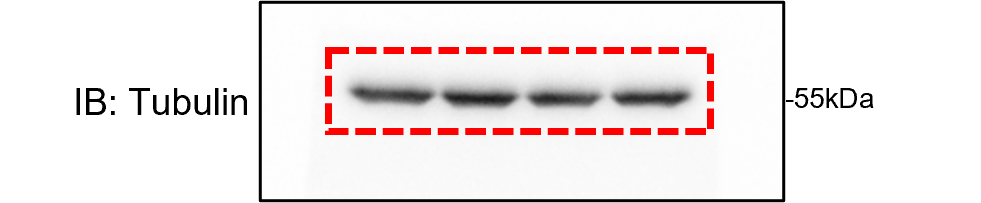

Supplement: Supplementary file 13 — Source data Fig. 5 [file 44318_2025_416_MOESM13_ESM.zip › EMBOJ-2024-119243R_SourceDataForFigure 5/5P/Input-Tubulin.tif]

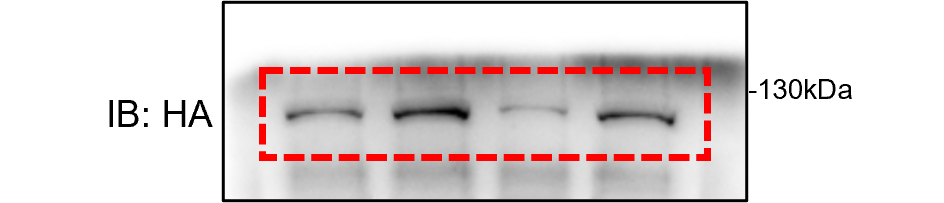

Supplement: Supplementary file 13 — Source data Fig. 5 [file 44318_2025_416_MOESM13_ESM.zip › EMBOJ-2024-119243R_SourceDataForFigure 5/5P/IP-HA.tif]

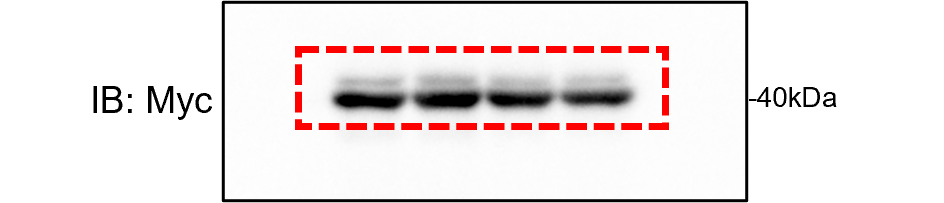

Supplement: Supplementary file 13 — Source data Fig. 5 [file 44318_2025_416_MOESM13_ESM.zip › EMBOJ-2024-119243R_SourceDataForFigure 5/5P/IP-Myc.tif]

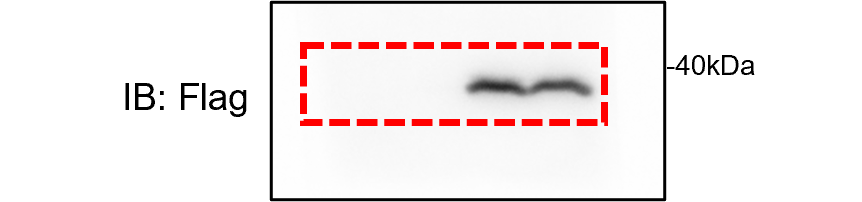

Supplement: Supplementary file 13 — Source data Fig. 5 [file 44318_2025_416_MOESM13_ESM.zip › EMBOJ-2024-119243R_SourceDataForFigure 5/5Q/Input-Flag.tif]

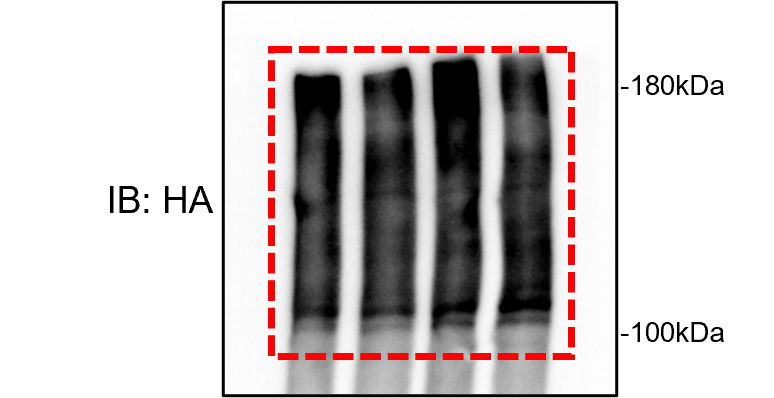

Supplement: Supplementary file 13 — Source data Fig. 5 [file 44318_2025_416_MOESM13_ESM.zip › EMBOJ-2024-119243R_SourceDataForFigure 5/5Q/Input-HA.tif]

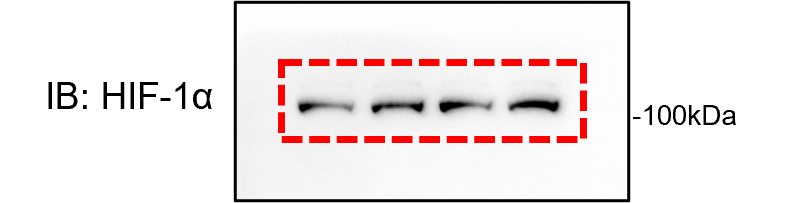

Supplement: Supplementary file 13 — Source data Fig. 5 [file 44318_2025_416_MOESM13_ESM.zip › EMBOJ-2024-119243R_SourceDataForFigure 5/5Q/Input-HIF-1α.tif]

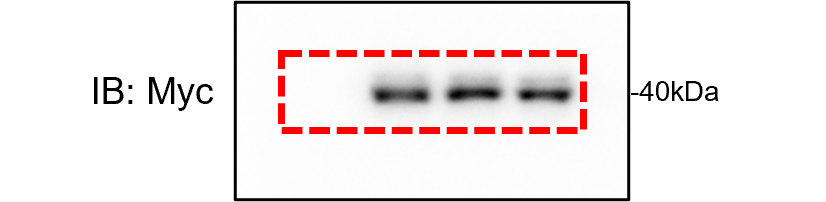

Supplement: Supplementary file 13 — Source data Fig. 5 [file 44318_2025_416_MOESM13_ESM.zip › EMBOJ-2024-119243R_SourceDataForFigure 5/5Q/Input-Myc.tif]

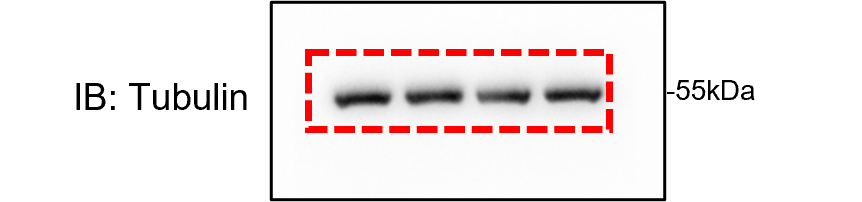

Supplement: Supplementary file 13 — Source data Fig. 5 [file 44318_2025_416_MOESM13_ESM.zip › EMBOJ-2024-119243R_SourceDataForFigure 5/5Q/Input-Tubulin.tif]

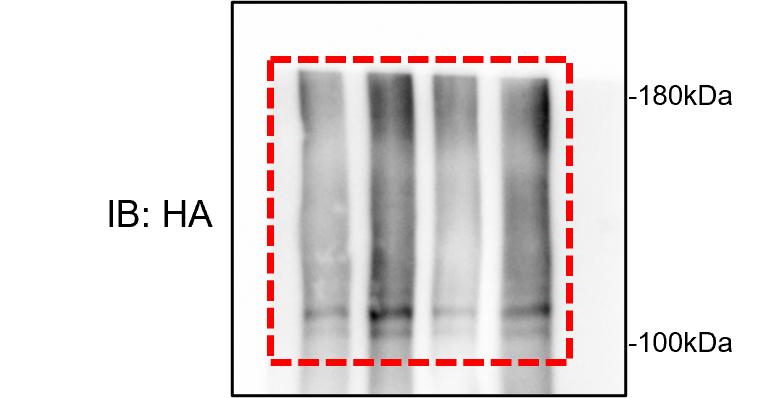

Supplement: Supplementary file 13 — Source data Fig. 5 [file 44318_2025_416_MOESM13_ESM.zip › EMBOJ-2024-119243R_SourceDataForFigure 5/5Q/IP-HA.tif]

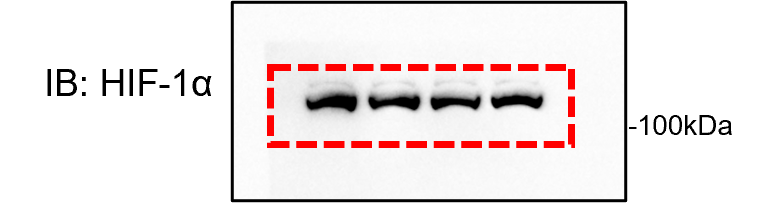

Supplement: Supplementary file 13 — Source data Fig. 5 [file 44318_2025_416_MOESM13_ESM.zip › EMBOJ-2024-119243R_SourceDataForFigure 5/5Q/IP-HIF-1α.tif]

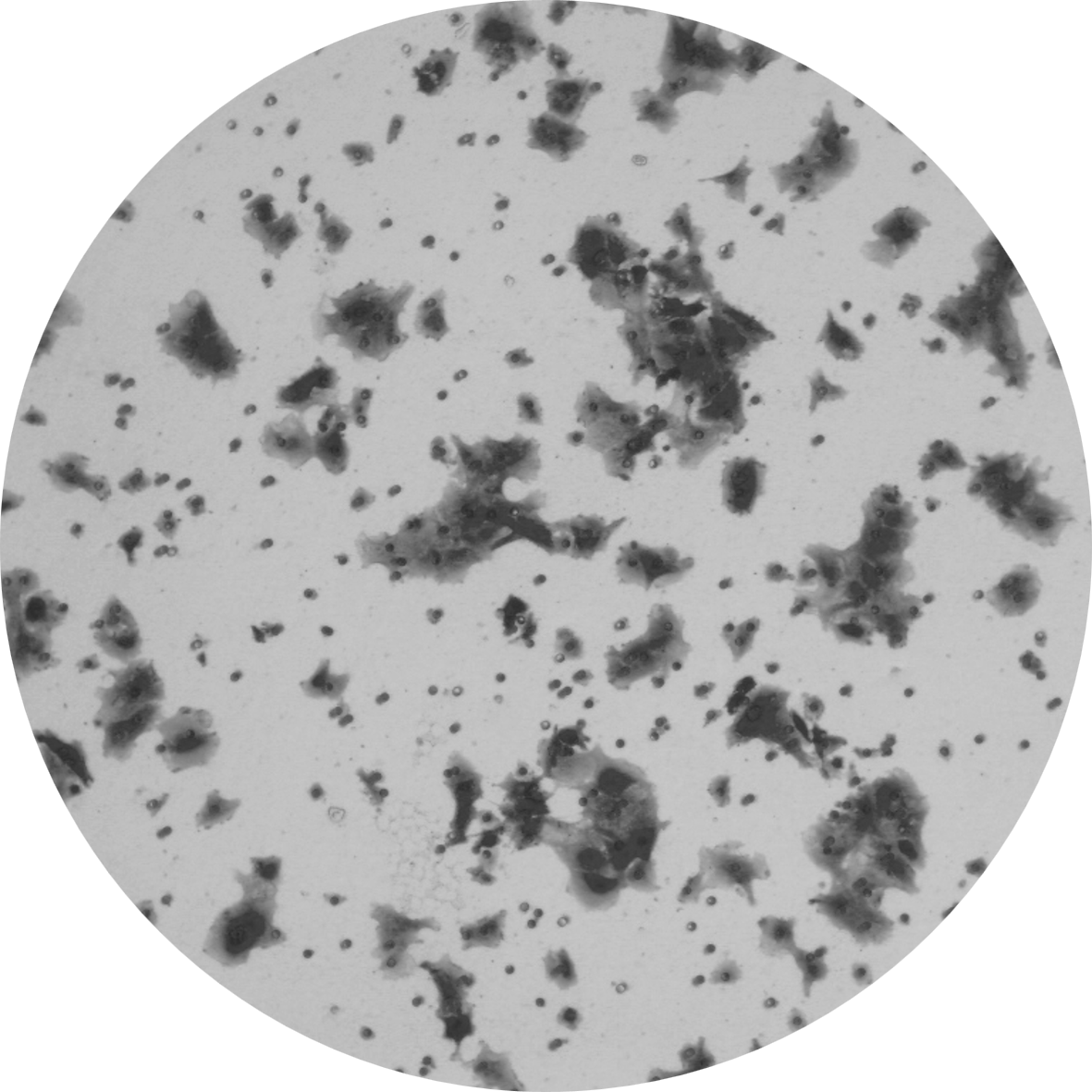

Supplement: Supplementary file 14 — Source data Fig. 6 [file 44318_2025_416_MOESM14_ESM.zip › EMBOJ-2024-119243R_SourceDataForFigure 6/6A/DLD1-HO-K176E-Migration.tif]

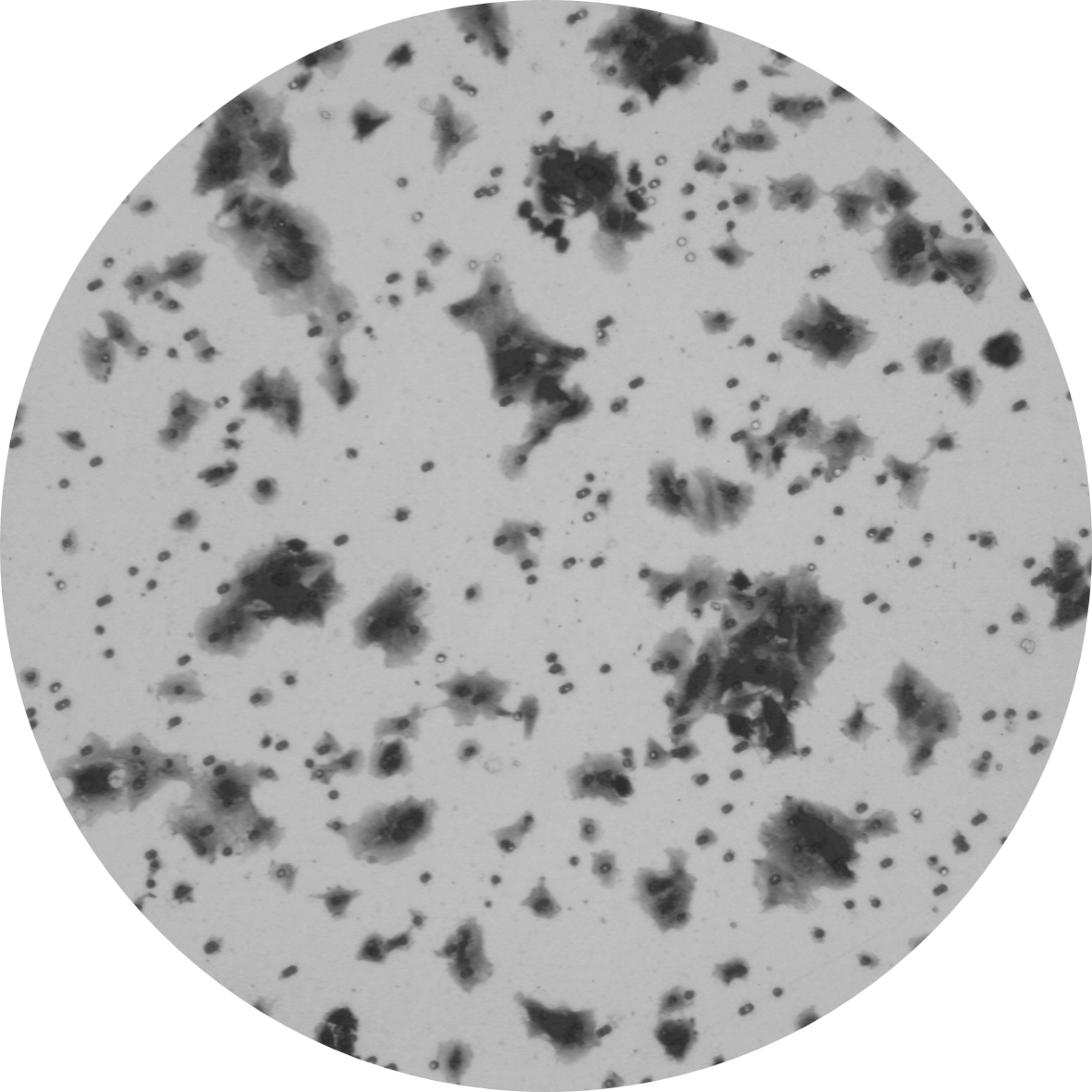

Supplement: Supplementary file 14 — Source data Fig. 6 [file 44318_2025_416_MOESM14_ESM.zip › EMBOJ-2024-119243R_SourceDataForFigure 6/6A/DLD1-HO-WT-Migration.tif]

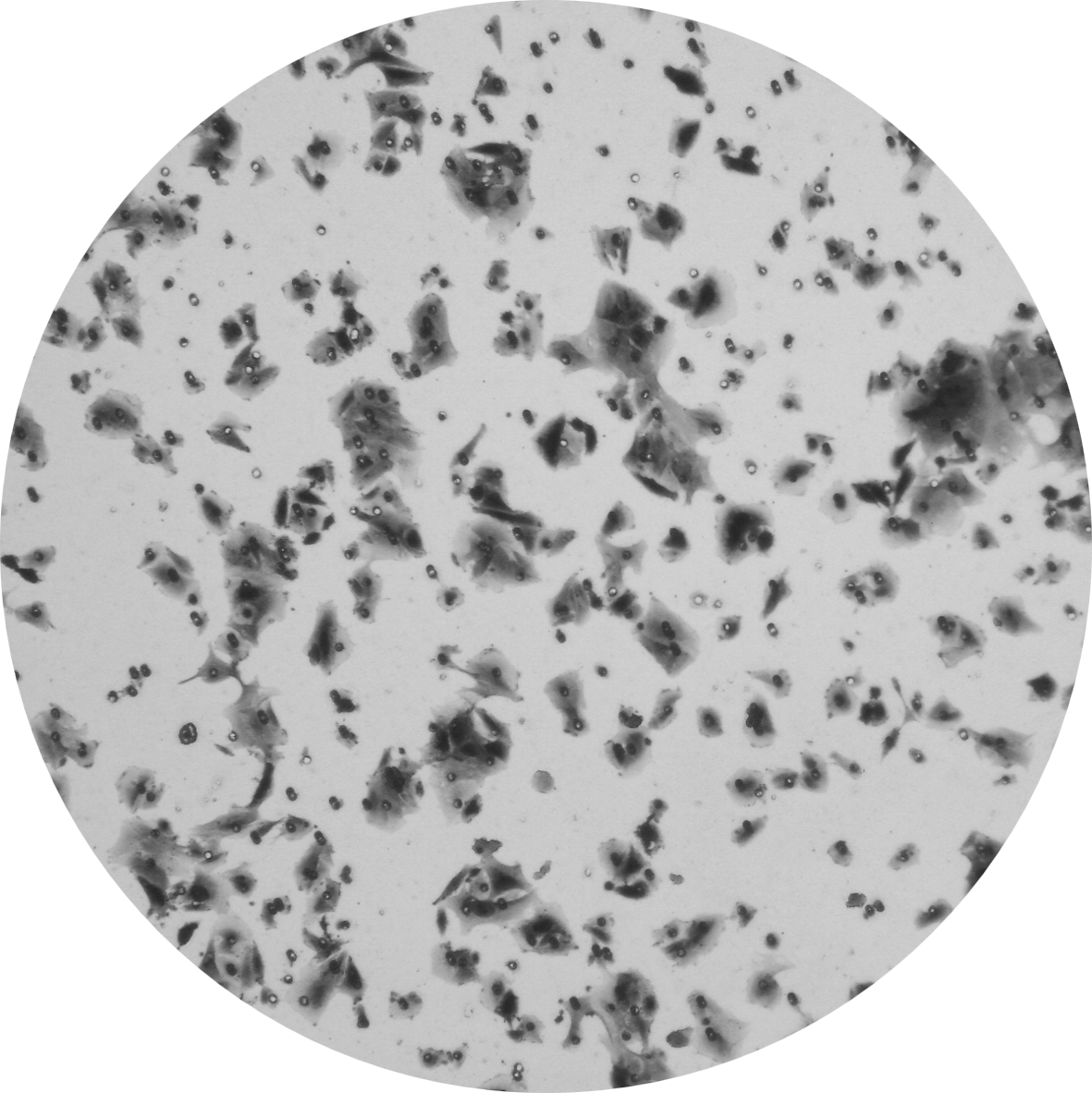

Supplement: Supplementary file 14 — Source data Fig. 6 [file 44318_2025_416_MOESM14_ESM.zip › EMBOJ-2024-119243R_SourceDataForFigure 6/6A/DLD1-LO-K176E-Migration.tif]

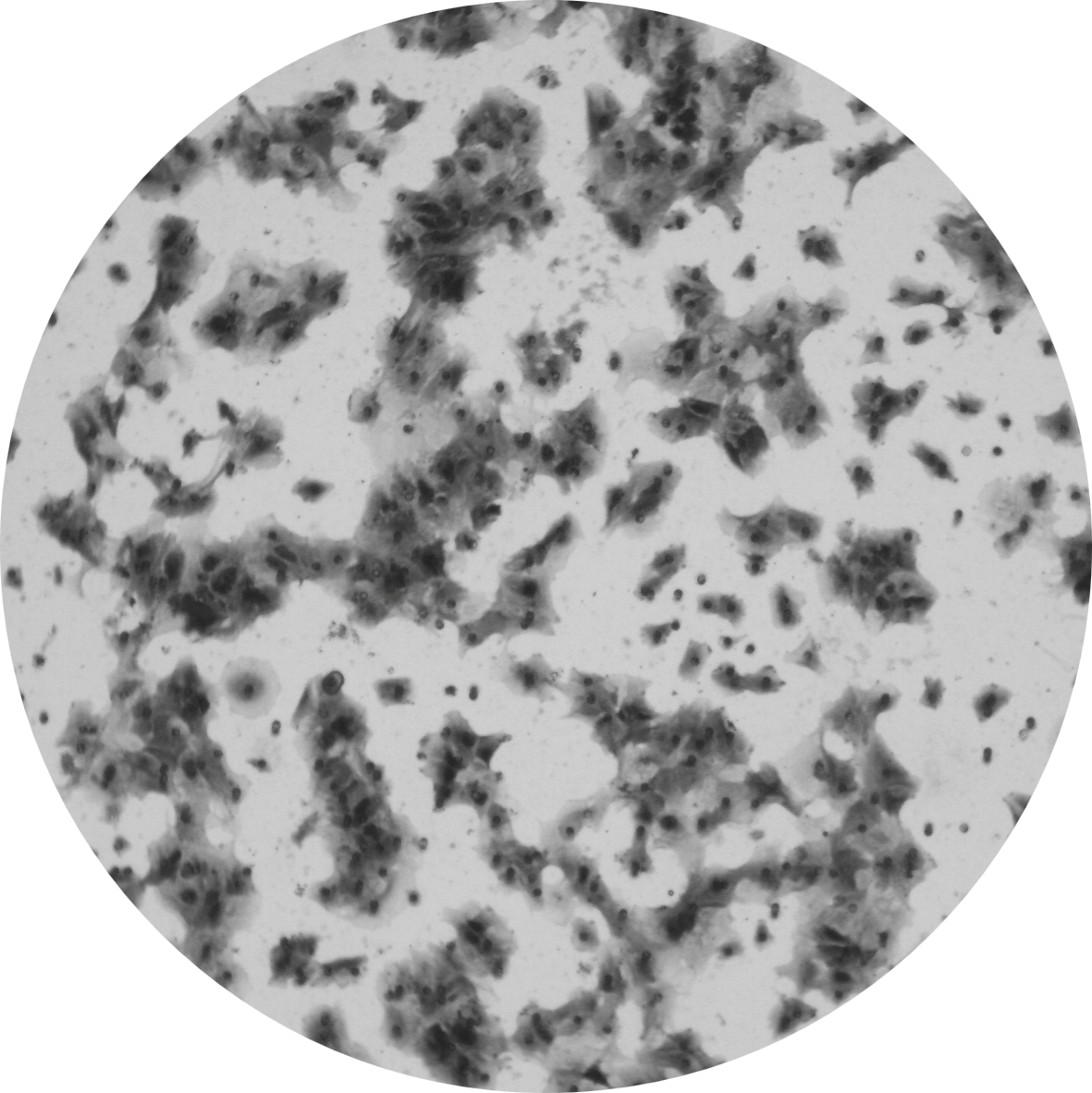

Supplement: Supplementary file 14 — Source data Fig. 6 [file 44318_2025_416_MOESM14_ESM.zip › EMBOJ-2024-119243R_SourceDataForFigure 6/6A/DLD1-LO-WT-Migration.tif]

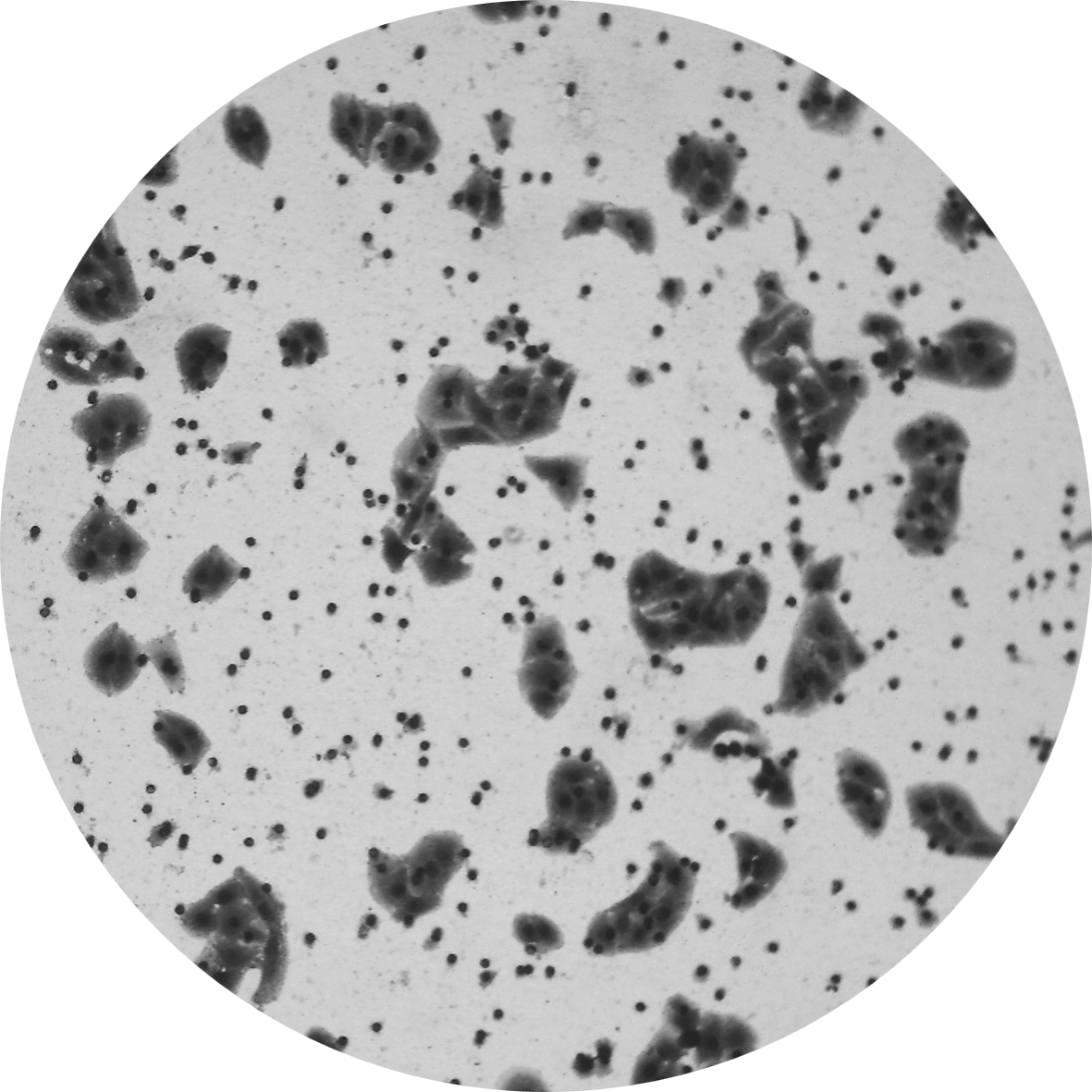

Supplement: Supplementary file 14 — Source data Fig. 6 [file 44318_2025_416_MOESM14_ESM.zip › EMBOJ-2024-119243R_SourceDataForFigure 6/6A/SW1116-HO-K176E-Migration.tif]

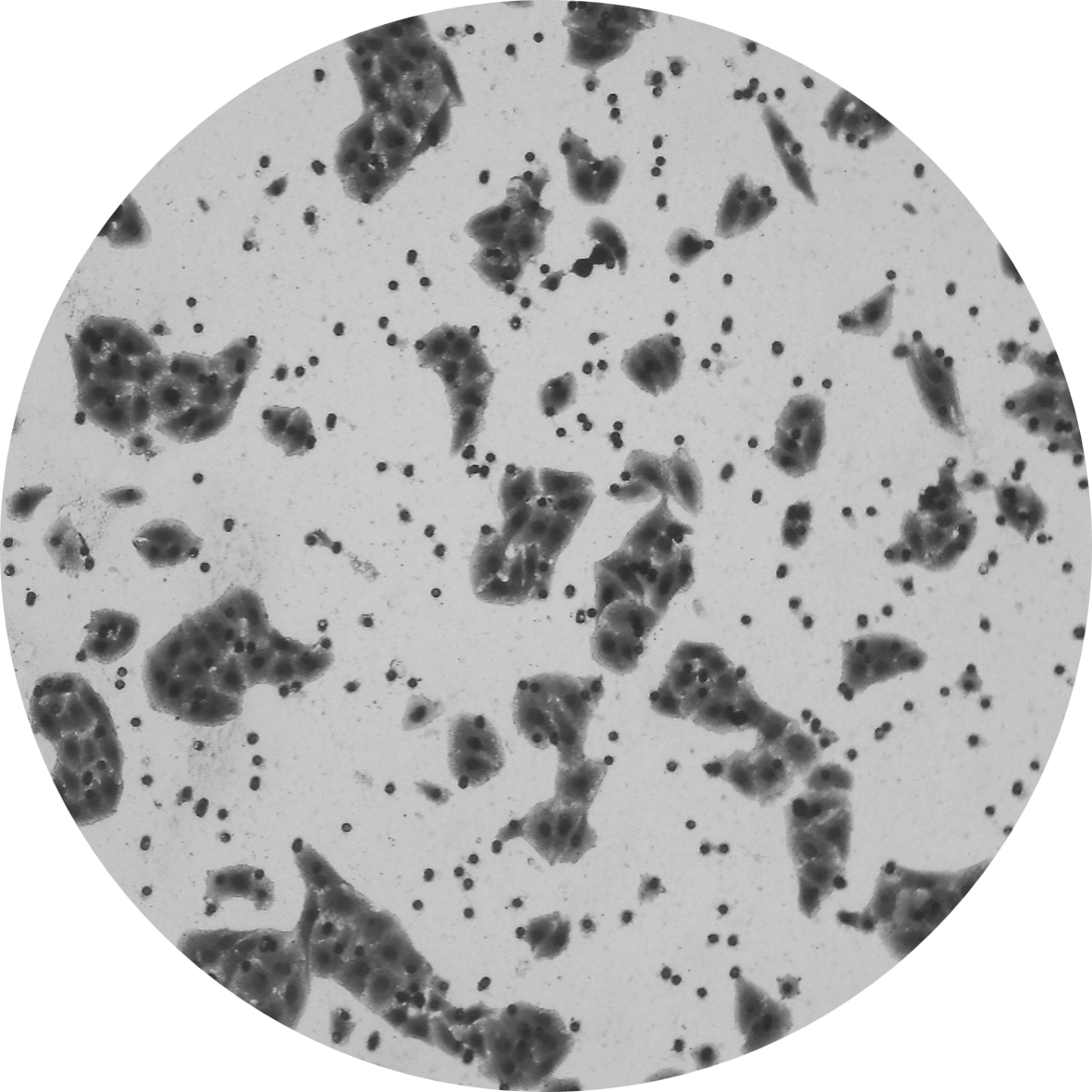

Supplement: Supplementary file 14 — Source data Fig. 6 [file 44318_2025_416_MOESM14_ESM.zip › EMBOJ-2024-119243R_SourceDataForFigure 6/6A/SW1116-HO-WT-Migration.tif]

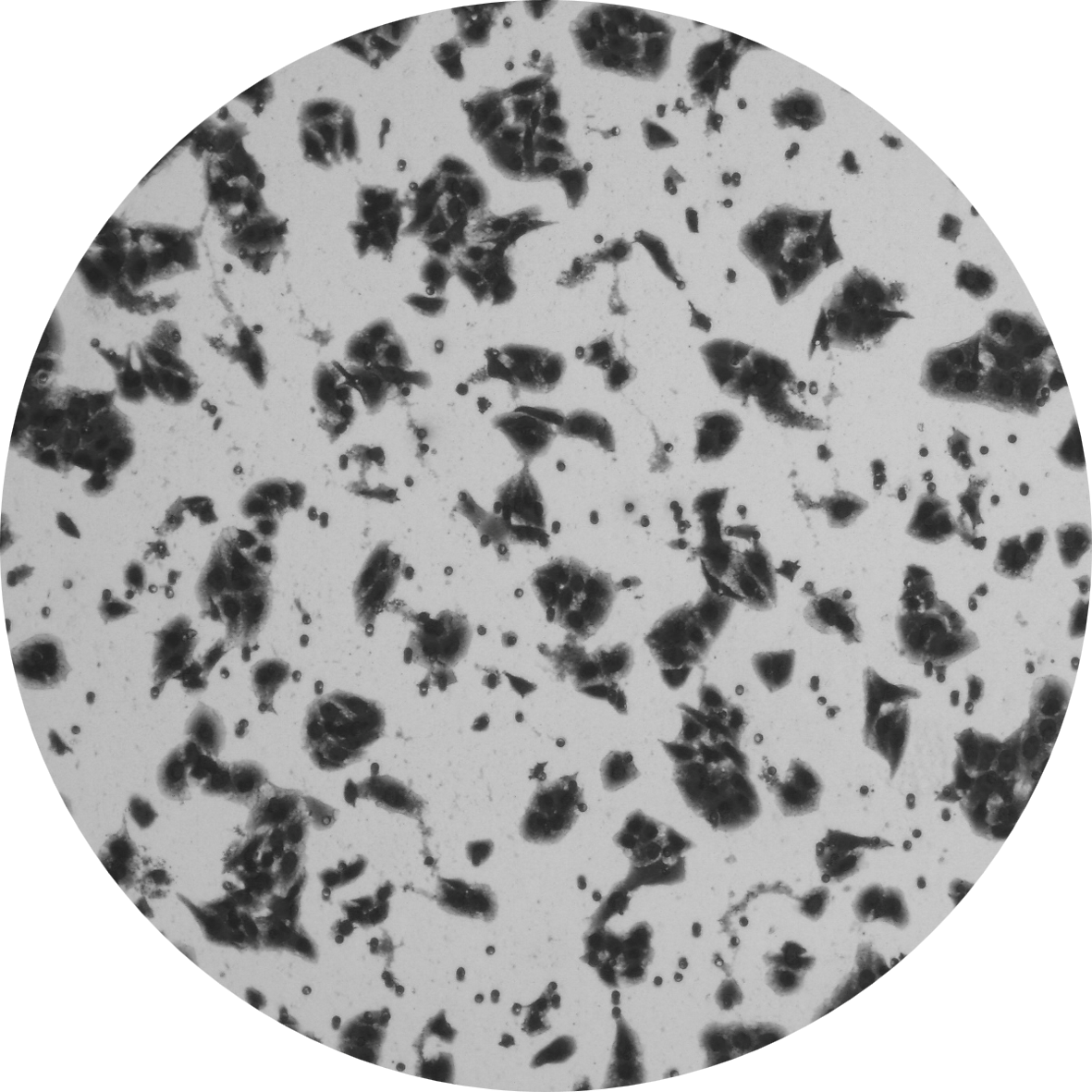

Supplement: Supplementary file 14 — Source data Fig. 6 [file 44318_2025_416_MOESM14_ESM.zip › EMBOJ-2024-119243R_SourceDataForFigure 6/6A/SW1116-LO-K176E-Migration.tif]

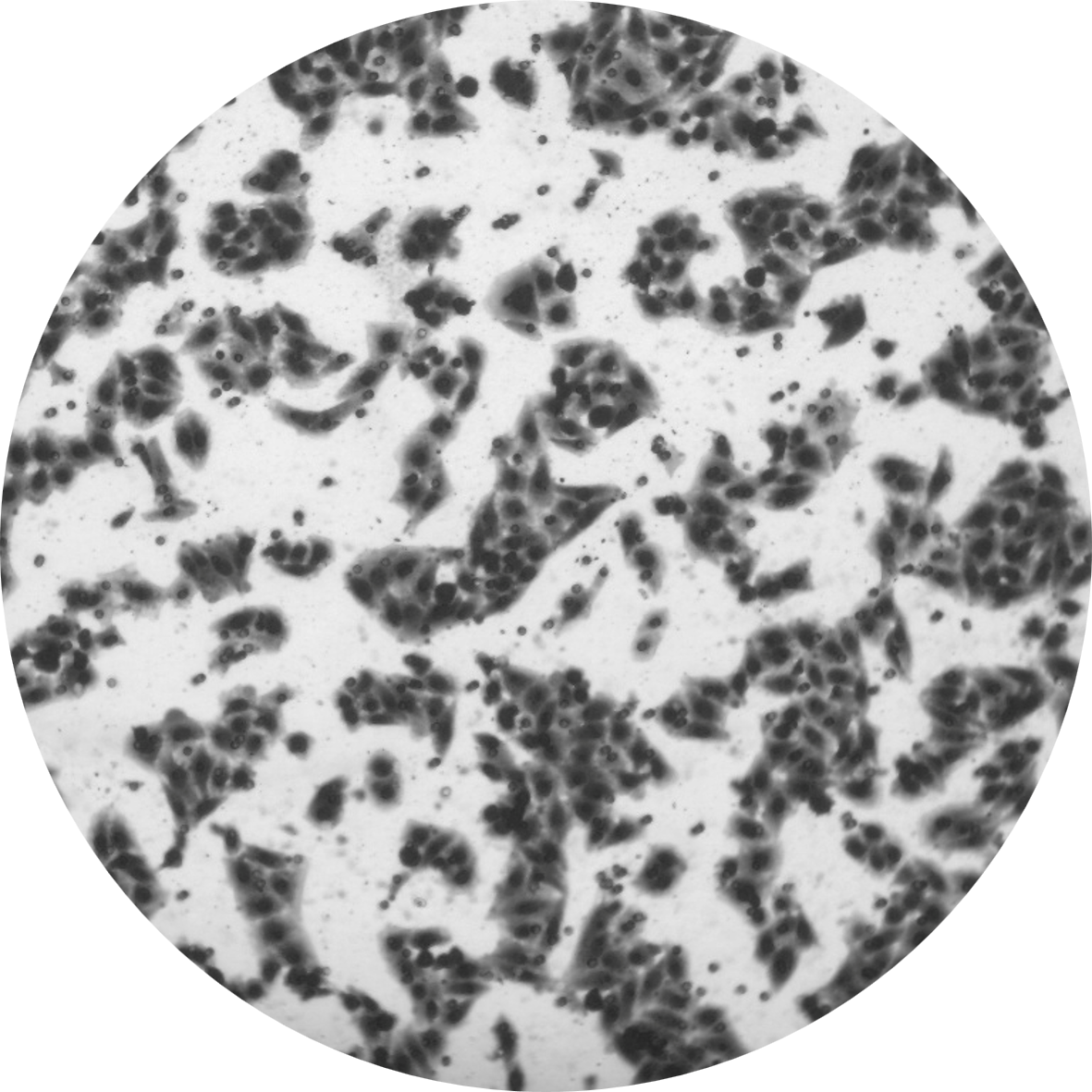

Supplement: Supplementary file 14 — Source data Fig. 6 [file 44318_2025_416_MOESM14_ESM.zip › EMBOJ-2024-119243R_SourceDataForFigure 6/6A/SW1116-LO-WT-Migration.tif]

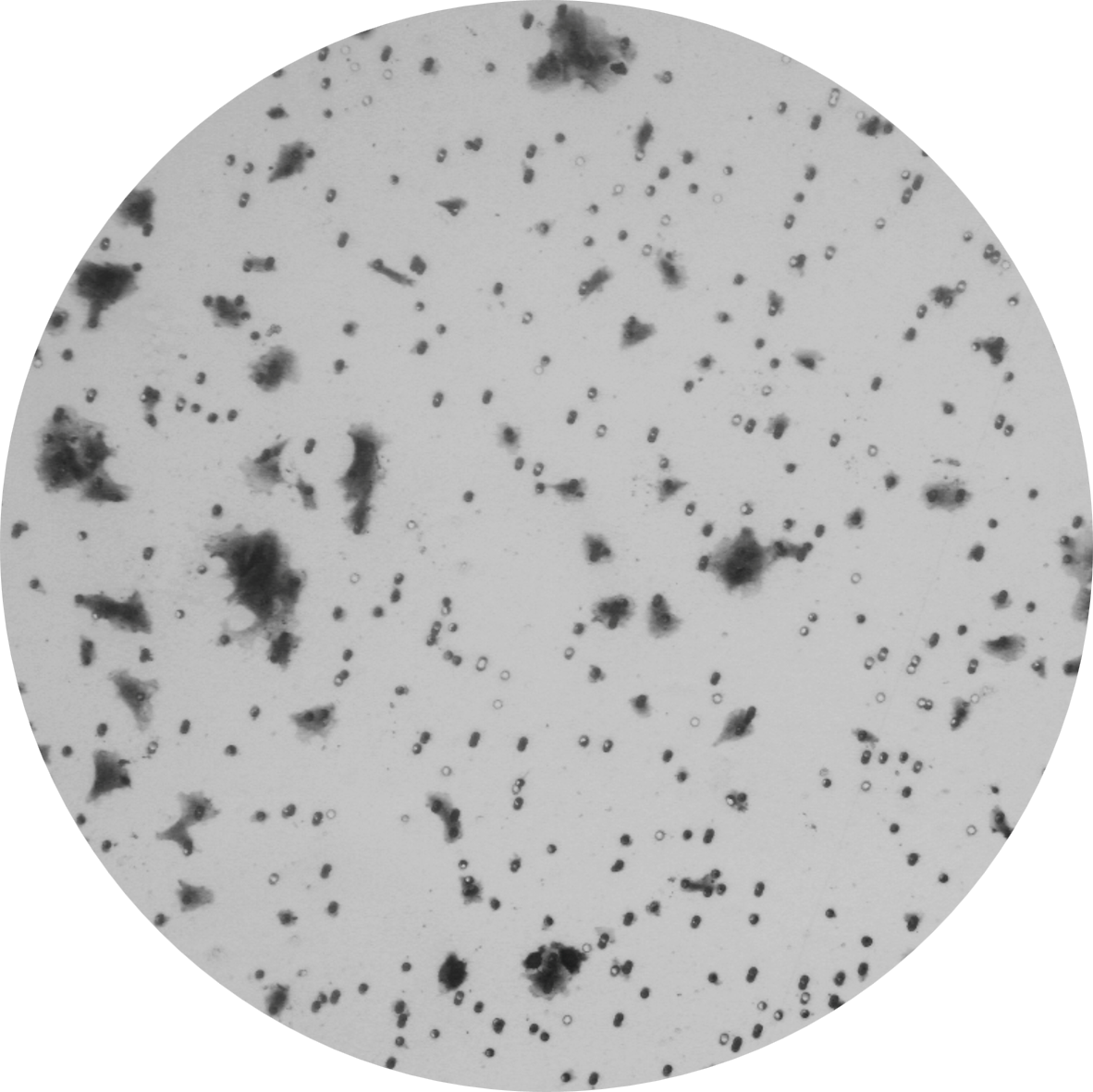

Supplement: Supplementary file 14 — Source data Fig. 6 [file 44318_2025_416_MOESM14_ESM.zip › EMBOJ-2024-119243R_SourceDataForFigure 6/6B/DLD1-HO-K176E-Invasion.tif]

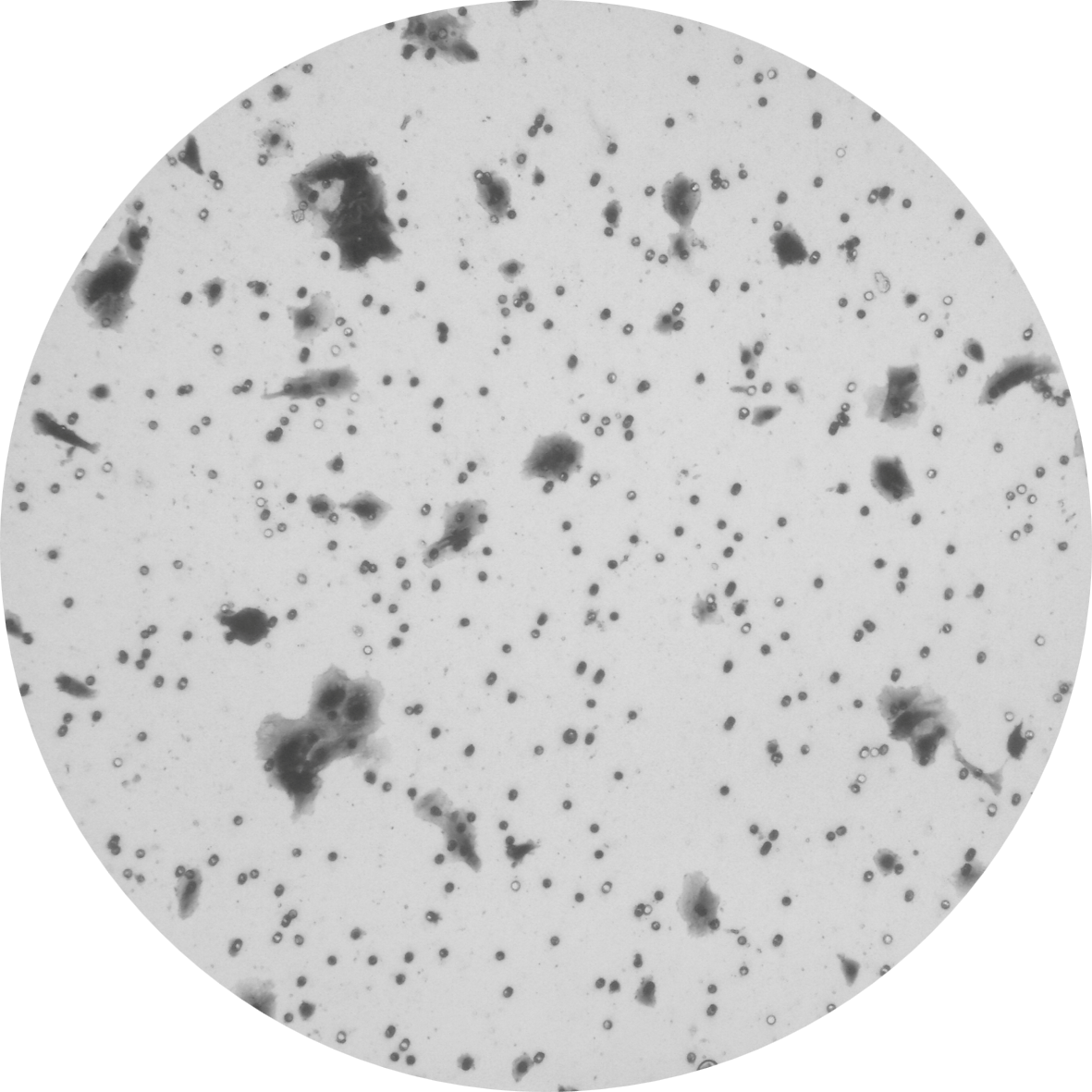

Supplement: Supplementary file 14 — Source data Fig. 6 [file 44318_2025_416_MOESM14_ESM.zip › EMBOJ-2024-119243R_SourceDataForFigure 6/6B/DLD1-HO-WT-Invasion.tif]

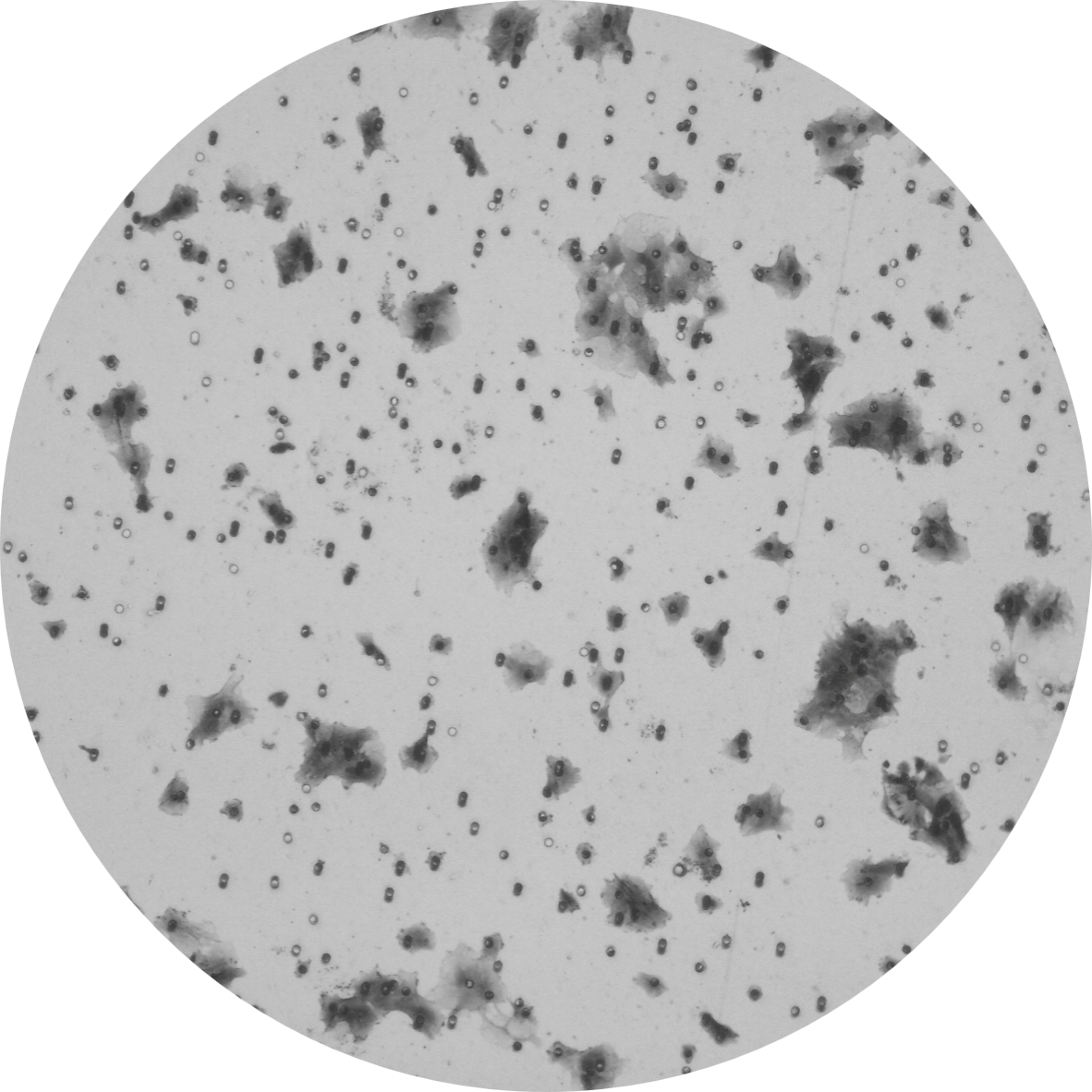

Supplement: Supplementary file 14 — Source data Fig. 6 [file 44318_2025_416_MOESM14_ESM.zip › EMBOJ-2024-119243R_SourceDataForFigure 6/6B/DLD1-LO-K176E-Invasion.tif]

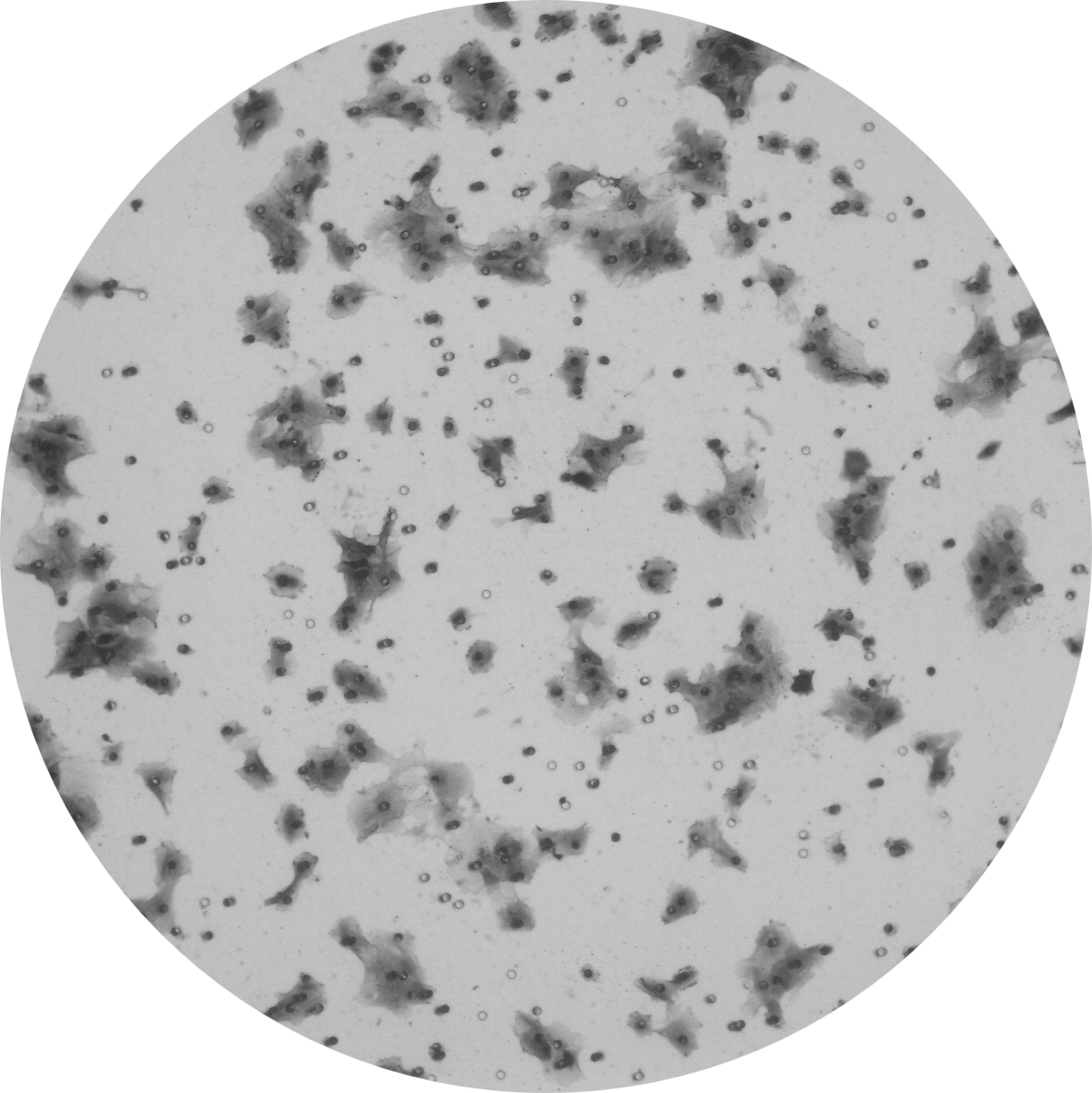

Supplement: Supplementary file 14 — Source data Fig. 6 [file 44318_2025_416_MOESM14_ESM.zip › EMBOJ-2024-119243R_SourceDataForFigure 6/6B/DLD1-LO-WT-Invasion.tif]

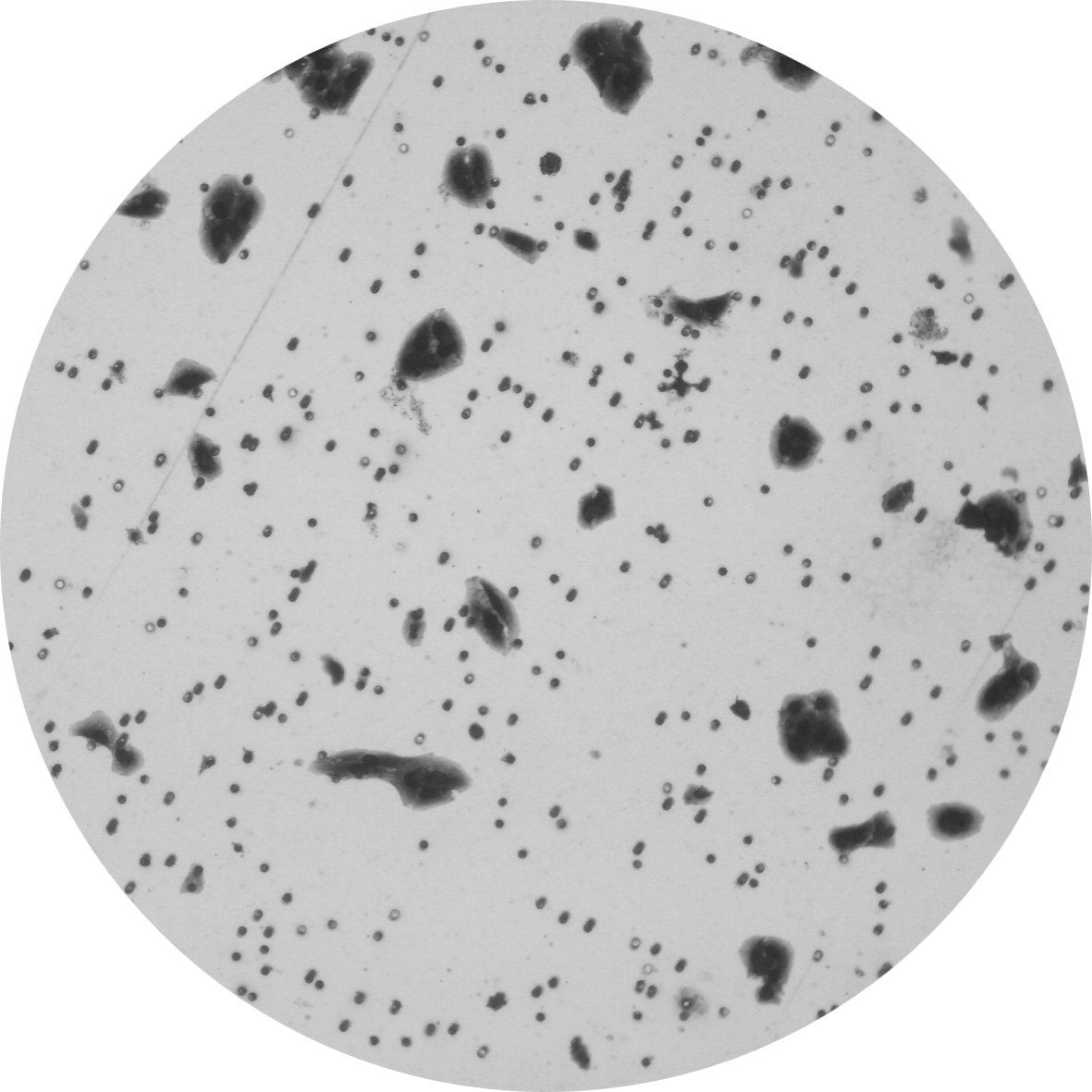

Supplement: Supplementary file 14 — Source data Fig. 6 [file 44318_2025_416_MOESM14_ESM.zip › EMBOJ-2024-119243R_SourceDataForFigure 6/6B/SW1116-HO-K176E-Invasion.tif]

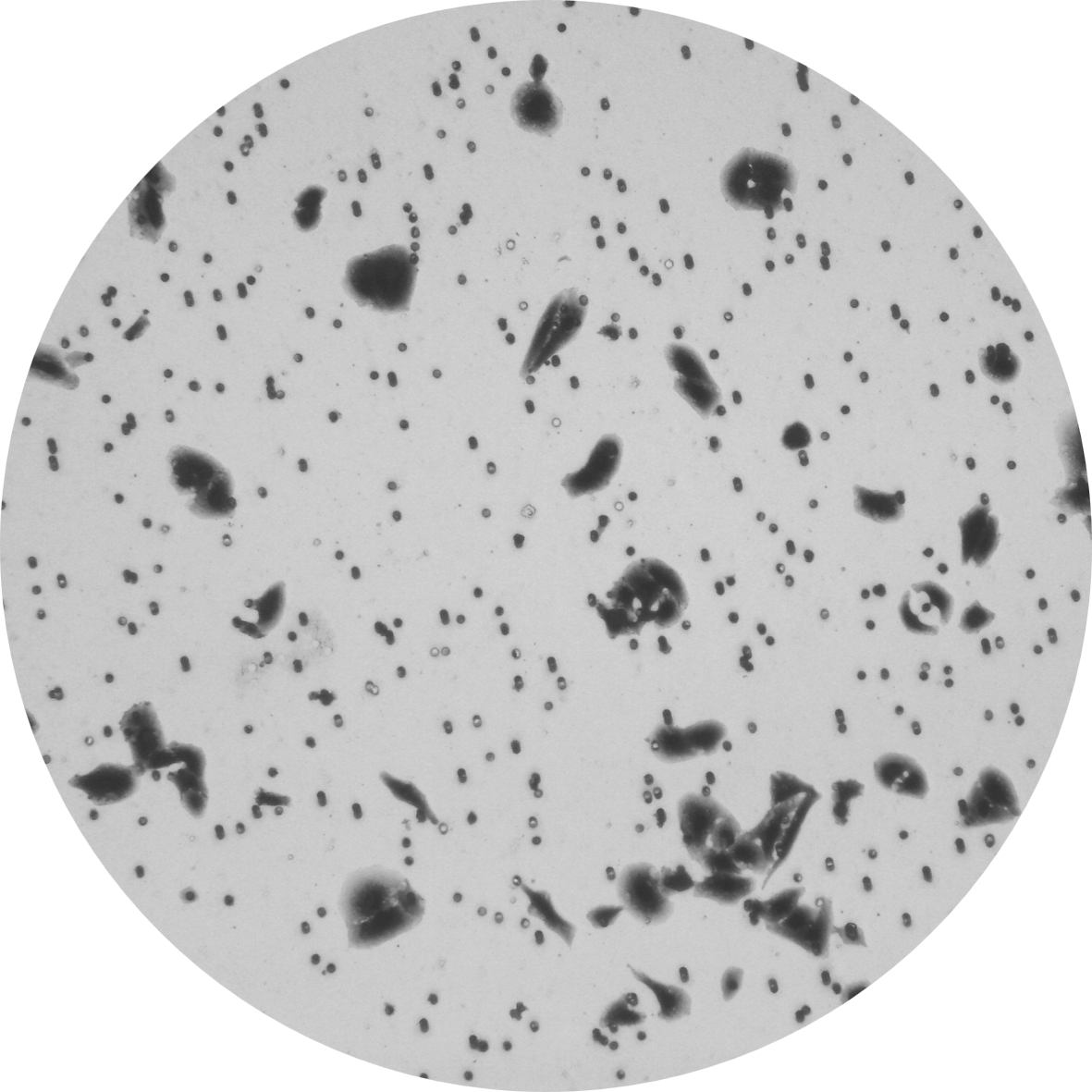

Supplement: Supplementary file 14 — Source data Fig. 6 [file 44318_2025_416_MOESM14_ESM.zip › EMBOJ-2024-119243R_SourceDataForFigure 6/6B/SW1116-HO-WT-Invasion.tif]

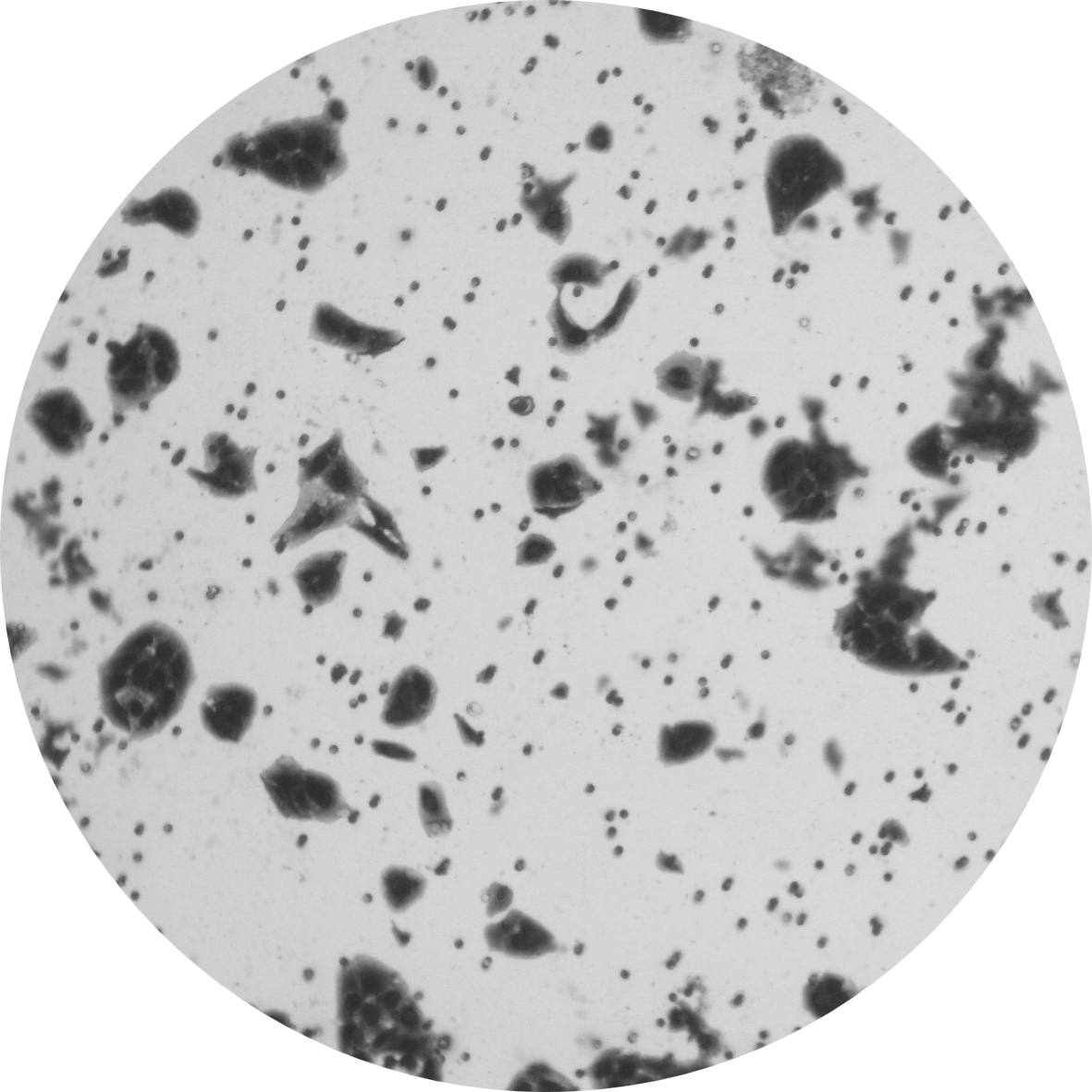

Supplement: Supplementary file 14 — Source data Fig. 6 [file 44318_2025_416_MOESM14_ESM.zip › EMBOJ-2024-119243R_SourceDataForFigure 6/6B/SW1116-LO-K176E-Invasion.tif]

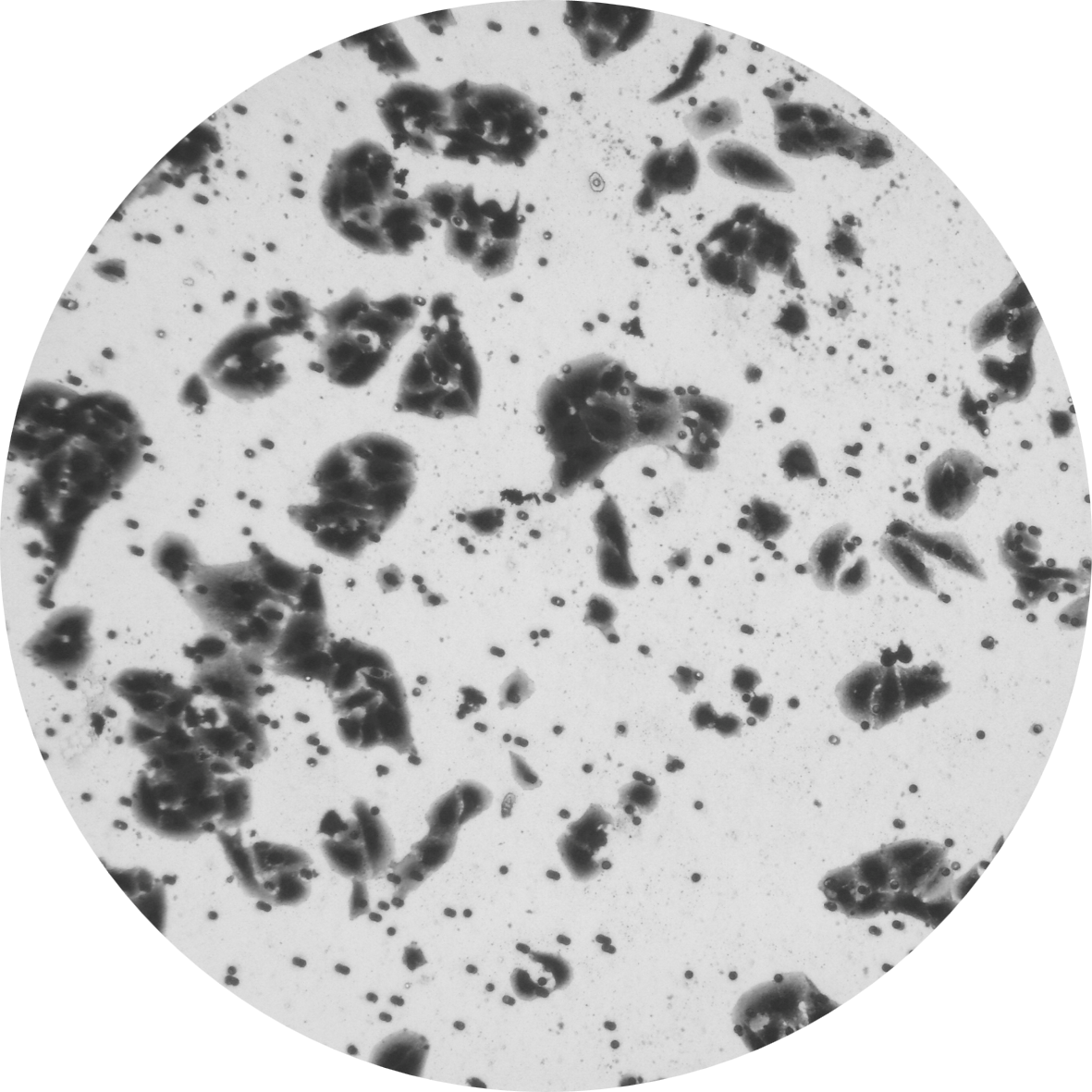

Supplement: Supplementary file 14 — Source data Fig. 6 [file 44318_2025_416_MOESM14_ESM.zip › EMBOJ-2024-119243R_SourceDataForFigure 6/6B/SW1116-LO-WT-Invasion.tif]
