# Supplementary figures and images for: SIRT5-mediated desuccinylation of PPA2 enhances HIF-1alpha-dependent adaptation to hypoxic stress and colorectal cancer metastasis (part 4 of 5)
Source: EMBO J. 2025 Mar 31;44(9):2514–40. doi: 10.1038/s44318-025-00416-1 (PMC12048626; doi:10.1038/s44318-025-00416-1)

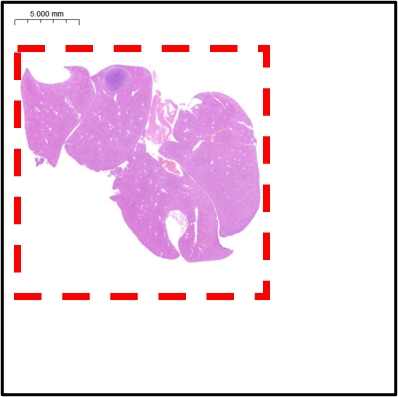

Supplement: Supplementary file 14 — Source data Fig. 6 [file 44318_2025_416_MOESM14_ESM.zip › EMBOJ-2024-119243R_SourceDataForFigure 6/6D/rK176E-HE.tif]

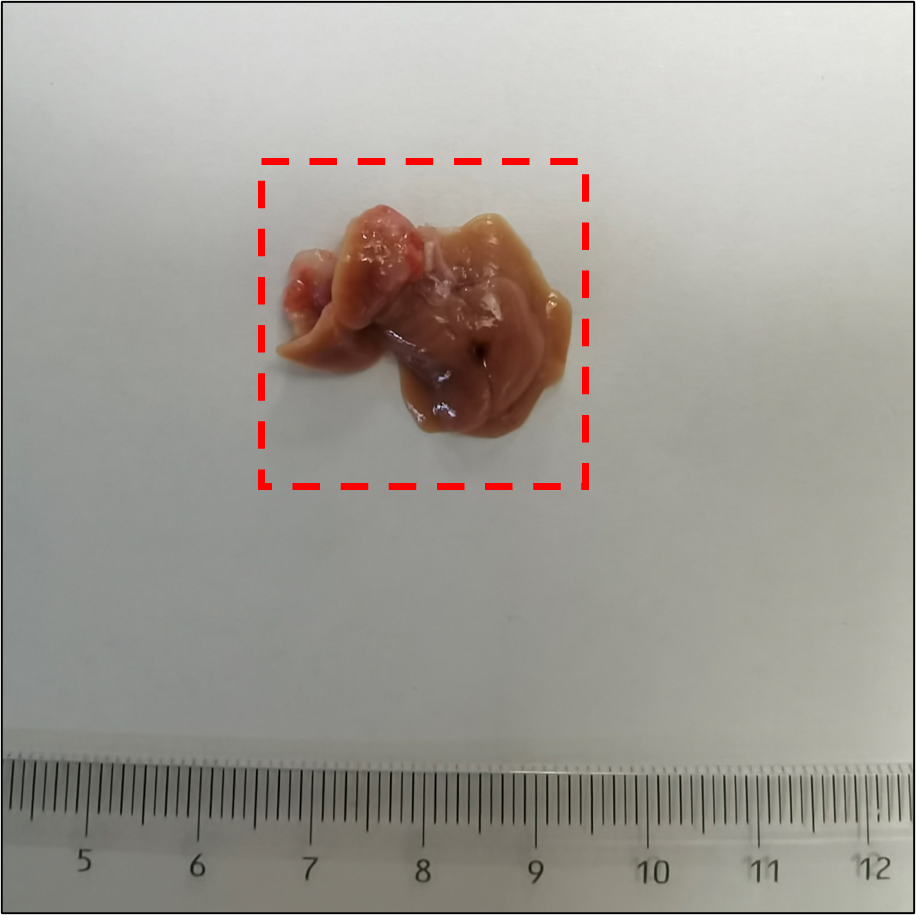

Supplement: Supplementary file 14 — Source data Fig. 6 [file 44318_2025_416_MOESM14_ESM.zip › EMBOJ-2024-119243R_SourceDataForFigure 6/6D/rK176E.tif]

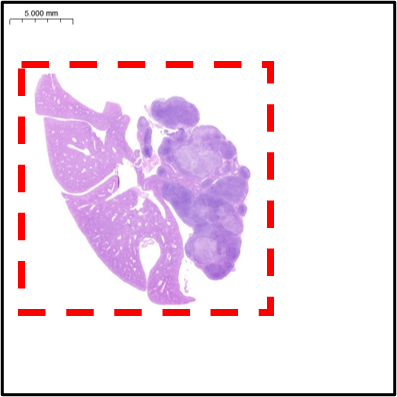

Supplement: Supplementary file 14 — Source data Fig. 6 [file 44318_2025_416_MOESM14_ESM.zip › EMBOJ-2024-119243R_SourceDataForFigure 6/6D/rWT-HE.tif]

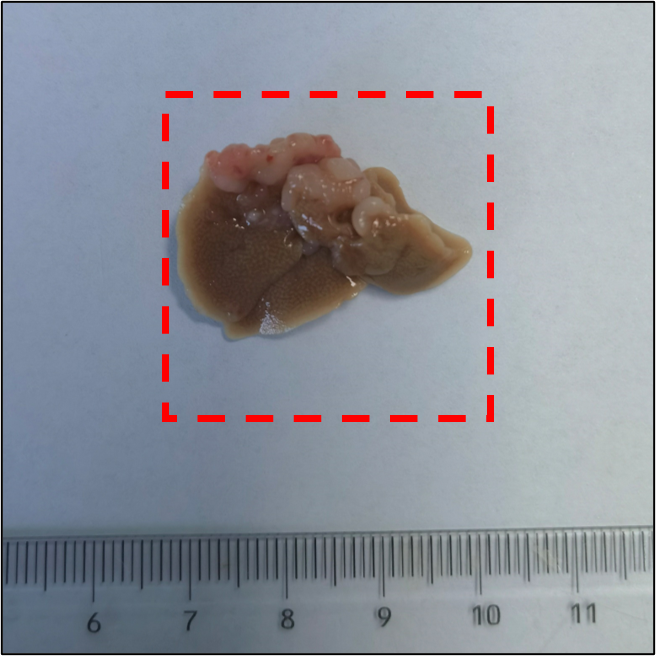

Supplement: Supplementary file 14 — Source data Fig. 6 [file 44318_2025_416_MOESM14_ESM.zip › EMBOJ-2024-119243R_SourceDataForFigure 6/6D/rWT.tif]

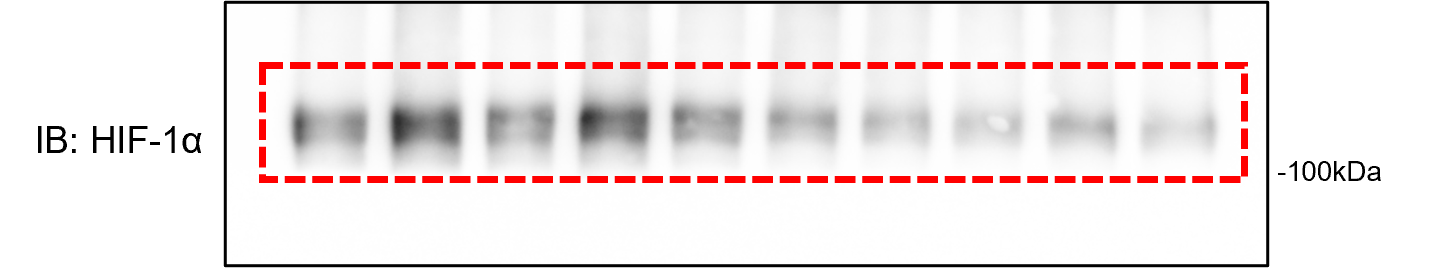

Supplement: Supplementary file 14 — Source data Fig. 6 [file 44318_2025_416_MOESM14_ESM.zip › EMBOJ-2024-119243R_SourceDataForFigure 6/6E/HIF-1α.tif]

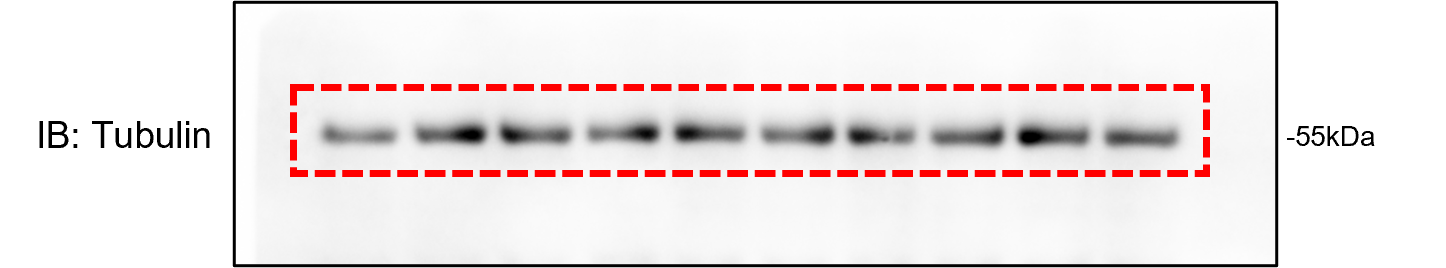

Supplement: Supplementary file 14 — Source data Fig. 6 [file 44318_2025_416_MOESM14_ESM.zip › EMBOJ-2024-119243R_SourceDataForFigure 6/6E/Tubulin.tif]

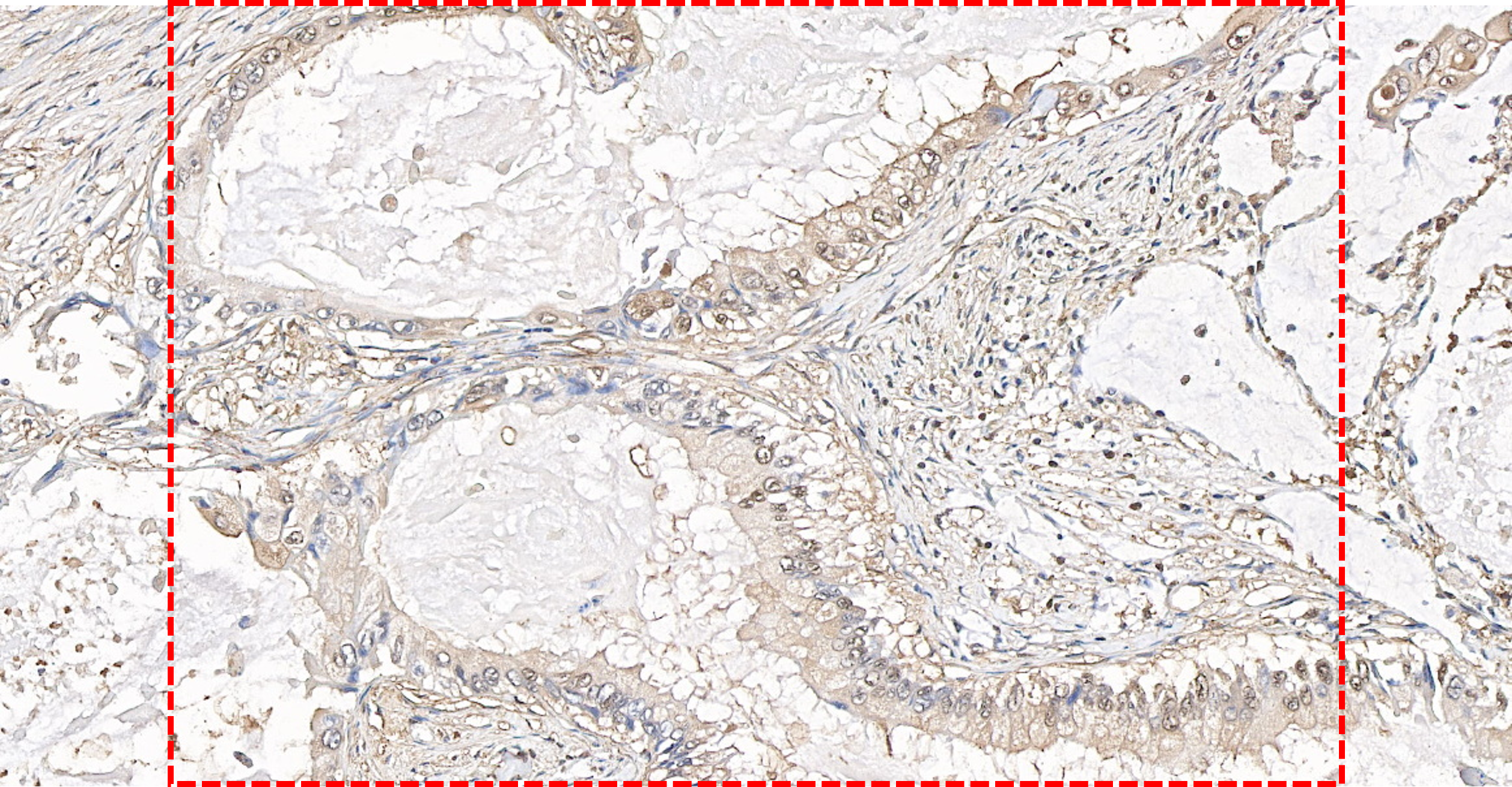

Supplement: Supplementary file 14 — Source data Fig. 6 [file 44318_2025_416_MOESM14_ESM.zip › EMBOJ-2024-119243R_SourceDataForFigure 6/6H/Patient#1-ENO1.tif]

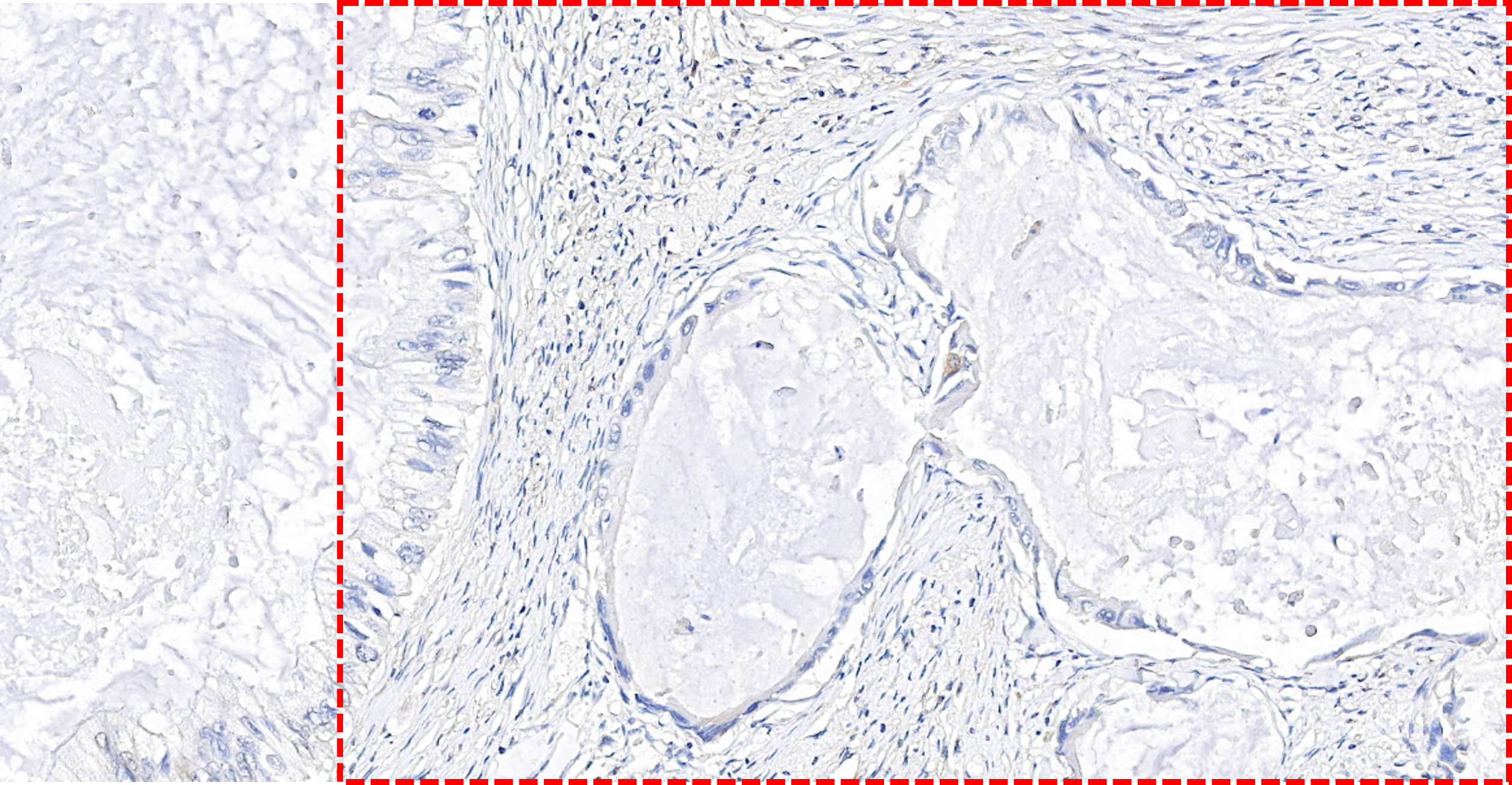

Supplement: Supplementary file 14 — Source data Fig. 6 [file 44318_2025_416_MOESM14_ESM.zip › EMBOJ-2024-119243R_SourceDataForFigure 6/6H/Patient#1-HIF-1α.tif]

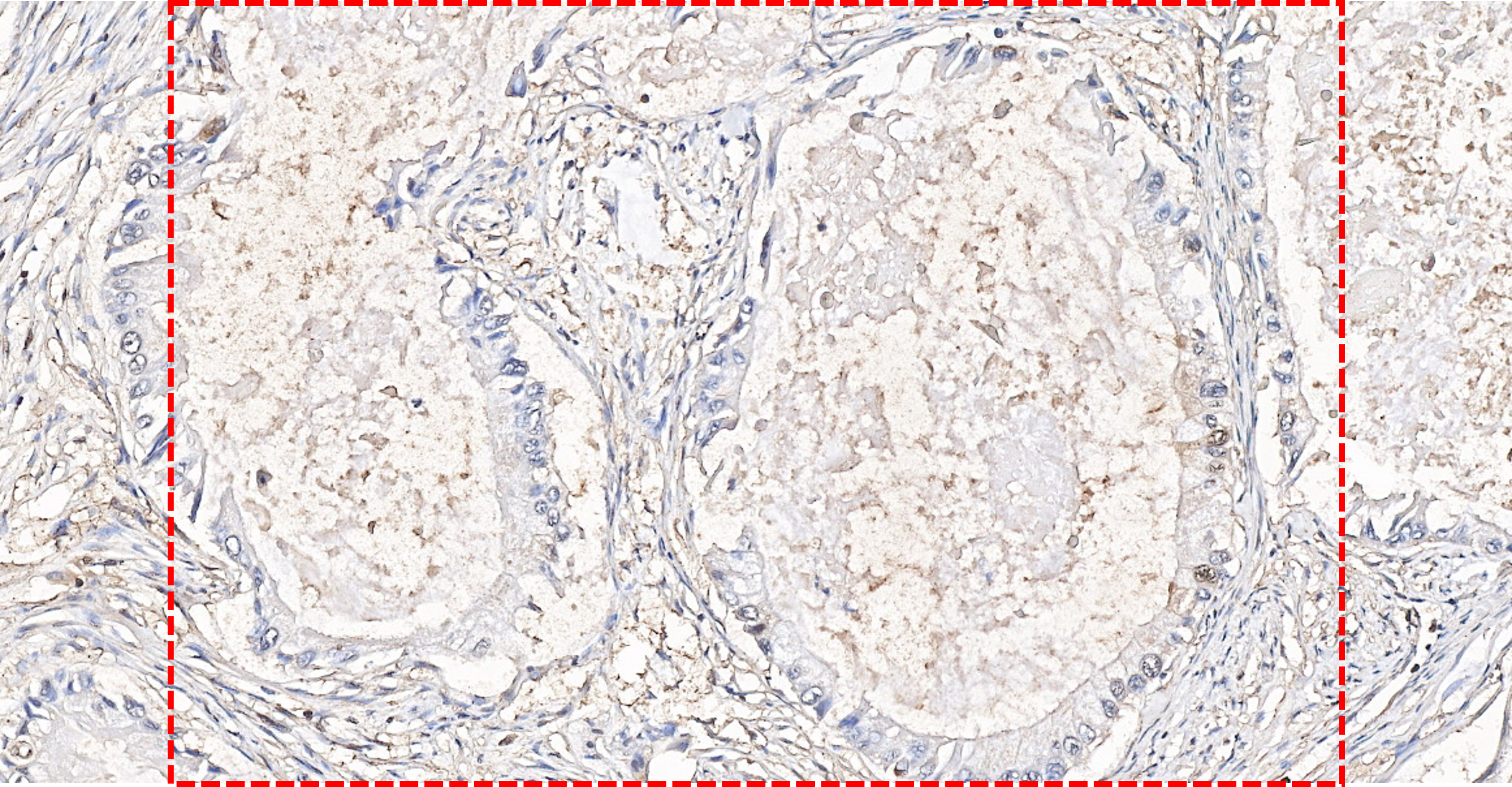

Supplement: Supplementary file 14 — Source data Fig. 6 [file 44318_2025_416_MOESM14_ESM.zip › EMBOJ-2024-119243R_SourceDataForFigure 6/6H/Patient#1-LDHA.tif]

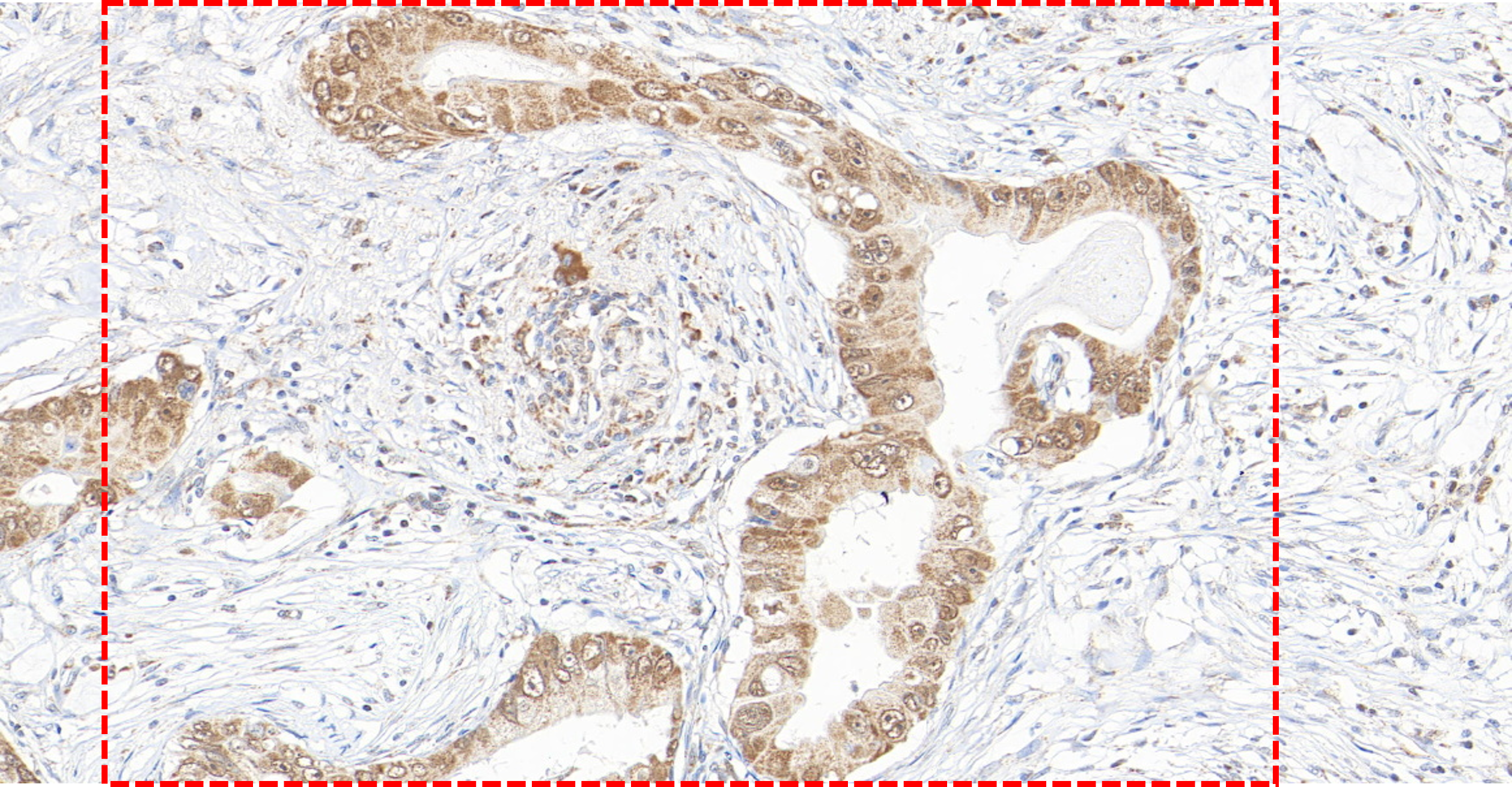

Supplement: Supplementary file 14 — Source data Fig. 6 [file 44318_2025_416_MOESM14_ESM.zip › EMBOJ-2024-119243R_SourceDataForFigure 6/6H/Patient#1-PPA2.tif]

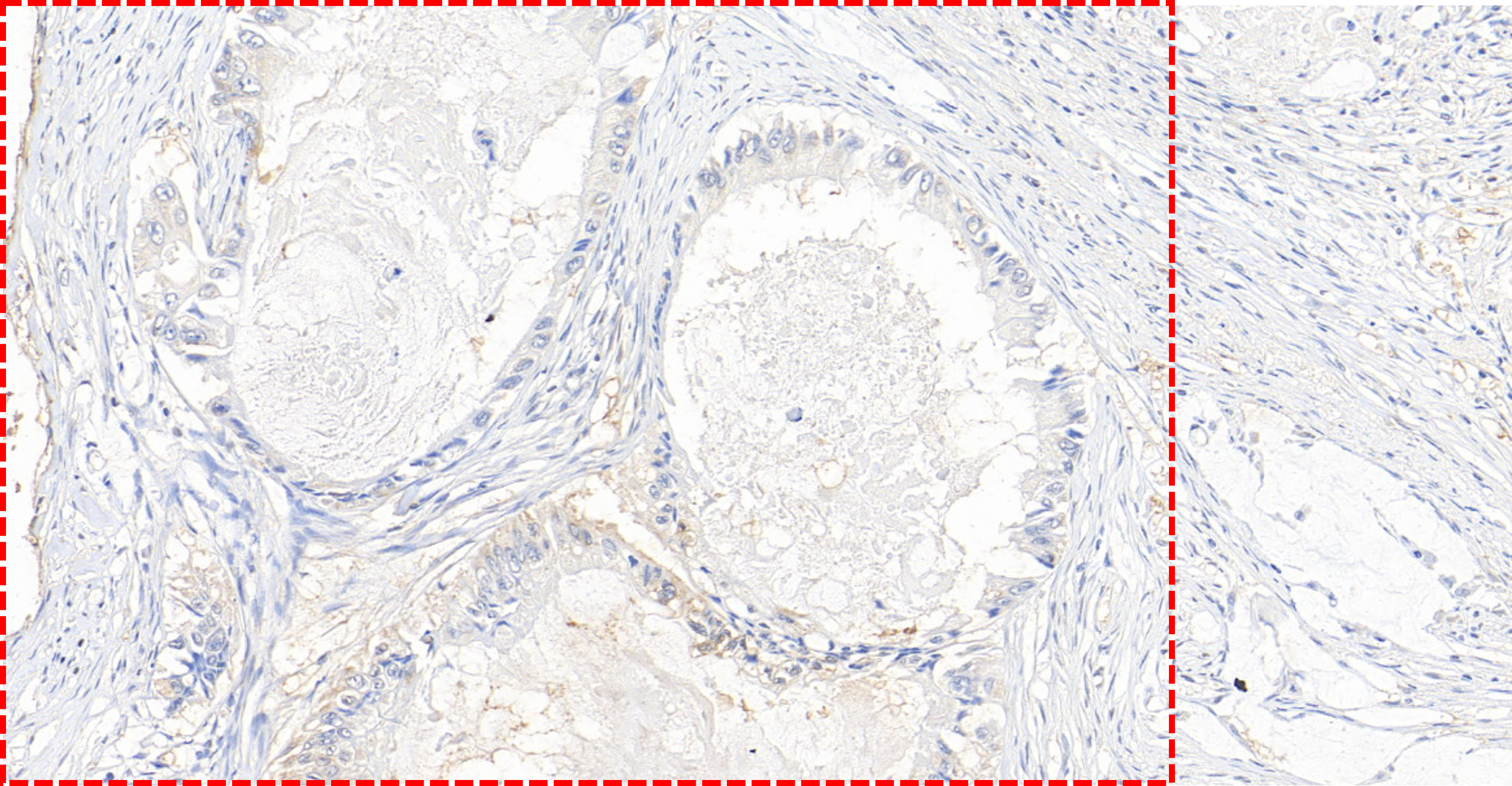

Supplement: Supplementary file 14 — Source data Fig. 6 [file 44318_2025_416_MOESM14_ESM.zip › EMBOJ-2024-119243R_SourceDataForFigure 6/6H/Patient#1-SNAI1.tif]

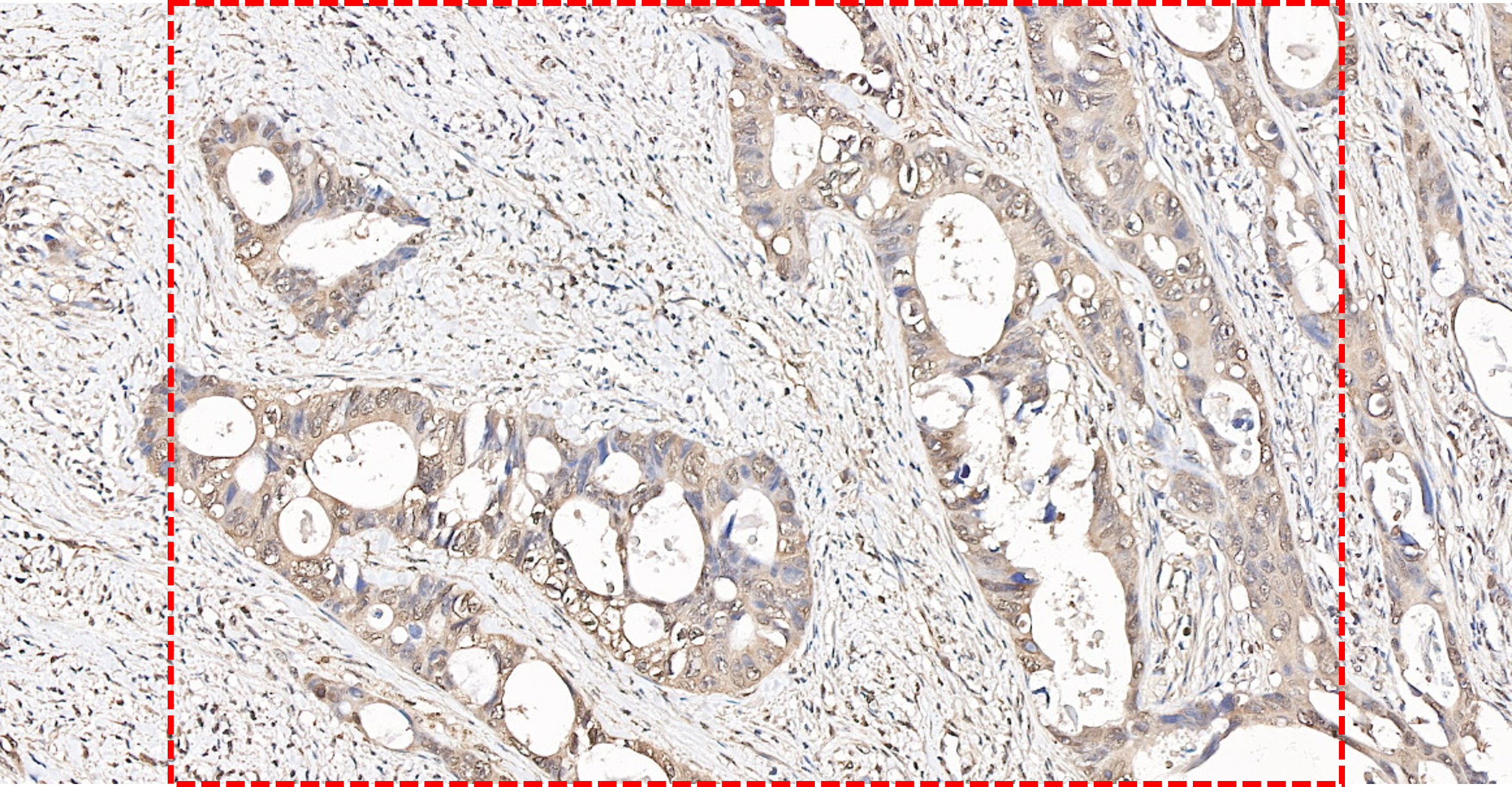

Supplement: Supplementary file 14 — Source data Fig. 6 [file 44318_2025_416_MOESM14_ESM.zip › EMBOJ-2024-119243R_SourceDataForFigure 6/6H/Patient#2-ENO1.tif]

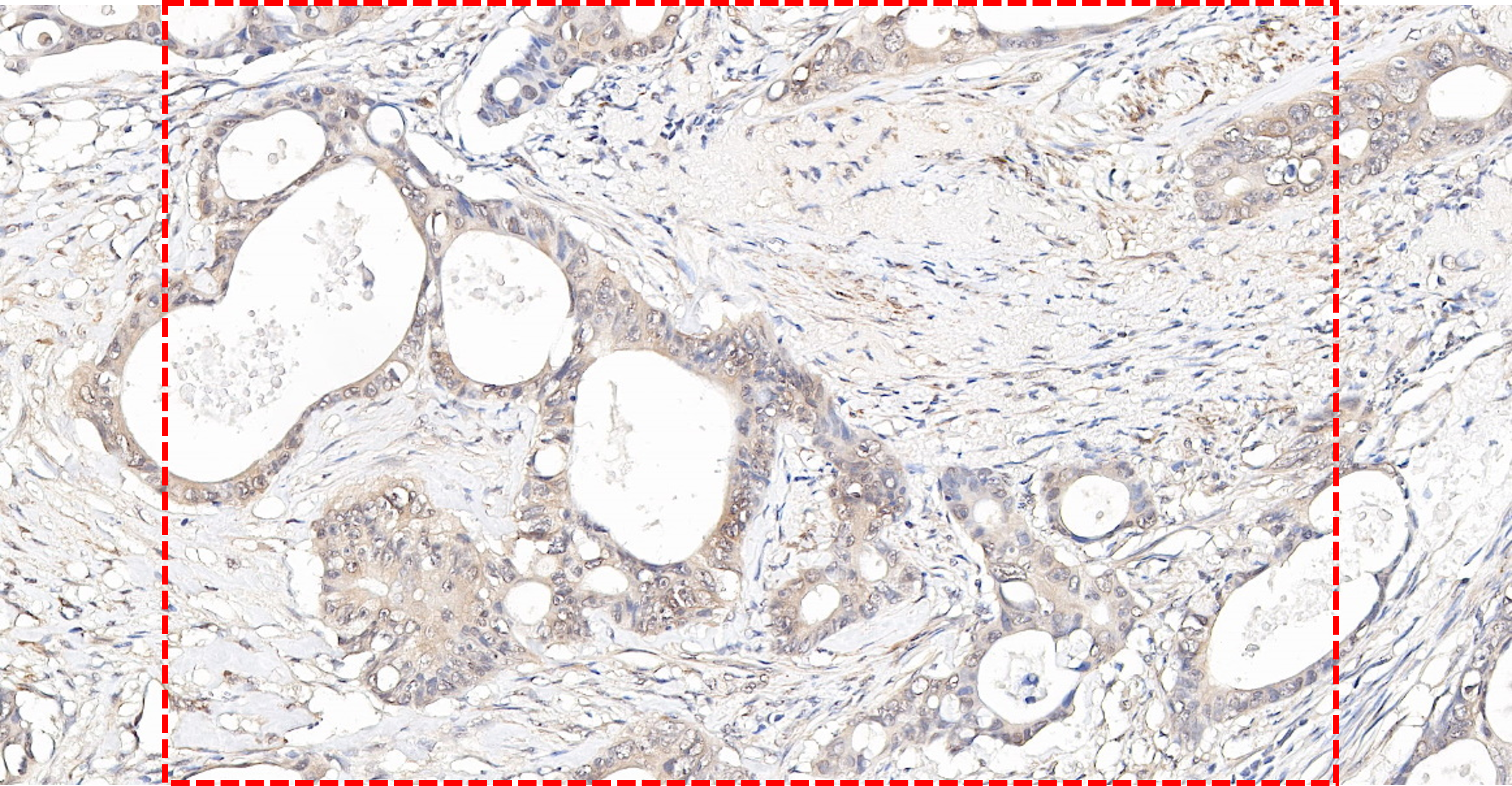

Supplement: Supplementary file 14 — Source data Fig. 6 [file 44318_2025_416_MOESM14_ESM.zip › EMBOJ-2024-119243R_SourceDataForFigure 6/6H/Patient#2-HIF-1α.tif]

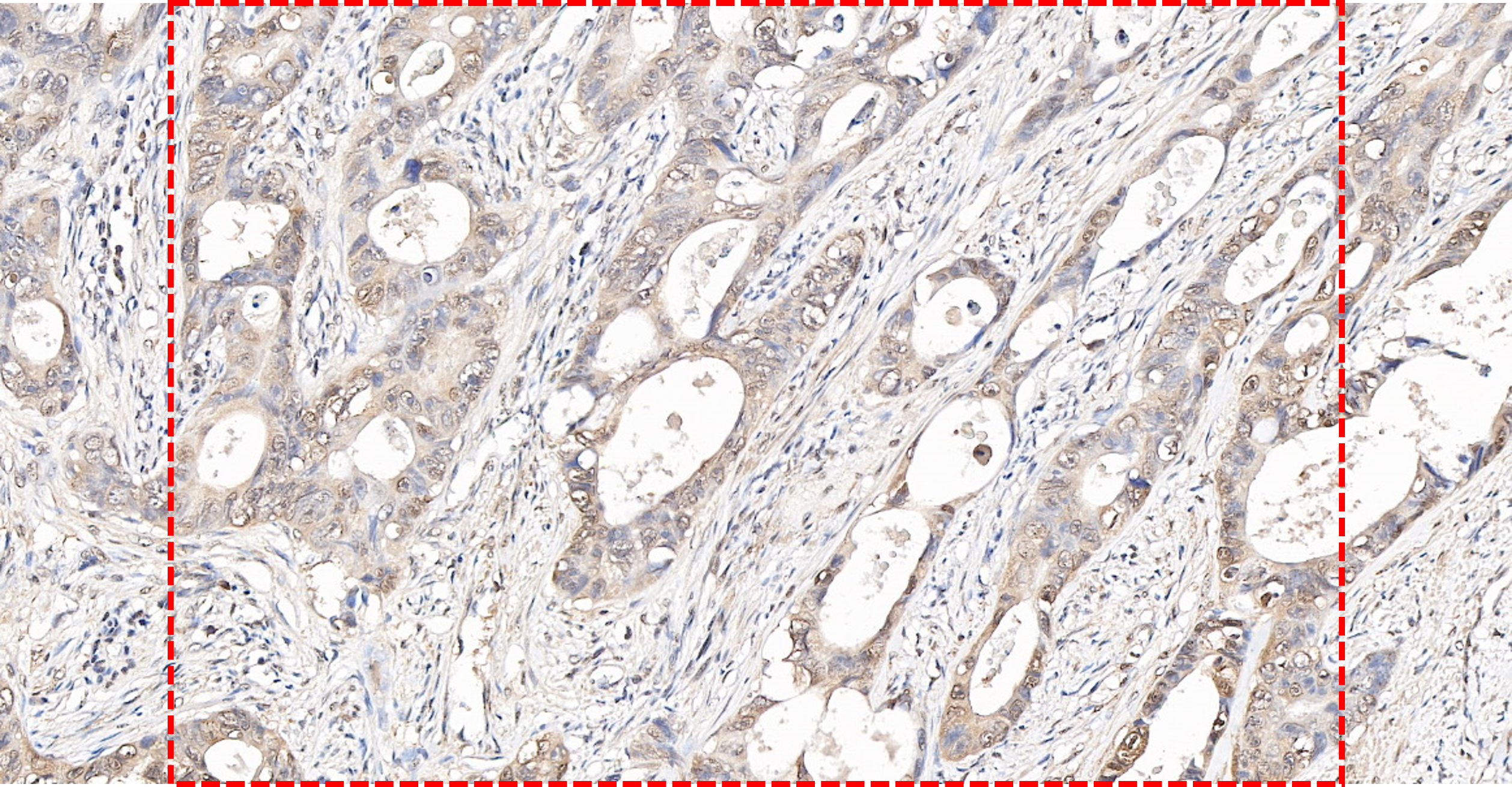

Supplement: Supplementary file 14 — Source data Fig. 6 [file 44318_2025_416_MOESM14_ESM.zip › EMBOJ-2024-119243R_SourceDataForFigure 6/6H/Patient#2-LDHA.tif]

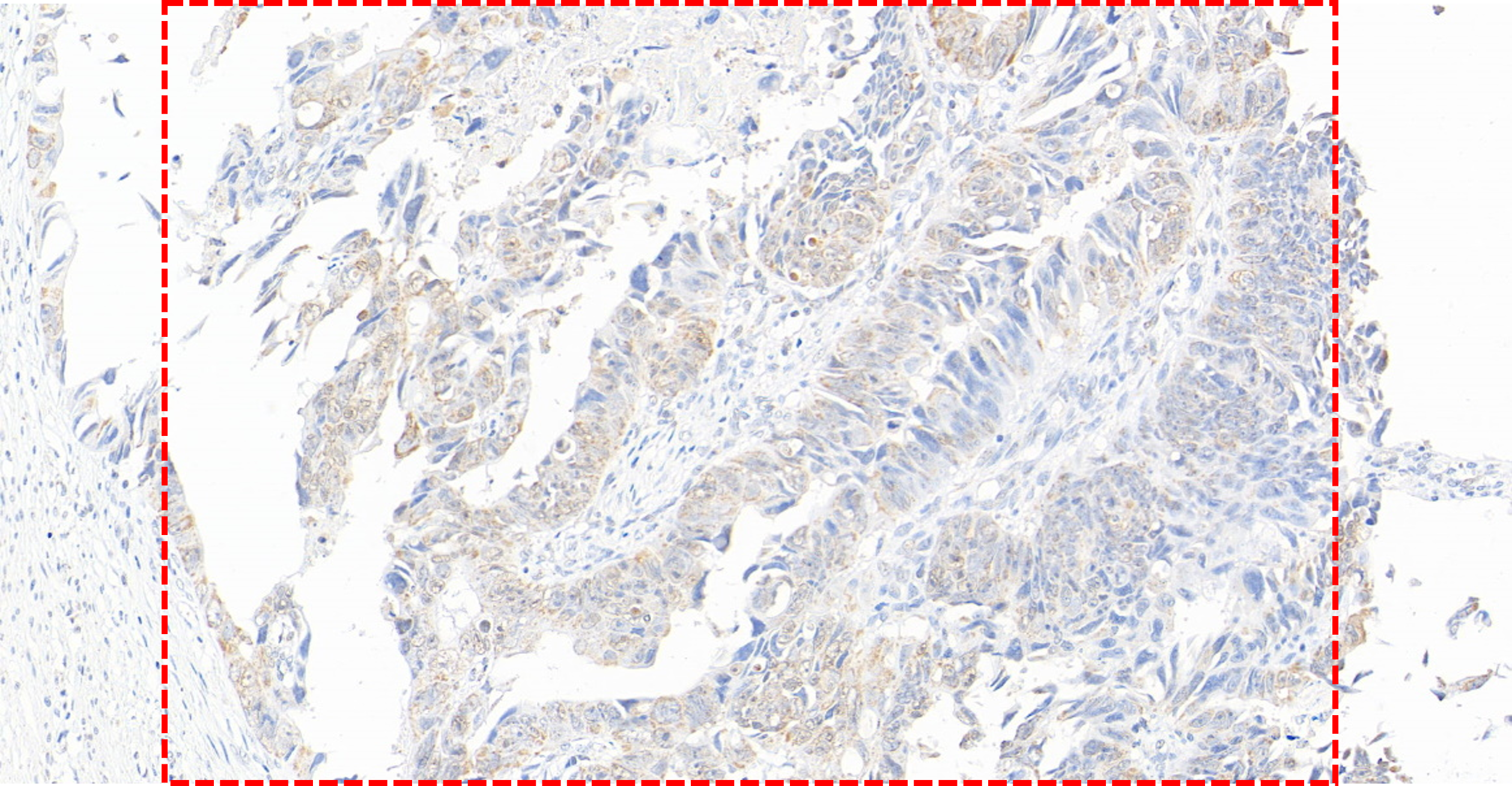

Supplement: Supplementary file 14 — Source data Fig. 6 [file 44318_2025_416_MOESM14_ESM.zip › EMBOJ-2024-119243R_SourceDataForFigure 6/6H/Patient#2-PPA2.tif]

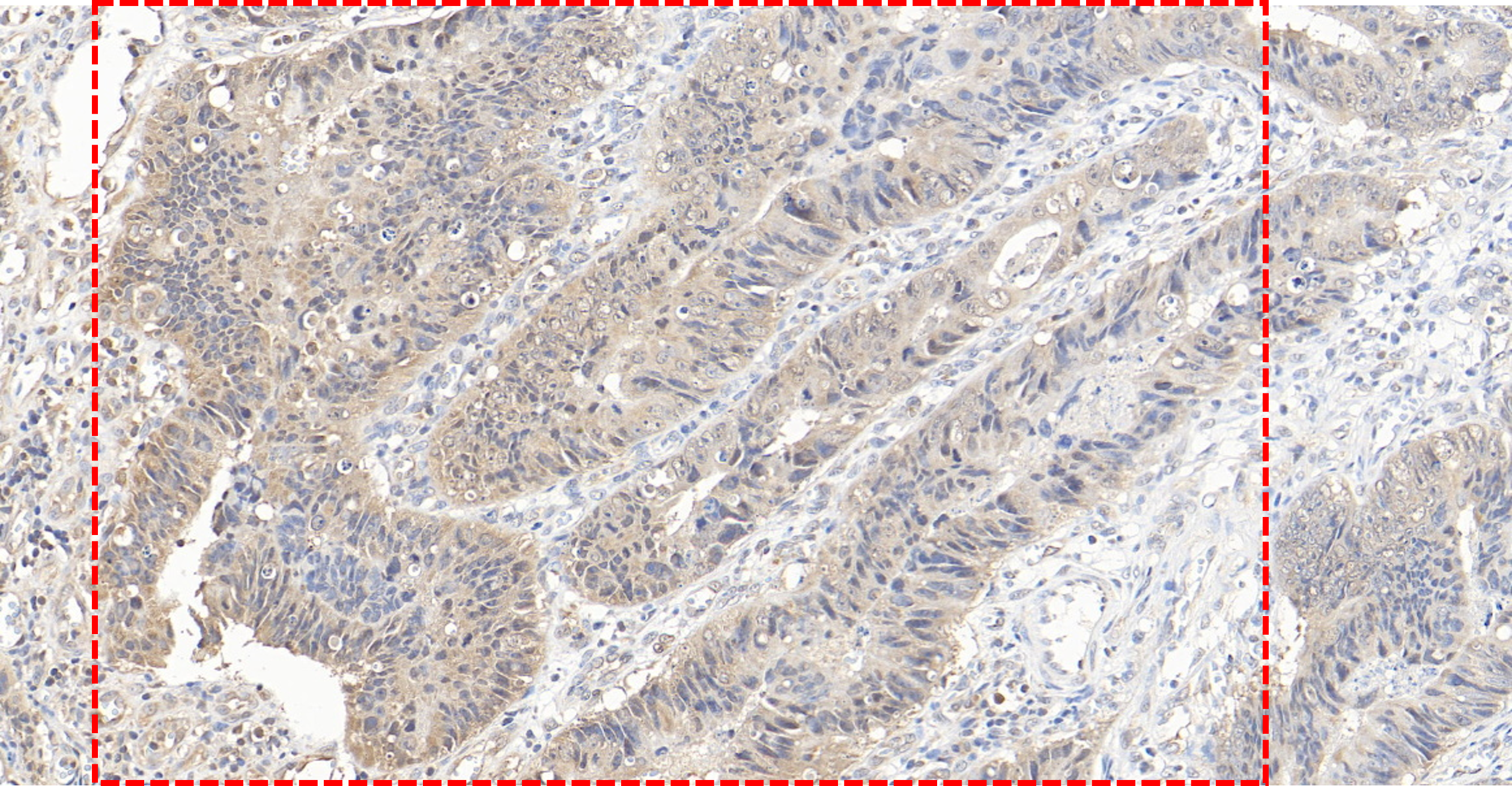

Supplement: Supplementary file 14 — Source data Fig. 6 [file 44318_2025_416_MOESM14_ESM.zip › EMBOJ-2024-119243R_SourceDataForFigure 6/6H/Patient#2-SNAI1.tif]

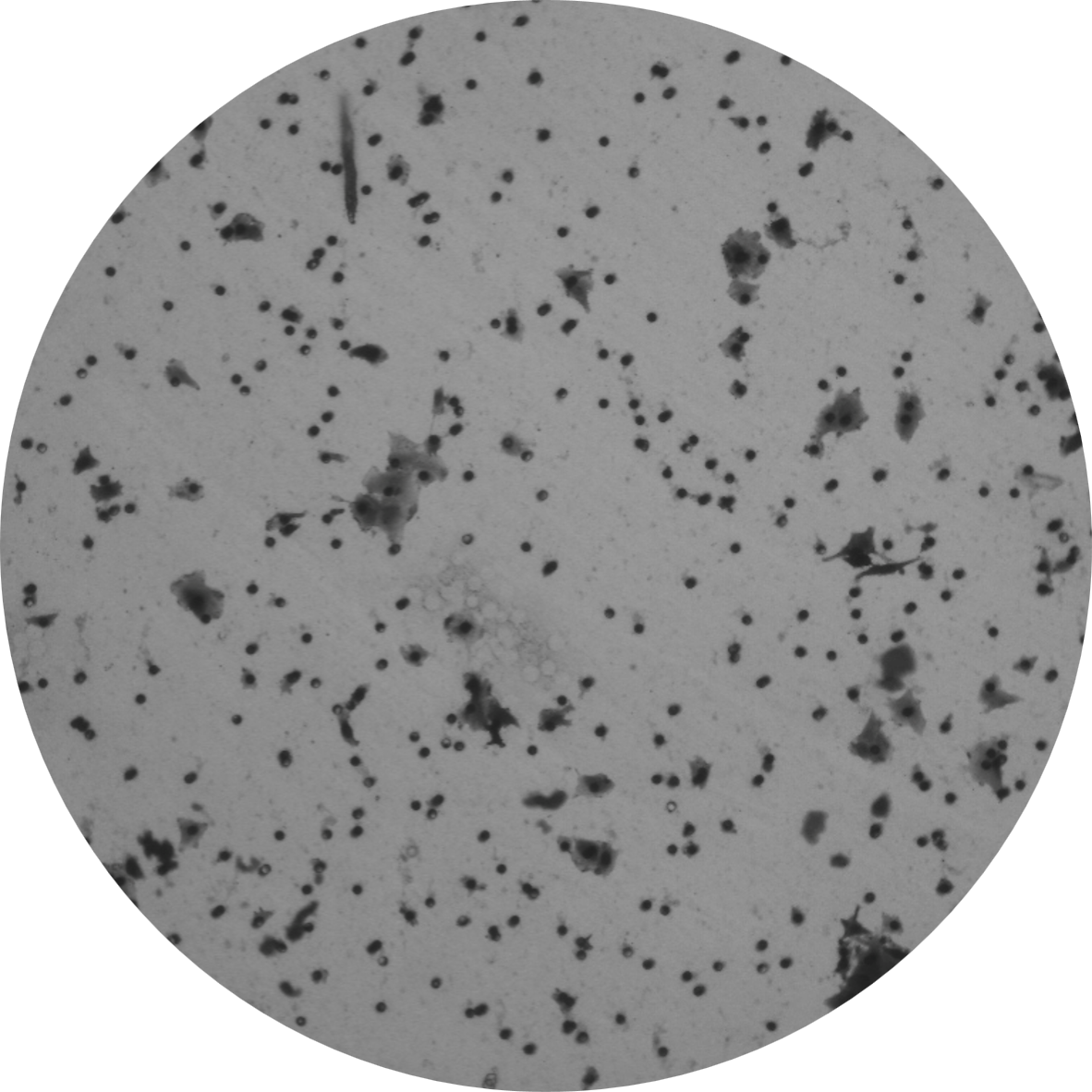

Supplement: Supplementary file 15 — Figure EV1-5 Source Data [file 44318_2025_416_MOESM15_ESM.zip › EMBOJ-2024-119243R_SourceDataForExpandedView/EMBOJ-2024-119243R_SourceDataForFigure EV1/EV1B/siDLAT.tif]

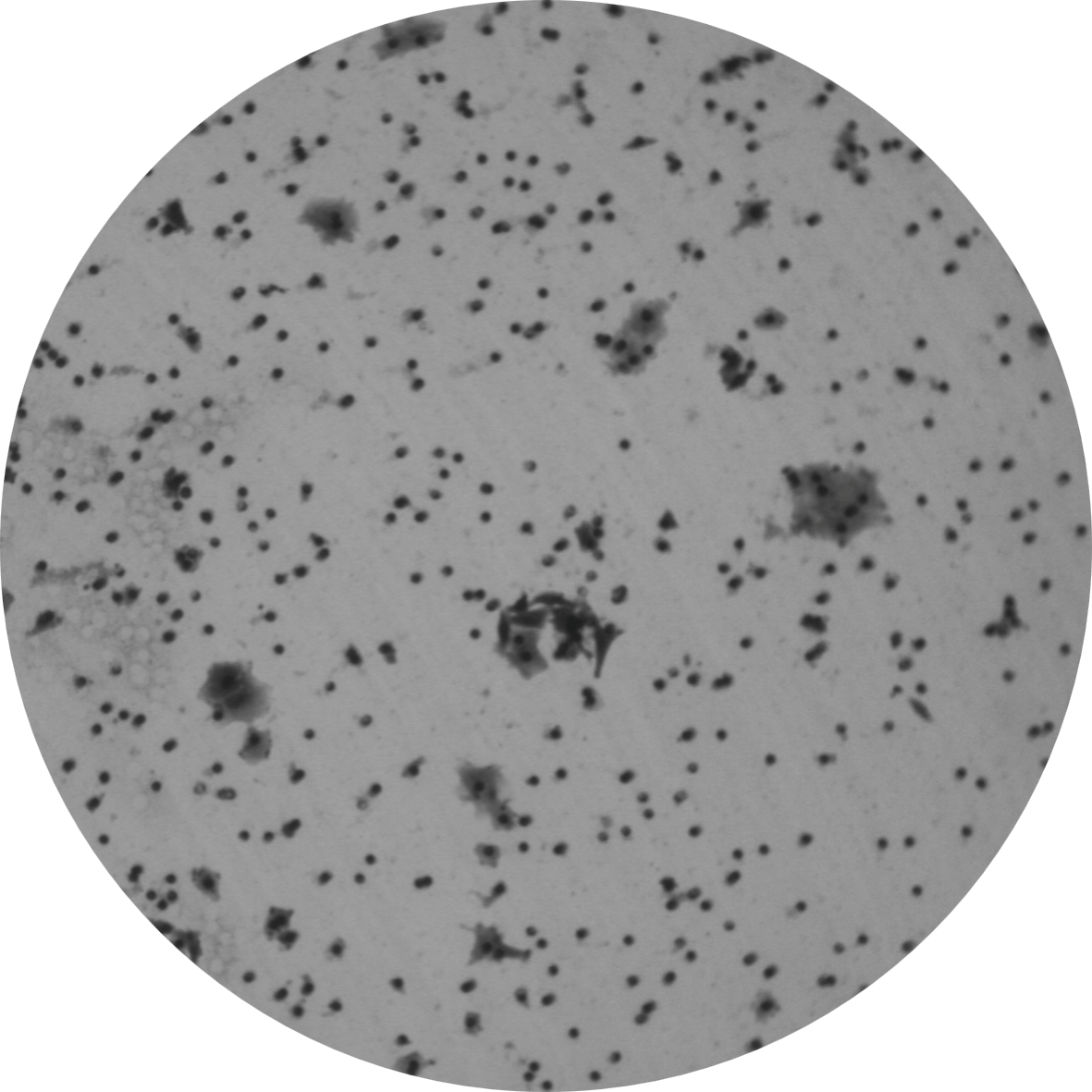

Supplement: Supplementary file 15 — Figure EV1-5 Source Data [file 44318_2025_416_MOESM15_ESM.zip › EMBOJ-2024-119243R_SourceDataForExpandedView/EMBOJ-2024-119243R_SourceDataForFigure EV1/EV1B/siELOVL6.tif]

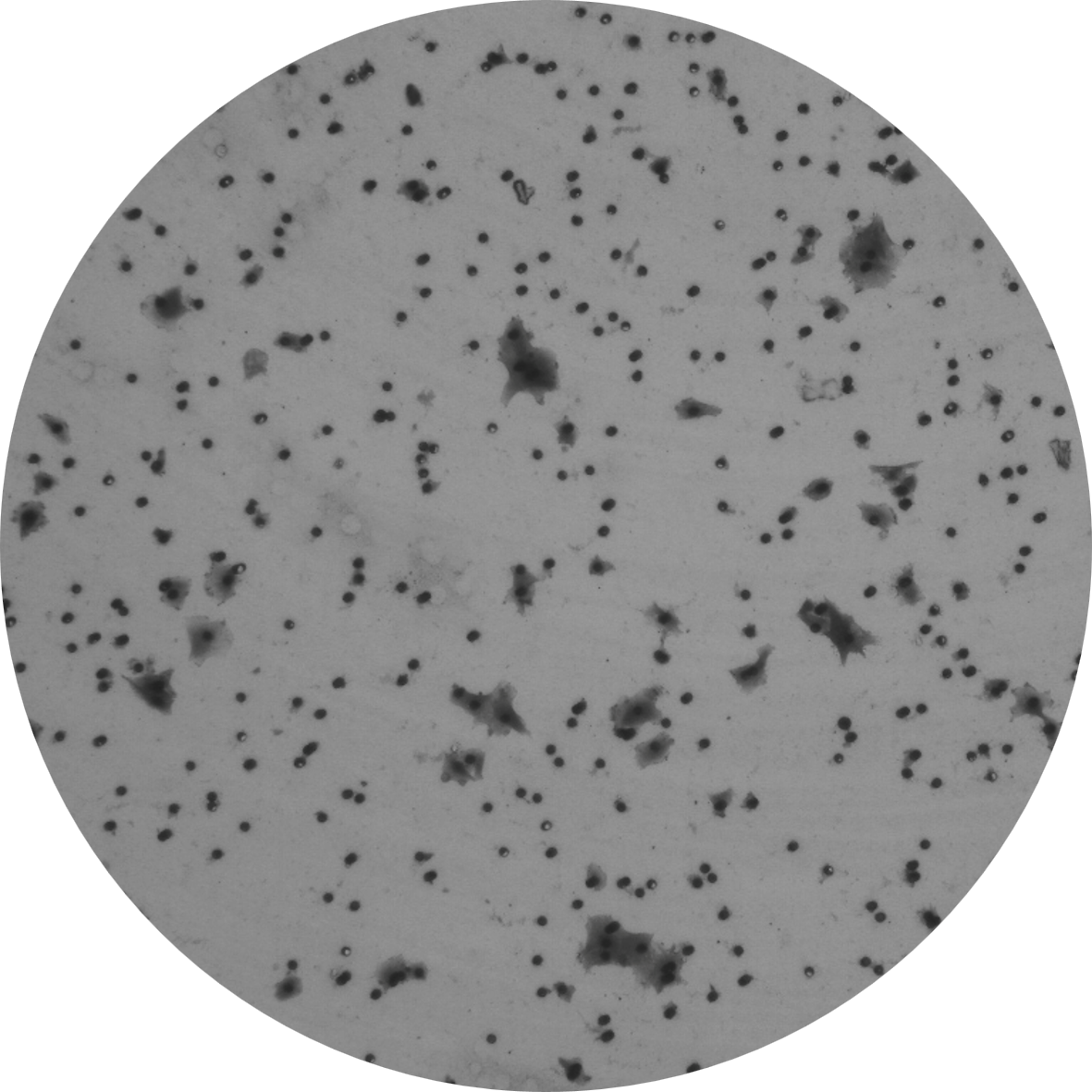

Supplement: Supplementary file 15 — Figure EV1-5 Source Data [file 44318_2025_416_MOESM15_ESM.zip › EMBOJ-2024-119243R_SourceDataForExpandedView/EMBOJ-2024-119243R_SourceDataForFigure EV1/EV1B/siNC.tif]

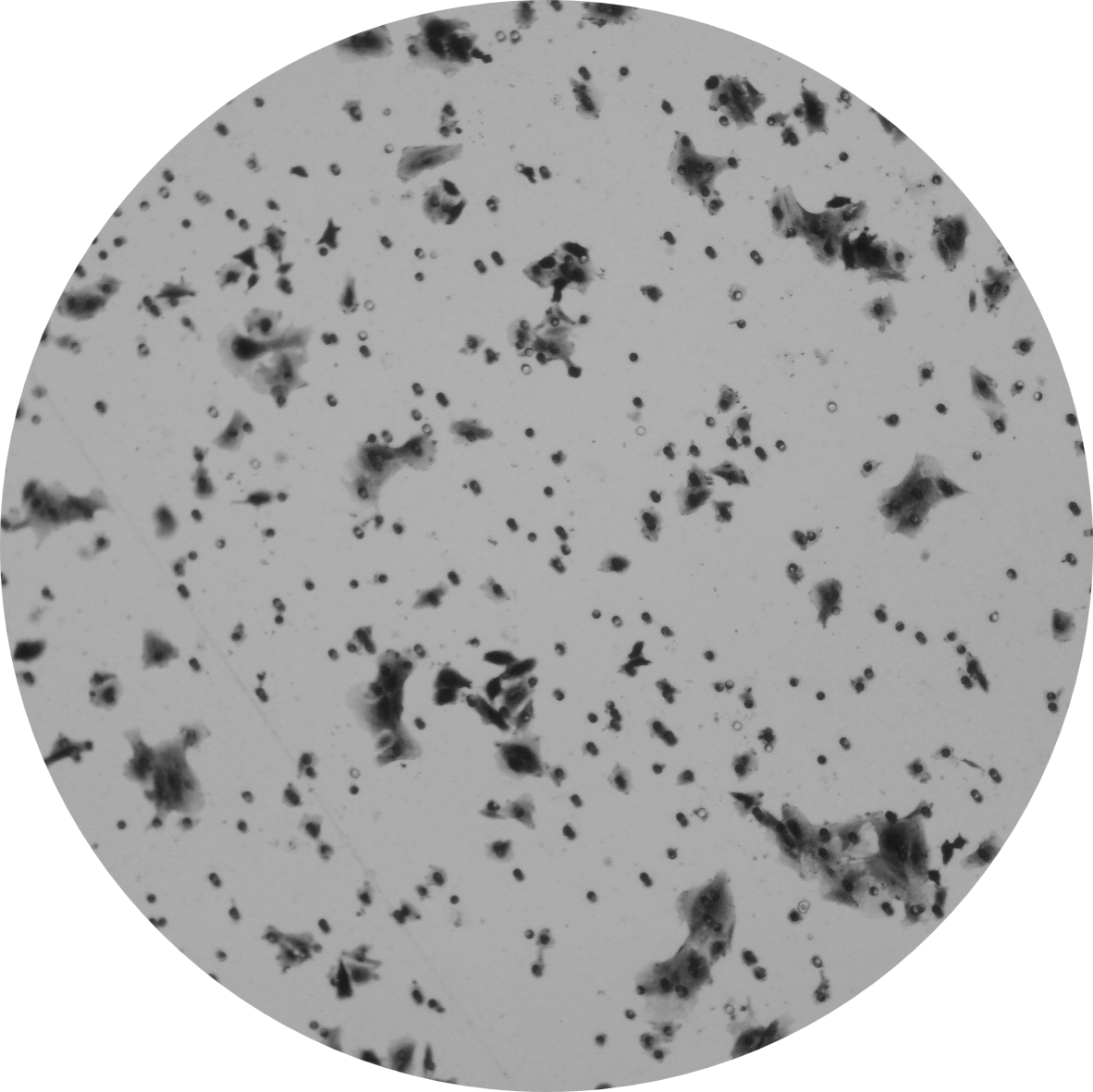

Supplement: Supplementary file 15 — Figure EV1-5 Source Data [file 44318_2025_416_MOESM15_ESM.zip › EMBOJ-2024-119243R_SourceDataForExpandedView/EMBOJ-2024-119243R_SourceDataForFigure EV1/EV1B/siPPA2.tif]

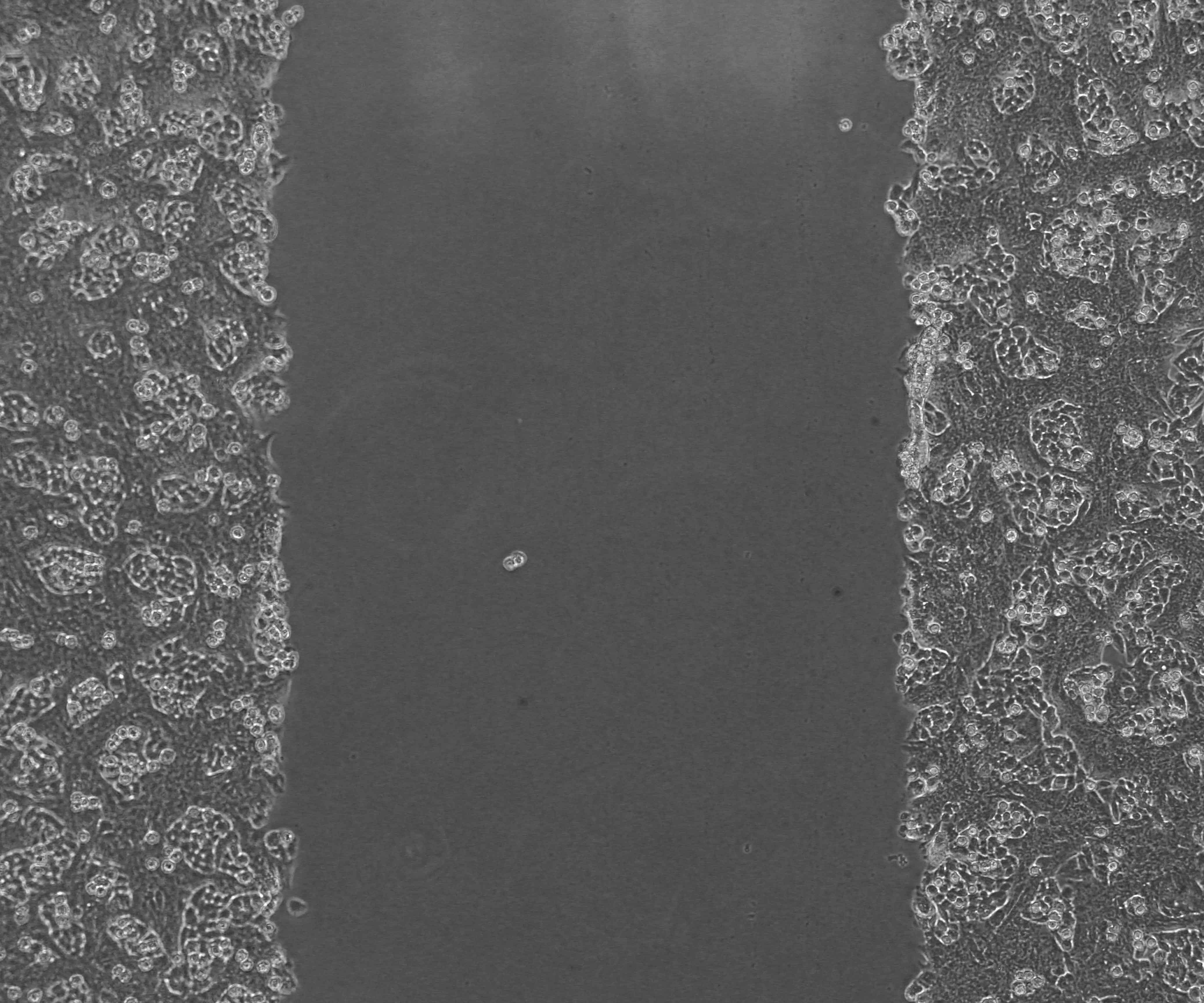

Supplement: Supplementary file 15 — Figure EV1-5 Source Data [file 44318_2025_416_MOESM15_ESM.zip › EMBOJ-2024-119243R_SourceDataForExpandedView/EMBOJ-2024-119243R_SourceDataForFigure EV2/EV2B/DLD1-0h-HO-shNT.jpg]

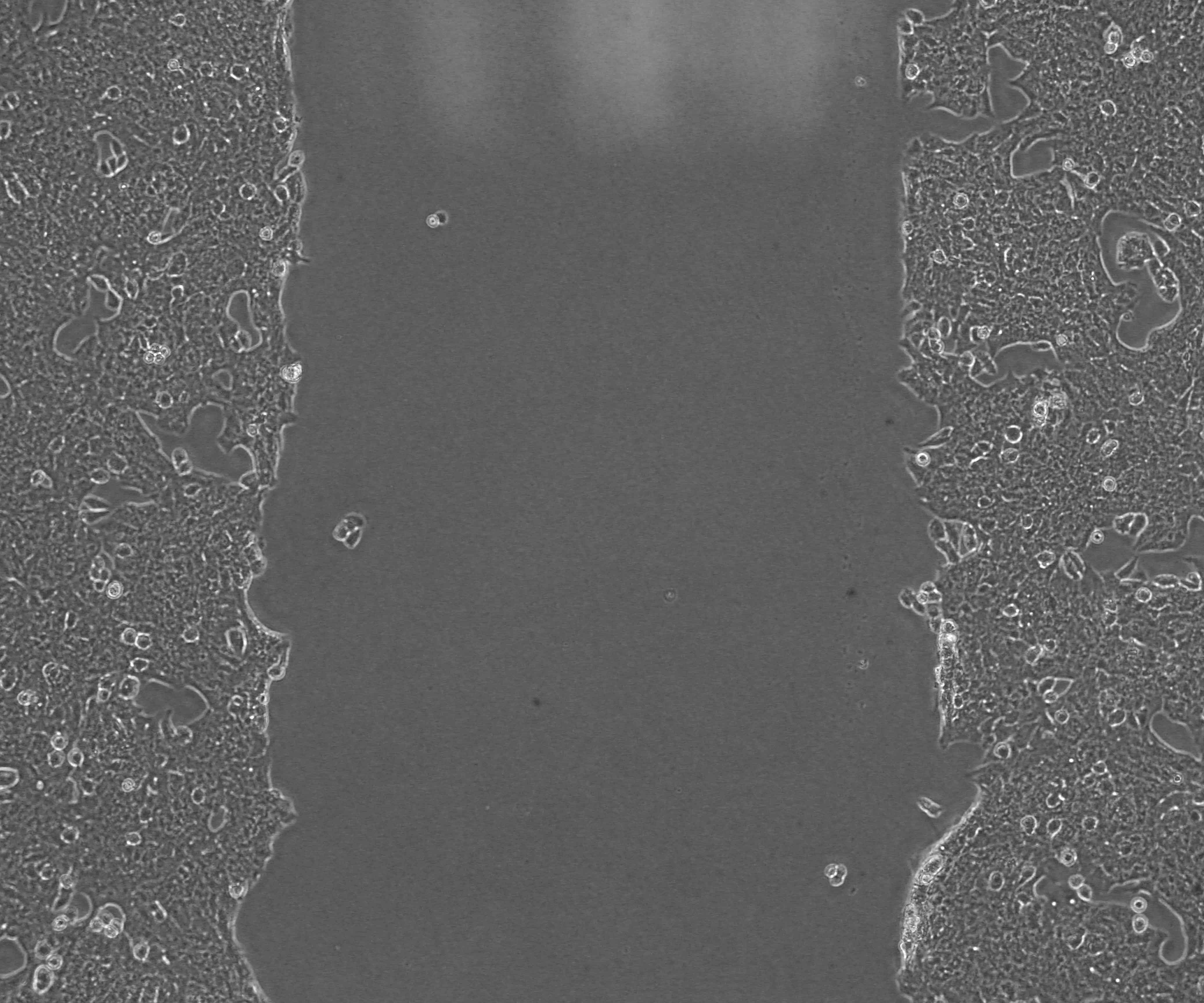

Supplement: Supplementary file 15 — Figure EV1-5 Source Data [file 44318_2025_416_MOESM15_ESM.zip › EMBOJ-2024-119243R_SourceDataForExpandedView/EMBOJ-2024-119243R_SourceDataForFigure EV2/EV2B/DLD1-0h-HO-shPPA2#1.jpg]

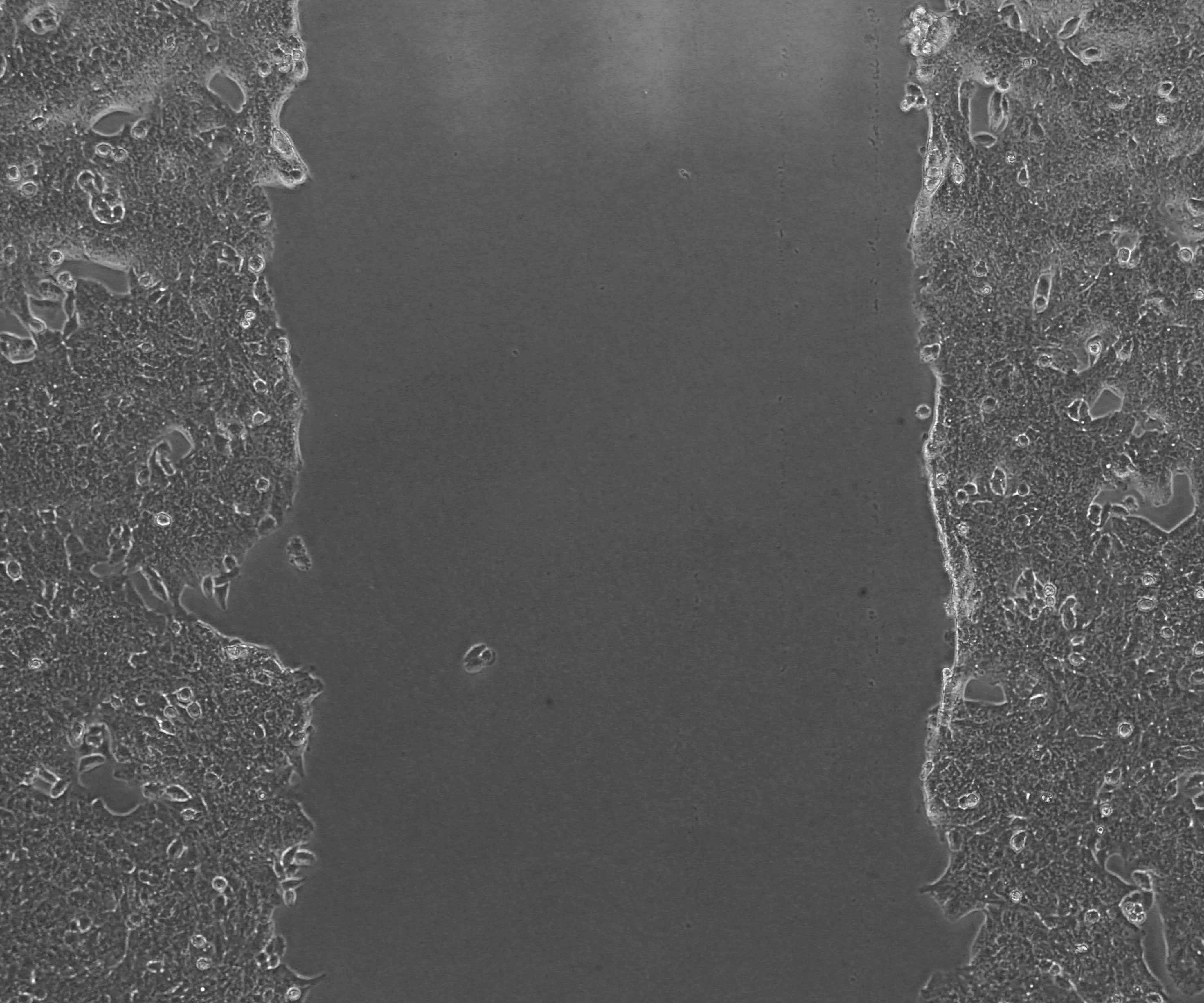

Supplement: Supplementary file 15 — Figure EV1-5 Source Data [file 44318_2025_416_MOESM15_ESM.zip › EMBOJ-2024-119243R_SourceDataForExpandedView/EMBOJ-2024-119243R_SourceDataForFigure EV2/EV2B/DLD1-0h-HO-shPPA2#2.jpg]

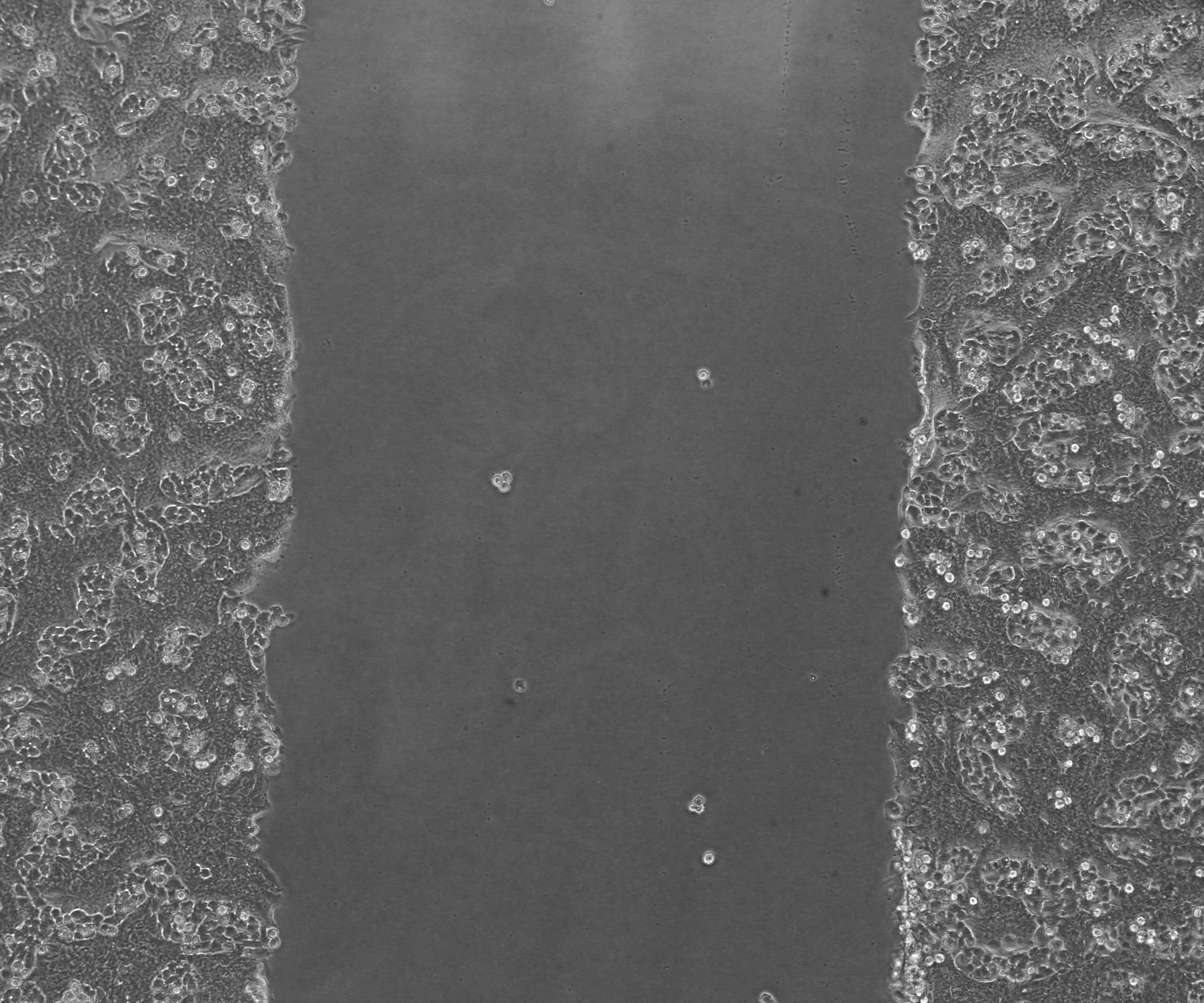

Supplement: Supplementary file 15 — Figure EV1-5 Source Data [file 44318_2025_416_MOESM15_ESM.zip › EMBOJ-2024-119243R_SourceDataForExpandedView/EMBOJ-2024-119243R_SourceDataForFigure EV2/EV2B/DLD1-0h-LO-shNT.jpg]

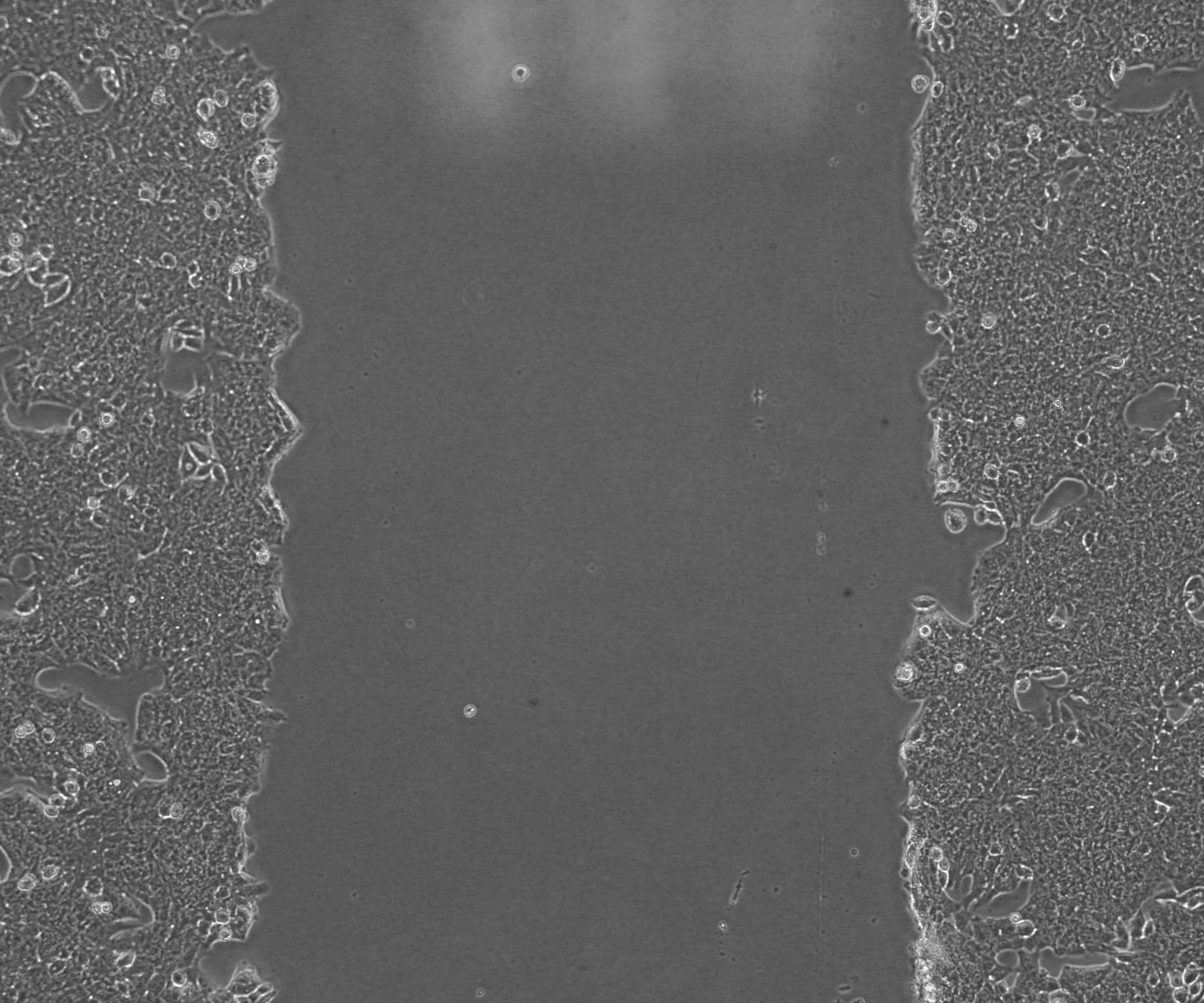

Supplement: Supplementary file 15 — Figure EV1-5 Source Data [file 44318_2025_416_MOESM15_ESM.zip › EMBOJ-2024-119243R_SourceDataForExpandedView/EMBOJ-2024-119243R_SourceDataForFigure EV2/EV2B/DLD1-0h-LO-shPPA2#1.jpg]

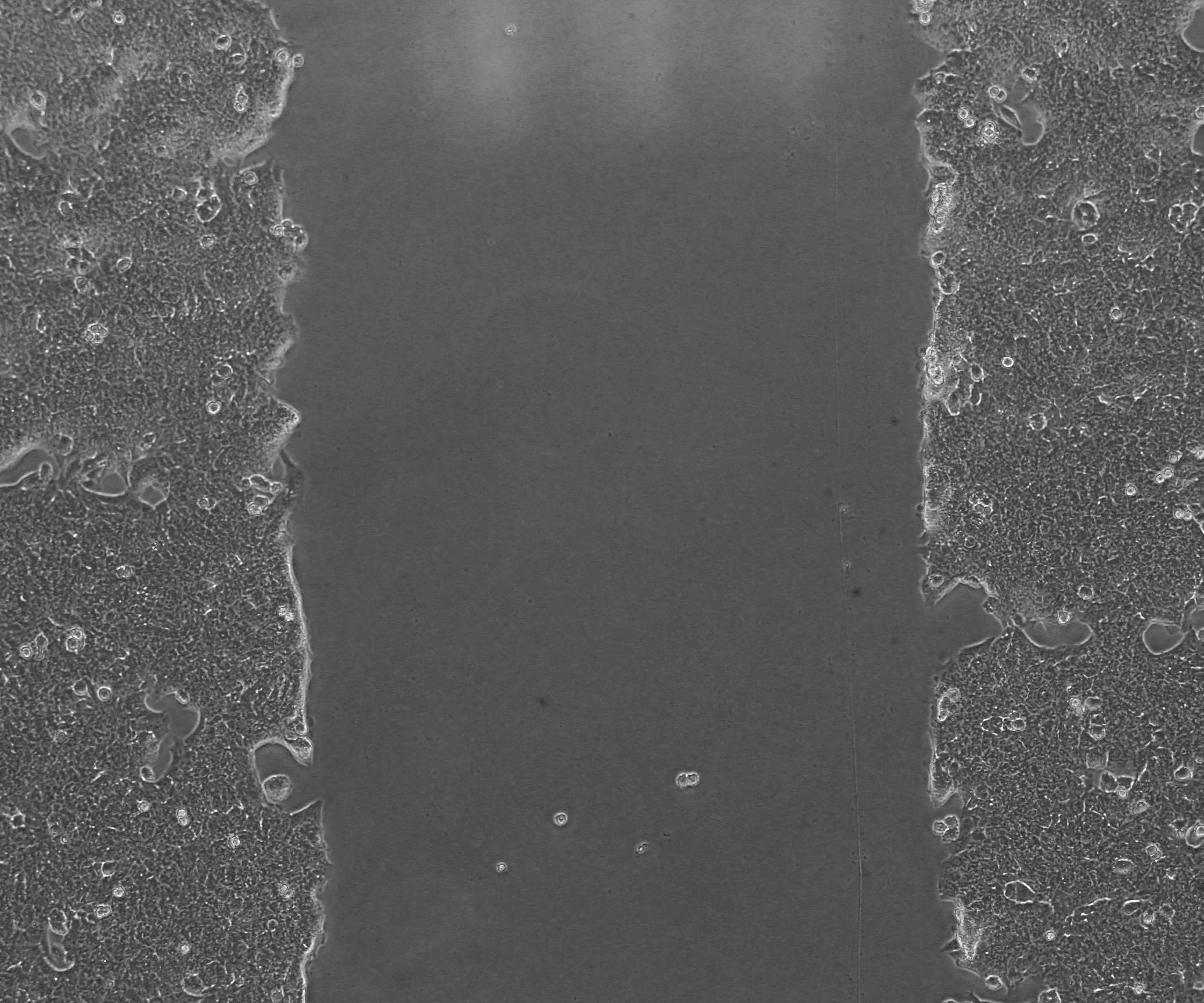

Supplement: Supplementary file 15 — Figure EV1-5 Source Data [file 44318_2025_416_MOESM15_ESM.zip › EMBOJ-2024-119243R_SourceDataForExpandedView/EMBOJ-2024-119243R_SourceDataForFigure EV2/EV2B/DLD1-0h-LO-shPPA2#2.jpg]

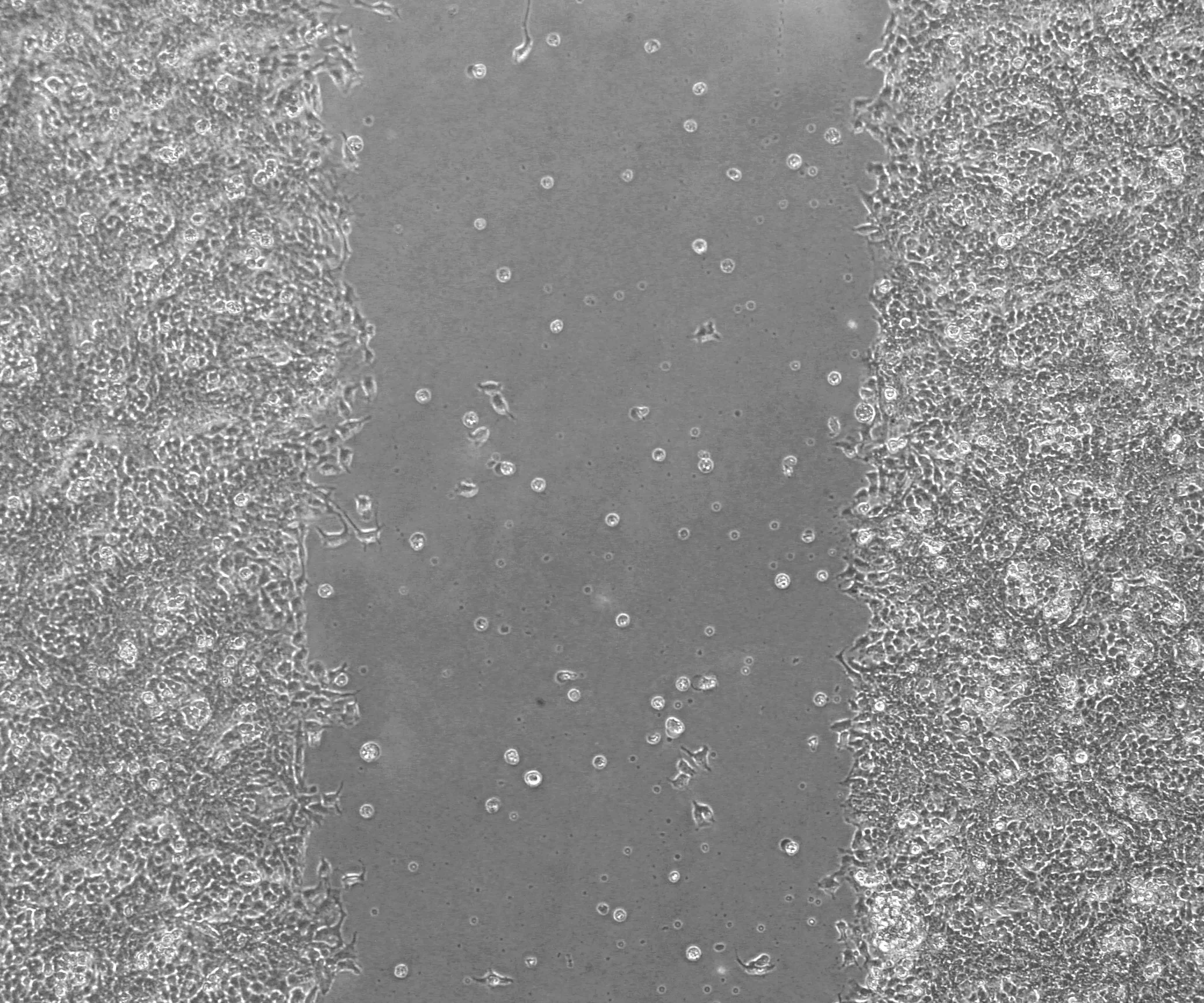

Supplement: Supplementary file 15 — Figure EV1-5 Source Data [file 44318_2025_416_MOESM15_ESM.zip › EMBOJ-2024-119243R_SourceDataForExpandedView/EMBOJ-2024-119243R_SourceDataForFigure EV2/EV2B/DLD1-24h-HO-shNT.jpg]

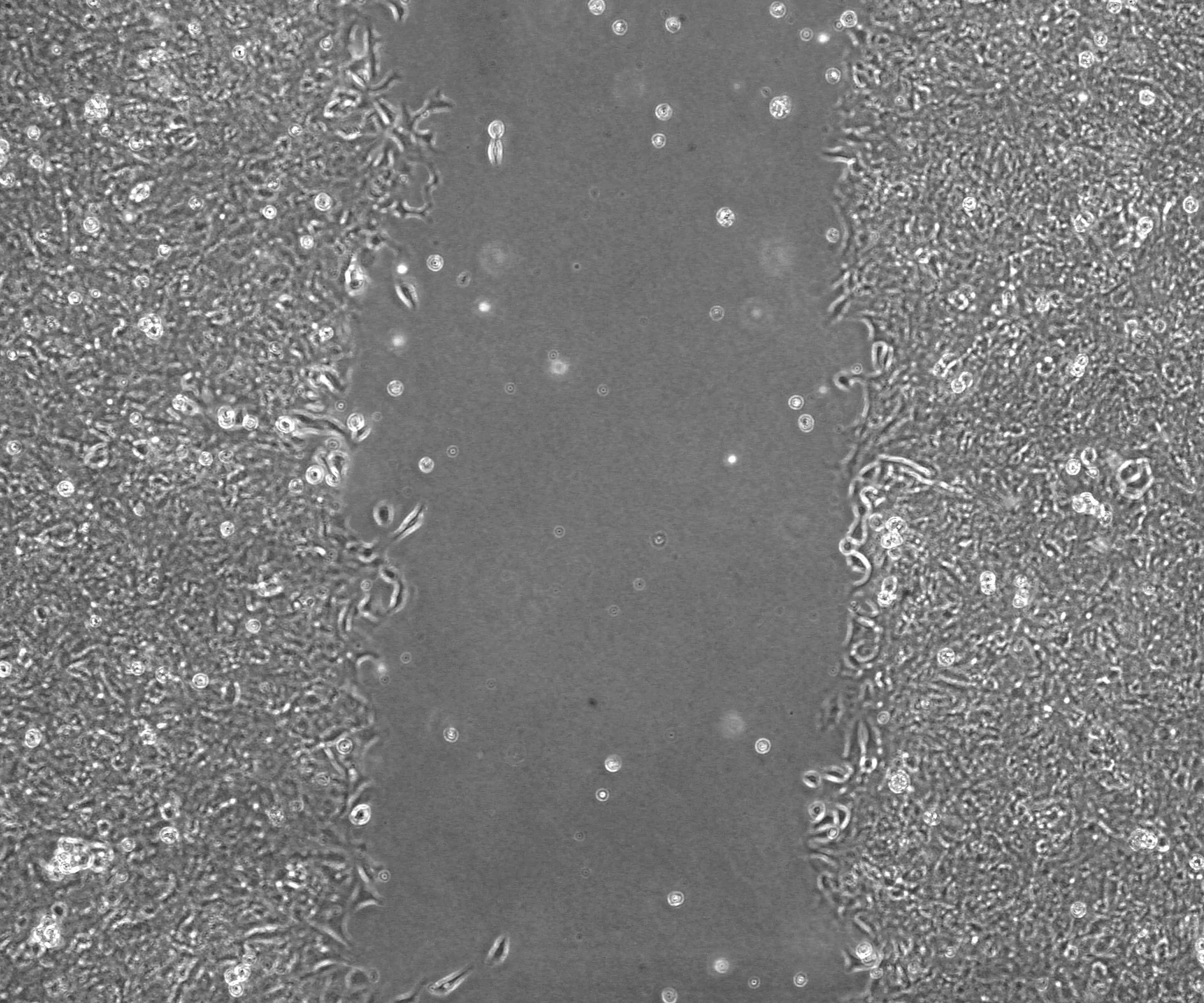

Supplement: Supplementary file 15 — Figure EV1-5 Source Data [file 44318_2025_416_MOESM15_ESM.zip › EMBOJ-2024-119243R_SourceDataForExpandedView/EMBOJ-2024-119243R_SourceDataForFigure EV2/EV2B/DLD1-24h-HO-shPPA2#1.jpg]

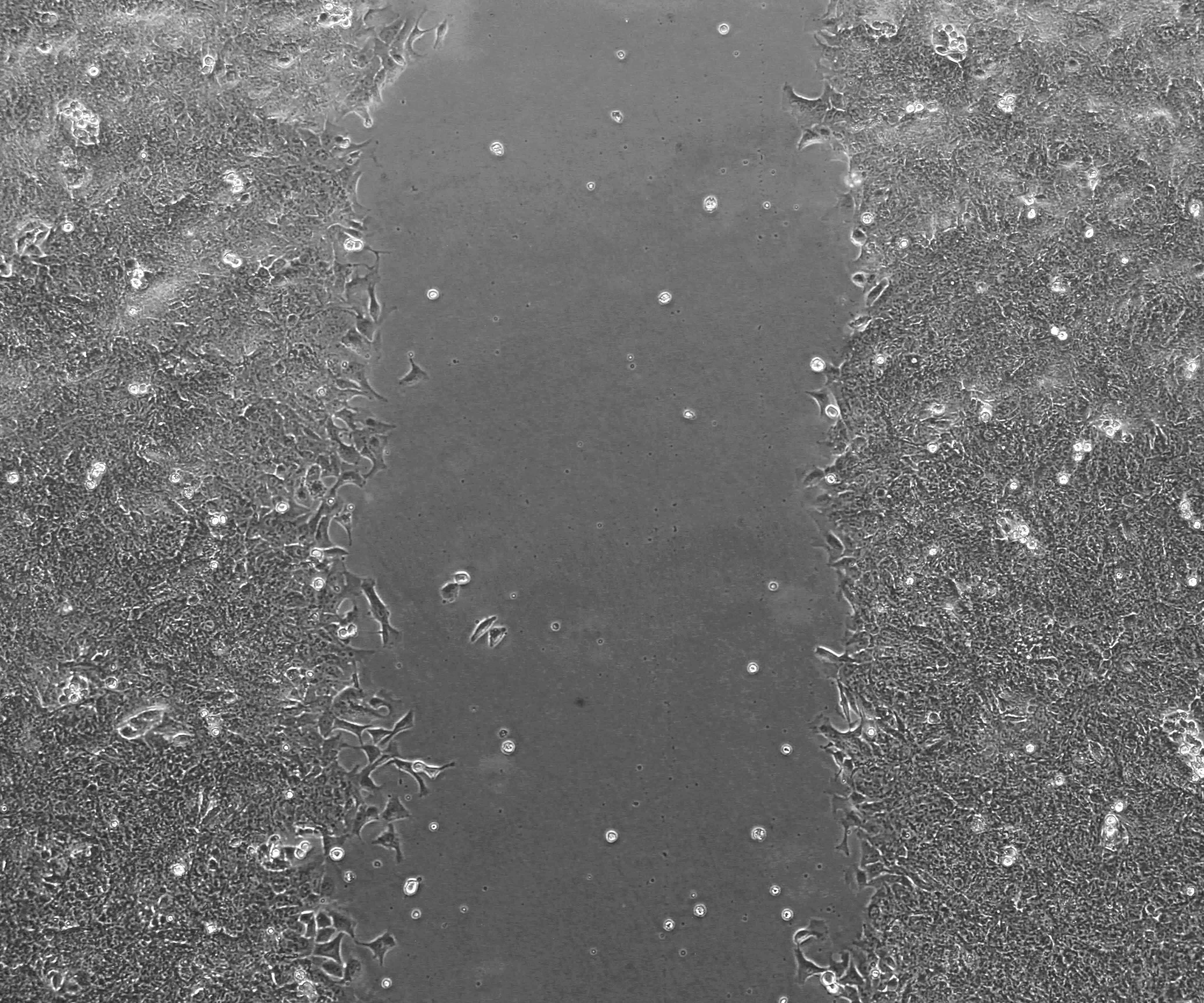

Supplement: Supplementary file 15 — Figure EV1-5 Source Data [file 44318_2025_416_MOESM15_ESM.zip › EMBOJ-2024-119243R_SourceDataForExpandedView/EMBOJ-2024-119243R_SourceDataForFigure EV2/EV2B/DLD1-24h-HO-shPPA2#2.jpg]

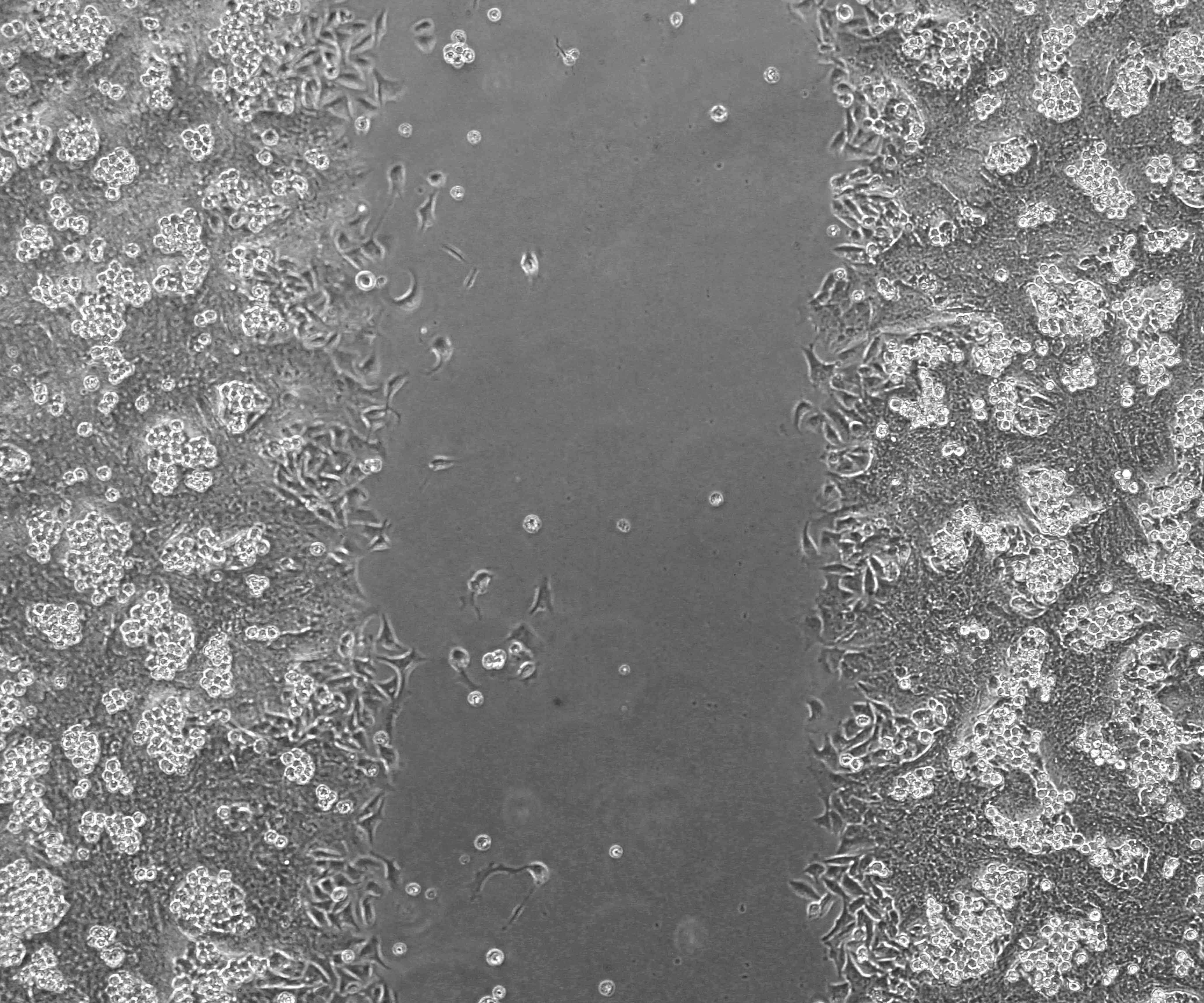

Supplement: Supplementary file 15 — Figure EV1-5 Source Data [file 44318_2025_416_MOESM15_ESM.zip › EMBOJ-2024-119243R_SourceDataForExpandedView/EMBOJ-2024-119243R_SourceDataForFigure EV2/EV2B/DLD1-24h-LO-shNT.jpg]

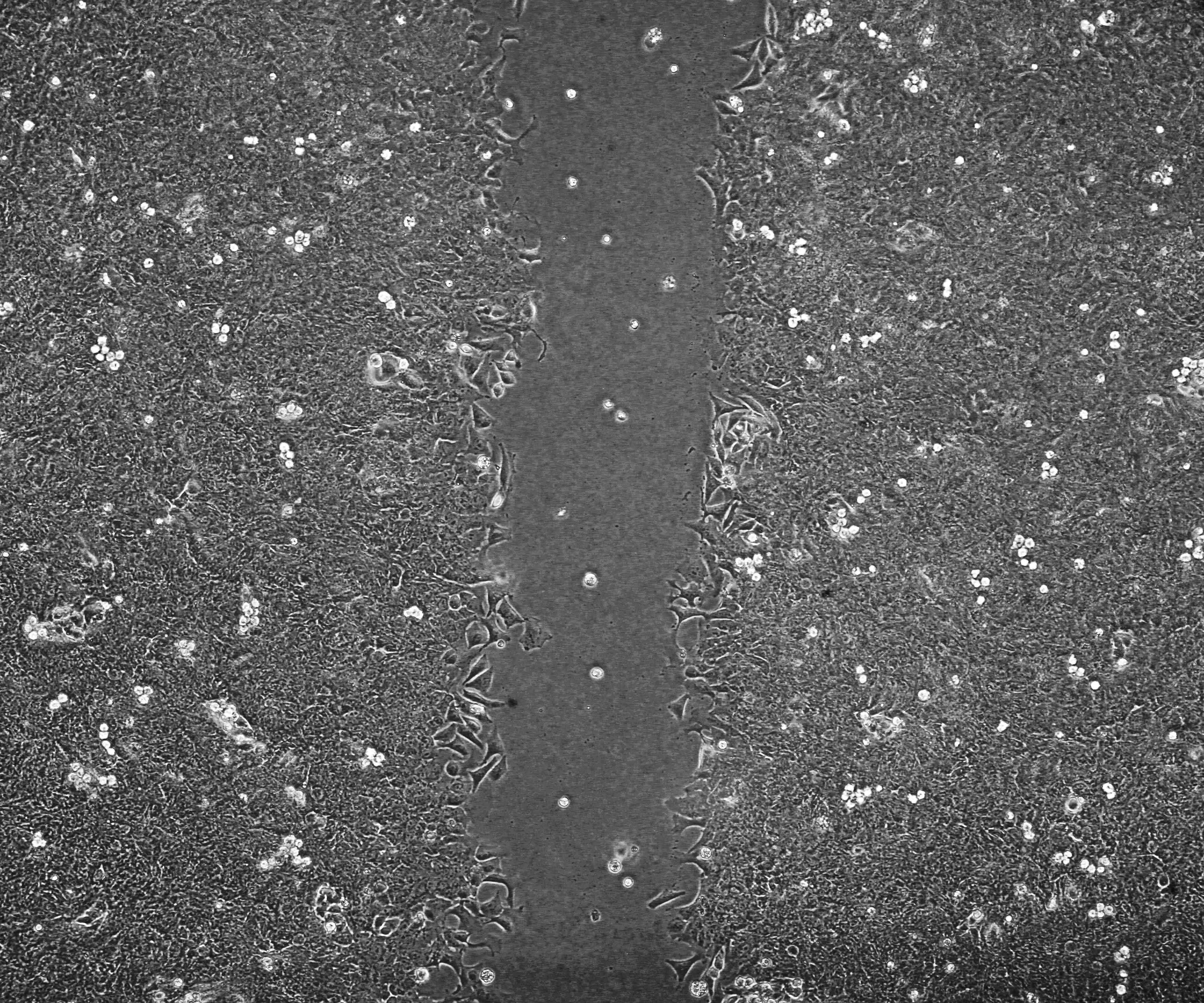

Supplement: Supplementary file 15 — Figure EV1-5 Source Data [file 44318_2025_416_MOESM15_ESM.zip › EMBOJ-2024-119243R_SourceDataForExpandedView/EMBOJ-2024-119243R_SourceDataForFigure EV2/EV2B/DLD1-24h-LO-shPPA2#1.jpg]

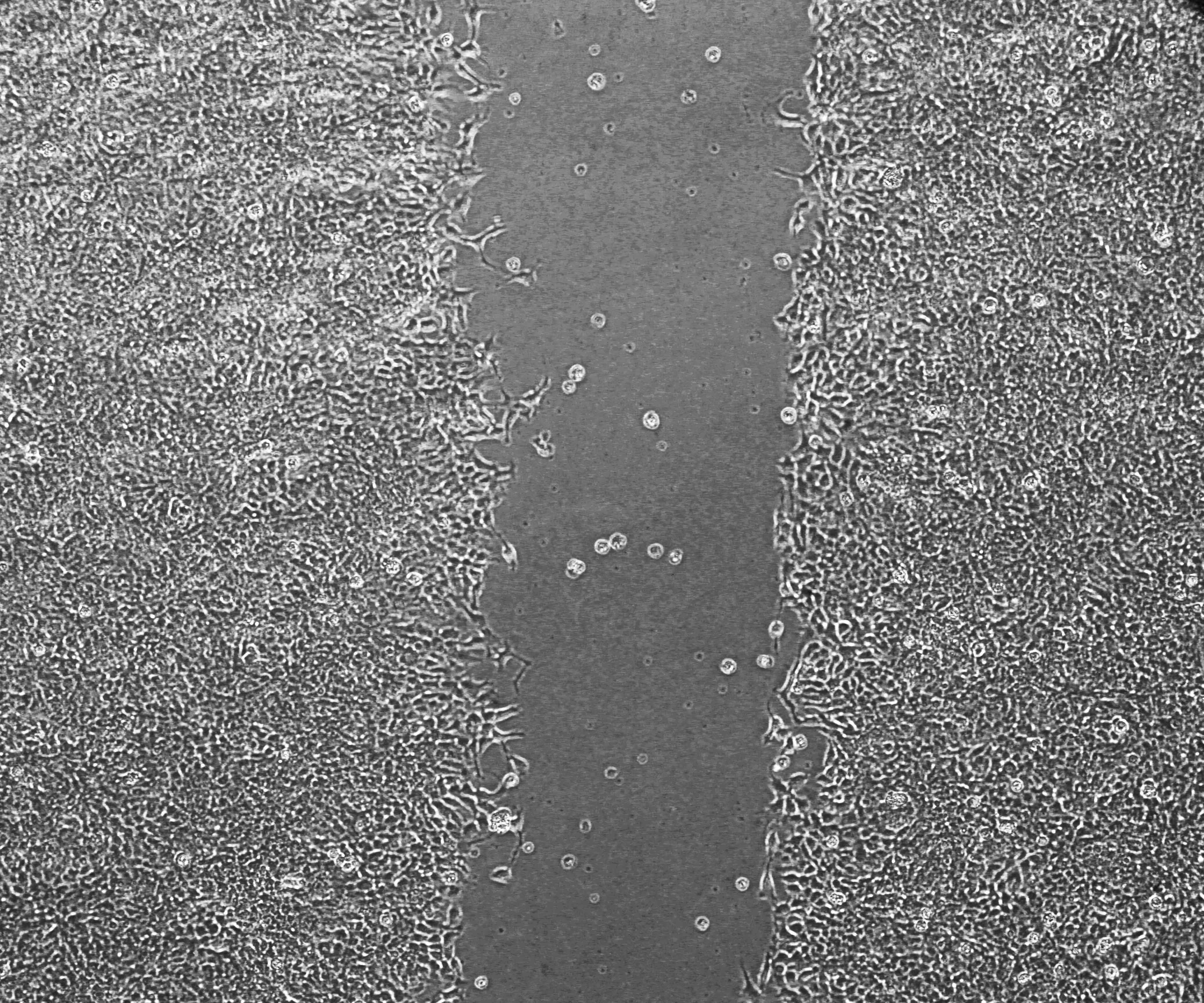

Supplement: Supplementary file 15 — Figure EV1-5 Source Data [file 44318_2025_416_MOESM15_ESM.zip › EMBOJ-2024-119243R_SourceDataForExpandedView/EMBOJ-2024-119243R_SourceDataForFigure EV2/EV2B/DLD1-24h-LO-shPPA2#2.jpg]

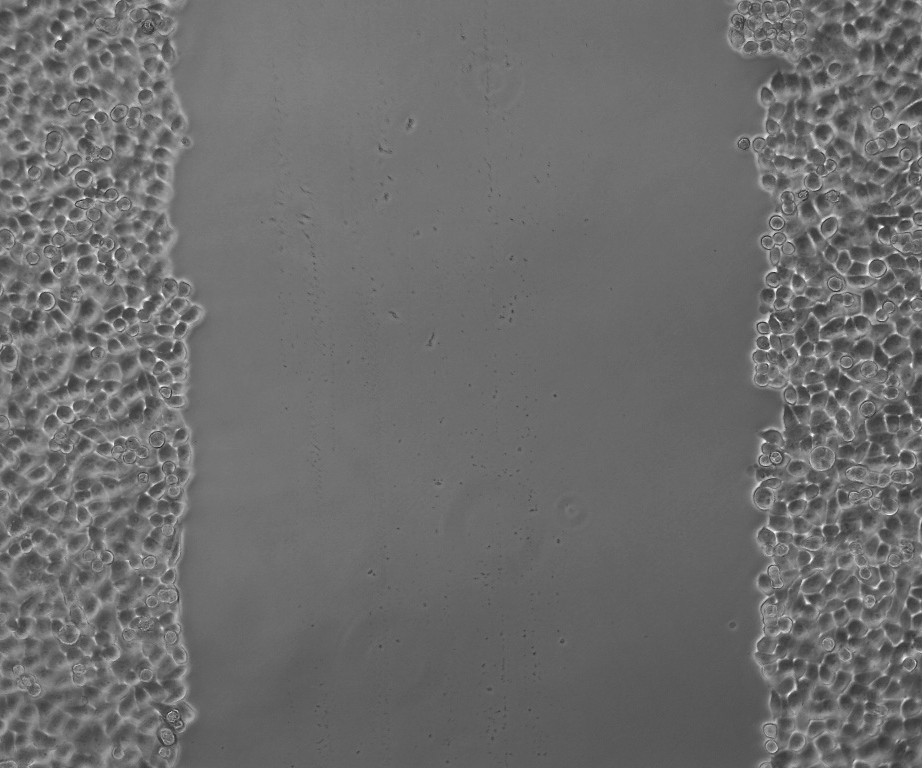

Supplement: Supplementary file 15 — Figure EV1-5 Source Data [file 44318_2025_416_MOESM15_ESM.zip › EMBOJ-2024-119243R_SourceDataForExpandedView/EMBOJ-2024-119243R_SourceDataForFigure EV2/EV2B/SW1116-0h-HO-shNT.jpg]

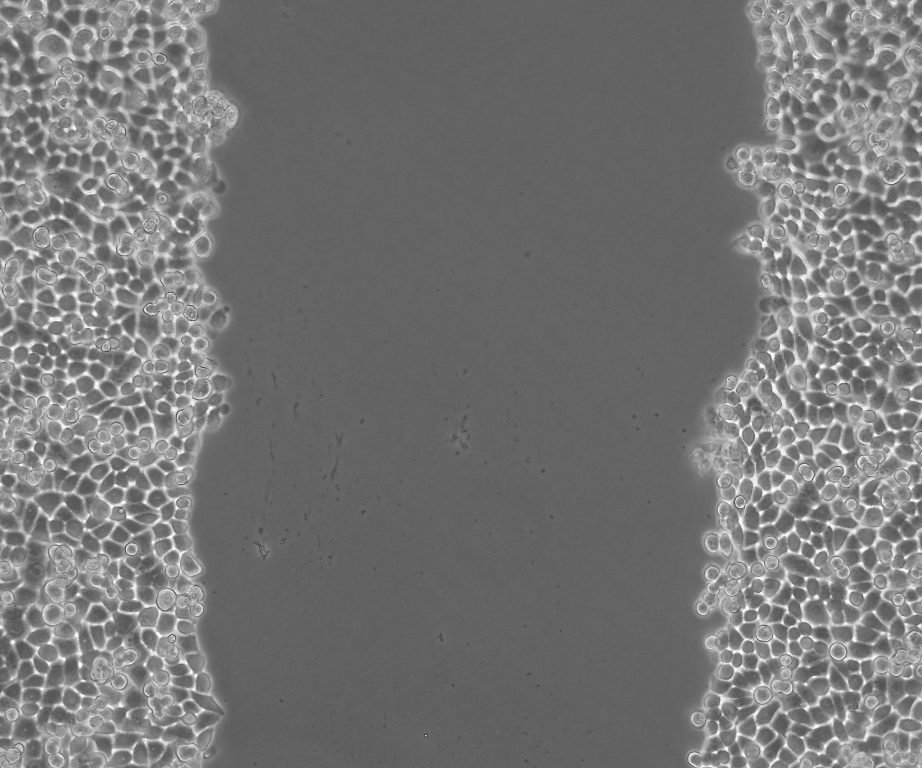

Supplement: Supplementary file 15 — Figure EV1-5 Source Data [file 44318_2025_416_MOESM15_ESM.zip › EMBOJ-2024-119243R_SourceDataForExpandedView/EMBOJ-2024-119243R_SourceDataForFigure EV2/EV2B/SW1116-0h-HO-shPPA2#1.jpg]

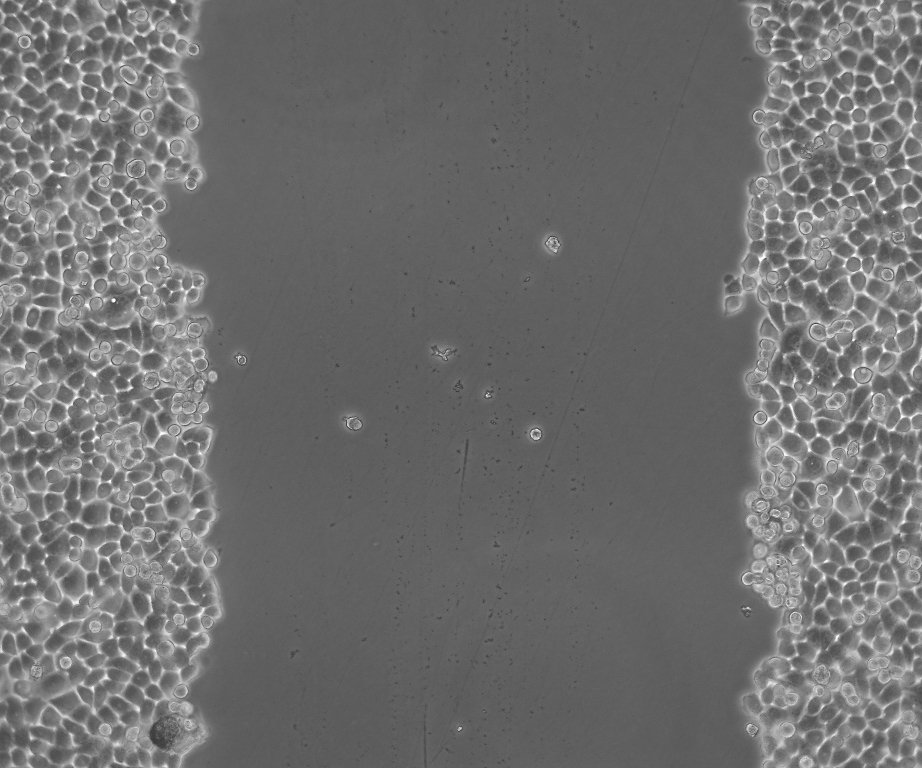

Supplement: Supplementary file 15 — Figure EV1-5 Source Data [file 44318_2025_416_MOESM15_ESM.zip › EMBOJ-2024-119243R_SourceDataForExpandedView/EMBOJ-2024-119243R_SourceDataForFigure EV2/EV2B/SW1116-0h-HO-shPPA2#2.jpg]

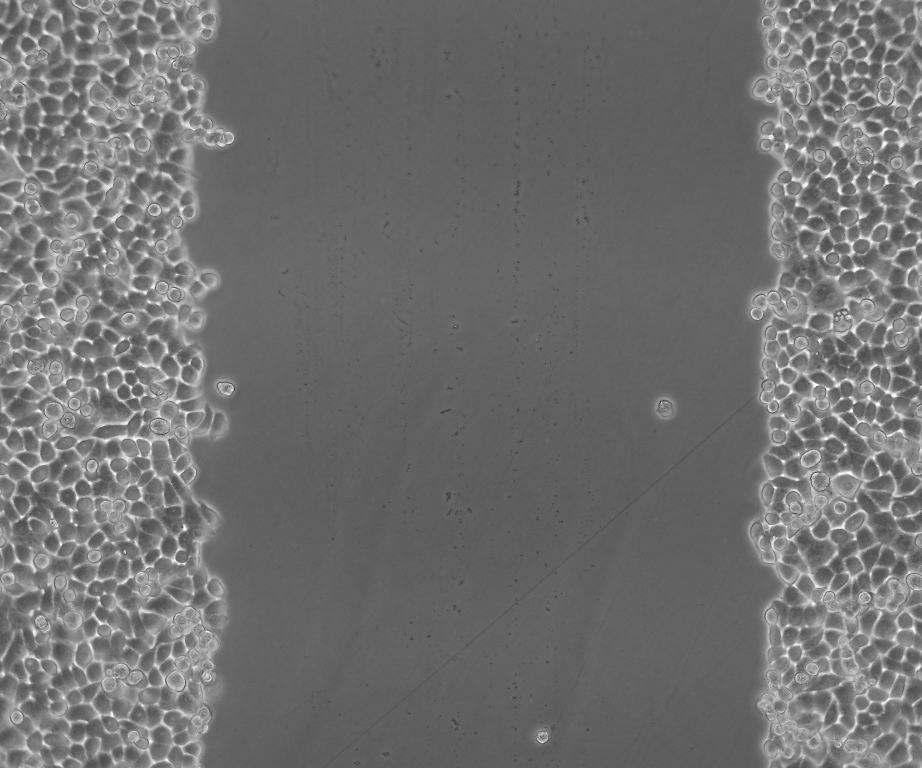

Supplement: Supplementary file 15 — Figure EV1-5 Source Data [file 44318_2025_416_MOESM15_ESM.zip › EMBOJ-2024-119243R_SourceDataForExpandedView/EMBOJ-2024-119243R_SourceDataForFigure EV2/EV2B/SW1116-0h-LO-shNT.jpg]

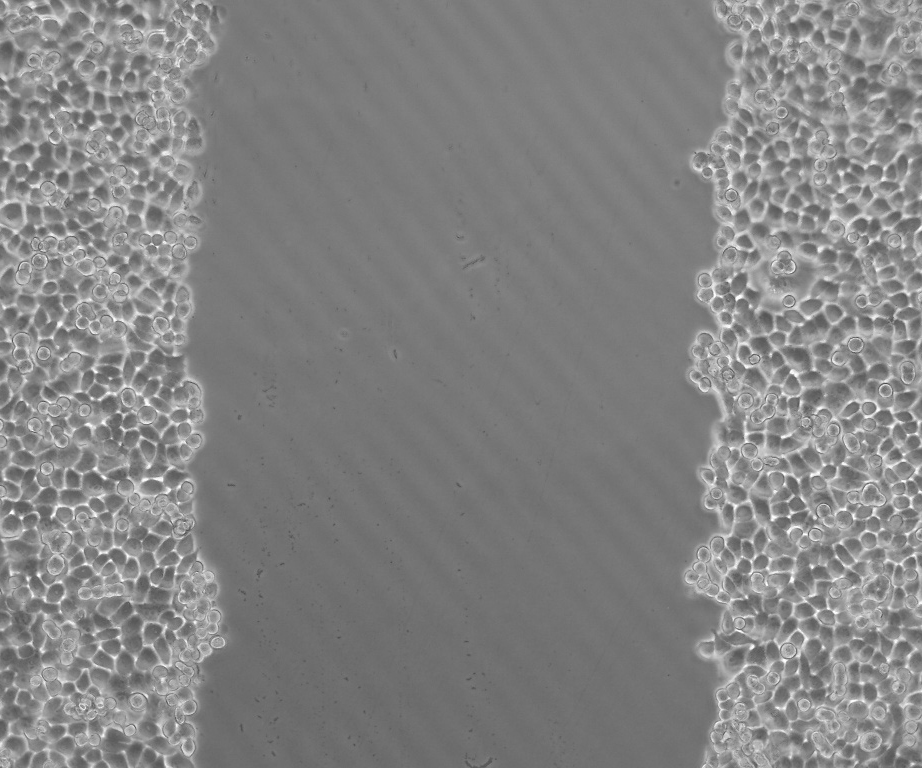

Supplement: Supplementary file 15 — Figure EV1-5 Source Data [file 44318_2025_416_MOESM15_ESM.zip › EMBOJ-2024-119243R_SourceDataForExpandedView/EMBOJ-2024-119243R_SourceDataForFigure EV2/EV2B/SW1116-0h-LO-shPPA2#1.jpg]

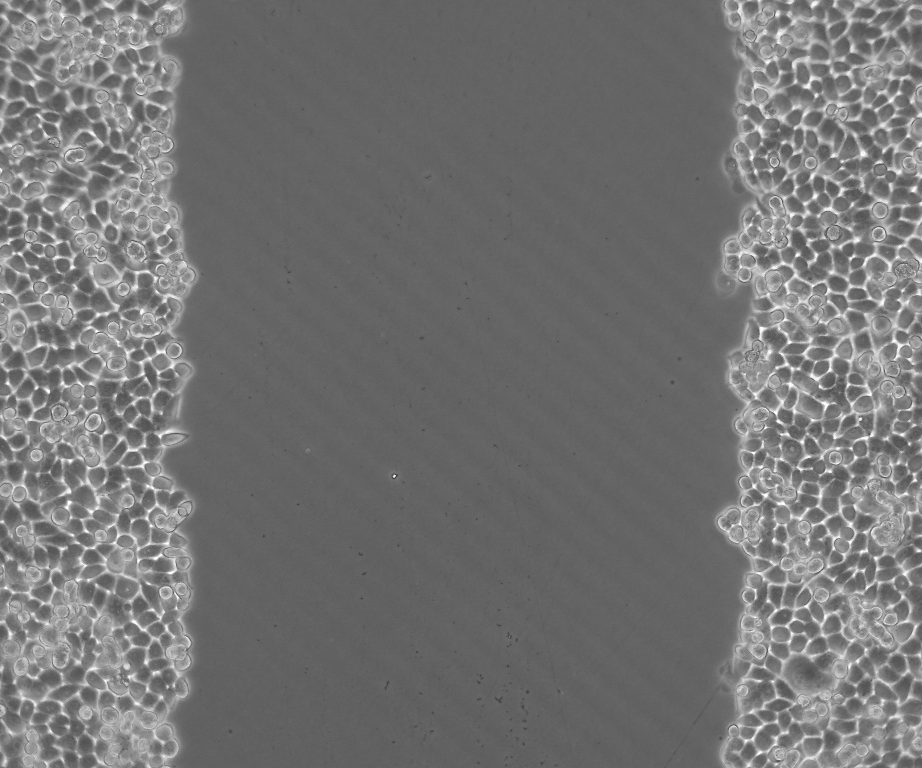

Supplement: Supplementary file 15 — Figure EV1-5 Source Data [file 44318_2025_416_MOESM15_ESM.zip › EMBOJ-2024-119243R_SourceDataForExpandedView/EMBOJ-2024-119243R_SourceDataForFigure EV2/EV2B/SW1116-0h-LO-shPPA2#2.jpg]

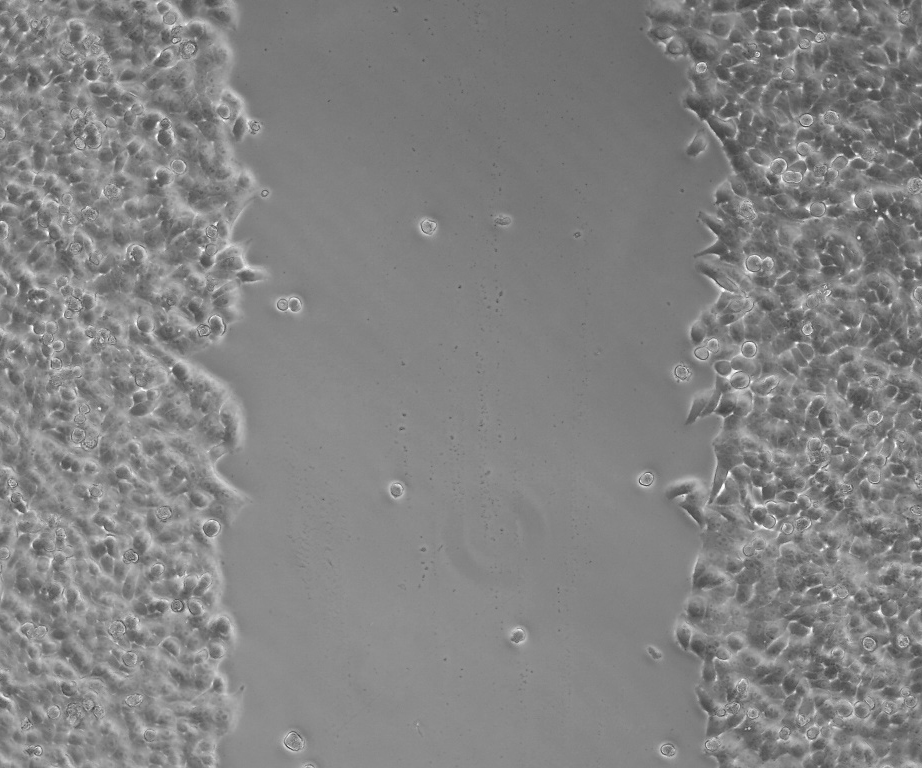

Supplement: Supplementary file 15 — Figure EV1-5 Source Data [file 44318_2025_416_MOESM15_ESM.zip › EMBOJ-2024-119243R_SourceDataForExpandedView/EMBOJ-2024-119243R_SourceDataForFigure EV2/EV2B/SW1116-24h-HO-shNT.jpg]

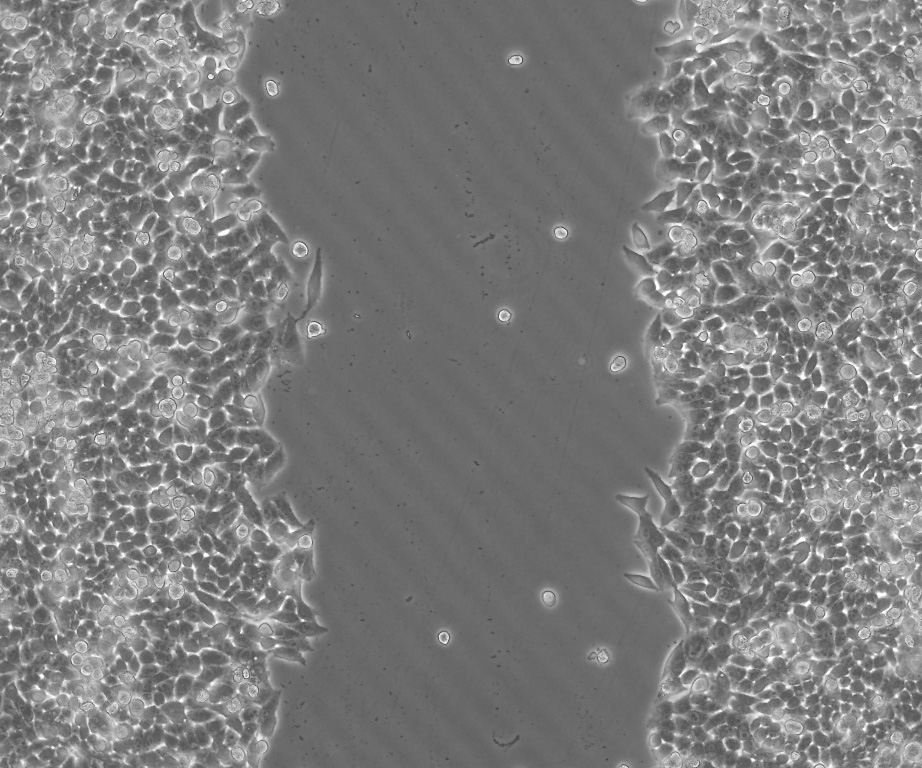

Supplement: Supplementary file 15 — Figure EV1-5 Source Data [file 44318_2025_416_MOESM15_ESM.zip › EMBOJ-2024-119243R_SourceDataForExpandedView/EMBOJ-2024-119243R_SourceDataForFigure EV2/EV2B/SW1116-HO-24h-shPPA2#1.jpg]

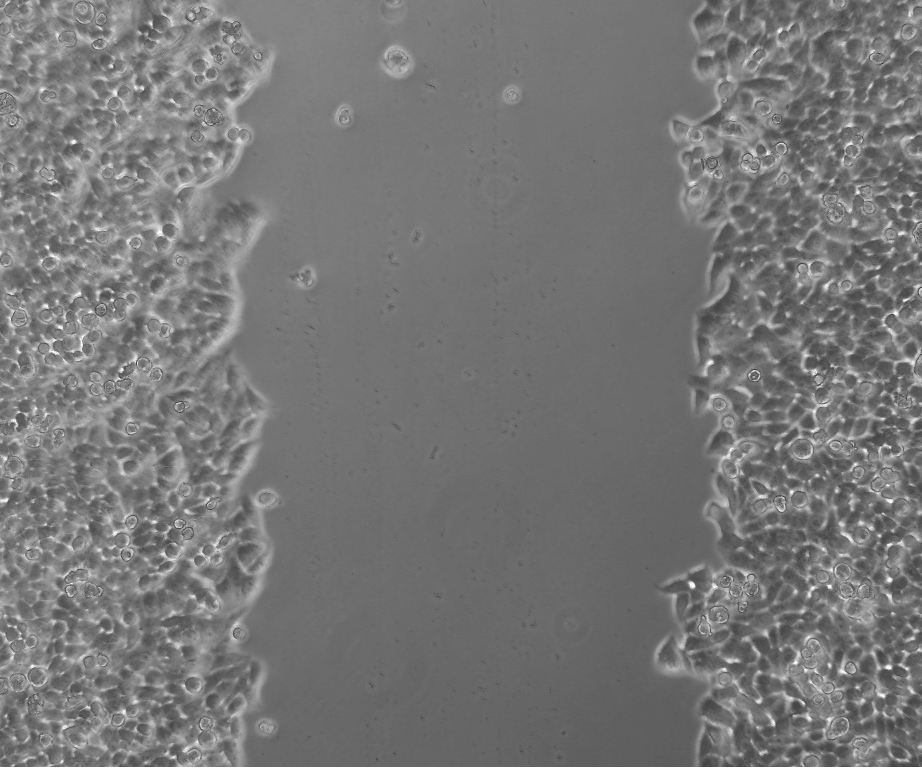

Supplement: Supplementary file 15 — Figure EV1-5 Source Data [file 44318_2025_416_MOESM15_ESM.zip › EMBOJ-2024-119243R_SourceDataForExpandedView/EMBOJ-2024-119243R_SourceDataForFigure EV2/EV2B/SW1116-HO-24h-shPPA2#2.jpg]

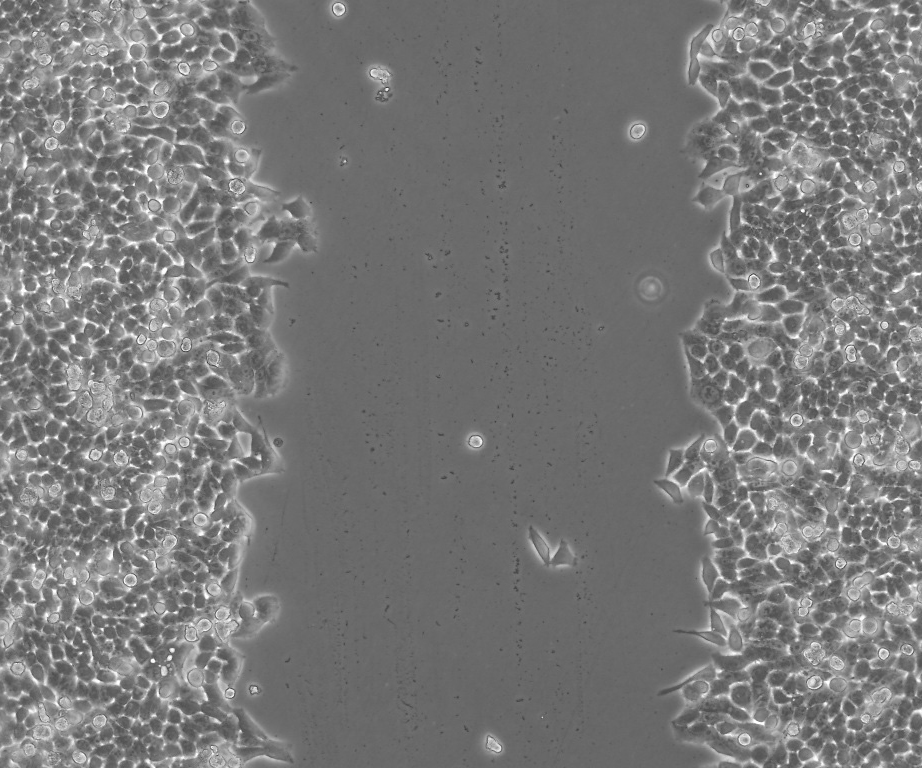

Supplement: Supplementary file 15 — Figure EV1-5 Source Data [file 44318_2025_416_MOESM15_ESM.zip › EMBOJ-2024-119243R_SourceDataForExpandedView/EMBOJ-2024-119243R_SourceDataForFigure EV2/EV2B/SW1116-LO-24h-shNT.jpg]

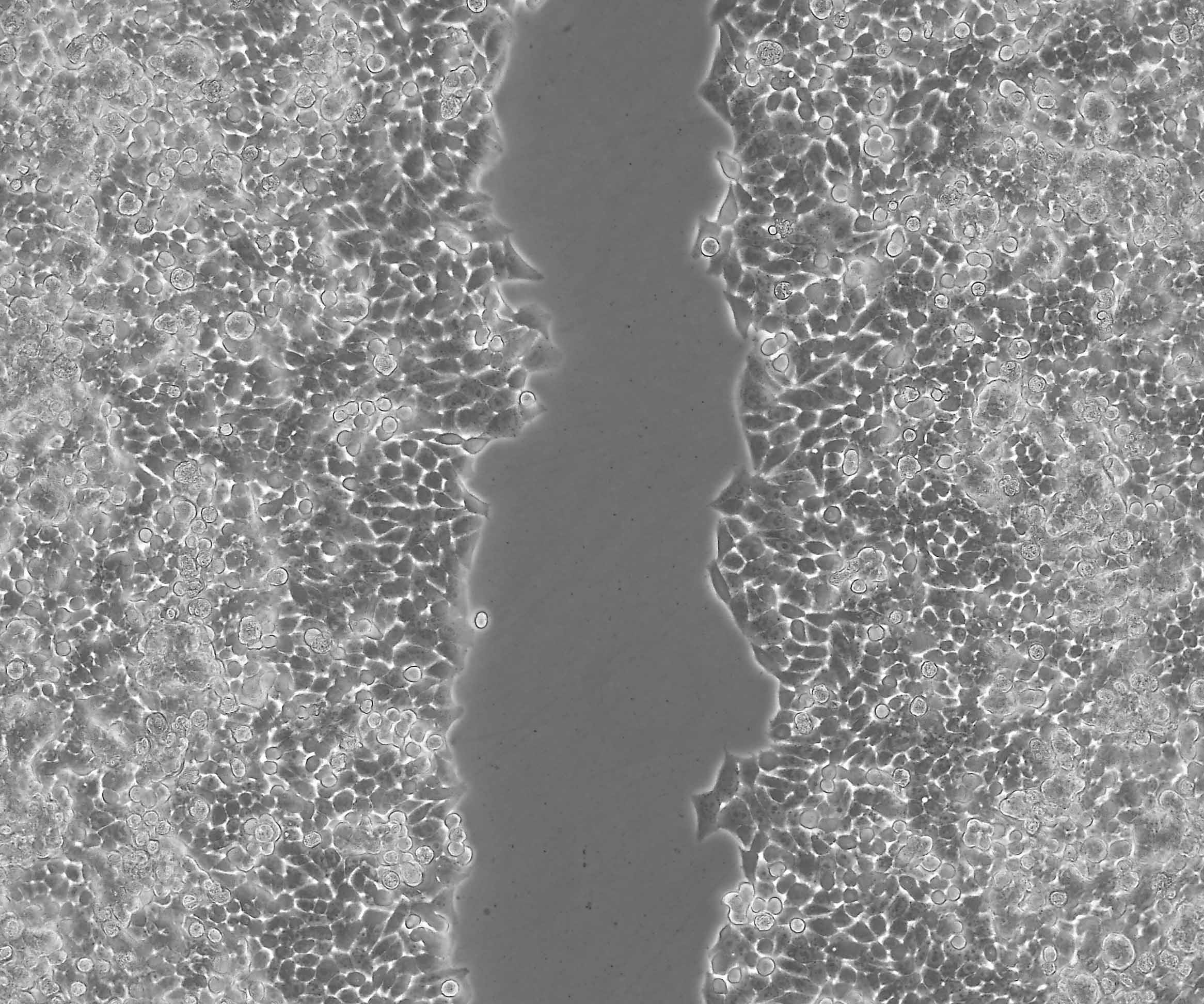

Supplement: Supplementary file 15 — Figure EV1-5 Source Data [file 44318_2025_416_MOESM15_ESM.zip › EMBOJ-2024-119243R_SourceDataForExpandedView/EMBOJ-2024-119243R_SourceDataForFigure EV2/EV2B/SW1116-LO-24h-shPPA2#1.jpg]

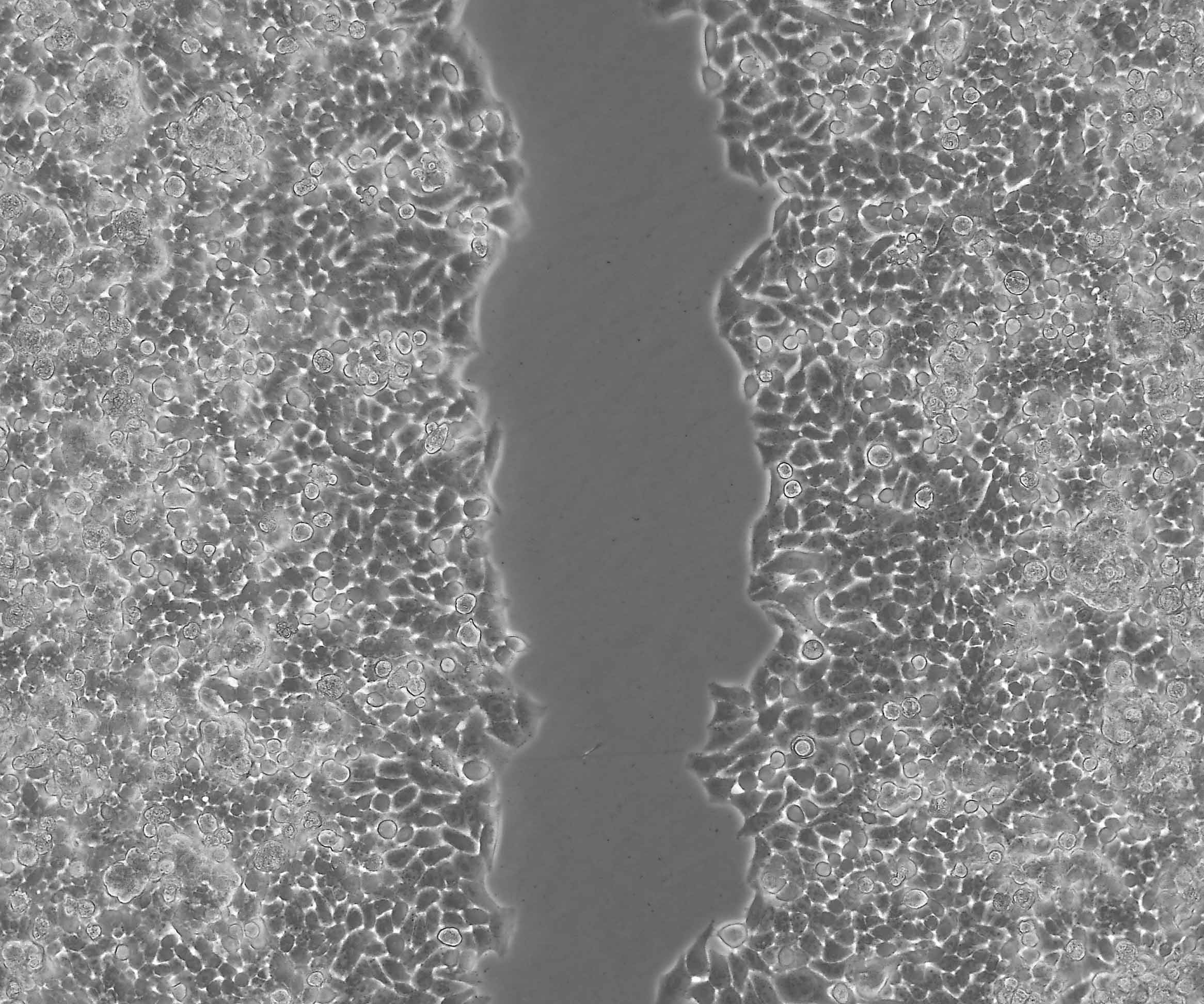

Supplement: Supplementary file 15 — Figure EV1-5 Source Data [file 44318_2025_416_MOESM15_ESM.zip › EMBOJ-2024-119243R_SourceDataForExpandedView/EMBOJ-2024-119243R_SourceDataForFigure EV2/EV2B/SW1116-LO-24h-shPPA2#2.jpg]

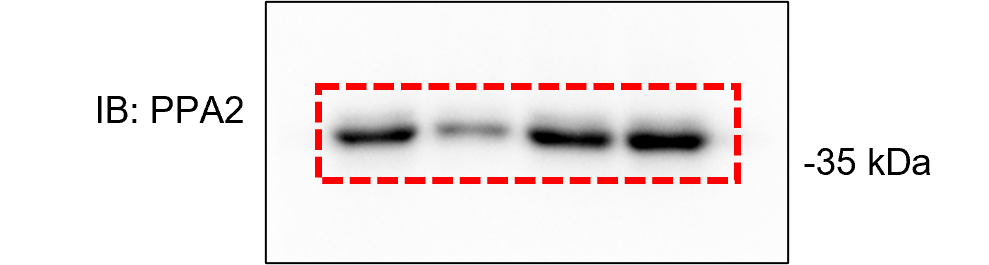

Supplement: Supplementary file 15 — Figure EV1-5 Source Data [file 44318_2025_416_MOESM15_ESM.zip › EMBOJ-2024-119243R_SourceDataForExpandedView/EMBOJ-2024-119243R_SourceDataForFigure EV2/EV2D/DLD1-PPA2.tif]

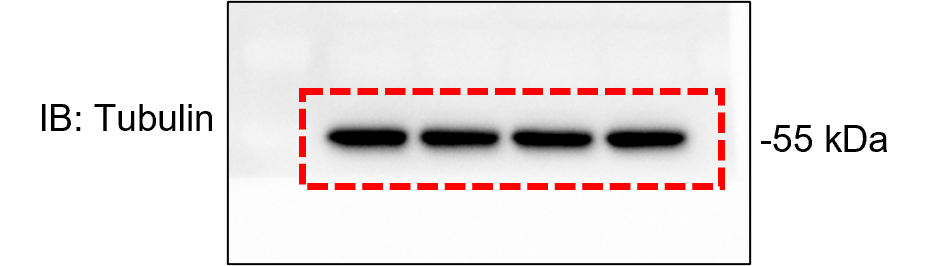

Supplement: Supplementary file 15 — Figure EV1-5 Source Data [file 44318_2025_416_MOESM15_ESM.zip › EMBOJ-2024-119243R_SourceDataForExpandedView/EMBOJ-2024-119243R_SourceDataForFigure EV2/EV2D/DLD1-Tubulin.tif]

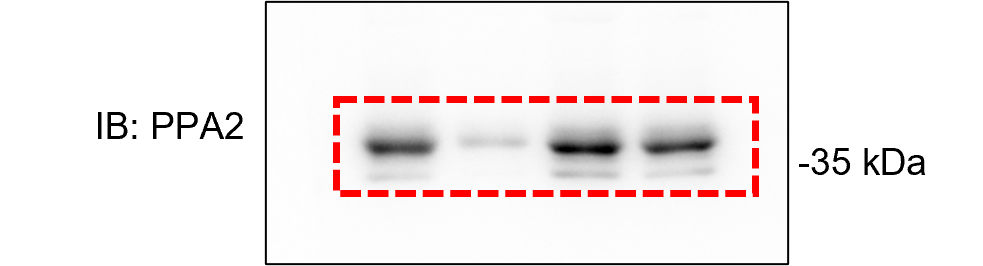

Supplement: Supplementary file 15 — Figure EV1-5 Source Data [file 44318_2025_416_MOESM15_ESM.zip › EMBOJ-2024-119243R_SourceDataForExpandedView/EMBOJ-2024-119243R_SourceDataForFigure EV2/EV2D/SW1116-PPA2.tif]

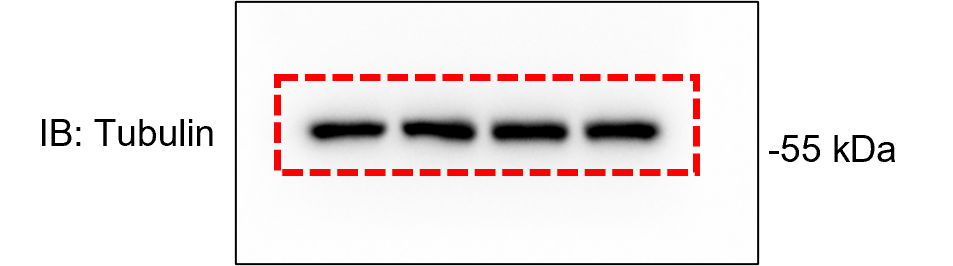

Supplement: Supplementary file 15 — Figure EV1-5 Source Data [file 44318_2025_416_MOESM15_ESM.zip › EMBOJ-2024-119243R_SourceDataForExpandedView/EMBOJ-2024-119243R_SourceDataForFigure EV2/EV2D/SW1116-Tubulin.tif]

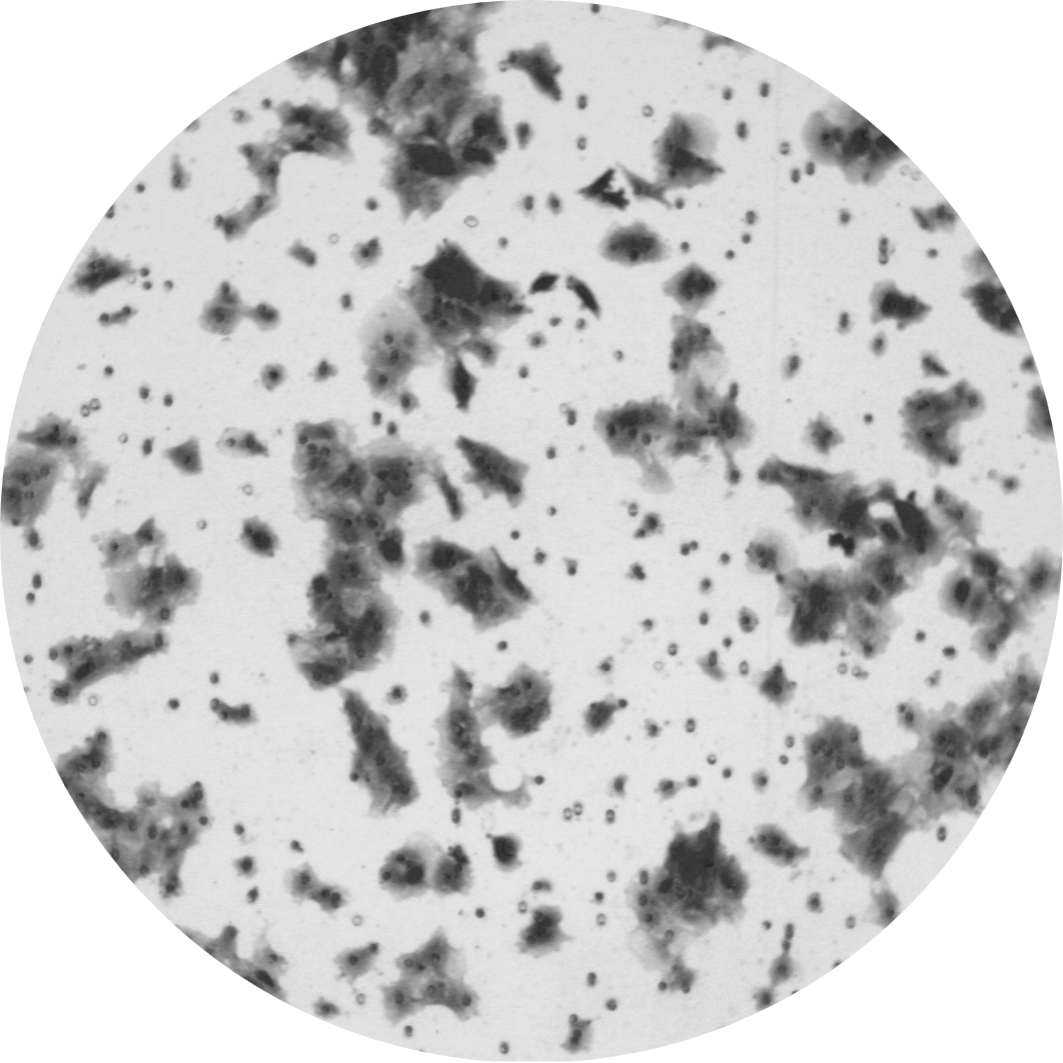

Supplement: Supplementary file 15 — Figure EV1-5 Source Data [file 44318_2025_416_MOESM15_ESM.zip › EMBOJ-2024-119243R_SourceDataForExpandedView/EMBOJ-2024-119243R_SourceDataForFigure EV2/EV2E/DLD1-HO-ED.tif]

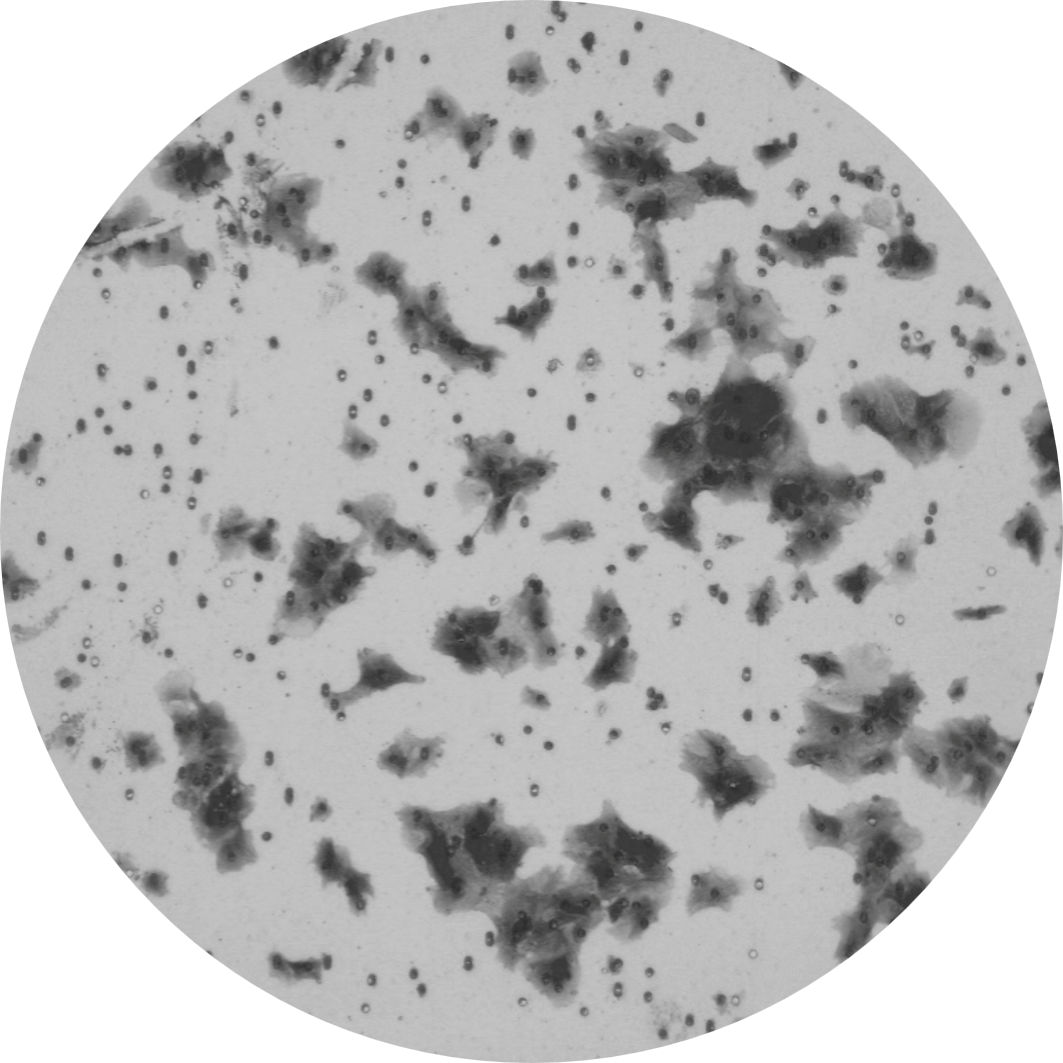

Supplement: Supplementary file 15 — Figure EV1-5 Source Data [file 44318_2025_416_MOESM15_ESM.zip › EMBOJ-2024-119243R_SourceDataForExpandedView/EMBOJ-2024-119243R_SourceDataForFigure EV2/EV2E/DLD1-HO-shPPA2.tif]

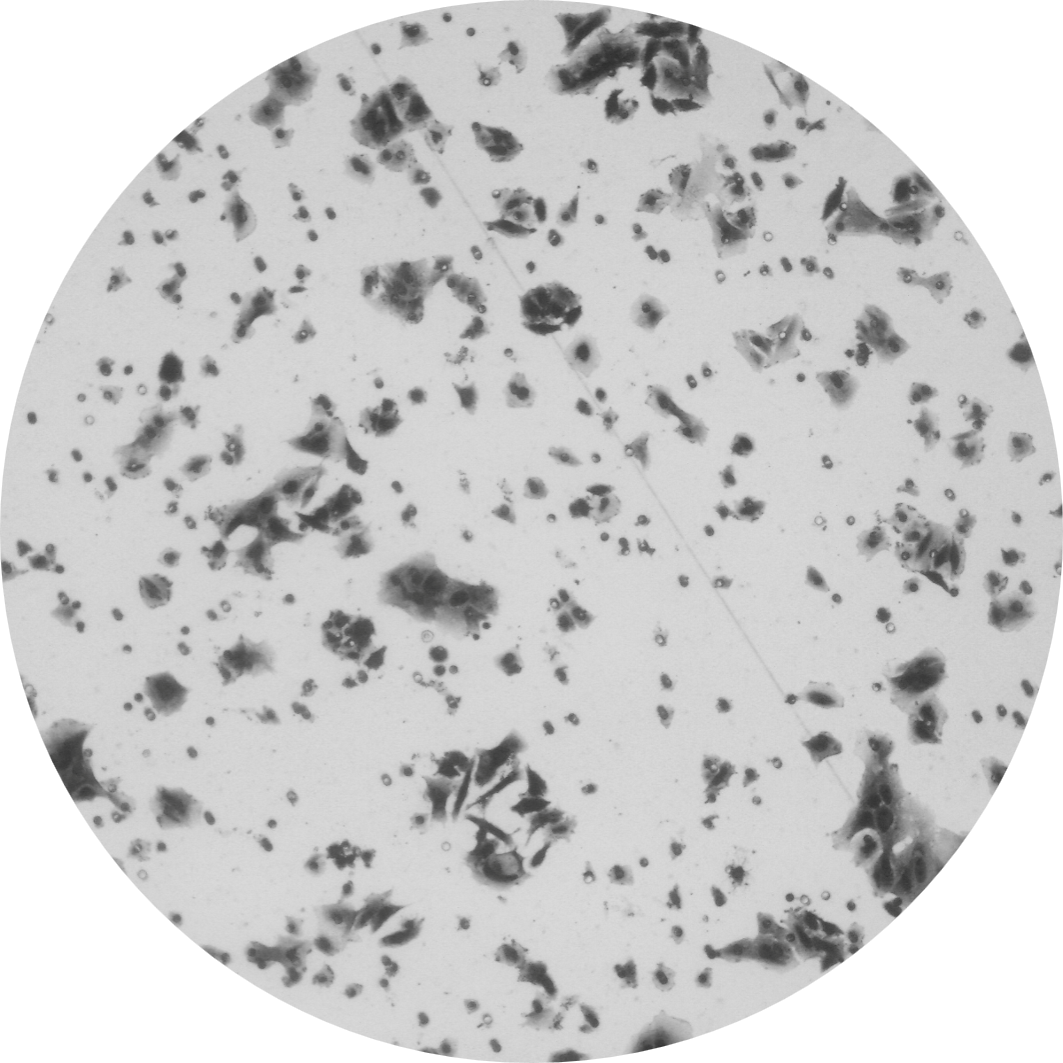

Supplement: Supplementary file 15 — Figure EV1-5 Source Data [file 44318_2025_416_MOESM15_ESM.zip › EMBOJ-2024-119243R_SourceDataForExpandedView/EMBOJ-2024-119243R_SourceDataForFigure EV2/EV2E/DLD1-HO-WT.tif]

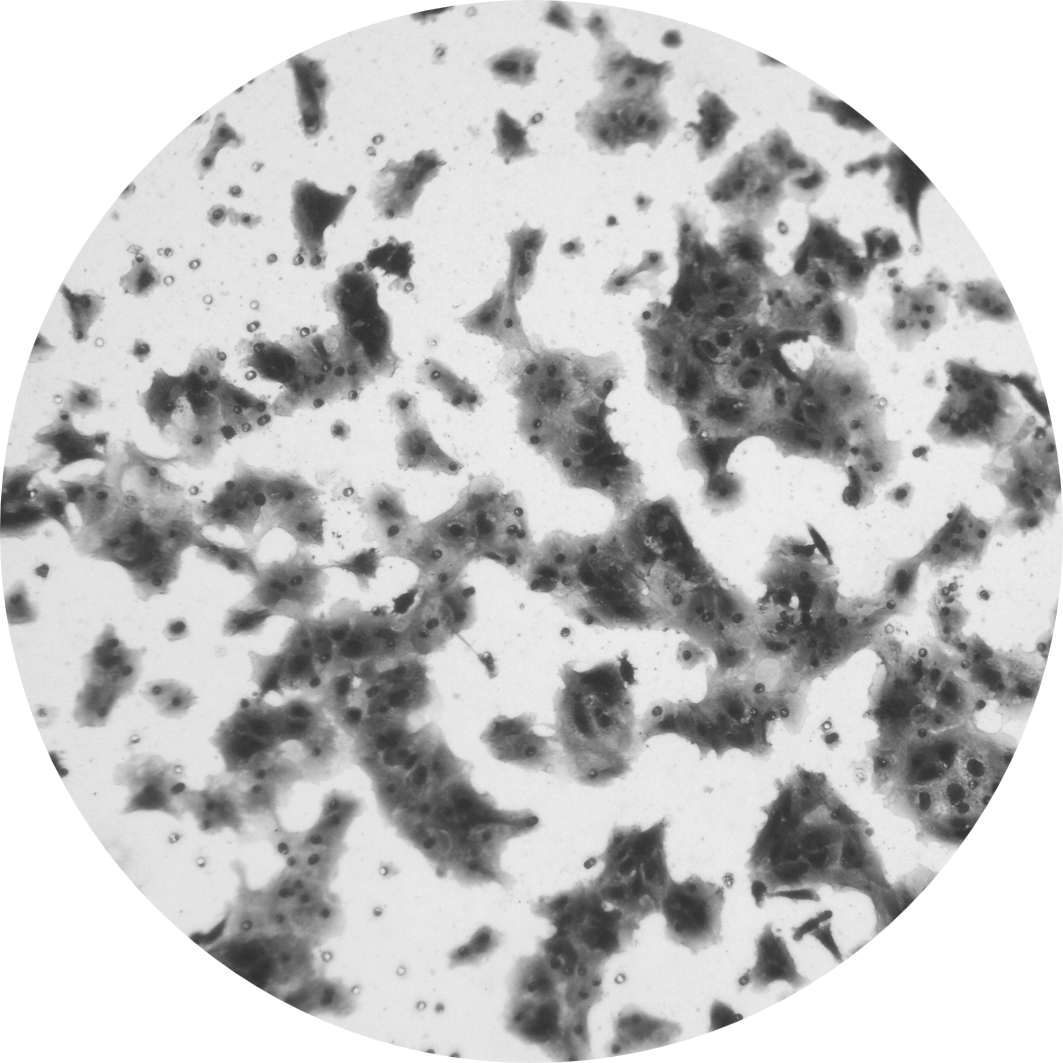

Supplement: Supplementary file 15 — Figure EV1-5 Source Data [file 44318_2025_416_MOESM15_ESM.zip › EMBOJ-2024-119243R_SourceDataForExpandedView/EMBOJ-2024-119243R_SourceDataForFigure EV2/EV2E/DLD1-LO-ED.tif]

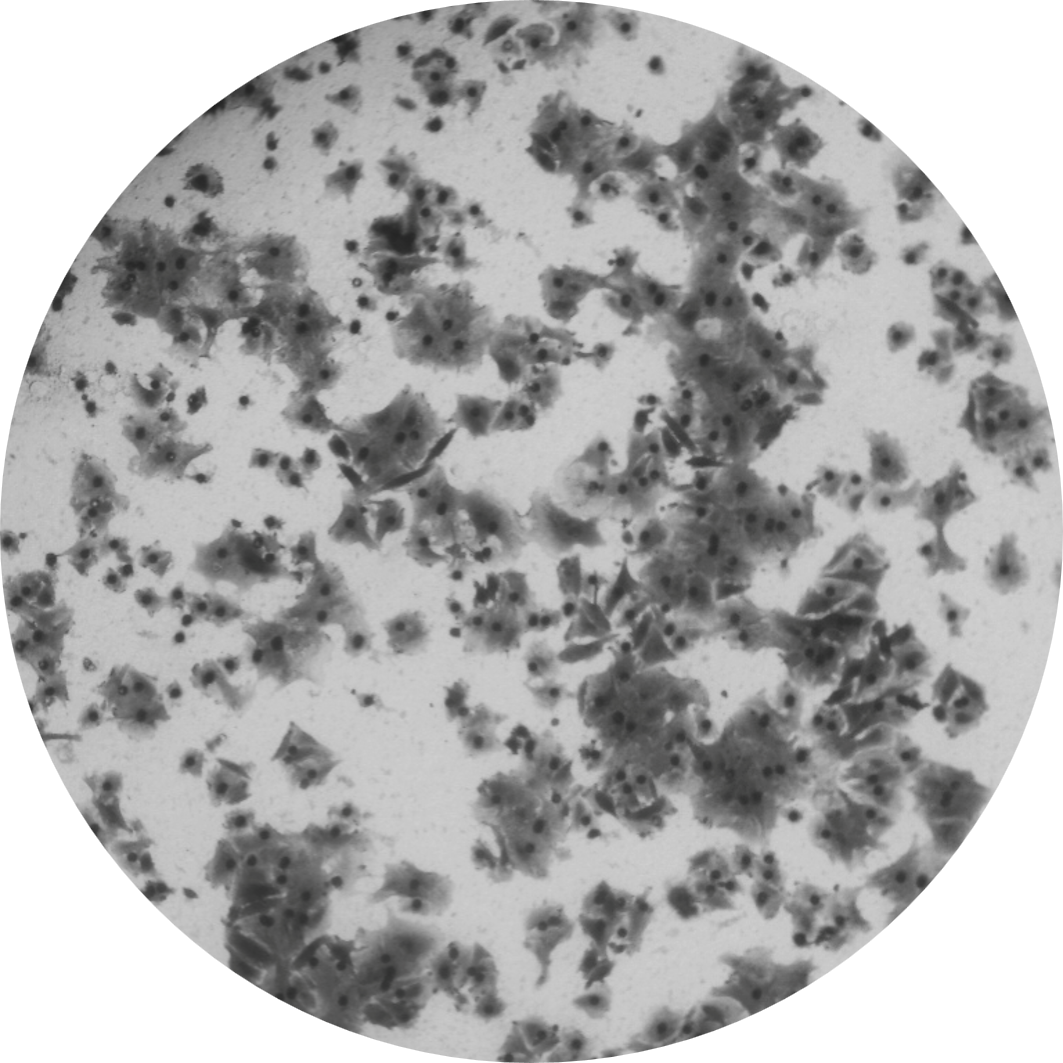

Supplement: Supplementary file 15 — Figure EV1-5 Source Data [file 44318_2025_416_MOESM15_ESM.zip › EMBOJ-2024-119243R_SourceDataForExpandedView/EMBOJ-2024-119243R_SourceDataForFigure EV2/EV2E/DLD1-LO-shPPA2.tif]

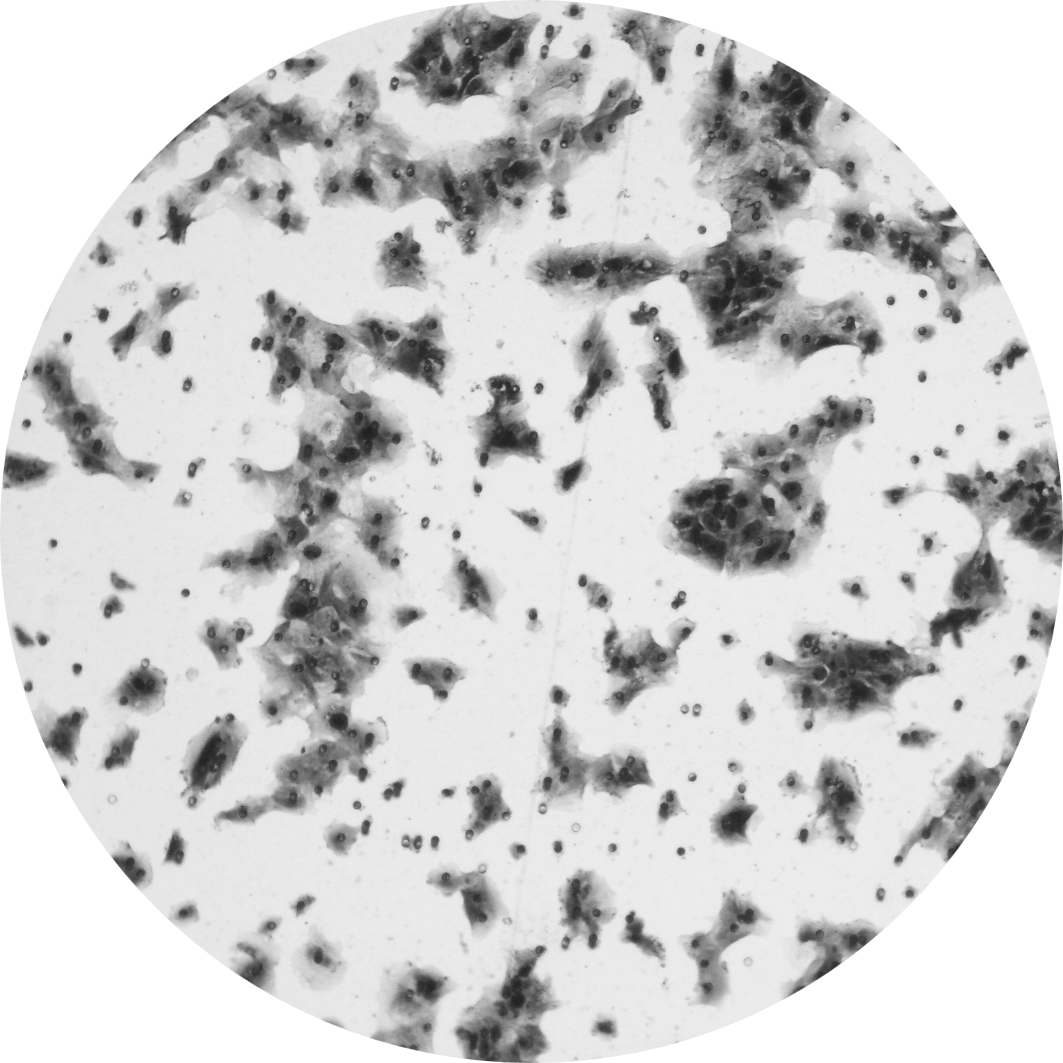

Supplement: Supplementary file 15 — Figure EV1-5 Source Data [file 44318_2025_416_MOESM15_ESM.zip › EMBOJ-2024-119243R_SourceDataForExpandedView/EMBOJ-2024-119243R_SourceDataForFigure EV2/EV2E/DLD1-LO-WT.tif]

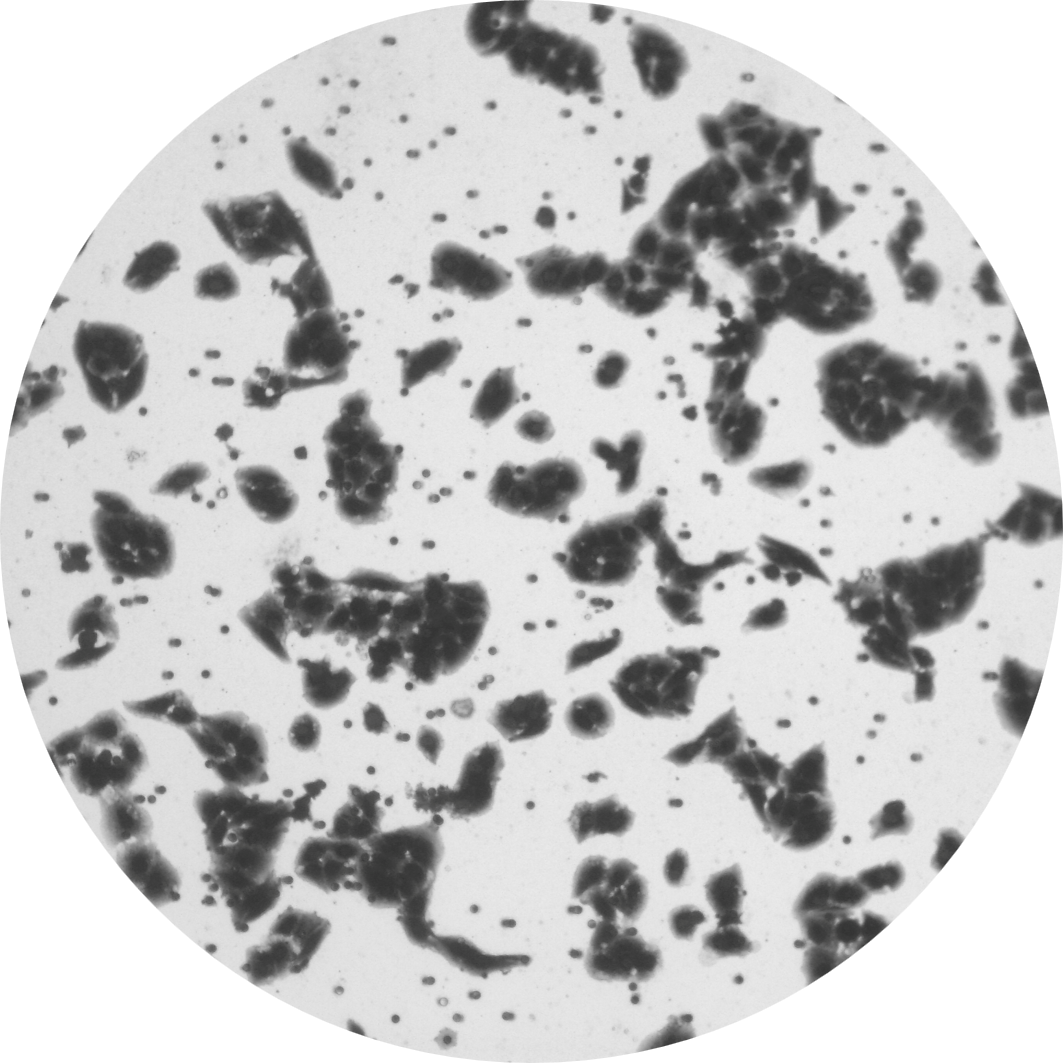

Supplement: Supplementary file 15 — Figure EV1-5 Source Data [file 44318_2025_416_MOESM15_ESM.zip › EMBOJ-2024-119243R_SourceDataForExpandedView/EMBOJ-2024-119243R_SourceDataForFigure EV2/EV2E/SW1116-HO-ED.tif]

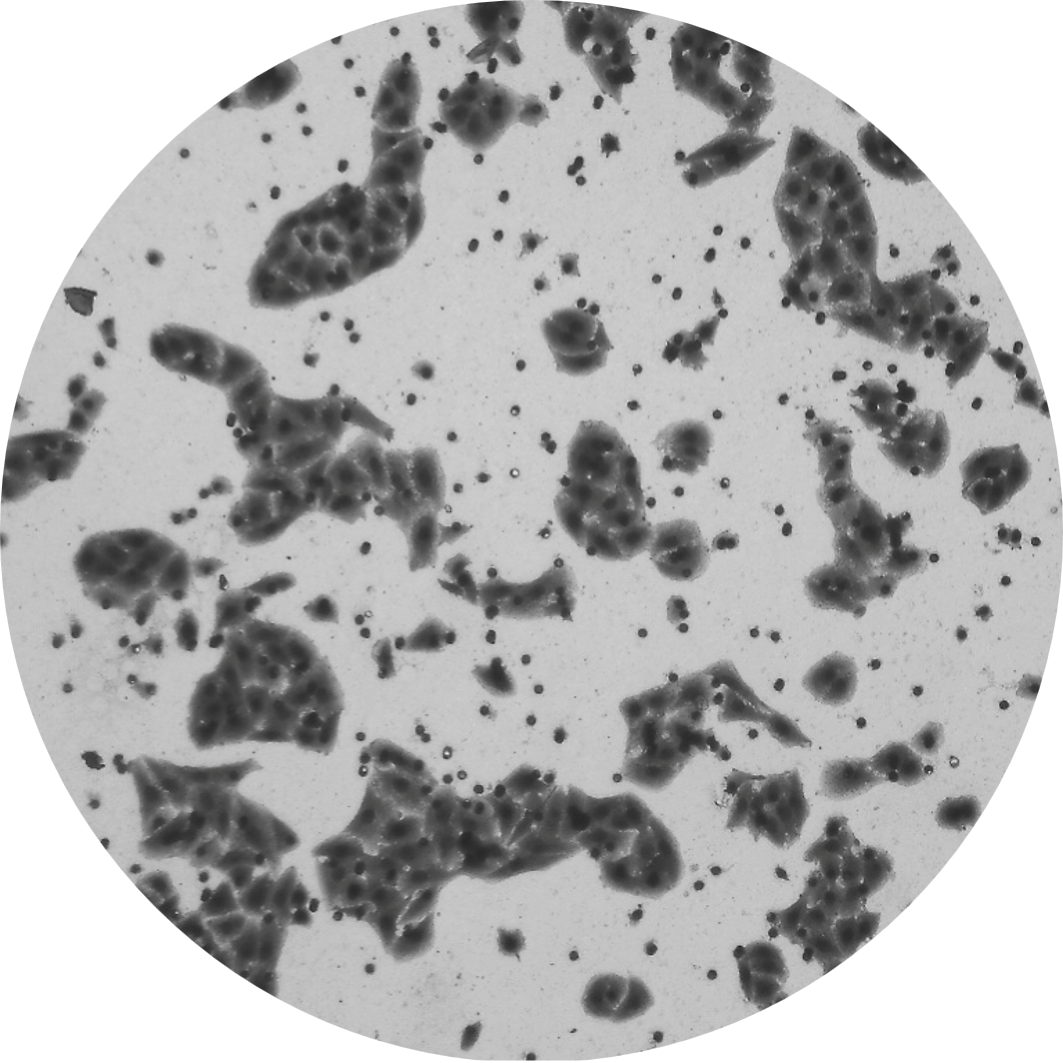

Supplement: Supplementary file 15 — Figure EV1-5 Source Data [file 44318_2025_416_MOESM15_ESM.zip › EMBOJ-2024-119243R_SourceDataForExpandedView/EMBOJ-2024-119243R_SourceDataForFigure EV2/EV2E/SW1116-HO-shPPA2.tif]

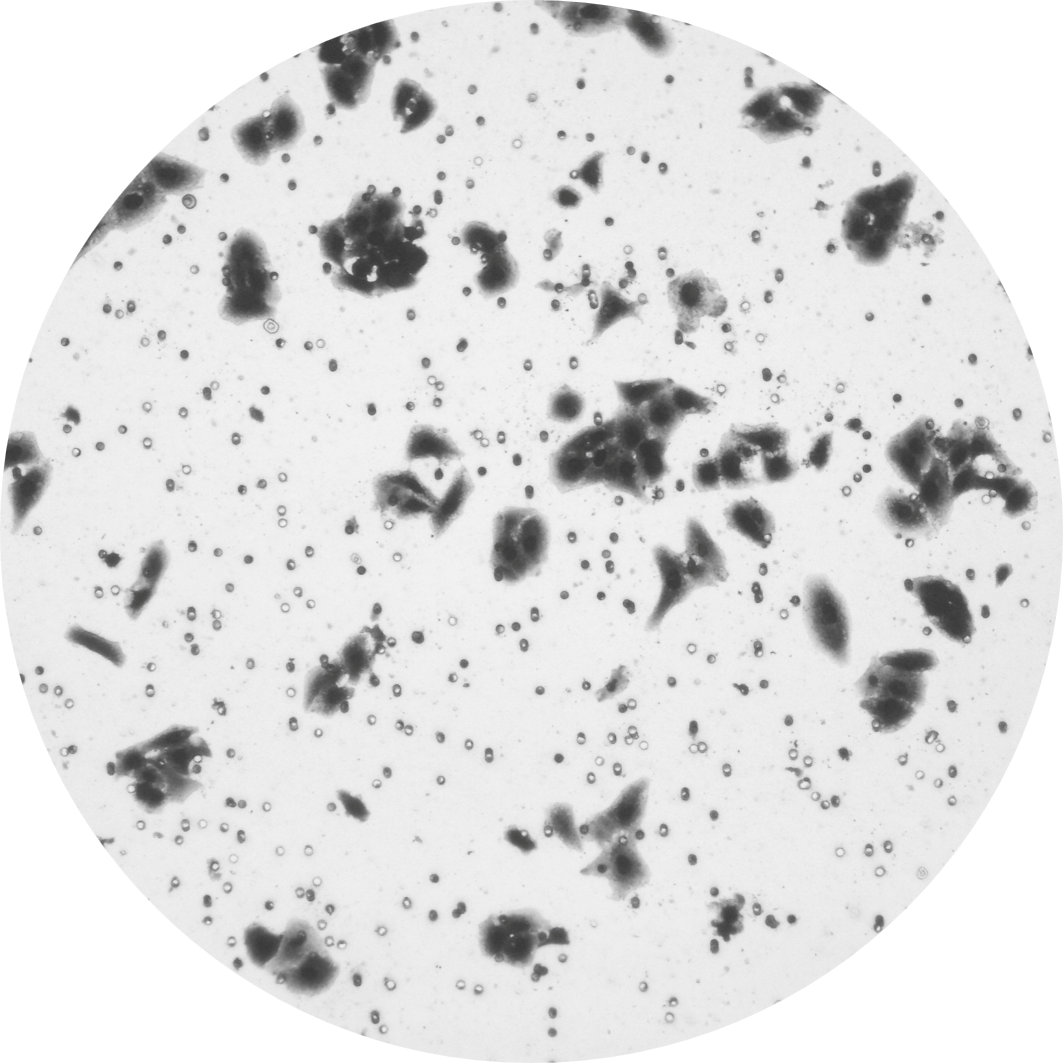

Supplement: Supplementary file 15 — Figure EV1-5 Source Data [file 44318_2025_416_MOESM15_ESM.zip › EMBOJ-2024-119243R_SourceDataForExpandedView/EMBOJ-2024-119243R_SourceDataForFigure EV2/EV2E/SW1116-HO-WT.tif]

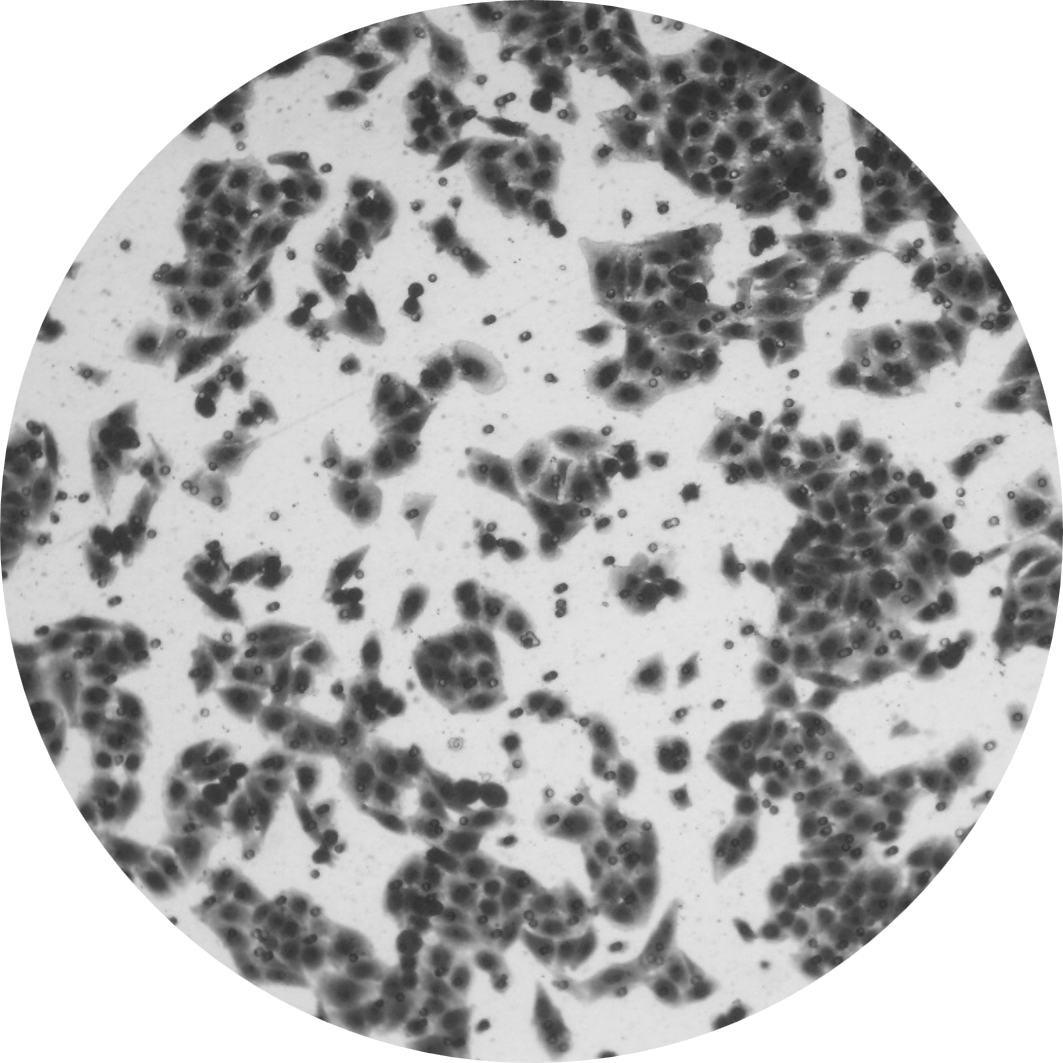

Supplement: Supplementary file 15 — Figure EV1-5 Source Data [file 44318_2025_416_MOESM15_ESM.zip › EMBOJ-2024-119243R_SourceDataForExpandedView/EMBOJ-2024-119243R_SourceDataForFigure EV2/EV2E/SW1116-LO-ED.tif]

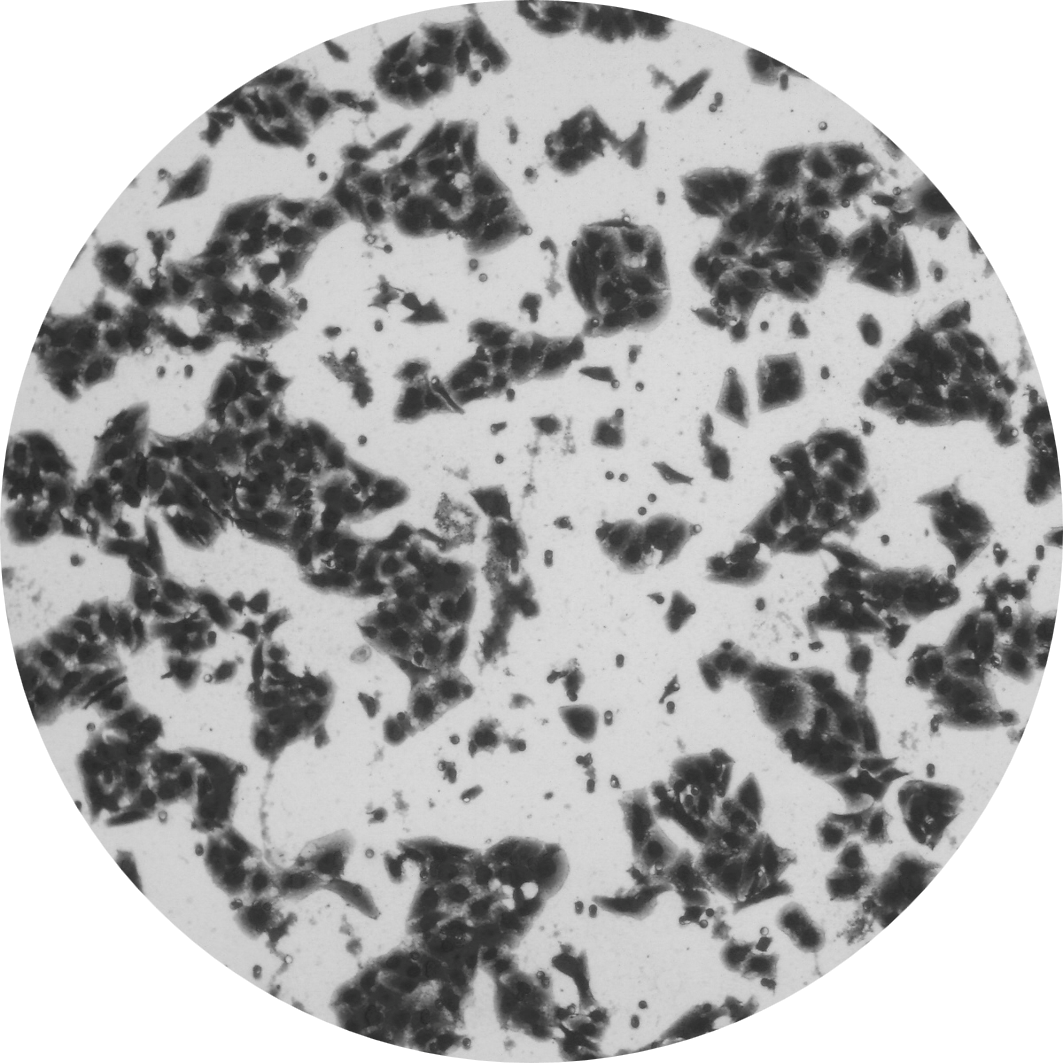

Supplement: Supplementary file 15 — Figure EV1-5 Source Data [file 44318_2025_416_MOESM15_ESM.zip › EMBOJ-2024-119243R_SourceDataForExpandedView/EMBOJ-2024-119243R_SourceDataForFigure EV2/EV2E/SW1116-LO-shPPA2.tif]

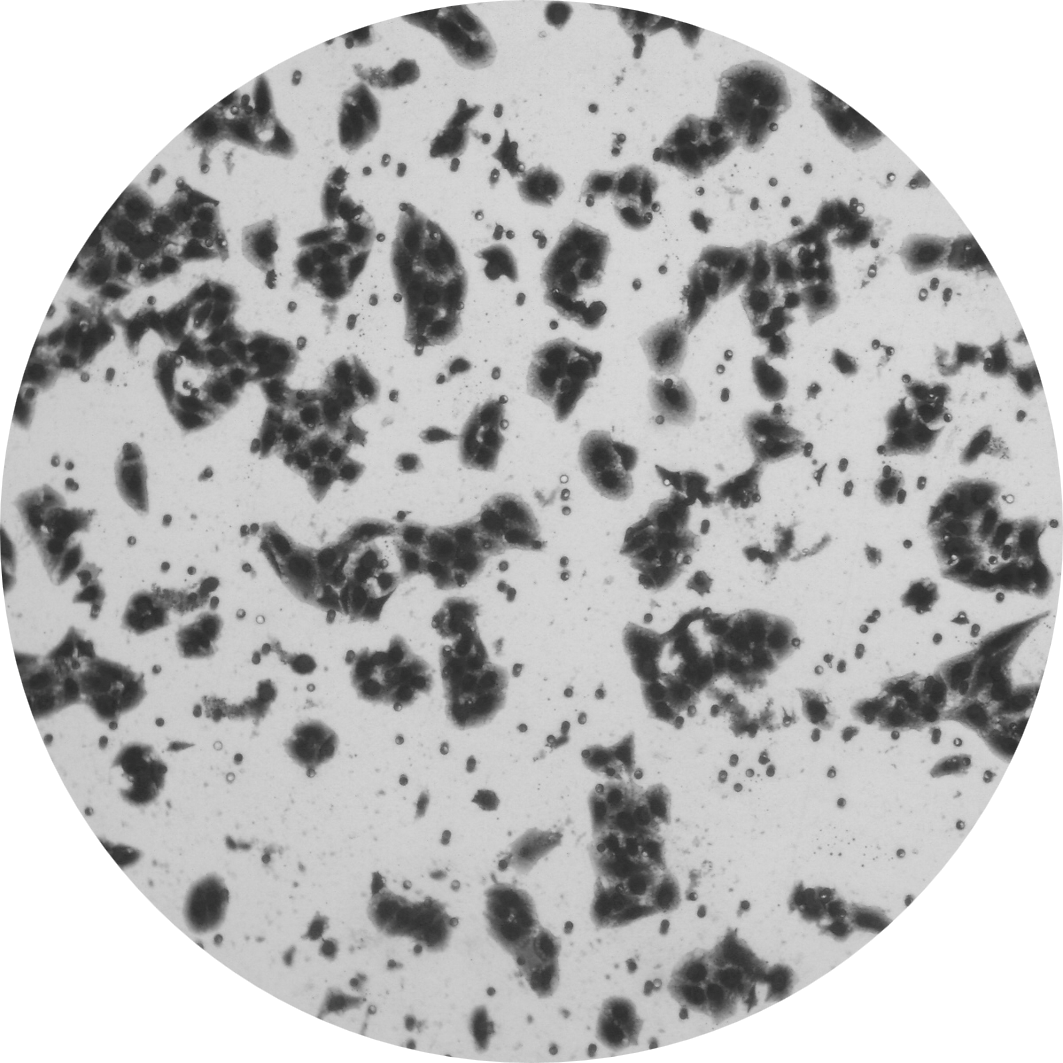

Supplement: Supplementary file 15 — Figure EV1-5 Source Data [file 44318_2025_416_MOESM15_ESM.zip › EMBOJ-2024-119243R_SourceDataForExpandedView/EMBOJ-2024-119243R_SourceDataForFigure EV2/EV2E/SW1116-LO-WT.tif]

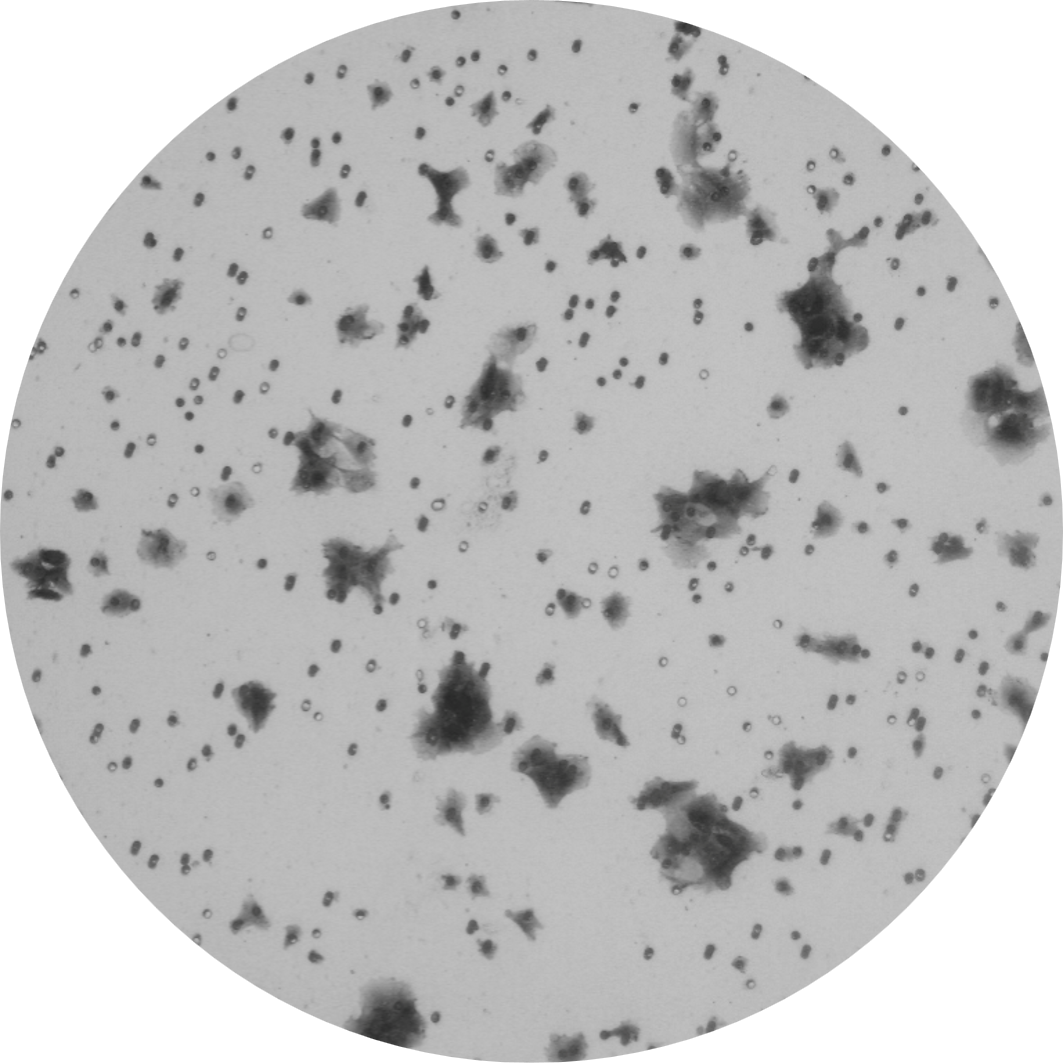

Supplement: Supplementary file 15 — Figure EV1-5 Source Data [file 44318_2025_416_MOESM15_ESM.zip › EMBOJ-2024-119243R_SourceDataForExpandedView/EMBOJ-2024-119243R_SourceDataForFigure EV2/EV2G/DLD1-HO-ED.tif]

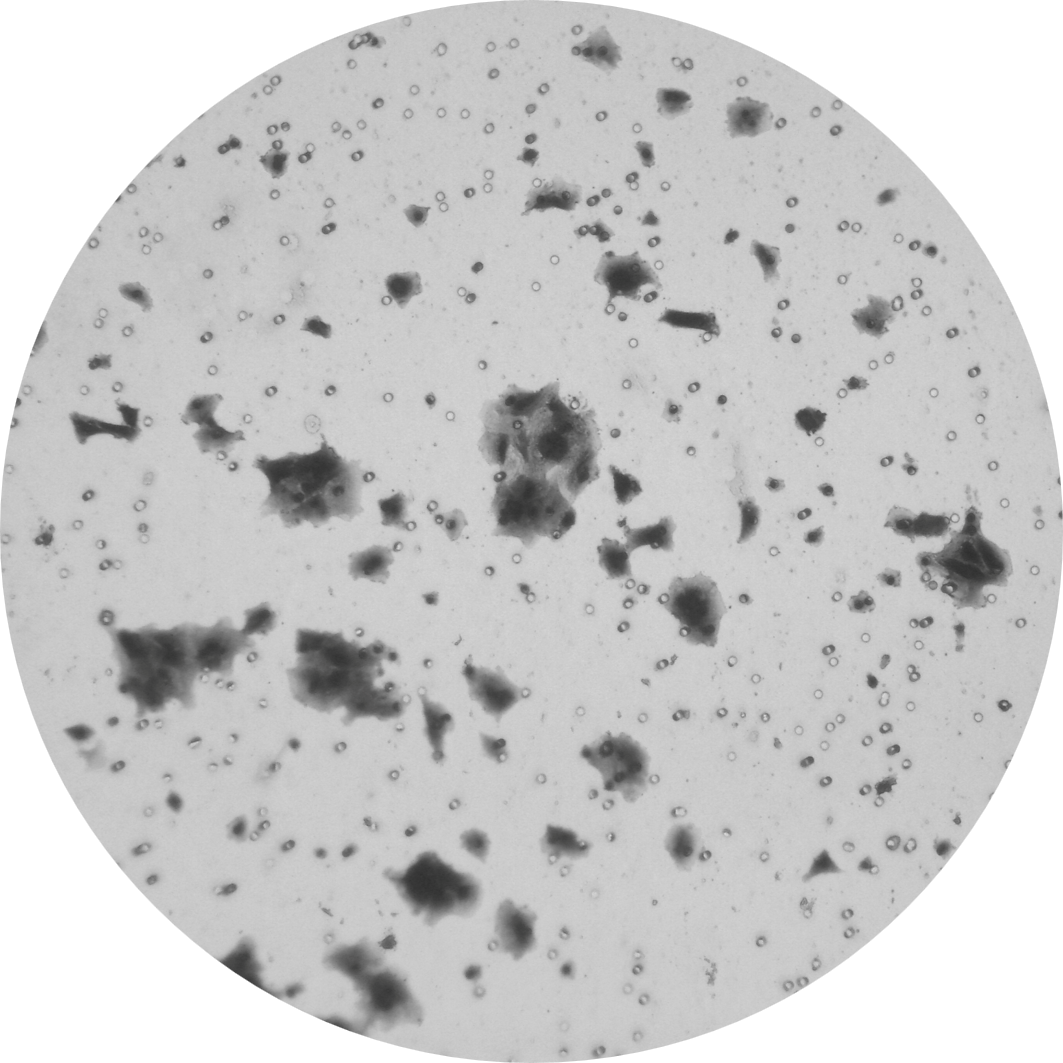

Supplement: Supplementary file 15 — Figure EV1-5 Source Data [file 44318_2025_416_MOESM15_ESM.zip › EMBOJ-2024-119243R_SourceDataForExpandedView/EMBOJ-2024-119243R_SourceDataForFigure EV2/EV2G/DLD1-HO-shPPA2.tif]

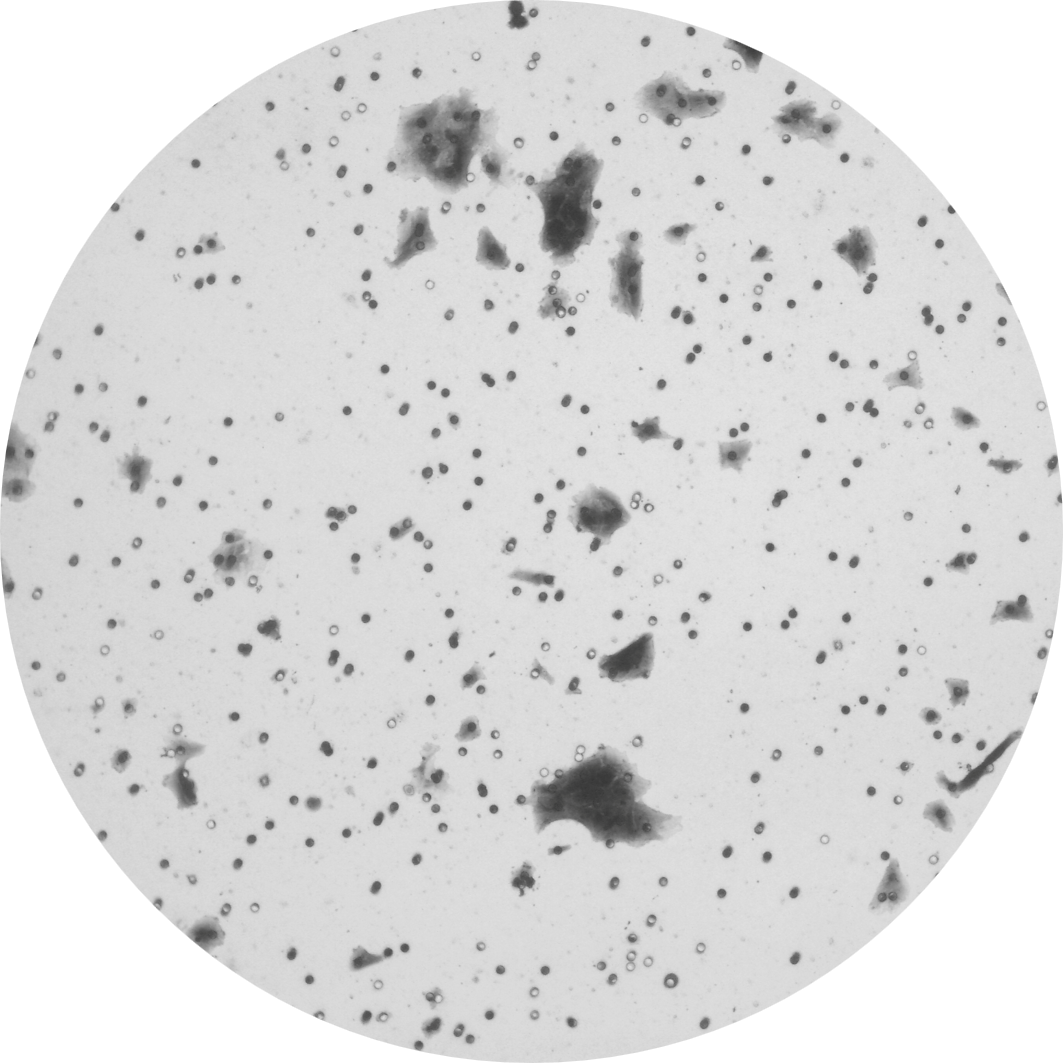

Supplement: Supplementary file 15 — Figure EV1-5 Source Data [file 44318_2025_416_MOESM15_ESM.zip › EMBOJ-2024-119243R_SourceDataForExpandedView/EMBOJ-2024-119243R_SourceDataForFigure EV2/EV2G/DLD1-HO-WT.tif]

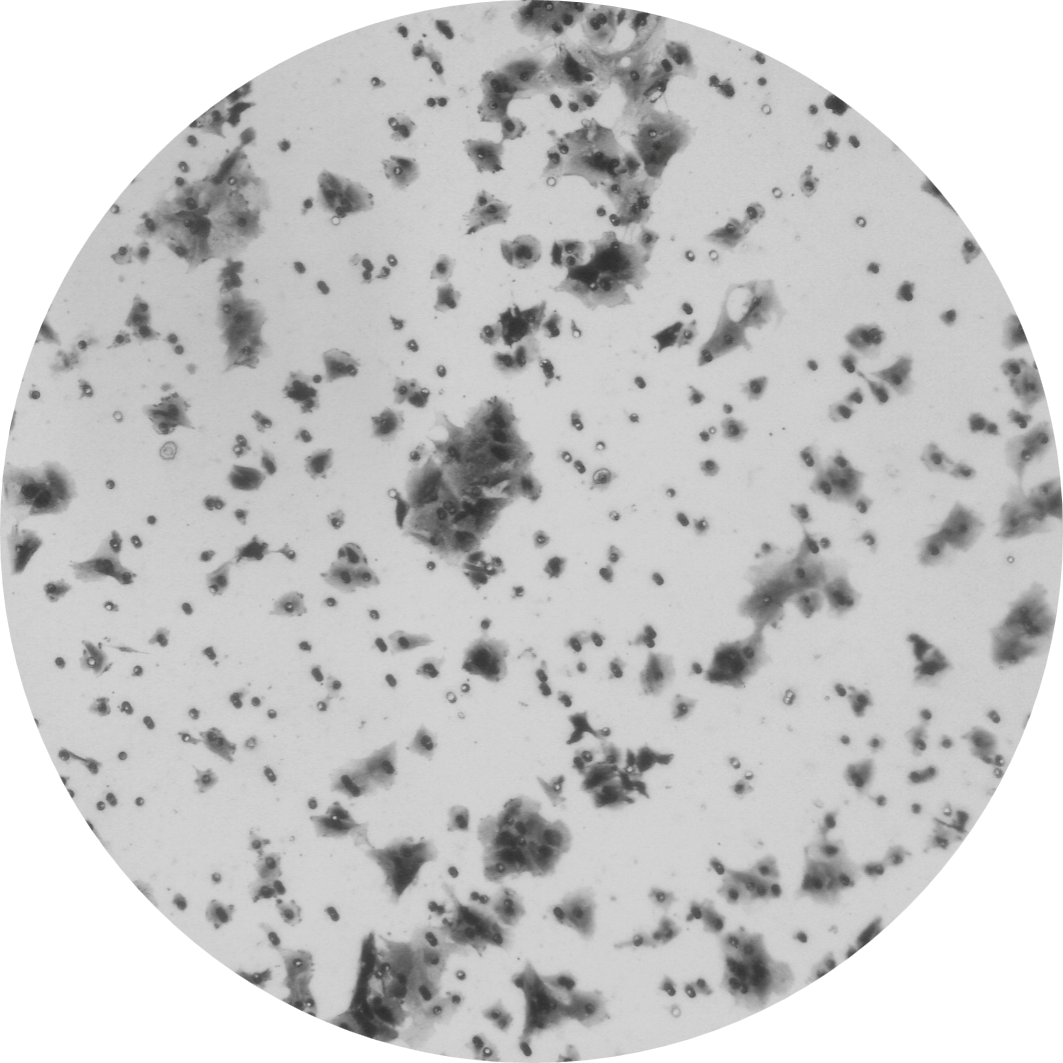

Supplement: Supplementary file 15 — Figure EV1-5 Source Data [file 44318_2025_416_MOESM15_ESM.zip › EMBOJ-2024-119243R_SourceDataForExpandedView/EMBOJ-2024-119243R_SourceDataForFigure EV2/EV2G/DLD1-LO-ED.tif]

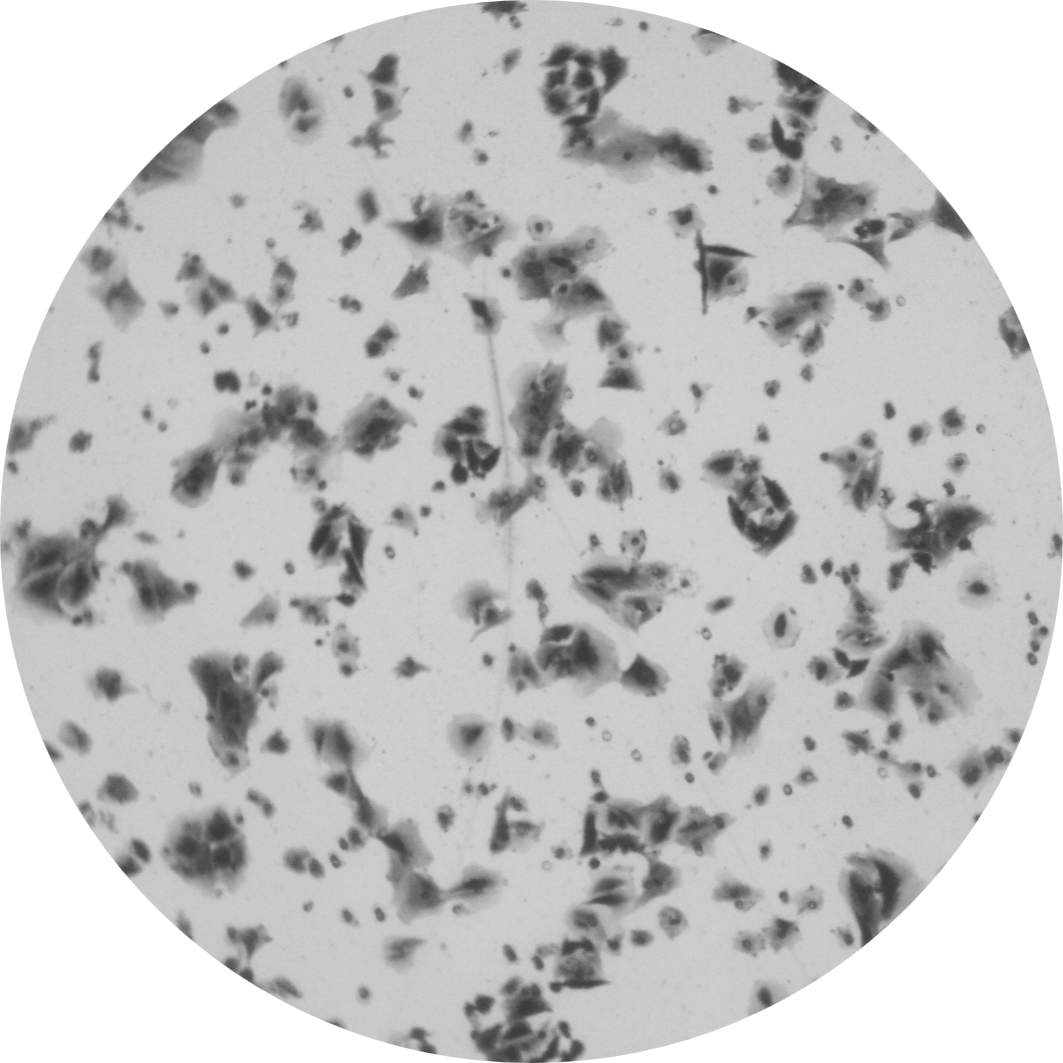

Supplement: Supplementary file 15 — Figure EV1-5 Source Data [file 44318_2025_416_MOESM15_ESM.zip › EMBOJ-2024-119243R_SourceDataForExpandedView/EMBOJ-2024-119243R_SourceDataForFigure EV2/EV2G/DLD1-LO-shPPA2.tif]

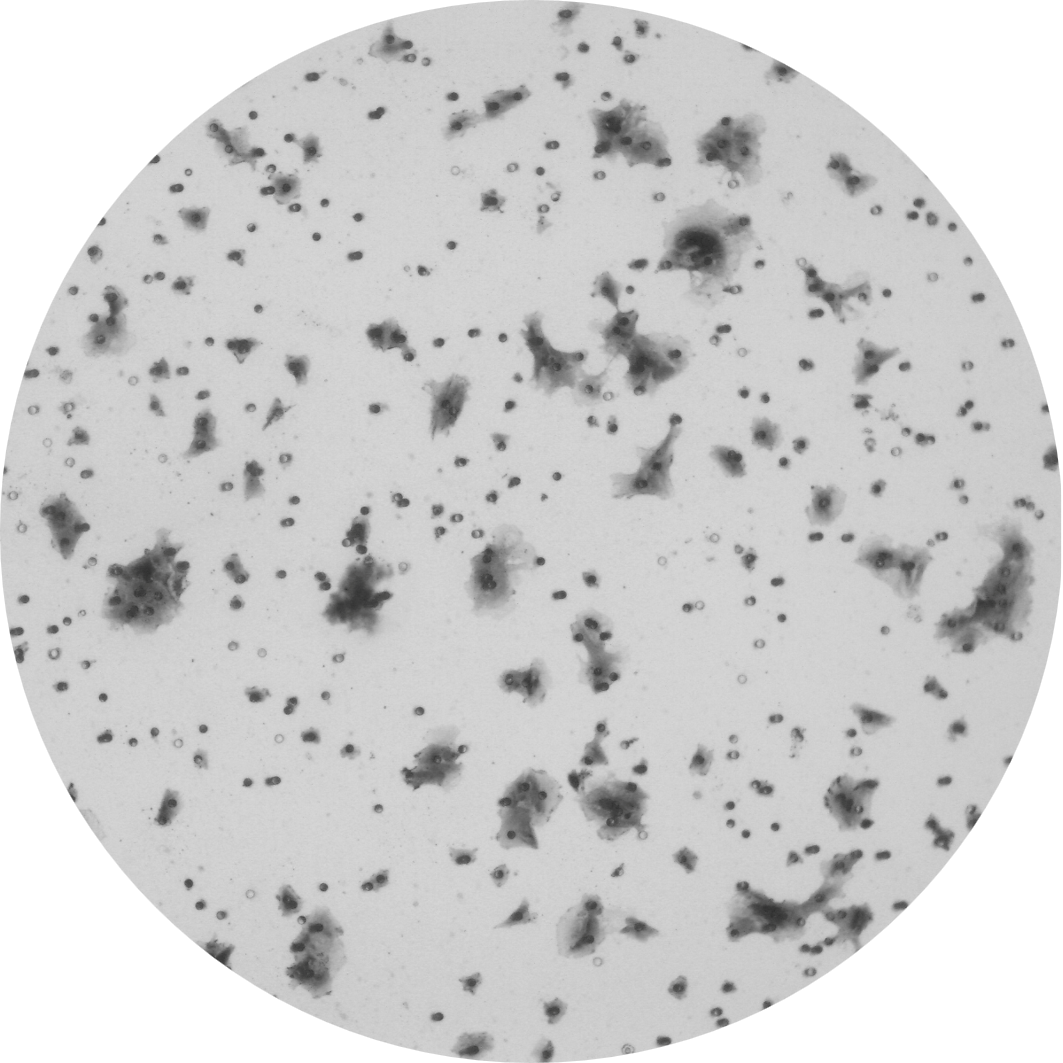

Supplement: Supplementary file 15 — Figure EV1-5 Source Data [file 44318_2025_416_MOESM15_ESM.zip › EMBOJ-2024-119243R_SourceDataForExpandedView/EMBOJ-2024-119243R_SourceDataForFigure EV2/EV2G/DLD1-LO-WT.tif]

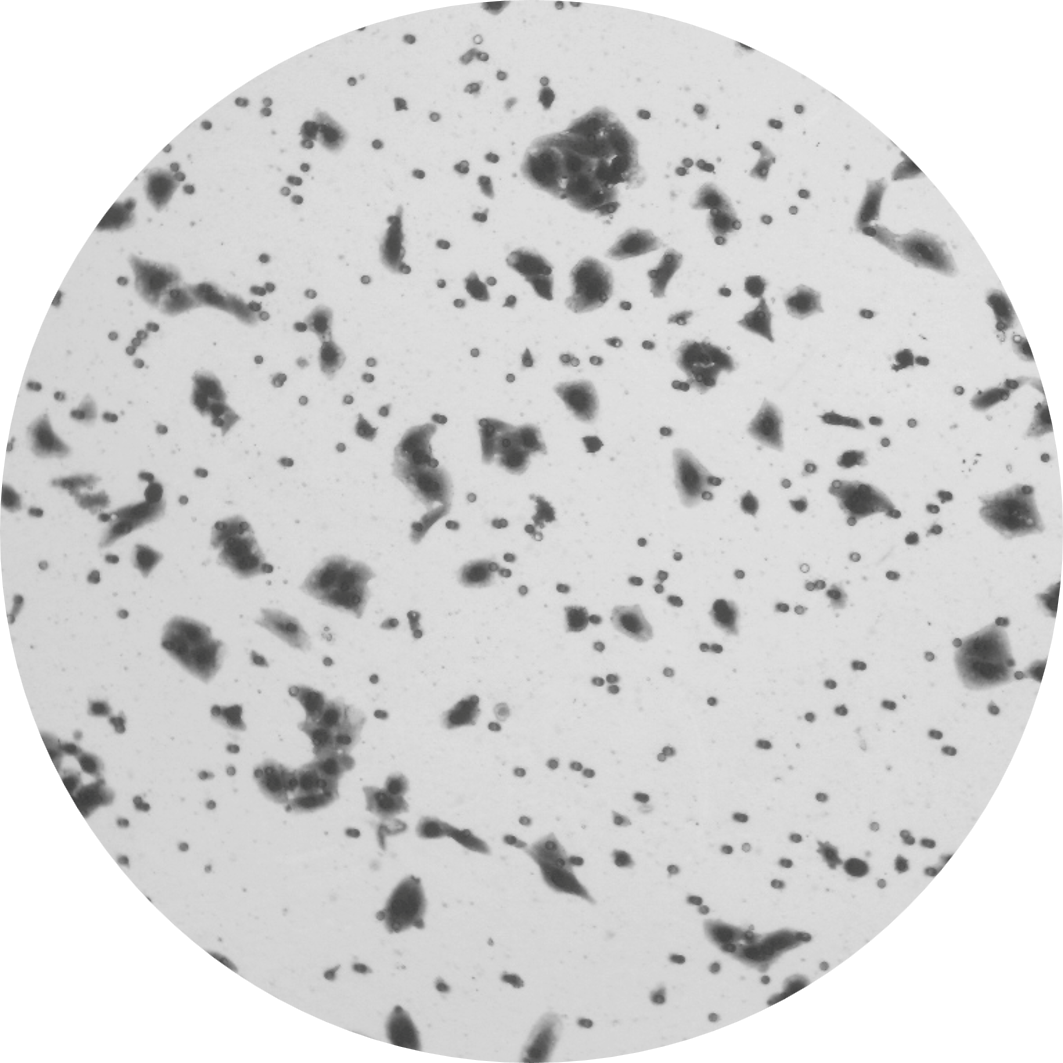

Supplement: Supplementary file 15 — Figure EV1-5 Source Data [file 44318_2025_416_MOESM15_ESM.zip › EMBOJ-2024-119243R_SourceDataForExpandedView/EMBOJ-2024-119243R_SourceDataForFigure EV2/EV2G/SW1116-HO-ED.tif]

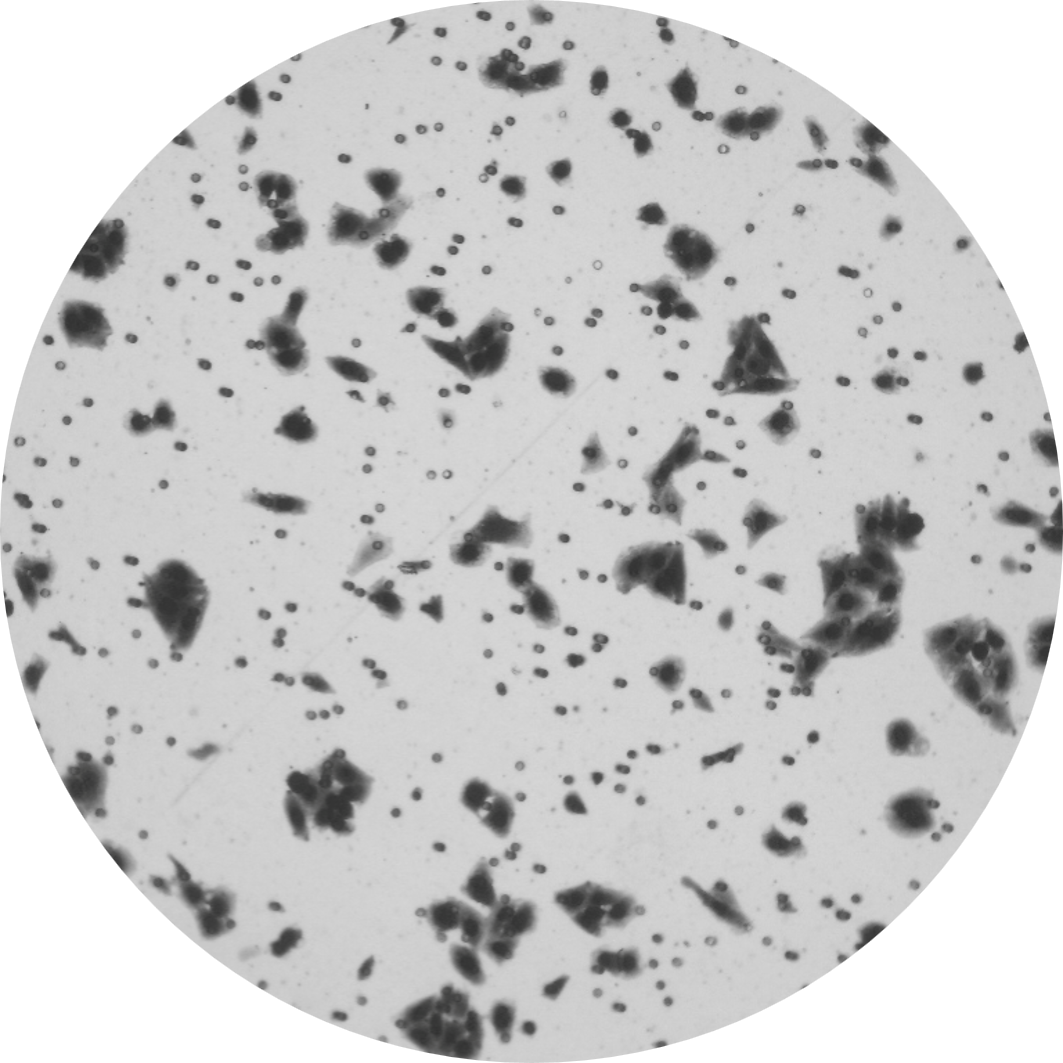

Supplement: Supplementary file 15 — Figure EV1-5 Source Data [file 44318_2025_416_MOESM15_ESM.zip › EMBOJ-2024-119243R_SourceDataForExpandedView/EMBOJ-2024-119243R_SourceDataForFigure EV2/EV2G/SW1116-HO-shPPA2.tif]

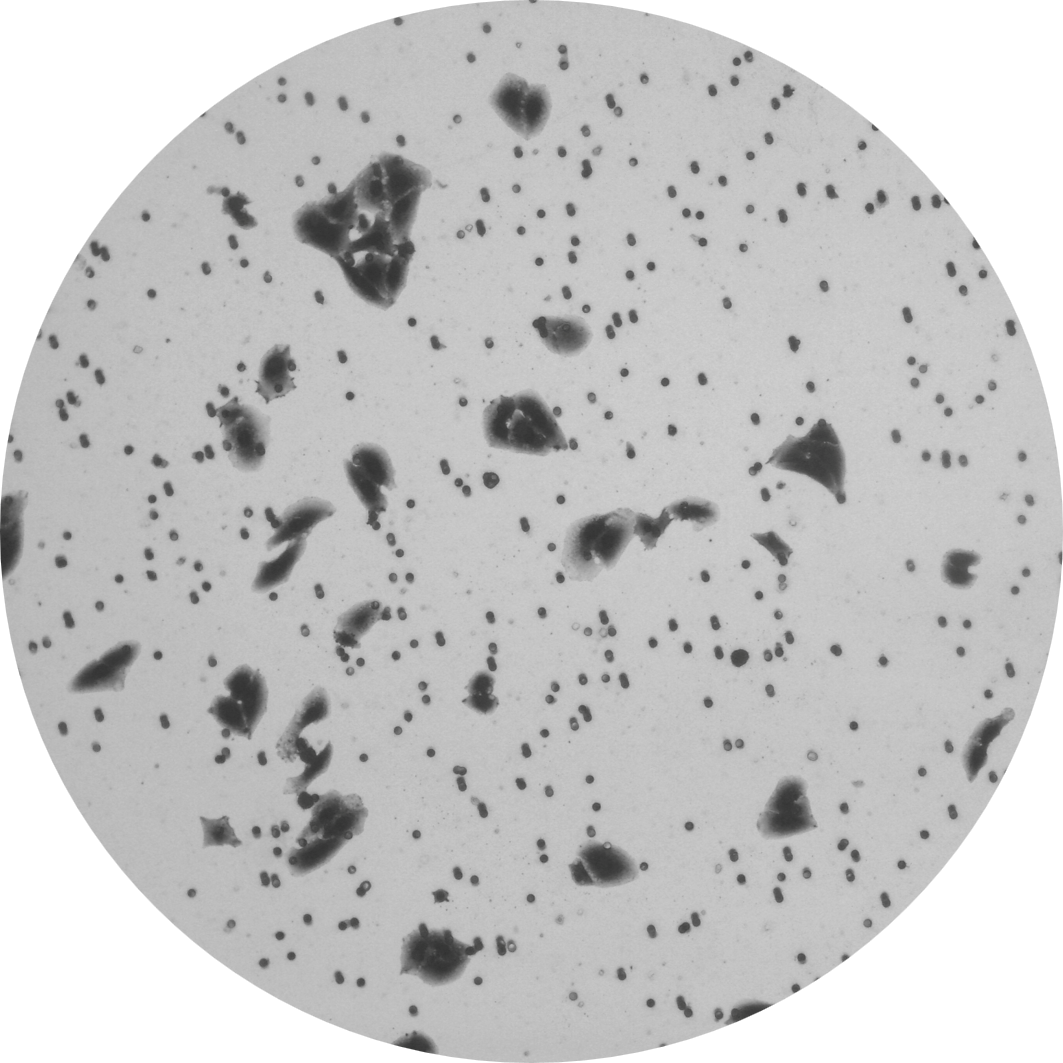

Supplement: Supplementary file 15 — Figure EV1-5 Source Data [file 44318_2025_416_MOESM15_ESM.zip › EMBOJ-2024-119243R_SourceDataForExpandedView/EMBOJ-2024-119243R_SourceDataForFigure EV2/EV2G/SW1116-HO-WT.tif]

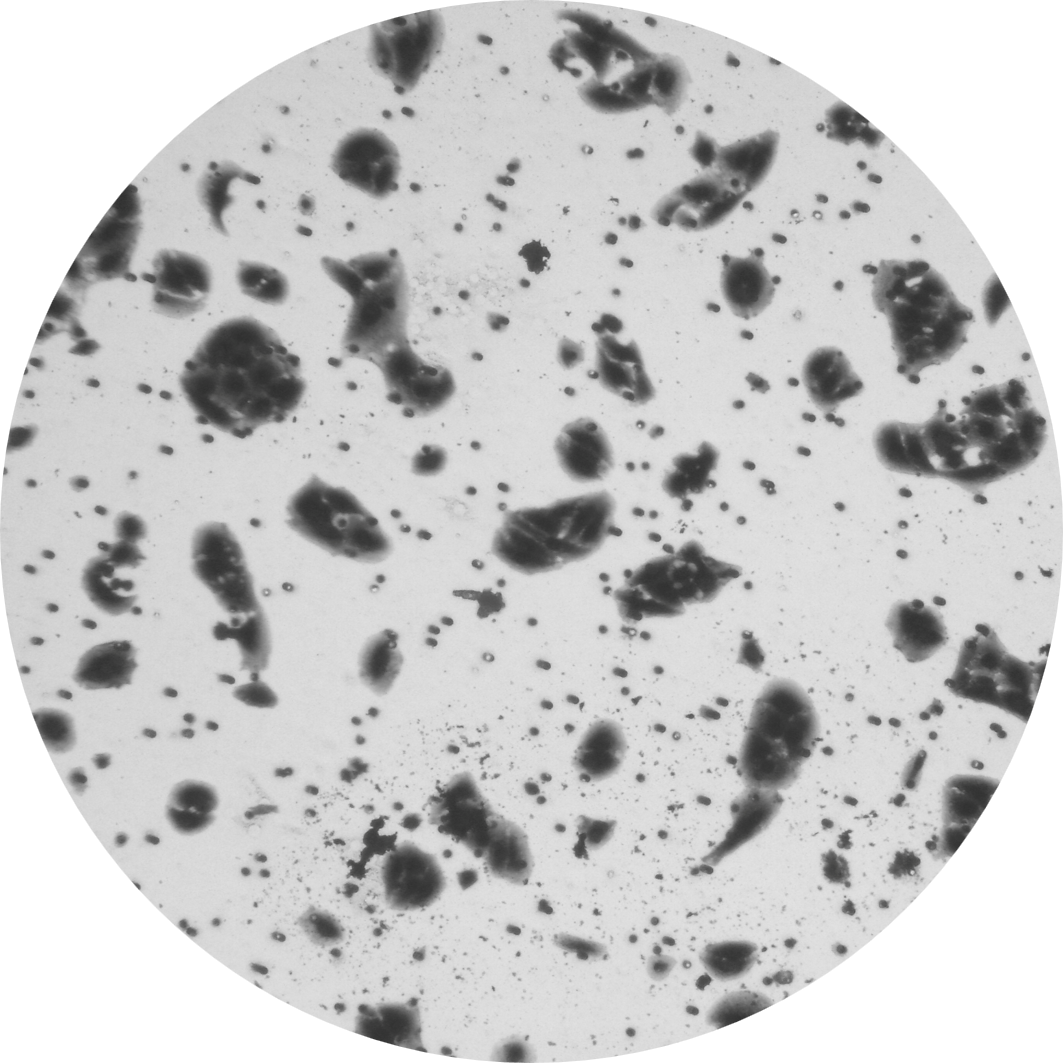

Supplement: Supplementary file 15 — Figure EV1-5 Source Data [file 44318_2025_416_MOESM15_ESM.zip › EMBOJ-2024-119243R_SourceDataForExpandedView/EMBOJ-2024-119243R_SourceDataForFigure EV2/EV2G/SW1116-LO-ED.tif]

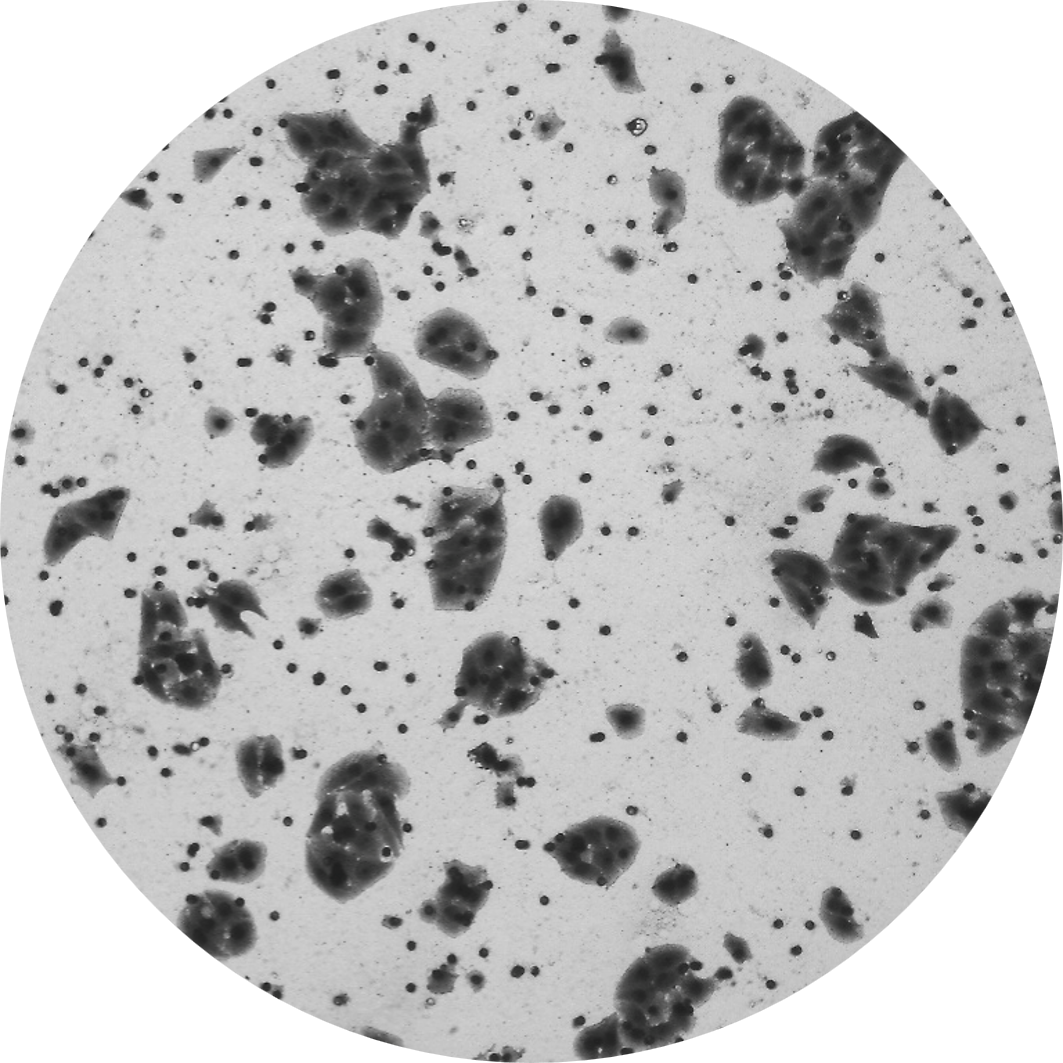

Supplement: Supplementary file 15 — Figure EV1-5 Source Data [file 44318_2025_416_MOESM15_ESM.zip › EMBOJ-2024-119243R_SourceDataForExpandedView/EMBOJ-2024-119243R_SourceDataForFigure EV2/EV2G/SW1116-LO-shPPA2.tif]

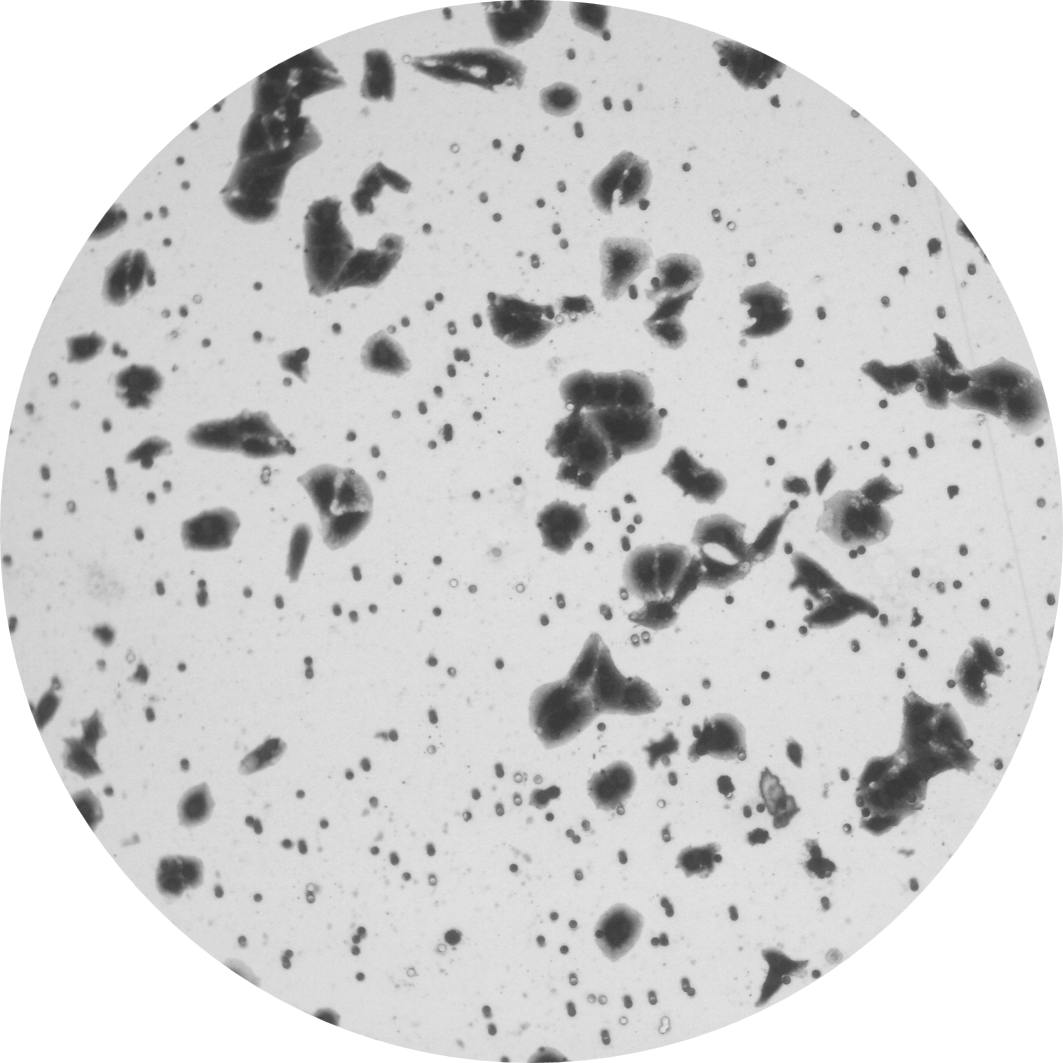

Supplement: Supplementary file 15 — Figure EV1-5 Source Data [file 44318_2025_416_MOESM15_ESM.zip › EMBOJ-2024-119243R_SourceDataForExpandedView/EMBOJ-2024-119243R_SourceDataForFigure EV2/EV2G/SW1116-LO-WT.tif]

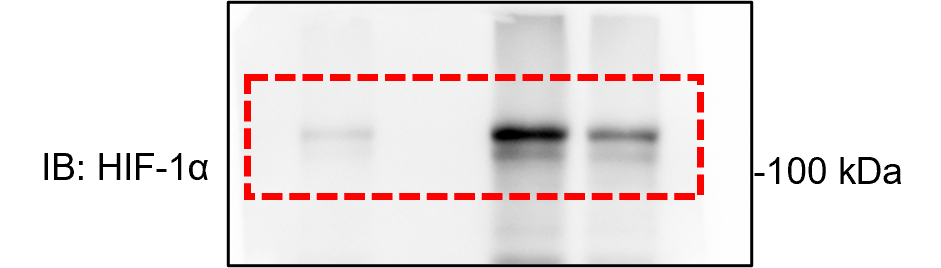

Supplement: Supplementary file 15 — Figure EV1-5 Source Data [file 44318_2025_416_MOESM15_ESM.zip › EMBOJ-2024-119243R_SourceDataForExpandedView/EMBOJ-2024-119243R_SourceDataForFigure EV3/EV3I/DLD1-HIF-1α.tif]

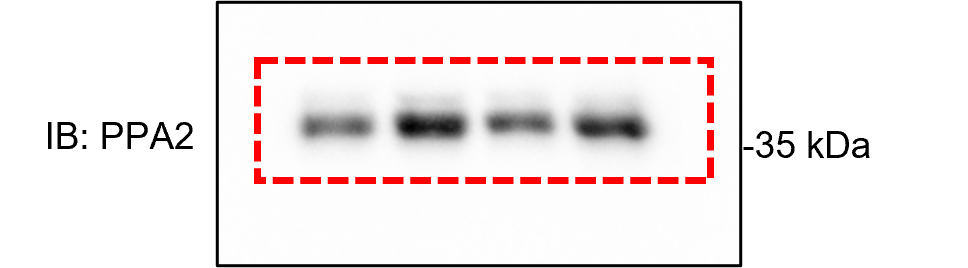

Supplement: Supplementary file 15 — Figure EV1-5 Source Data [file 44318_2025_416_MOESM15_ESM.zip › EMBOJ-2024-119243R_SourceDataForExpandedView/EMBOJ-2024-119243R_SourceDataForFigure EV3/EV3I/DLD1-PPA2.tif]

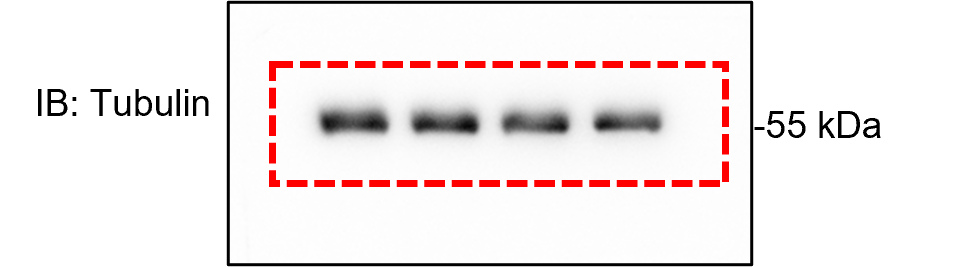

Supplement: Supplementary file 15 — Figure EV1-5 Source Data [file 44318_2025_416_MOESM15_ESM.zip › EMBOJ-2024-119243R_SourceDataForExpandedView/EMBOJ-2024-119243R_SourceDataForFigure EV3/EV3I/DLD1-Tubulin.tif]

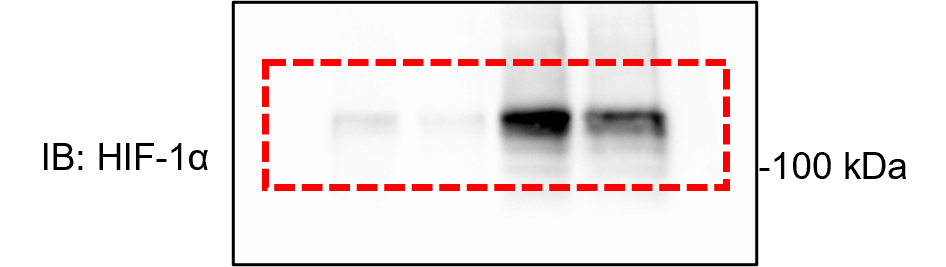

Supplement: Supplementary file 15 — Figure EV1-5 Source Data [file 44318_2025_416_MOESM15_ESM.zip › EMBOJ-2024-119243R_SourceDataForExpandedView/EMBOJ-2024-119243R_SourceDataForFigure EV3/EV3I/SW1116-HIF-1α.tif]

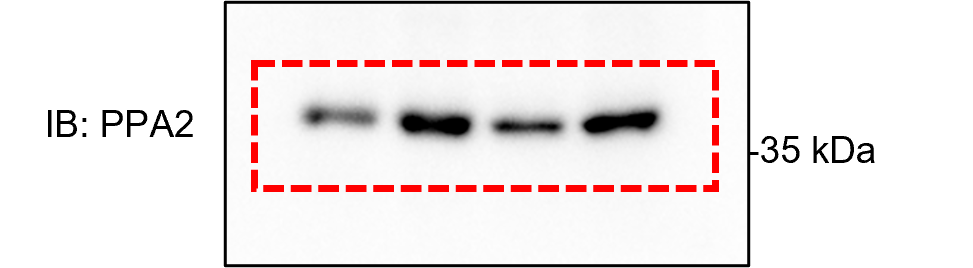

Supplement: Supplementary file 15 — Figure EV1-5 Source Data [file 44318_2025_416_MOESM15_ESM.zip › EMBOJ-2024-119243R_SourceDataForExpandedView/EMBOJ-2024-119243R_SourceDataForFigure EV3/EV3I/SW1116-PPA2.tif]

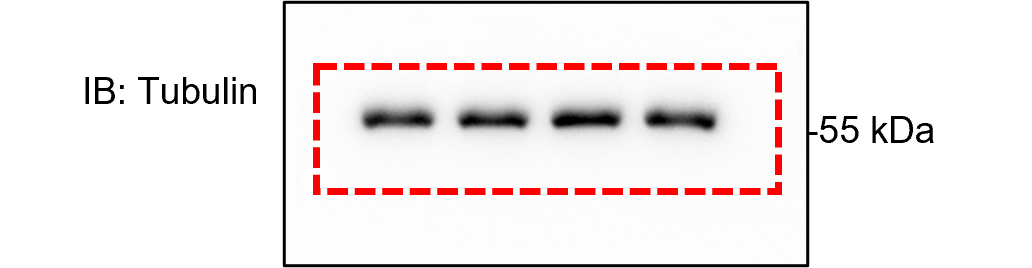

Supplement: Supplementary file 15 — Figure EV1-5 Source Data [file 44318_2025_416_MOESM15_ESM.zip › EMBOJ-2024-119243R_SourceDataForExpandedView/EMBOJ-2024-119243R_SourceDataForFigure EV3/EV3I/SW1116-Tubulin.tif]

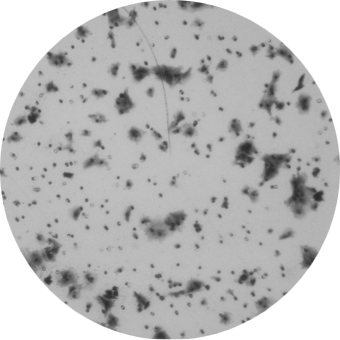

Supplement: Supplementary file 15 — Figure EV1-5 Source Data [file 44318_2025_416_MOESM15_ESM.zip › EMBOJ-2024-119243R_SourceDataForExpandedView/EMBOJ-2024-119243R_SourceDataForFigure EV3/EV3O/DLD1-sgCtrl-shNT.tif]

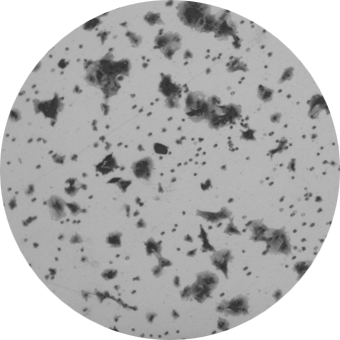

Supplement: Supplementary file 15 — Figure EV1-5 Source Data [file 44318_2025_416_MOESM15_ESM.zip › EMBOJ-2024-119243R_SourceDataForExpandedView/EMBOJ-2024-119243R_SourceDataForFigure EV3/EV3O/DLD1-sgCtrl-shPPA2.tif]

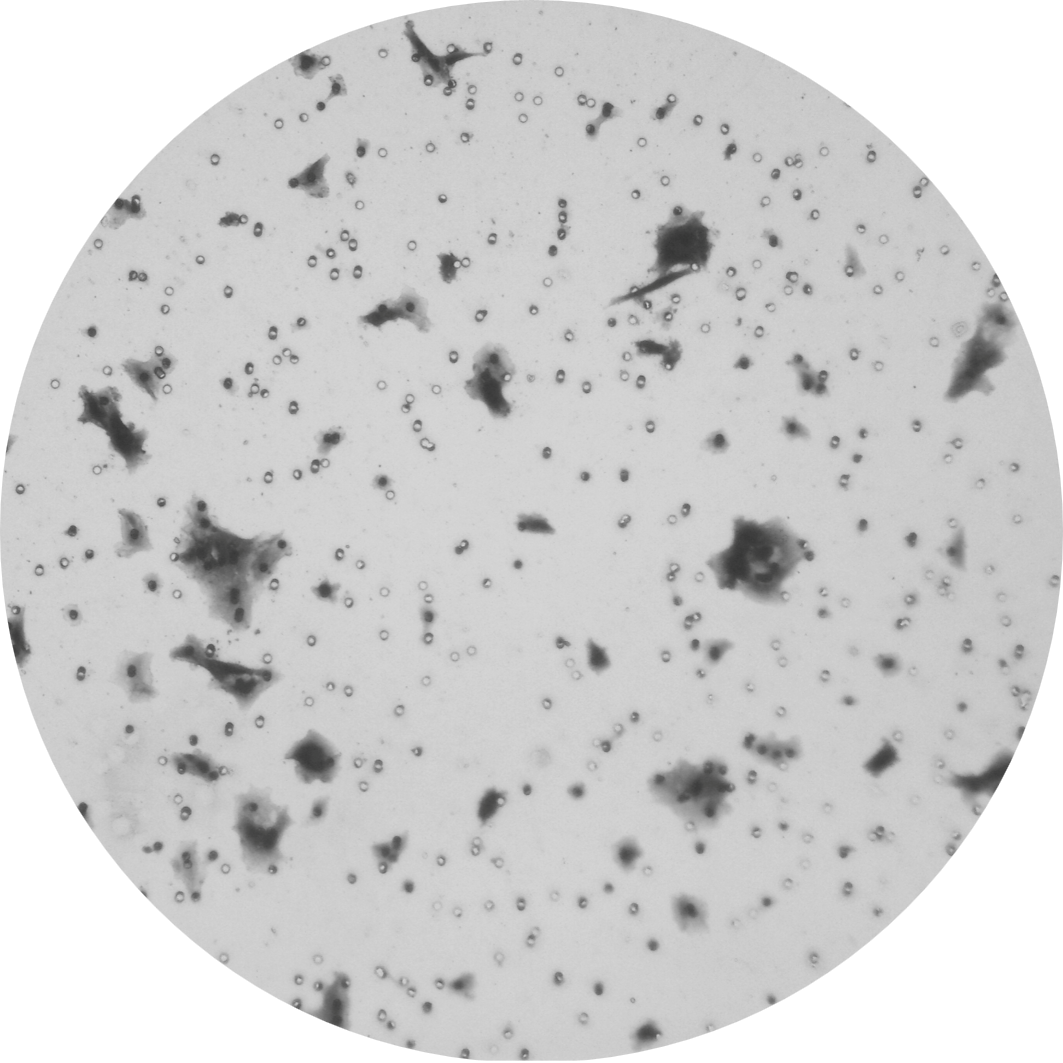

Supplement: Supplementary file 15 — Figure EV1-5 Source Data [file 44318_2025_416_MOESM15_ESM.zip › EMBOJ-2024-119243R_SourceDataForExpandedView/EMBOJ-2024-119243R_SourceDataForFigure EV3/EV3O/DLD1-sgHIF1a-shNT.tif]

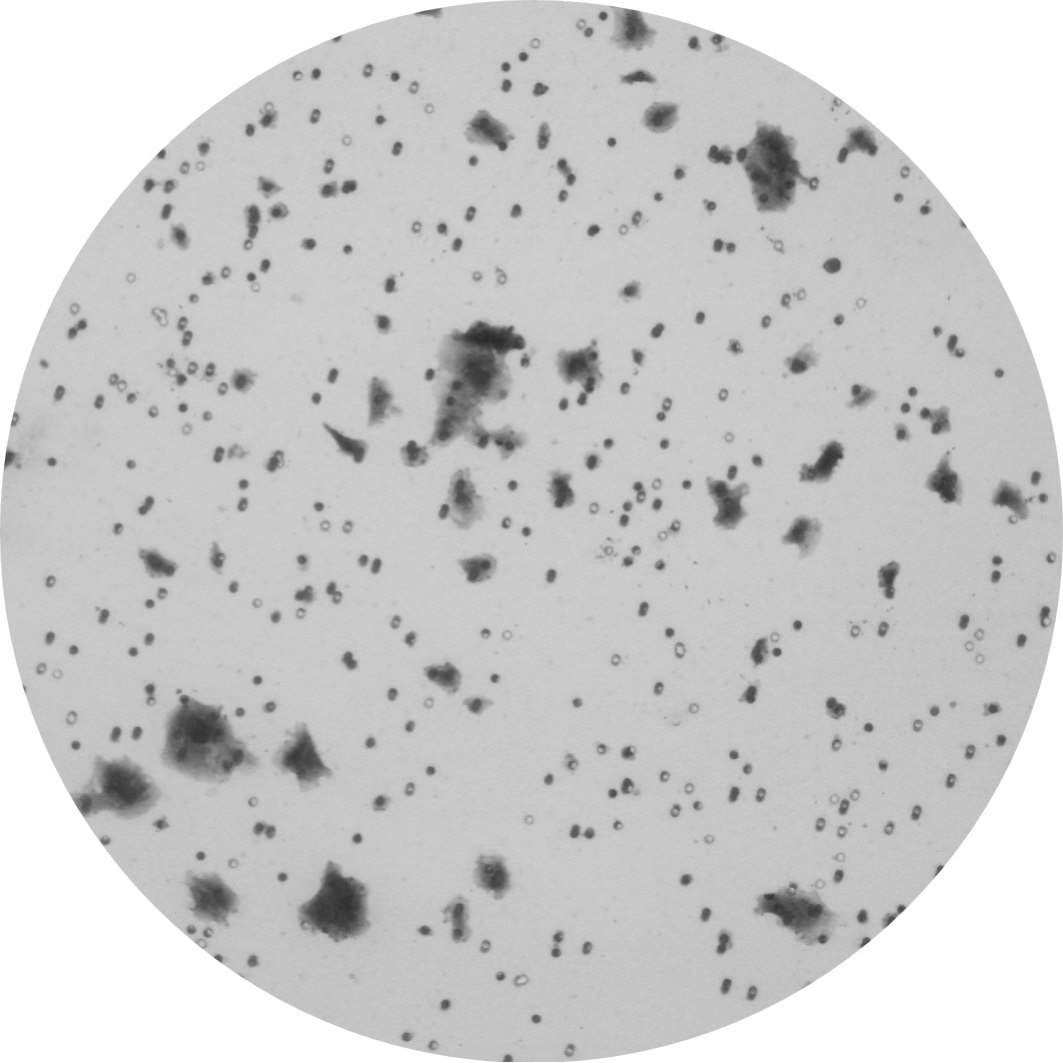

Supplement: Supplementary file 15 — Figure EV1-5 Source Data [file 44318_2025_416_MOESM15_ESM.zip › EMBOJ-2024-119243R_SourceDataForExpandedView/EMBOJ-2024-119243R_SourceDataForFigure EV3/EV3O/DLD1-sgHIF1a-shPPA2.tif]

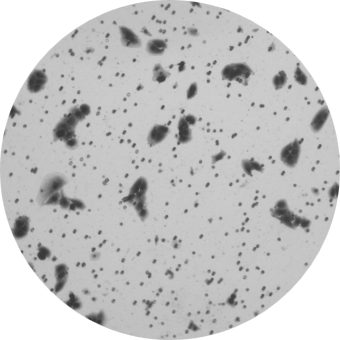

Supplement: Supplementary file 15 — Figure EV1-5 Source Data [file 44318_2025_416_MOESM15_ESM.zip › EMBOJ-2024-119243R_SourceDataForExpandedView/EMBOJ-2024-119243R_SourceDataForFigure EV3/EV3O/SW1116-sgCtrl-shNT.tif]

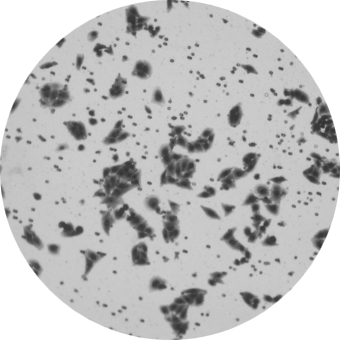

Supplement: Supplementary file 15 — Figure EV1-5 Source Data [file 44318_2025_416_MOESM15_ESM.zip › EMBOJ-2024-119243R_SourceDataForExpandedView/EMBOJ-2024-119243R_SourceDataForFigure EV3/EV3O/SW1116-sgCtrl-shPPA2.tif]

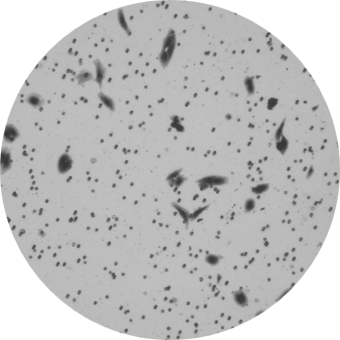

Supplement: Supplementary file 15 — Figure EV1-5 Source Data [file 44318_2025_416_MOESM15_ESM.zip › EMBOJ-2024-119243R_SourceDataForExpandedView/EMBOJ-2024-119243R_SourceDataForFigure EV3/EV3O/SW1116-sgHIF1a-shNT.tif]

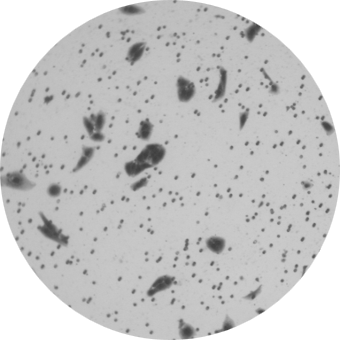

Supplement: Supplementary file 15 — Figure EV1-5 Source Data [file 44318_2025_416_MOESM15_ESM.zip › EMBOJ-2024-119243R_SourceDataForExpandedView/EMBOJ-2024-119243R_SourceDataForFigure EV3/EV3O/SW1116-sgHIF1a-shPPA2.tif]

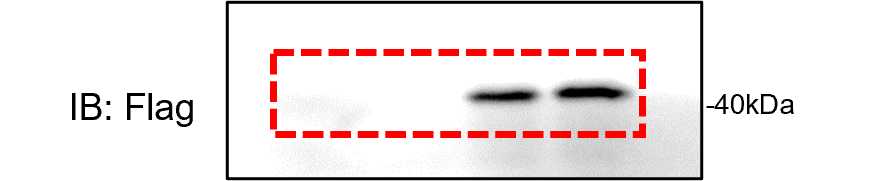

Supplement: Supplementary file 15 — Figure EV1-5 Source Data [file 44318_2025_416_MOESM15_ESM.zip › EMBOJ-2024-119243R_SourceDataForExpandedView/EMBOJ-2024-119243R_SourceDataForFigure EV4/EV4A/Input-Flag.tif]

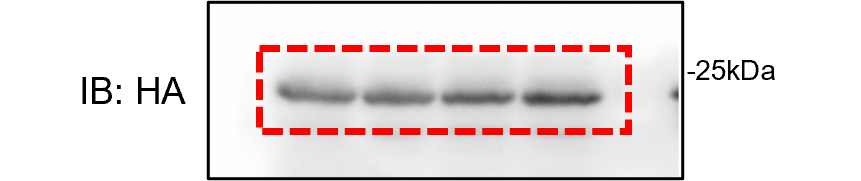

Supplement: Supplementary file 15 — Figure EV1-5 Source Data [file 44318_2025_416_MOESM15_ESM.zip › EMBOJ-2024-119243R_SourceDataForExpandedView/EMBOJ-2024-119243R_SourceDataForFigure EV4/EV4A/Input-HA.tif]

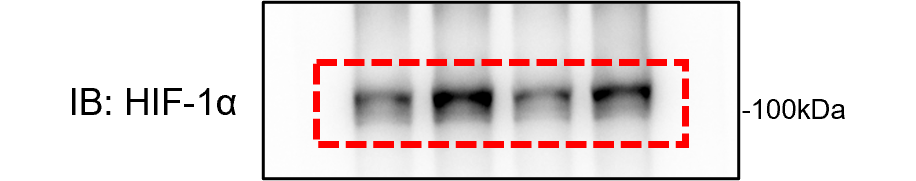

Supplement: Supplementary file 15 — Figure EV1-5 Source Data [file 44318_2025_416_MOESM15_ESM.zip › EMBOJ-2024-119243R_SourceDataForExpandedView/EMBOJ-2024-119243R_SourceDataForFigure EV4/EV4A/Input-HIF-1α.tif]

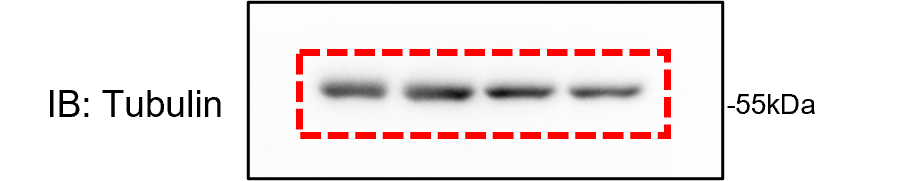

Supplement: Supplementary file 15 — Figure EV1-5 Source Data [file 44318_2025_416_MOESM15_ESM.zip › EMBOJ-2024-119243R_SourceDataForExpandedView/EMBOJ-2024-119243R_SourceDataForFigure EV4/EV4A/Input-Tubulin.tif]

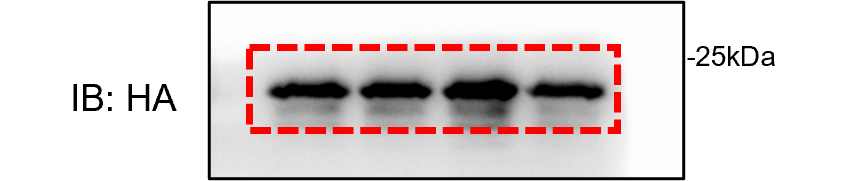

Supplement: Supplementary file 15 — Figure EV1-5 Source Data [file 44318_2025_416_MOESM15_ESM.zip › EMBOJ-2024-119243R_SourceDataForExpandedView/EMBOJ-2024-119243R_SourceDataForFigure EV4/EV4A/IP-HA.tif]

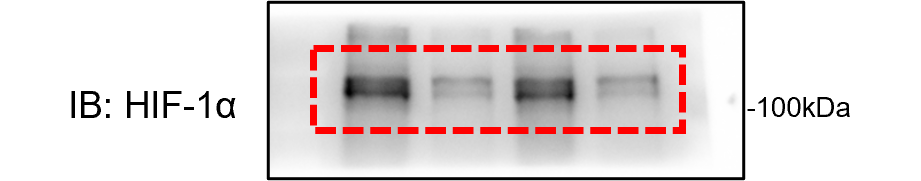

Supplement: Supplementary file 15 — Figure EV1-5 Source Data [file 44318_2025_416_MOESM15_ESM.zip › EMBOJ-2024-119243R_SourceDataForExpandedView/EMBOJ-2024-119243R_SourceDataForFigure EV4/EV4A/IP-HIF-1α.tif]

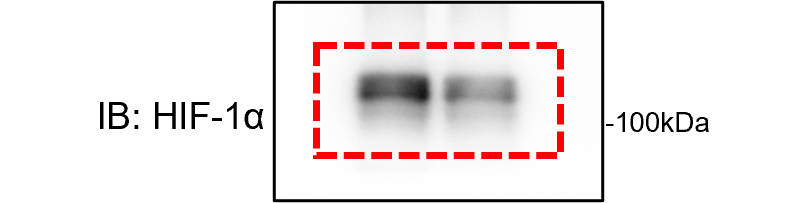

Supplement: Supplementary file 15 — Figure EV1-5 Source Data [file 44318_2025_416_MOESM15_ESM.zip › EMBOJ-2024-119243R_SourceDataForExpandedView/EMBOJ-2024-119243R_SourceDataForFigure EV4/EV4B/PPA2 OE-HIF-1α.tif]

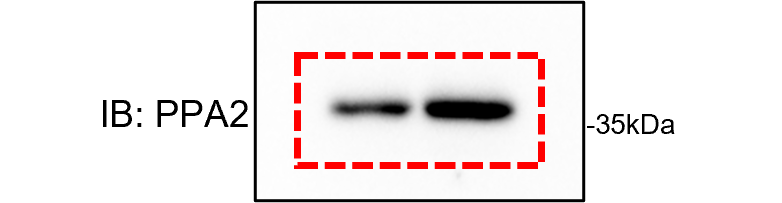

Supplement: Supplementary file 15 — Figure EV1-5 Source Data [file 44318_2025_416_MOESM15_ESM.zip › EMBOJ-2024-119243R_SourceDataForExpandedView/EMBOJ-2024-119243R_SourceDataForFigure EV4/EV4B/PPA2 OE-PPA2.tif]

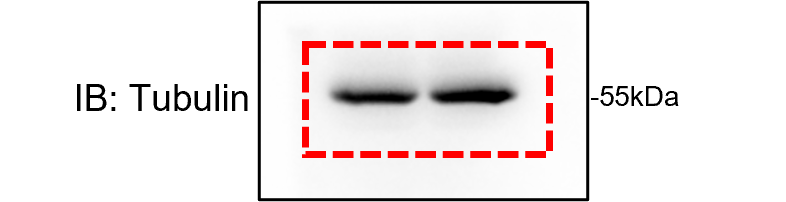

Supplement: Supplementary file 15 — Figure EV1-5 Source Data [file 44318_2025_416_MOESM15_ESM.zip › EMBOJ-2024-119243R_SourceDataForExpandedView/EMBOJ-2024-119243R_SourceDataForFigure EV4/EV4B/PPA2 OE-Tubulin.tif]

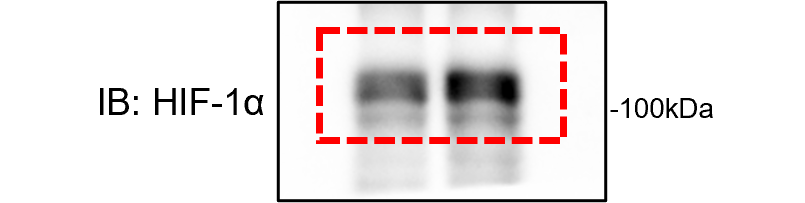

Supplement: Supplementary file 15 — Figure EV1-5 Source Data [file 44318_2025_416_MOESM15_ESM.zip › EMBOJ-2024-119243R_SourceDataForExpandedView/EMBOJ-2024-119243R_SourceDataForFigure EV4/EV4B/shPPA2-HIF-1α.tif]

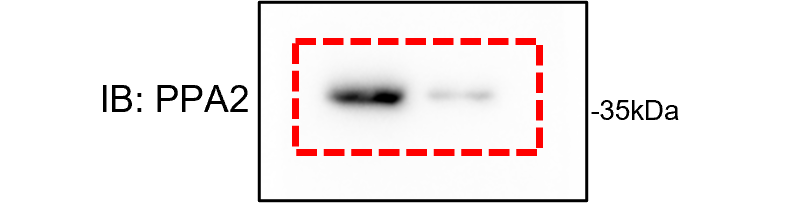

Supplement: Supplementary file 15 — Figure EV1-5 Source Data [file 44318_2025_416_MOESM15_ESM.zip › EMBOJ-2024-119243R_SourceDataForExpandedView/EMBOJ-2024-119243R_SourceDataForFigure EV4/EV4B/shPPA2-PPA2.tif]

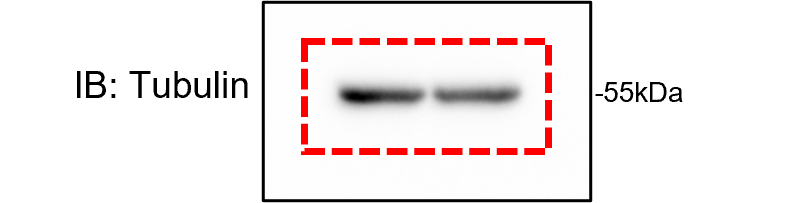

Supplement: Supplementary file 15 — Figure EV1-5 Source Data [file 44318_2025_416_MOESM15_ESM.zip › EMBOJ-2024-119243R_SourceDataForExpandedView/EMBOJ-2024-119243R_SourceDataForFigure EV4/EV4B/shPPA2-Tubulin.tif]

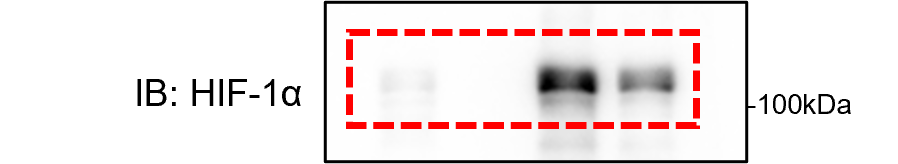

Supplement: Supplementary file 15 — Figure EV1-5 Source Data [file 44318_2025_416_MOESM15_ESM.zip › EMBOJ-2024-119243R_SourceDataForExpandedView/EMBOJ-2024-119243R_SourceDataForFigure EV4/EV4C/HO-HIF-1α.tif]

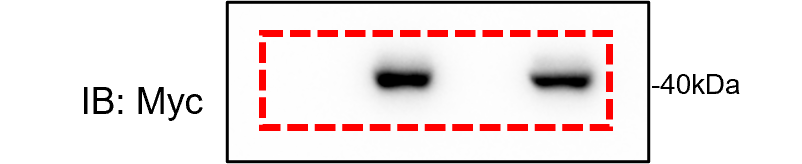

Supplement: Supplementary file 15 — Figure EV1-5 Source Data [file 44318_2025_416_MOESM15_ESM.zip › EMBOJ-2024-119243R_SourceDataForExpandedView/EMBOJ-2024-119243R_SourceDataForFigure EV4/EV4C/HO-Myc.tif]
