# Supplementary figures and images for: SIRT5-mediated desuccinylation of PPA2 enhances HIF-1alpha-dependent adaptation to hypoxic stress and colorectal cancer metastasis (part 5 of 5)
Source: EMBO J. 2025 Mar 31;44(9):2514–40. doi: 10.1038/s44318-025-00416-1 (PMC12048626; doi:10.1038/s44318-025-00416-1)

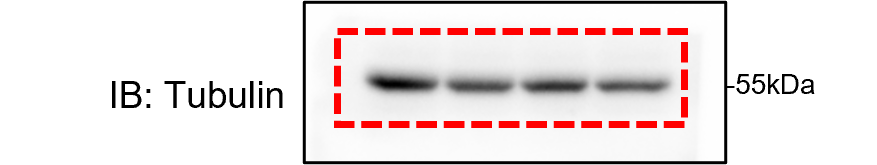

Supplement: Supplementary file 15 — Figure EV1-5 Source Data [file 44318_2025_416_MOESM15_ESM.zip › EMBOJ-2024-119243R_SourceDataForExpandedView/EMBOJ-2024-119243R_SourceDataForFigure EV4/EV4C/HO-Tubulin.tif]

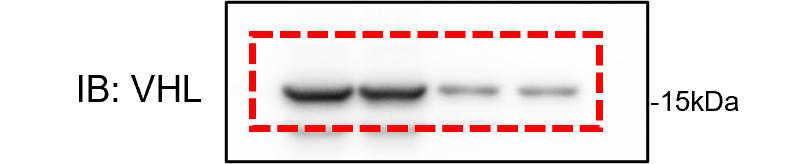

Supplement: Supplementary file 15 — Figure EV1-5 Source Data [file 44318_2025_416_MOESM15_ESM.zip › EMBOJ-2024-119243R_SourceDataForExpandedView/EMBOJ-2024-119243R_SourceDataForFigure EV4/EV4C/HO-VHL.tif]

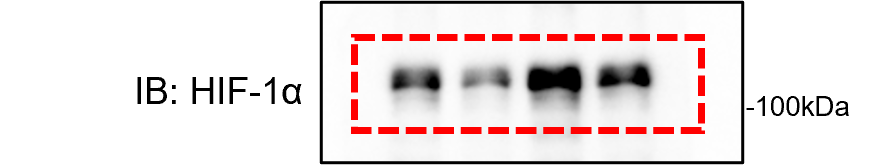

Supplement: Supplementary file 15 — Figure EV1-5 Source Data [file 44318_2025_416_MOESM15_ESM.zip › EMBOJ-2024-119243R_SourceDataForExpandedView/EMBOJ-2024-119243R_SourceDataForFigure EV4/EV4C/LO-HIF-1α.tif]

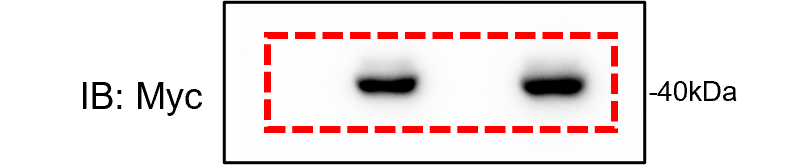

Supplement: Supplementary file 15 — Figure EV1-5 Source Data [file 44318_2025_416_MOESM15_ESM.zip › EMBOJ-2024-119243R_SourceDataForExpandedView/EMBOJ-2024-119243R_SourceDataForFigure EV4/EV4C/LO-Myc.tif]

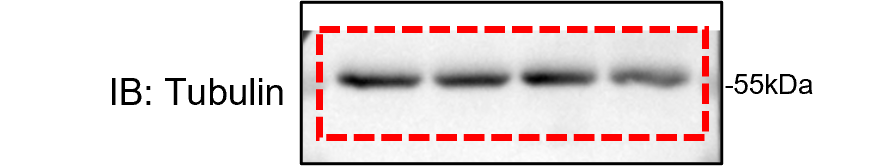

Supplement: Supplementary file 15 — Figure EV1-5 Source Data [file 44318_2025_416_MOESM15_ESM.zip › EMBOJ-2024-119243R_SourceDataForExpandedView/EMBOJ-2024-119243R_SourceDataForFigure EV4/EV4C/LO-Tubulin.tif]

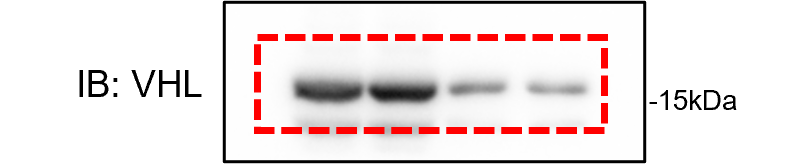

Supplement: Supplementary file 15 — Figure EV1-5 Source Data [file 44318_2025_416_MOESM15_ESM.zip › EMBOJ-2024-119243R_SourceDataForExpandedView/EMBOJ-2024-119243R_SourceDataForFigure EV4/EV4C/LO-VHL.tif]

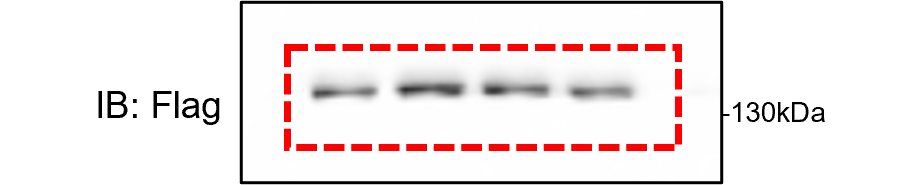

Supplement: Supplementary file 15 — Figure EV1-5 Source Data [file 44318_2025_416_MOESM15_ESM.zip › EMBOJ-2024-119243R_SourceDataForExpandedView/EMBOJ-2024-119243R_SourceDataForFigure EV4/EV4D/HO-Input-Flag.tif]

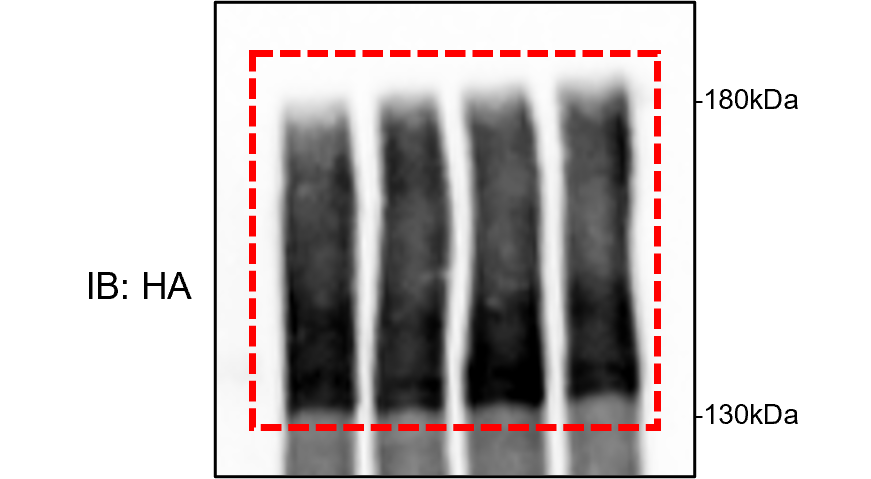

Supplement: Supplementary file 15 — Figure EV1-5 Source Data [file 44318_2025_416_MOESM15_ESM.zip › EMBOJ-2024-119243R_SourceDataForExpandedView/EMBOJ-2024-119243R_SourceDataForFigure EV4/EV4D/HO-Input-HA.tif]

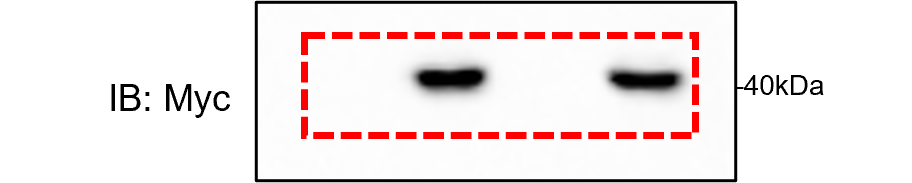

Supplement: Supplementary file 15 — Figure EV1-5 Source Data [file 44318_2025_416_MOESM15_ESM.zip › EMBOJ-2024-119243R_SourceDataForExpandedView/EMBOJ-2024-119243R_SourceDataForFigure EV4/EV4D/HO-Input-Myc.tif]

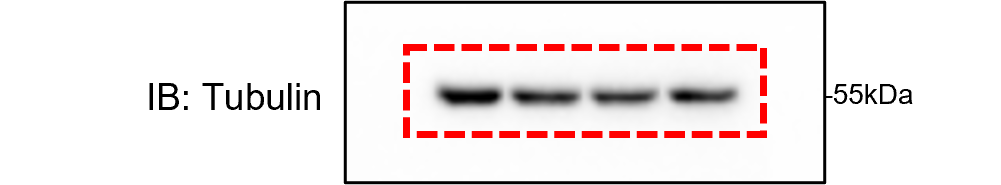

Supplement: Supplementary file 15 — Figure EV1-5 Source Data [file 44318_2025_416_MOESM15_ESM.zip › EMBOJ-2024-119243R_SourceDataForExpandedView/EMBOJ-2024-119243R_SourceDataForFigure EV4/EV4D/HO-Input-Tubulin.tif]

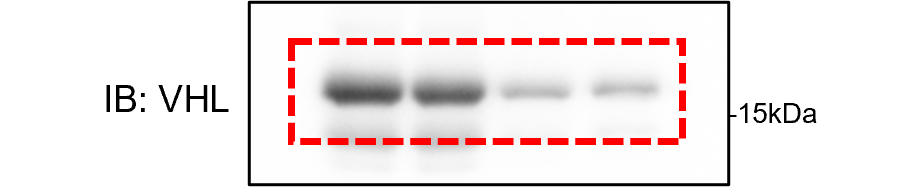

Supplement: Supplementary file 15 — Figure EV1-5 Source Data [file 44318_2025_416_MOESM15_ESM.zip › EMBOJ-2024-119243R_SourceDataForExpandedView/EMBOJ-2024-119243R_SourceDataForFigure EV4/EV4D/HO-Input-VHL.tif]

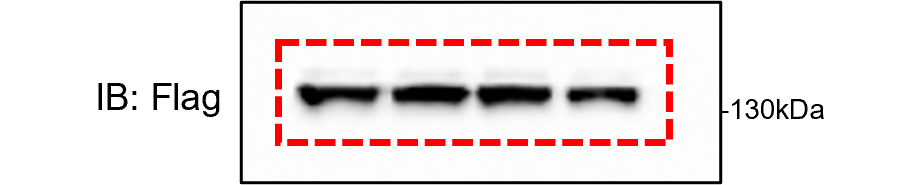

Supplement: Supplementary file 15 — Figure EV1-5 Source Data [file 44318_2025_416_MOESM15_ESM.zip › EMBOJ-2024-119243R_SourceDataForExpandedView/EMBOJ-2024-119243R_SourceDataForFigure EV4/EV4D/HO-IP-Flag.tif]

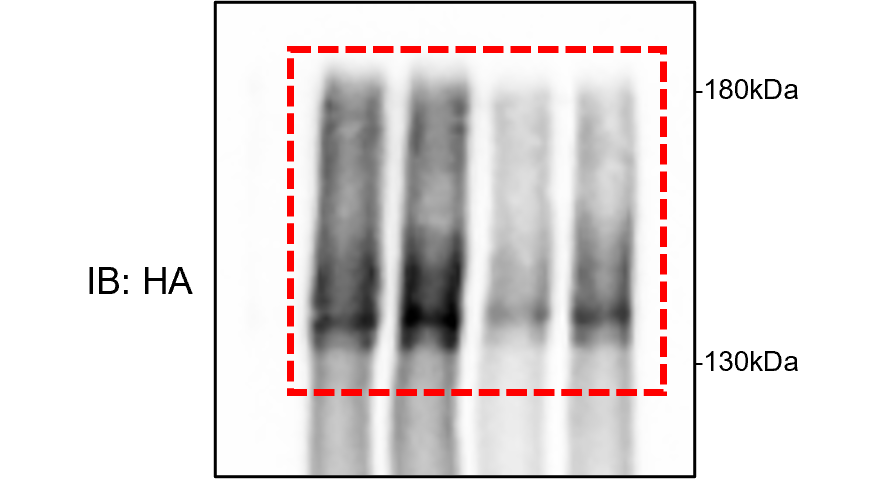

Supplement: Supplementary file 15 — Figure EV1-5 Source Data [file 44318_2025_416_MOESM15_ESM.zip › EMBOJ-2024-119243R_SourceDataForExpandedView/EMBOJ-2024-119243R_SourceDataForFigure EV4/EV4D/HO-IP-HA.tif]

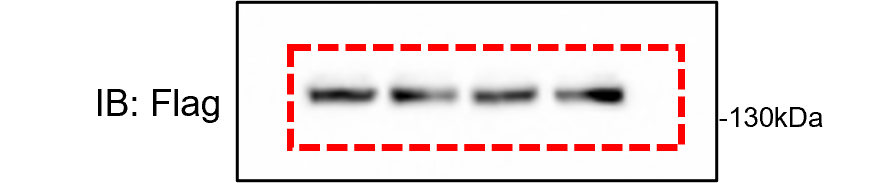

Supplement: Supplementary file 15 — Figure EV1-5 Source Data [file 44318_2025_416_MOESM15_ESM.zip › EMBOJ-2024-119243R_SourceDataForExpandedView/EMBOJ-2024-119243R_SourceDataForFigure EV4/EV4D/LO-Input-Flag.tif]

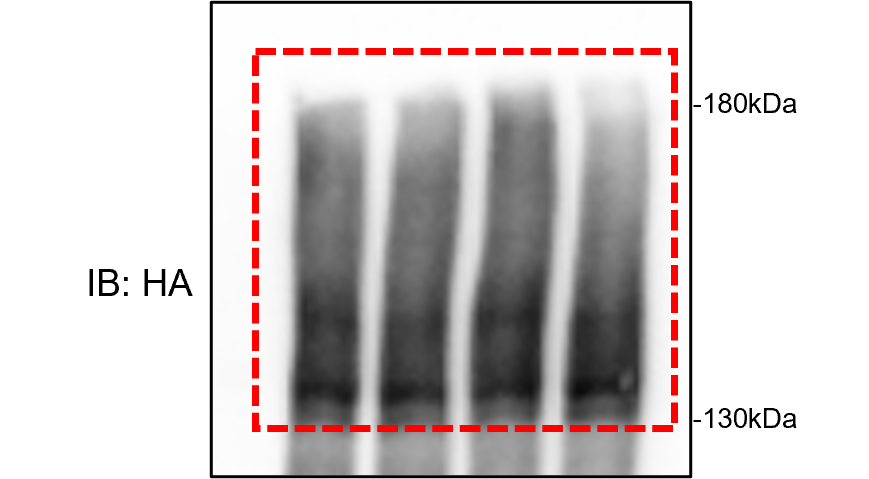

Supplement: Supplementary file 15 — Figure EV1-5 Source Data [file 44318_2025_416_MOESM15_ESM.zip › EMBOJ-2024-119243R_SourceDataForExpandedView/EMBOJ-2024-119243R_SourceDataForFigure EV4/EV4D/LO-Input-HA.tif]

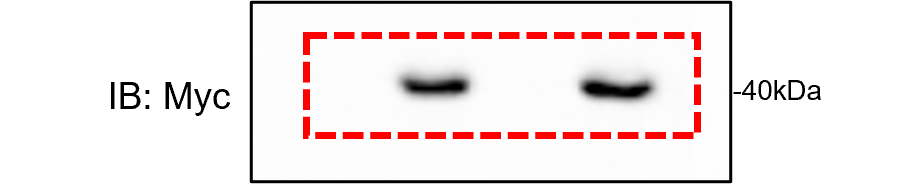

Supplement: Supplementary file 15 — Figure EV1-5 Source Data [file 44318_2025_416_MOESM15_ESM.zip › EMBOJ-2024-119243R_SourceDataForExpandedView/EMBOJ-2024-119243R_SourceDataForFigure EV4/EV4D/LO-Input-Myc.tif]

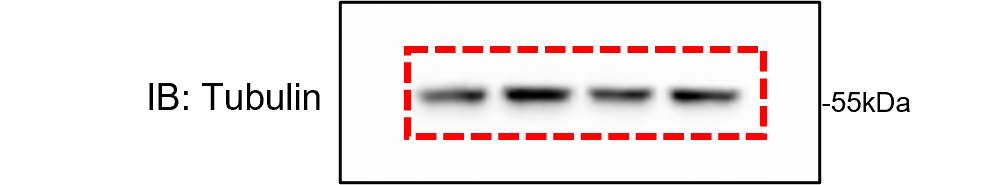

Supplement: Supplementary file 15 — Figure EV1-5 Source Data [file 44318_2025_416_MOESM15_ESM.zip › EMBOJ-2024-119243R_SourceDataForExpandedView/EMBOJ-2024-119243R_SourceDataForFigure EV4/EV4D/LO-Input-Tubulin.tif]

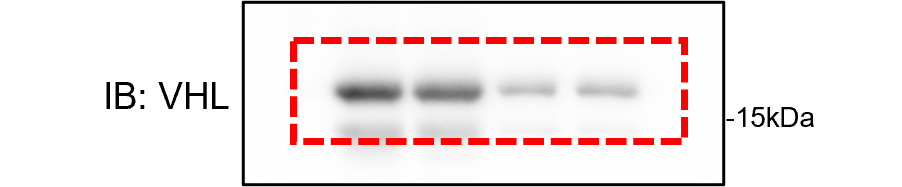

Supplement: Supplementary file 15 — Figure EV1-5 Source Data [file 44318_2025_416_MOESM15_ESM.zip › EMBOJ-2024-119243R_SourceDataForExpandedView/EMBOJ-2024-119243R_SourceDataForFigure EV4/EV4D/LO-Input-VHL.tif]

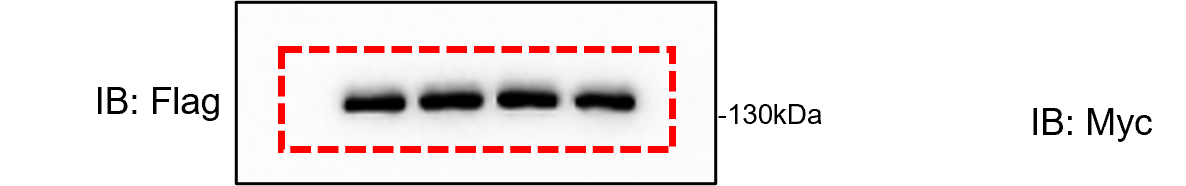

Supplement: Supplementary file 15 — Figure EV1-5 Source Data [file 44318_2025_416_MOESM15_ESM.zip › EMBOJ-2024-119243R_SourceDataForExpandedView/EMBOJ-2024-119243R_SourceDataForFigure EV4/EV4D/LO-IP-Flag.tif]

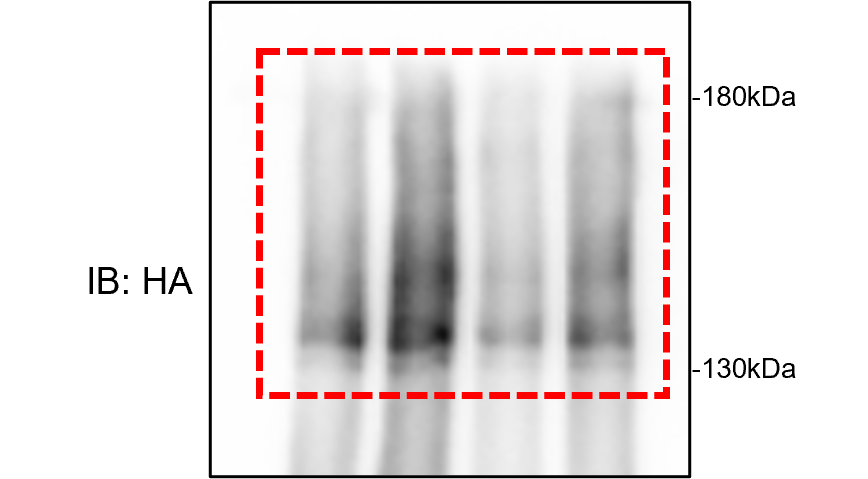

Supplement: Supplementary file 15 — Figure EV1-5 Source Data [file 44318_2025_416_MOESM15_ESM.zip › EMBOJ-2024-119243R_SourceDataForExpandedView/EMBOJ-2024-119243R_SourceDataForFigure EV4/EV4D/LO-IP-HA.tif]

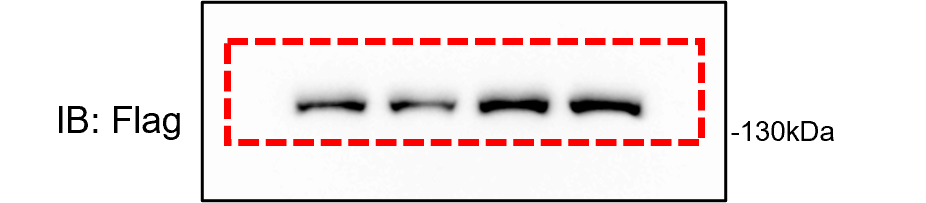

Supplement: Supplementary file 15 — Figure EV1-5 Source Data [file 44318_2025_416_MOESM15_ESM.zip › EMBOJ-2024-119243R_SourceDataForExpandedView/EMBOJ-2024-119243R_SourceDataForFigure EV4/EV4E/Input-Flag.tif]

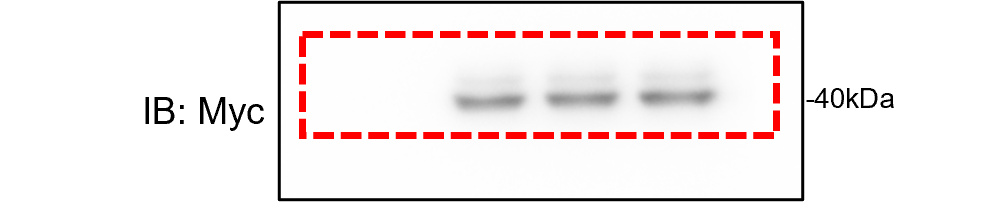

Supplement: Supplementary file 15 — Figure EV1-5 Source Data [file 44318_2025_416_MOESM15_ESM.zip › EMBOJ-2024-119243R_SourceDataForExpandedView/EMBOJ-2024-119243R_SourceDataForFigure EV4/EV4E/Input-Myc.tif]

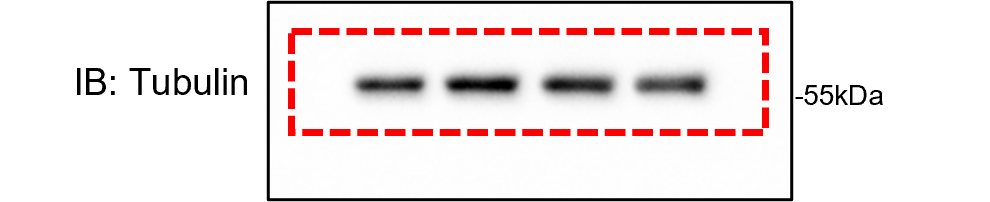

Supplement: Supplementary file 15 — Figure EV1-5 Source Data [file 44318_2025_416_MOESM15_ESM.zip › EMBOJ-2024-119243R_SourceDataForExpandedView/EMBOJ-2024-119243R_SourceDataForFigure EV4/EV4E/Input-Tubulin.tif]

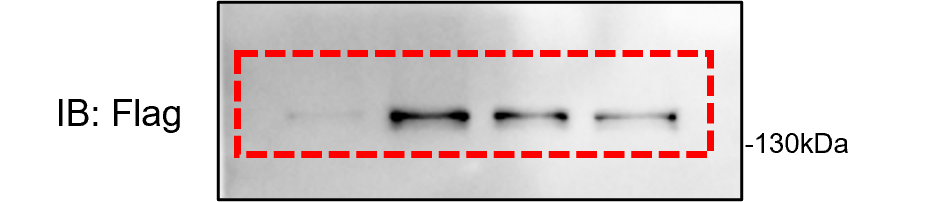

Supplement: Supplementary file 15 — Figure EV1-5 Source Data [file 44318_2025_416_MOESM15_ESM.zip › EMBOJ-2024-119243R_SourceDataForExpandedView/EMBOJ-2024-119243R_SourceDataForFigure EV4/EV4E/IP-Flag.tif]

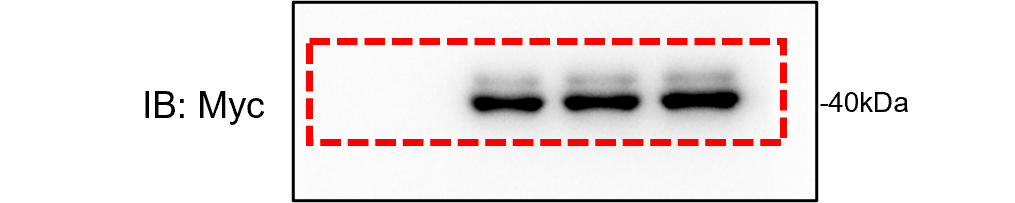

Supplement: Supplementary file 15 — Figure EV1-5 Source Data [file 44318_2025_416_MOESM15_ESM.zip › EMBOJ-2024-119243R_SourceDataForExpandedView/EMBOJ-2024-119243R_SourceDataForFigure EV4/EV4E/IP-Myc.tif]

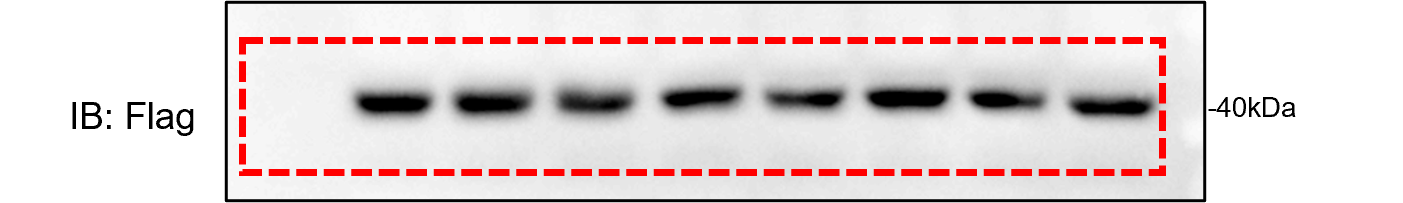

Supplement: Supplementary file 15 — Figure EV1-5 Source Data [file 44318_2025_416_MOESM15_ESM.zip › EMBOJ-2024-119243R_SourceDataForExpandedView/EMBOJ-2024-119243R_SourceDataForFigure EV4/EV4H/Flag.tif]

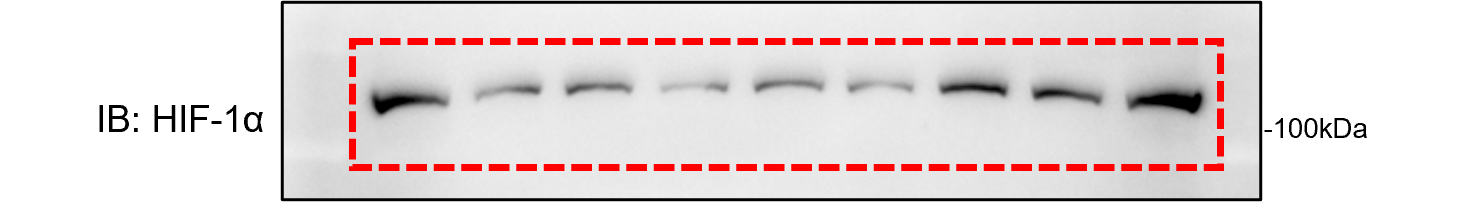

Supplement: Supplementary file 15 — Figure EV1-5 Source Data [file 44318_2025_416_MOESM15_ESM.zip › EMBOJ-2024-119243R_SourceDataForExpandedView/EMBOJ-2024-119243R_SourceDataForFigure EV4/EV4H/HIF-1α.tif]

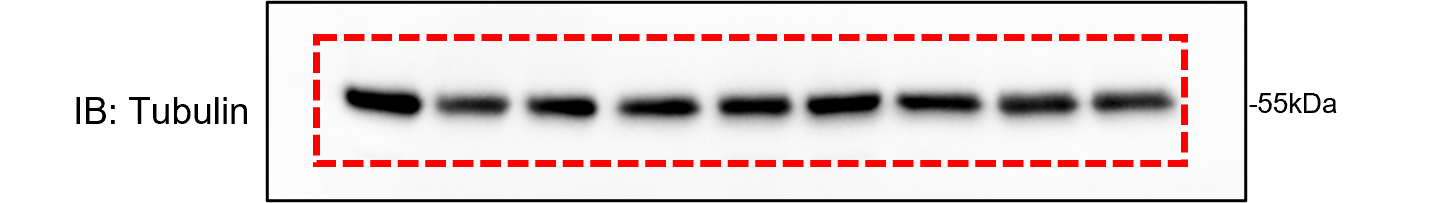

Supplement: Supplementary file 15 — Figure EV1-5 Source Data [file 44318_2025_416_MOESM15_ESM.zip › EMBOJ-2024-119243R_SourceDataForExpandedView/EMBOJ-2024-119243R_SourceDataForFigure EV4/EV4H/Tubulin.tif]

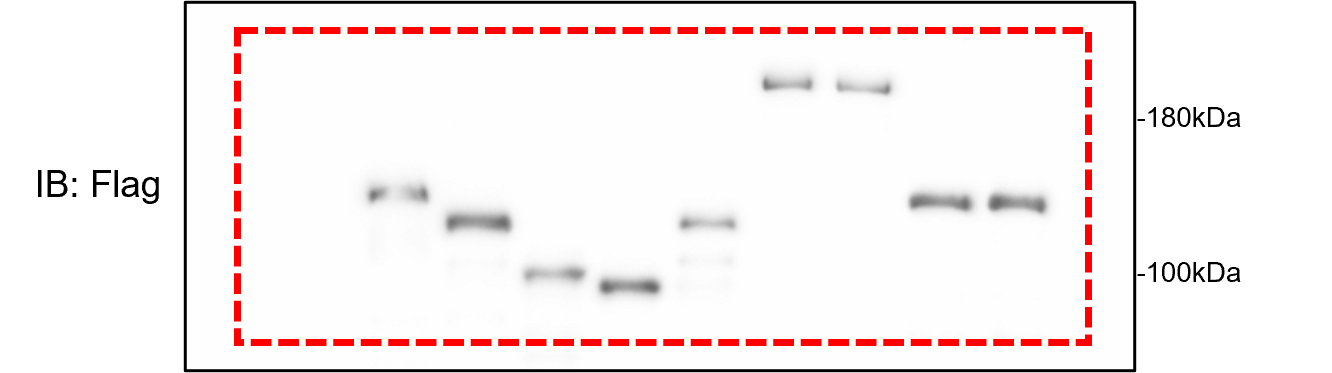

Supplement: Supplementary file 15 — Figure EV1-5 Source Data [file 44318_2025_416_MOESM15_ESM.zip › EMBOJ-2024-119243R_SourceDataForExpandedView/EMBOJ-2024-119243R_SourceDataForFigure EV4/EV4I/Input-Flag.tif]

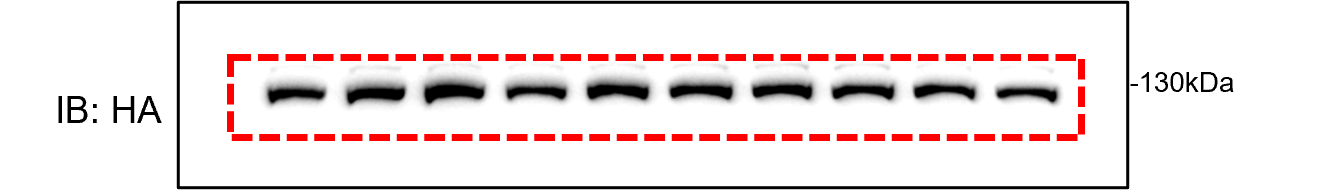

Supplement: Supplementary file 15 — Figure EV1-5 Source Data [file 44318_2025_416_MOESM15_ESM.zip › EMBOJ-2024-119243R_SourceDataForExpandedView/EMBOJ-2024-119243R_SourceDataForFigure EV4/EV4I/Input-HA.tif]

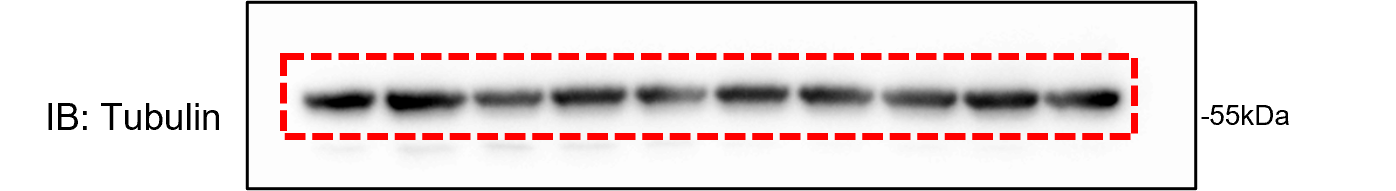

Supplement: Supplementary file 15 — Figure EV1-5 Source Data [file 44318_2025_416_MOESM15_ESM.zip › EMBOJ-2024-119243R_SourceDataForExpandedView/EMBOJ-2024-119243R_SourceDataForFigure EV4/EV4I/Input-Tubulin.tif]

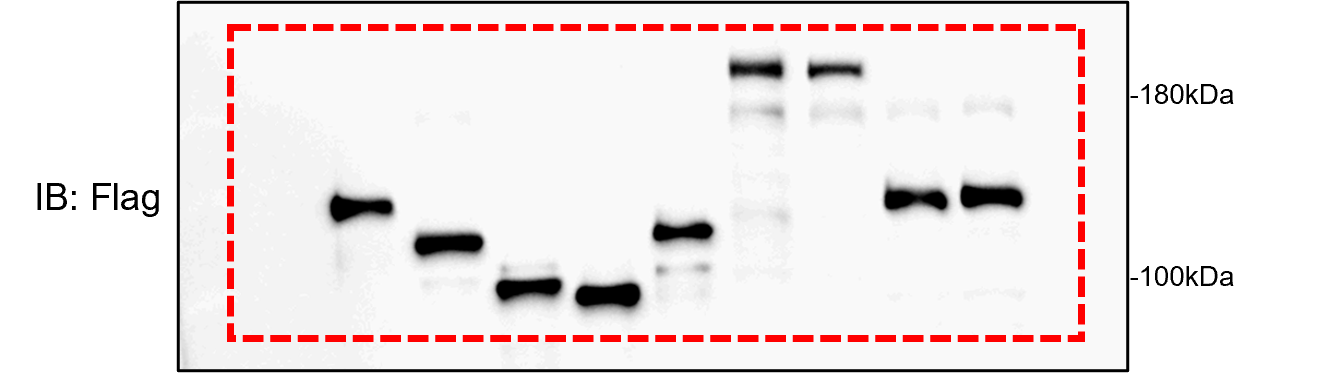

Supplement: Supplementary file 15 — Figure EV1-5 Source Data [file 44318_2025_416_MOESM15_ESM.zip › EMBOJ-2024-119243R_SourceDataForExpandedView/EMBOJ-2024-119243R_SourceDataForFigure EV4/EV4I/IP-Flag.tif]

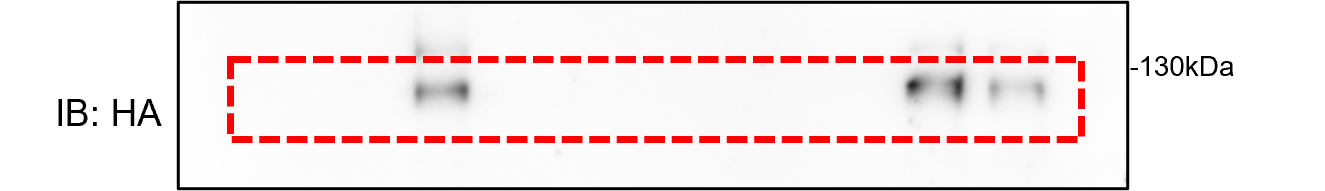

Supplement: Supplementary file 15 — Figure EV1-5 Source Data [file 44318_2025_416_MOESM15_ESM.zip › EMBOJ-2024-119243R_SourceDataForExpandedView/EMBOJ-2024-119243R_SourceDataForFigure EV4/EV4I/IP-HA.tif]

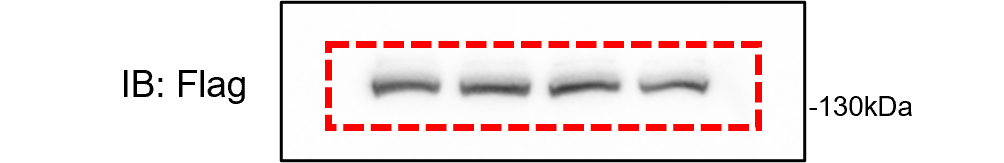

Supplement: Supplementary file 15 — Figure EV1-5 Source Data [file 44318_2025_416_MOESM15_ESM.zip › EMBOJ-2024-119243R_SourceDataForExpandedView/EMBOJ-2024-119243R_SourceDataForFigure EV4/EV4J/Input-Flag.tif]

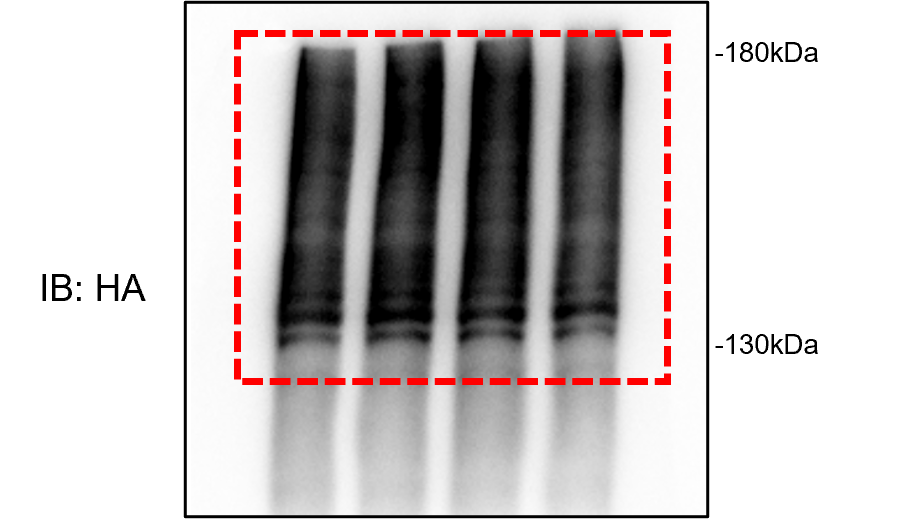

Supplement: Supplementary file 15 — Figure EV1-5 Source Data [file 44318_2025_416_MOESM15_ESM.zip › EMBOJ-2024-119243R_SourceDataForExpandedView/EMBOJ-2024-119243R_SourceDataForFigure EV4/EV4J/Input-HA.tif]

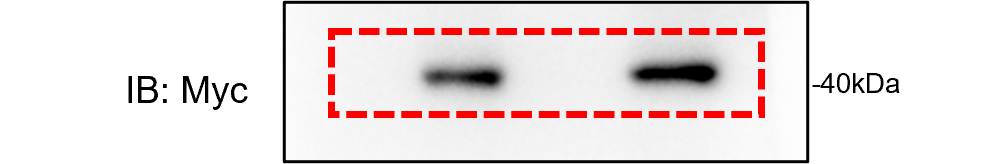

Supplement: Supplementary file 15 — Figure EV1-5 Source Data [file 44318_2025_416_MOESM15_ESM.zip › EMBOJ-2024-119243R_SourceDataForExpandedView/EMBOJ-2024-119243R_SourceDataForFigure EV4/EV4J/Input-Myc.tif]

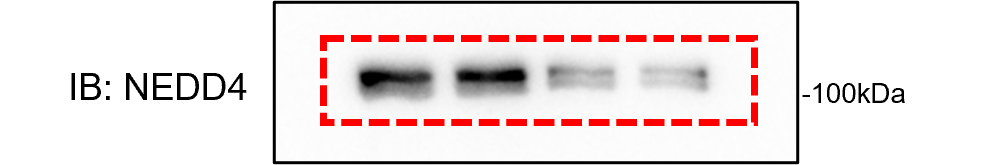

Supplement: Supplementary file 15 — Figure EV1-5 Source Data [file 44318_2025_416_MOESM15_ESM.zip › EMBOJ-2024-119243R_SourceDataForExpandedView/EMBOJ-2024-119243R_SourceDataForFigure EV4/EV4J/Input-NEDD4.tif]

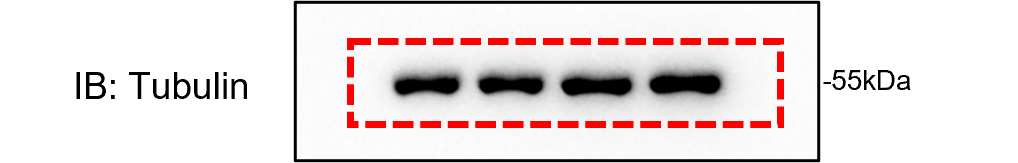

Supplement: Supplementary file 15 — Figure EV1-5 Source Data [file 44318_2025_416_MOESM15_ESM.zip › EMBOJ-2024-119243R_SourceDataForExpandedView/EMBOJ-2024-119243R_SourceDataForFigure EV4/EV4J/Input-Tubulin.tif]

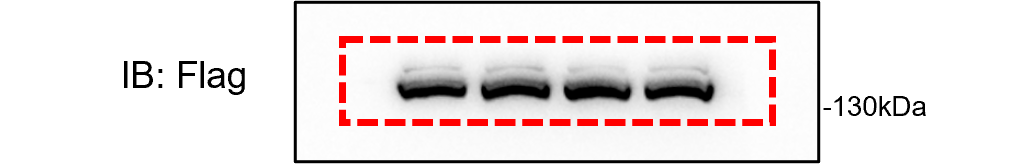

Supplement: Supplementary file 15 — Figure EV1-5 Source Data [file 44318_2025_416_MOESM15_ESM.zip › EMBOJ-2024-119243R_SourceDataForExpandedView/EMBOJ-2024-119243R_SourceDataForFigure EV4/EV4J/IP-Flag.tif]

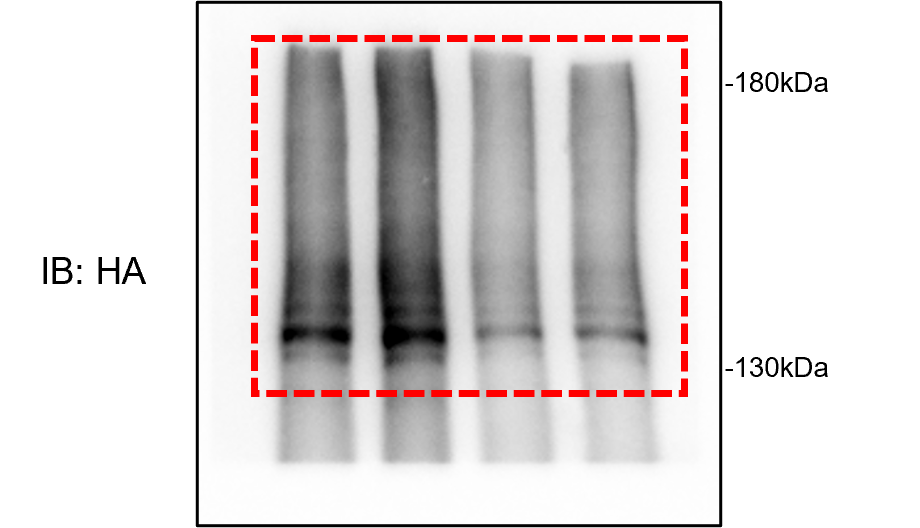

Supplement: Supplementary file 15 — Figure EV1-5 Source Data [file 44318_2025_416_MOESM15_ESM.zip › EMBOJ-2024-119243R_SourceDataForExpandedView/EMBOJ-2024-119243R_SourceDataForFigure EV4/EV4J/IP-HA.tif]

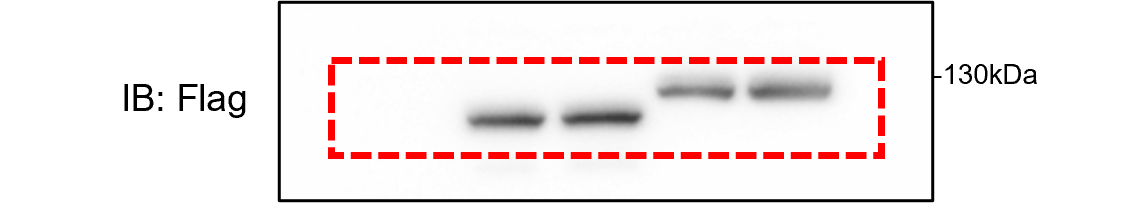

Supplement: Supplementary file 15 — Figure EV1-5 Source Data [file 44318_2025_416_MOESM15_ESM.zip › EMBOJ-2024-119243R_SourceDataForExpandedView/EMBOJ-2024-119243R_SourceDataForFigure EV4/EV4K/Input-Flag.tif]

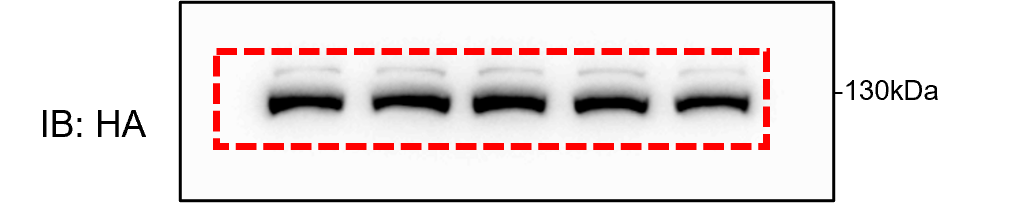

Supplement: Supplementary file 15 — Figure EV1-5 Source Data [file 44318_2025_416_MOESM15_ESM.zip › EMBOJ-2024-119243R_SourceDataForExpandedView/EMBOJ-2024-119243R_SourceDataForFigure EV4/EV4K/Input-HA.tif]

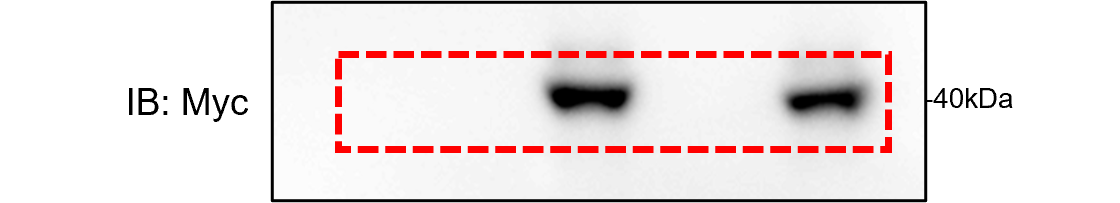

Supplement: Supplementary file 15 — Figure EV1-5 Source Data [file 44318_2025_416_MOESM15_ESM.zip › EMBOJ-2024-119243R_SourceDataForExpandedView/EMBOJ-2024-119243R_SourceDataForFigure EV4/EV4K/Input-Myc.tif]

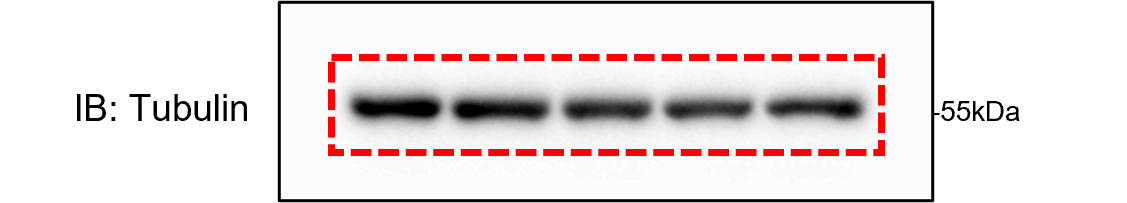

Supplement: Supplementary file 15 — Figure EV1-5 Source Data [file 44318_2025_416_MOESM15_ESM.zip › EMBOJ-2024-119243R_SourceDataForExpandedView/EMBOJ-2024-119243R_SourceDataForFigure EV4/EV4K/Input-Tubulin.tif]

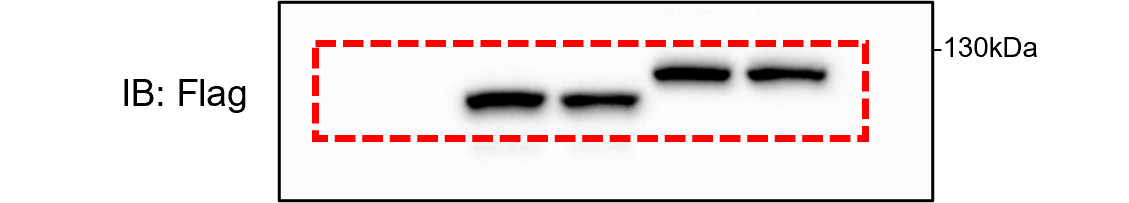

Supplement: Supplementary file 15 — Figure EV1-5 Source Data [file 44318_2025_416_MOESM15_ESM.zip › EMBOJ-2024-119243R_SourceDataForExpandedView/EMBOJ-2024-119243R_SourceDataForFigure EV4/EV4K/IP-Flag.tif]

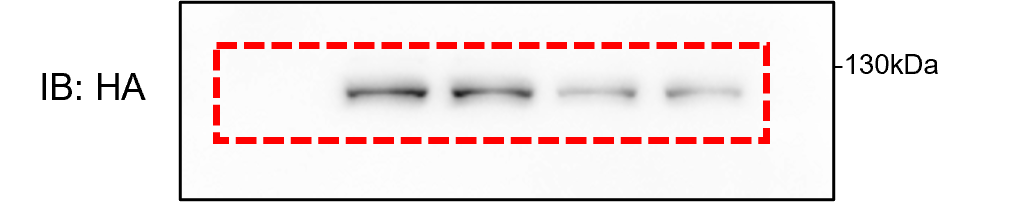

Supplement: Supplementary file 15 — Figure EV1-5 Source Data [file 44318_2025_416_MOESM15_ESM.zip › EMBOJ-2024-119243R_SourceDataForExpandedView/EMBOJ-2024-119243R_SourceDataForFigure EV4/EV4K/IP-HA.tif]

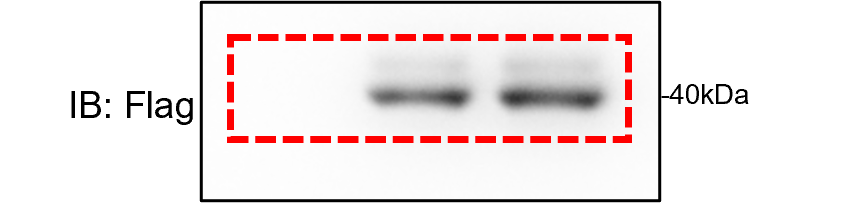

Supplement: Supplementary file 15 — Figure EV1-5 Source Data [file 44318_2025_416_MOESM15_ESM.zip › EMBOJ-2024-119243R_SourceDataForExpandedView/EMBOJ-2024-119243R_SourceDataForFigure EV4/EV4L/Input-Flag.tif]

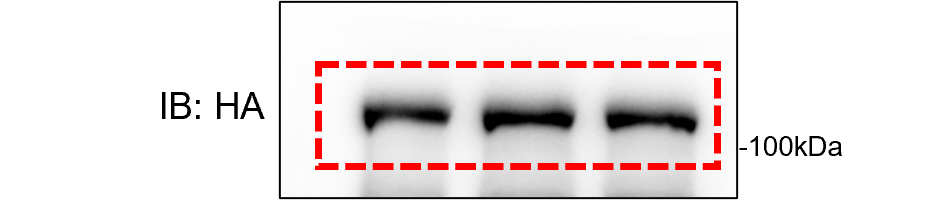

Supplement: Supplementary file 15 — Figure EV1-5 Source Data [file 44318_2025_416_MOESM15_ESM.zip › EMBOJ-2024-119243R_SourceDataForExpandedView/EMBOJ-2024-119243R_SourceDataForFigure EV4/EV4L/Input-HA.tif]

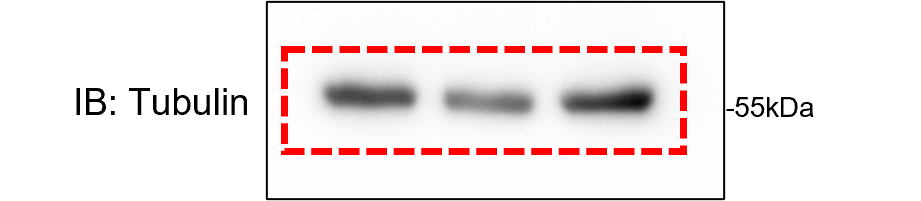

Supplement: Supplementary file 15 — Figure EV1-5 Source Data [file 44318_2025_416_MOESM15_ESM.zip › EMBOJ-2024-119243R_SourceDataForExpandedView/EMBOJ-2024-119243R_SourceDataForFigure EV4/EV4L/Input-Tubulin.tif]

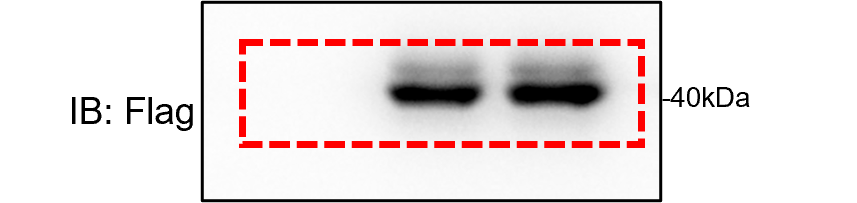

Supplement: Supplementary file 15 — Figure EV1-5 Source Data [file 44318_2025_416_MOESM15_ESM.zip › EMBOJ-2024-119243R_SourceDataForExpandedView/EMBOJ-2024-119243R_SourceDataForFigure EV4/EV4L/IP-Flag.tif]

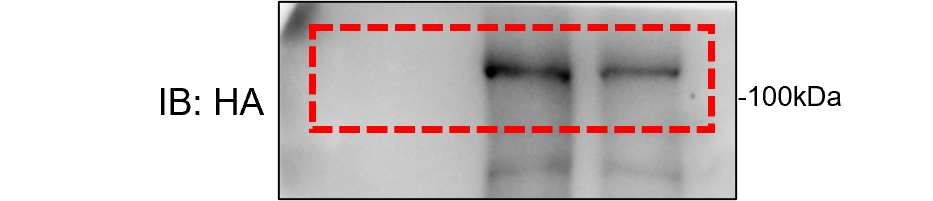

Supplement: Supplementary file 15 — Figure EV1-5 Source Data [file 44318_2025_416_MOESM15_ESM.zip › EMBOJ-2024-119243R_SourceDataForExpandedView/EMBOJ-2024-119243R_SourceDataForFigure EV4/EV4L/IP-HA.tif]

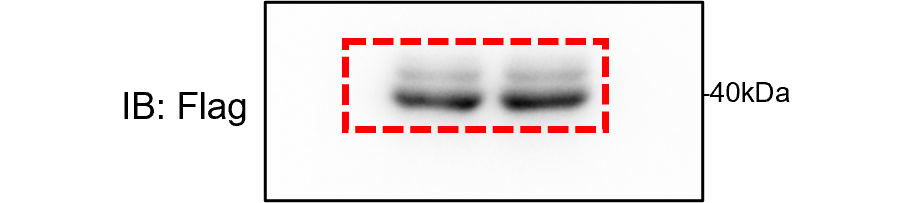

Supplement: Supplementary file 15 — Figure EV1-5 Source Data [file 44318_2025_416_MOESM15_ESM.zip › EMBOJ-2024-119243R_SourceDataForExpandedView/EMBOJ-2024-119243R_SourceDataForFigure EV5/EV5D/Flag.tif]

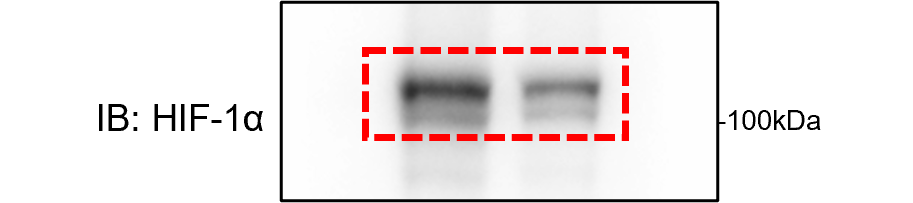

Supplement: Supplementary file 15 — Figure EV1-5 Source Data [file 44318_2025_416_MOESM15_ESM.zip › EMBOJ-2024-119243R_SourceDataForExpandedView/EMBOJ-2024-119243R_SourceDataForFigure EV5/EV5D/HIF-1α.tif]

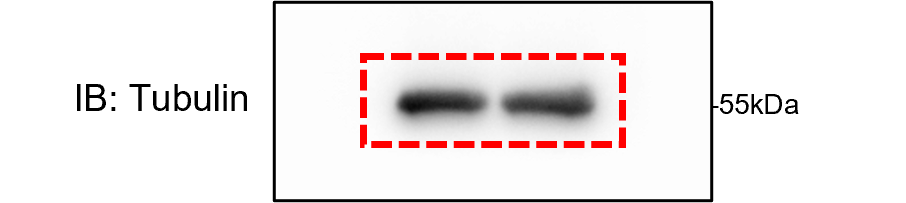

Supplement: Supplementary file 15 — Figure EV1-5 Source Data [file 44318_2025_416_MOESM15_ESM.zip › EMBOJ-2024-119243R_SourceDataForExpandedView/EMBOJ-2024-119243R_SourceDataForFigure EV5/EV5D/Tubulin.tif]

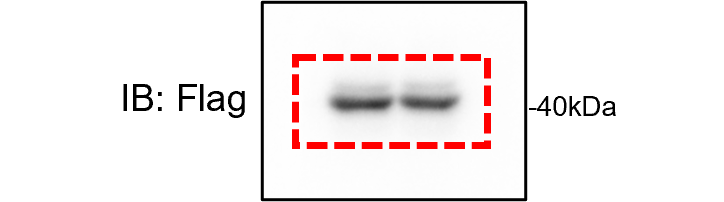

Supplement: Supplementary file 15 — Figure EV1-5 Source Data [file 44318_2025_416_MOESM15_ESM.zip › EMBOJ-2024-119243R_SourceDataForExpandedView/EMBOJ-2024-119243R_SourceDataForFigure EV5/EV5E/Input-Flag.tif]

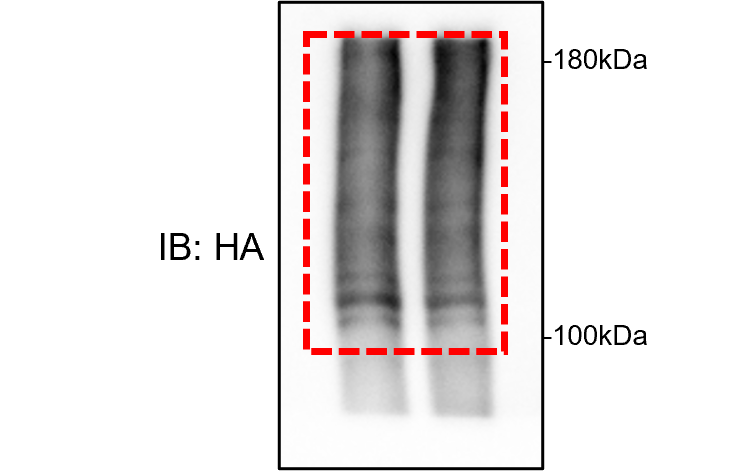

Supplement: Supplementary file 15 — Figure EV1-5 Source Data [file 44318_2025_416_MOESM15_ESM.zip › EMBOJ-2024-119243R_SourceDataForExpandedView/EMBOJ-2024-119243R_SourceDataForFigure EV5/EV5E/Input-HA.tif]

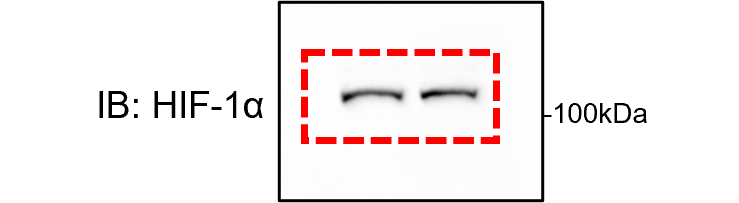

Supplement: Supplementary file 15 — Figure EV1-5 Source Data [file 44318_2025_416_MOESM15_ESM.zip › EMBOJ-2024-119243R_SourceDataForExpandedView/EMBOJ-2024-119243R_SourceDataForFigure EV5/EV5E/Input-HIF-1α.tif]

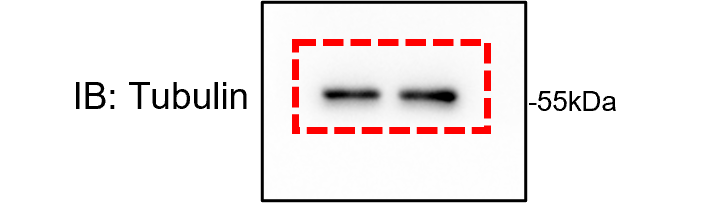

Supplement: Supplementary file 15 — Figure EV1-5 Source Data [file 44318_2025_416_MOESM15_ESM.zip › EMBOJ-2024-119243R_SourceDataForExpandedView/EMBOJ-2024-119243R_SourceDataForFigure EV5/EV5E/Input-Tubulin.tif]

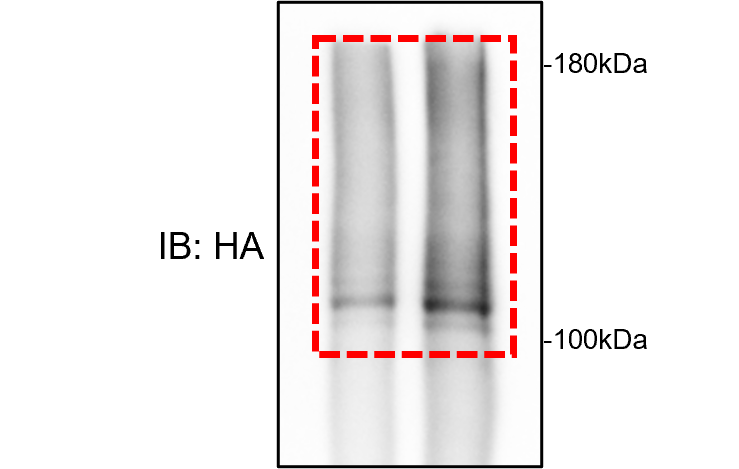

Supplement: Supplementary file 15 — Figure EV1-5 Source Data [file 44318_2025_416_MOESM15_ESM.zip › EMBOJ-2024-119243R_SourceDataForExpandedView/EMBOJ-2024-119243R_SourceDataForFigure EV5/EV5E/IP-HA.tif]

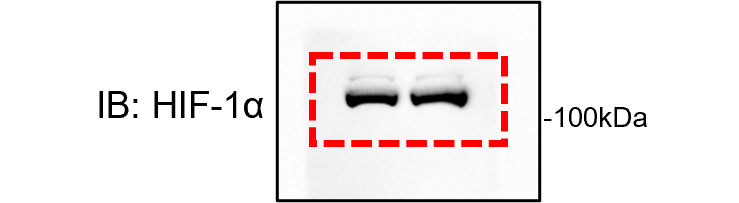

Supplement: Supplementary file 15 — Figure EV1-5 Source Data [file 44318_2025_416_MOESM15_ESM.zip › EMBOJ-2024-119243R_SourceDataForExpandedView/EMBOJ-2024-119243R_SourceDataForFigure EV5/EV5E/IP-HIF-1α.tif]

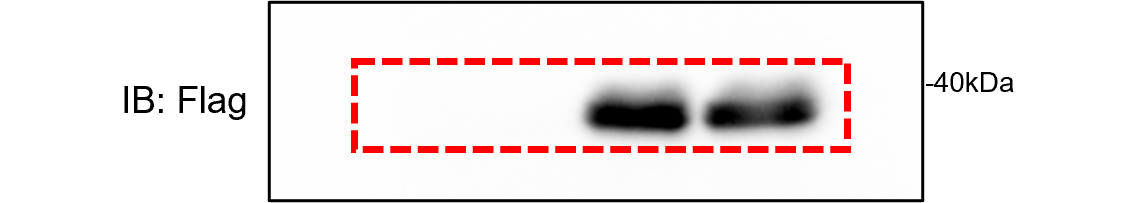

Supplement: Supplementary file 15 — Figure EV1-5 Source Data [file 44318_2025_416_MOESM15_ESM.zip › EMBOJ-2024-119243R_SourceDataForExpandedView/EMBOJ-2024-119243R_SourceDataForFigure EV5/EV5K/Flag.tif]

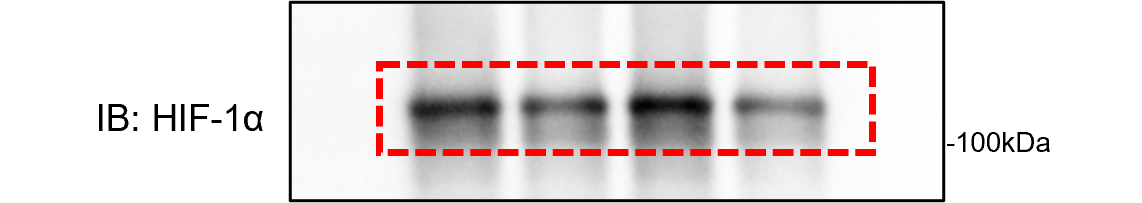

Supplement: Supplementary file 15 — Figure EV1-5 Source Data [file 44318_2025_416_MOESM15_ESM.zip › EMBOJ-2024-119243R_SourceDataForExpandedView/EMBOJ-2024-119243R_SourceDataForFigure EV5/EV5K/HIF-1α.tif]

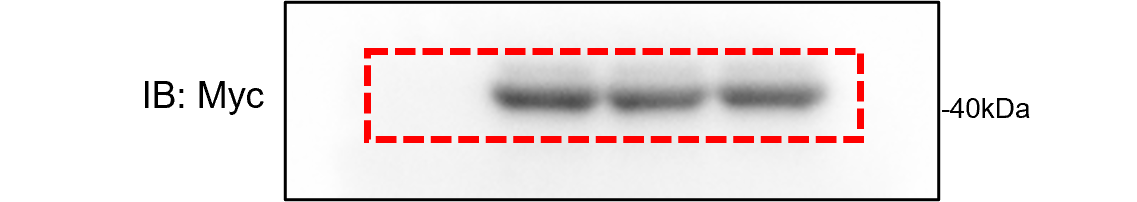

Supplement: Supplementary file 15 — Figure EV1-5 Source Data [file 44318_2025_416_MOESM15_ESM.zip › EMBOJ-2024-119243R_SourceDataForExpandedView/EMBOJ-2024-119243R_SourceDataForFigure EV5/EV5K/Myc.tif]

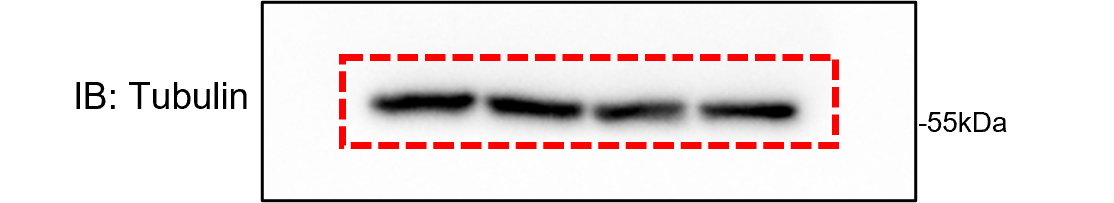

Supplement: Supplementary file 15 — Figure EV1-5 Source Data [file 44318_2025_416_MOESM15_ESM.zip › EMBOJ-2024-119243R_SourceDataForExpandedView/EMBOJ-2024-119243R_SourceDataForFigure EV5/EV5K/Tubulin.tif]
