# Supplementary material for: SIRT5-mediated desuccinylation of PPA2 enhances HIF-1alpha-dependent adaptation to hypoxic stress and colorectal cancer metastasis
Source: EMBO J. 2025 Mar 31;44(9):2514–40. doi: 10.1038/s44318-025-00416-1 (PMC12048626; doi:10.1038/s44318-025-00416-1)
Supplement: Supplementary file 16 — Expanded View Figures [file 44318_2025_416_MOESM16_ESM.pdf]

Expanded View Figures

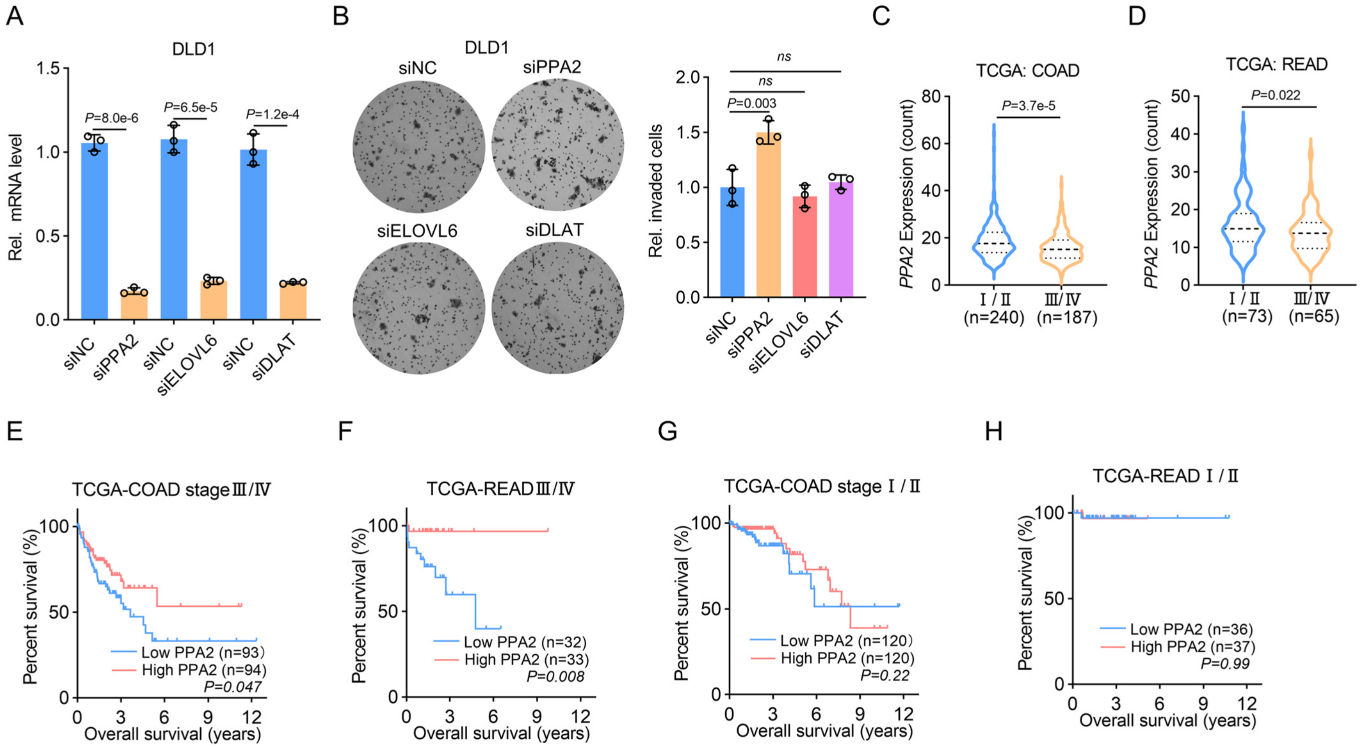

Figure EV1. PPA2 downregulation correlates with increased CRC metastasis and poor prognosis.

(**A**) qRT-PCR validated the efficiency of PPA2, ELOVL6 and DLAT knockdown in DLD1 cells. (**B**) Cells in (**A**) were used for transwell invasion assay. (**C**, **D**) TCGA RNA-seq data of patients with COAD (**C**) and READ (**D**) were analyzed. *PPA2* mRNA levels were compared between different stages. (**E–H**) Kaplan–Meier analysis of survival durations based on the PPA2 expression status at advanced stages (**E**, **F**) or early stages (**G**, **H**) from TCGA-COAD (**E**, **G**) or TCGA-READ (**F**, **H**) datasets. Data information: Data are mean ± SD from three biological replicates (**A**, **B**). Statistics: unpaired two-tailed student's t-test (**A**, **C**, **D**); one-way ANOVA (**B**); log-rank test (**E–H**). Source data are available online for this figure.

© The Author(s)

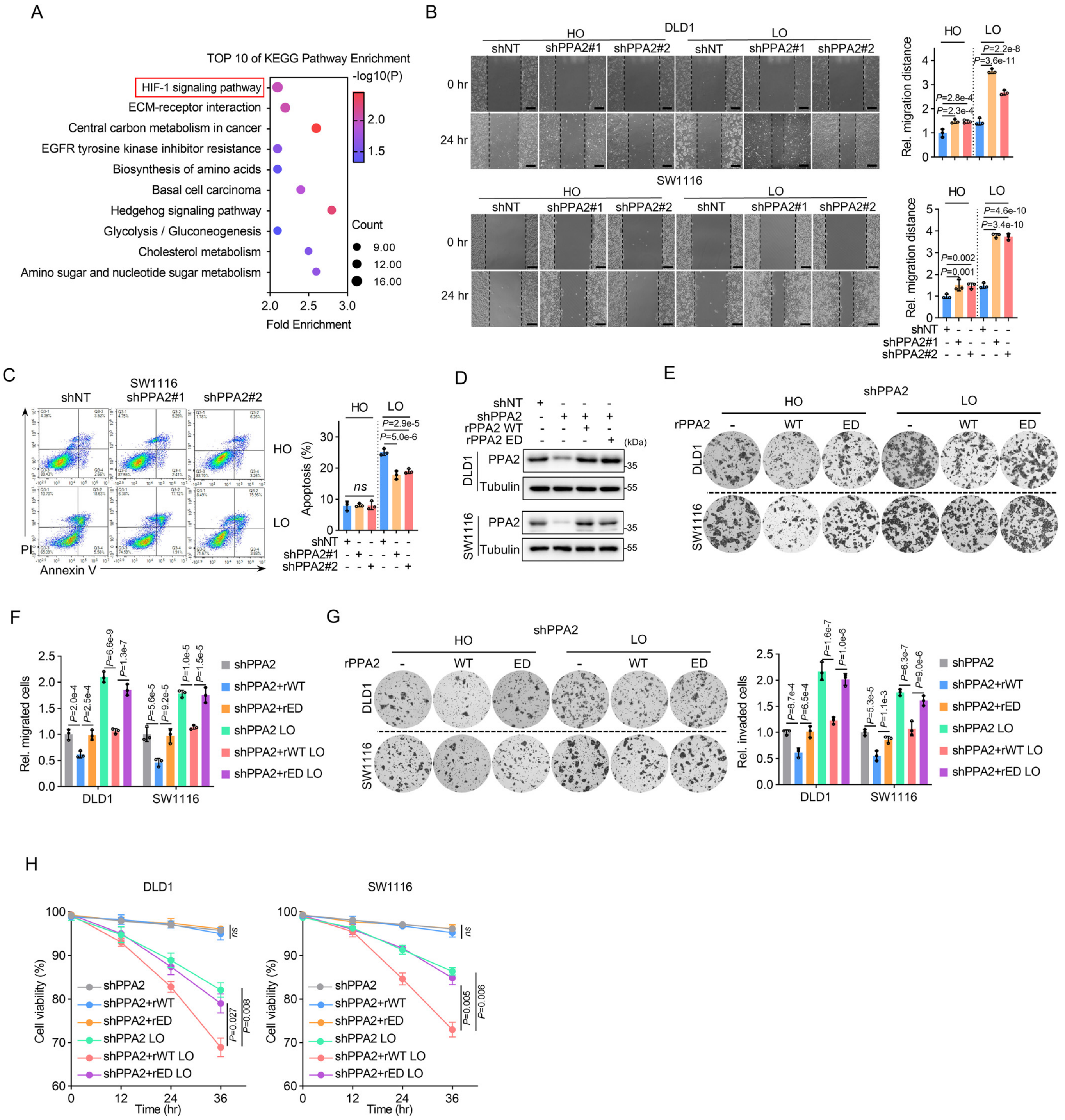

Figure EV2. PPA2 inhibits tumor cell migration and hypoxia resistance in an enzymatic activity-dependent manner.

(**A**) KEGG analysis of pathways upregulated in PPA2-depleted compared to control DLD1 cells. (**B**) Control or PPA2-depleted DLD1 and SW1116 cells were used for wound-healing assay under normoxia or hypoxia, respectively. Scale bar, 200 µm. (**C**) Control or PPA2-depleted SW1116 cells were collected after incubation under normoxia or hypoxia, respectively; PI/Annexin V double staining was then used to measure the percentage of apoptotic cells via flow cytometry. (**D**) IB analysis in control or endogenous PPA2-depleted DLD1 and SW1116 cells reconstituted with rPPA2 WT or ED mutant. (**E–G**) Cells in (**D**) were used for transwell migration (**E**, **F**) and invasion (**G**) assay under normoxia or hypoxia, respectively. (**H**) Cells in (**D**) were collected after incubation under normoxia or hypoxia for the indicated time, and cell viability was assessed by trypan blue staining. Data information: Data are mean ± SD from three biological replicates (**B**, **C**, **F–H**). Statistics: one-way ANOVA (**B**, **C**, **F**, **G**); two-way ANOVA (**H**). Source data are available online for this figure.

 © The Author(s)

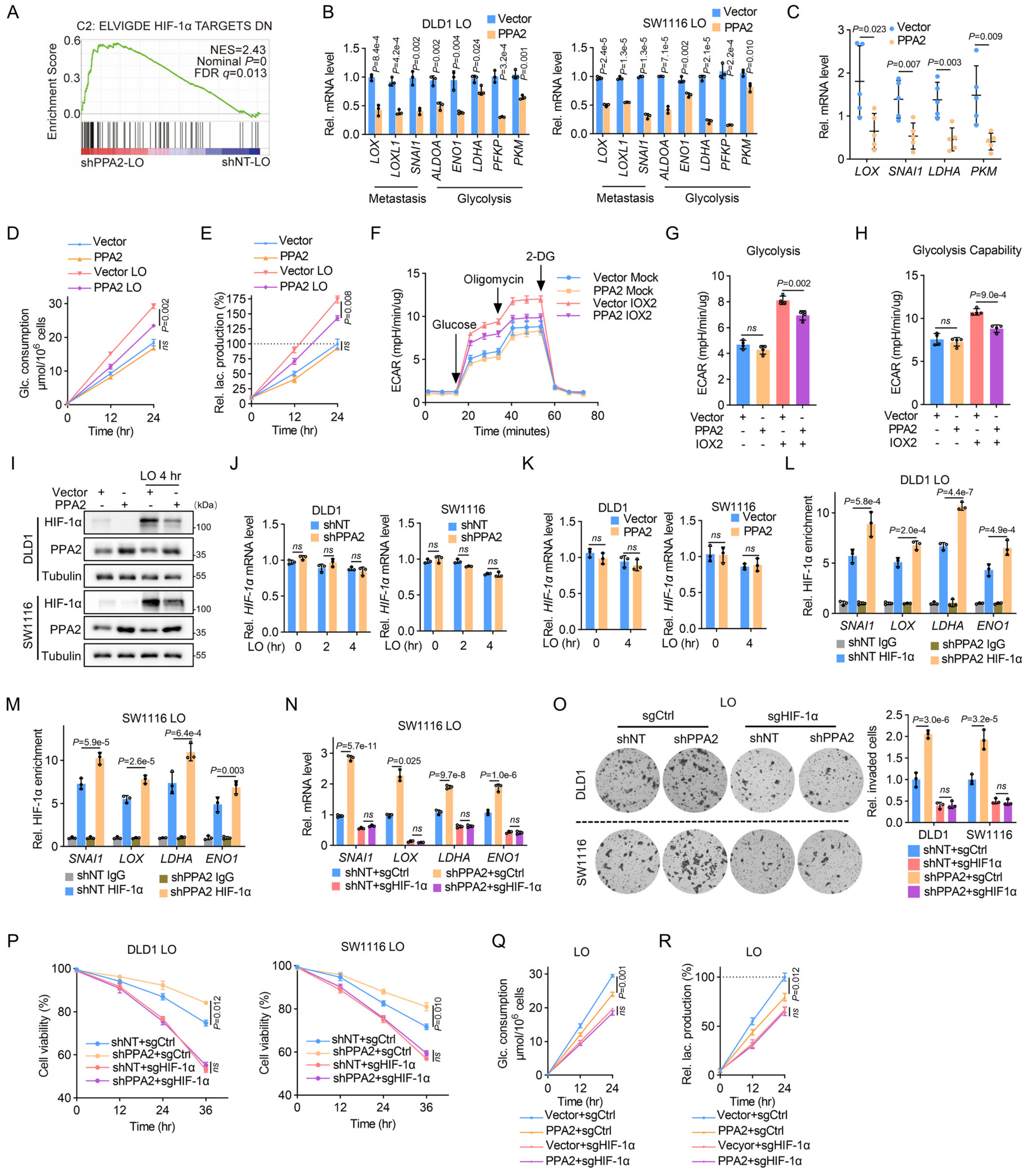
A
C2: ELVIGDE HIF-1α TARGETS DN
NES=2.43
Nominal P=0
FDR q=0.013
Enrichment Score
shPPA2-LO
shNT-LO
B
DLD1 LO
SW1116 LO
Vector
PPA2
Rel. mRNA level
LOX
LOXL1
SNAI1
ALDOA
ENO1
LDHA
PFKP
PKM
Metastasis
Glycolysis
C
Vector
PPA2
Rel. mRNA level
P=0.023
P=0.007
P=0.003
P=0.009
D
Vector
PPA2
Vector LO
PPA2 LO
Glc. consumption μmol/10^6 cells
Time (hr)
E
Rel. lac. production (%)
F
Glucose
Oligomycin
2-DG
Vector Mock
PPA2 Mock
Vector IOX2
PPA2 IOX2
ECAR (mpH/min/ug)
Time (minutes)
G
Glycolysis
H
Glycolysis Capability
IOX2
I
LO 4 hr
HIF-1α
PPA2
Tubulin
DLD1
SW1116
(kDa)
J
DLD1
SW1116
shNT
shPPA2
Rel. HIF-1α mRNA level
LO (hr)
K
L
DLD1 LO
Rel. HIF-1α enrichment
shNT IgG
shNT HIF-1α
shPPA2 IgG
shPPA2 HIF-1α
M
SW1116 LO
N
shNT+sgCtrl
shNT+sgHIF-1α
shPPA2+sgCtrl
shPPA2+sgHIF-1α
O
LO
sgCtrl
sgHIF-1α
shNT
shPPA2
Rel. invaded cells
P
DLD1 LO
SW1116 LO
Cell viability (%)
Q
LO
Vector+sgCtrl
PPA2+sgCtrl
Vector+sgHIF-1α
PPA2+sgHIF-1α
R
Vecyor+sgHIF-1α

Figure EV3. PPA2 inhibits glycolysis-mediated CRC metastasis by facilitating the degradation of HIF-1α.

(**A**) GSEA analysis of HIF-1α targets upregulated in PPA2-depleted compared to control DLD1 cells after incubation under hypoxia for 12 h. NES, normalized enriched score. FDR, False discovery rate. (**B**) DLD1 and SW1116 cells overexpressing vector or PPA2 were collected after incubation under hypoxia for 12 h, the mRNA level of HIF-1α target genes were analyzed via qRT-PCR. (**C**) The mRNA level of the HIF-1α target genes was analyzed via qRT-PCR in liver metastasis tissues derived from splenic injection using PPA2-overexpressed DLD1 cells. (**D**, **E**) The media of DLD1 cells overexpressing vector or PPA2 were collected after incubation under normoxia or hypoxia for the indicated time to detect the glucose (**D**) and lactate levels (**E**). The lactate level of vector group at 24 h was used as the standard for data normalization in (**E**). (**F–H**) DLD1 cells overexpressing vector or PPA2 were used for ECAR experiment with or without IOX2. (**I**) Control or PPA2-overexpressed DLD1 and SW1116 cells were collected after incubation under normoxia or hypoxia induction for 4 h. IB analysis was performed using the indicated antibodies. (**J**, **K**) PPA2-depleted (**J**) or PPA2-overexpressed (**K**) DLD1 and SW1116 cells were collected after incubation under normoxia or hypoxia for the indicated time to detect the mRNA level of *HIF-1α* via qRT-PCR. (**L**, **M**) Control or PPA2-depleted DLD1 and SW1116 cells were collected after incubation under hypoxia for 6 h. ChIP assays were performed with the indicated antibodies, and analyzed via qPCR with primers targeting the promoter region of the indicated genes. (**N**) Control or PPA2-depleted SW1116 cells treated with or without sgRNA targeting HIF-1α were collected after incubation under hypoxia for 12 h to detect the mRNA level of HIF-1α target genes via qRT-PCR. (**O**) Control or PPA2-depleted DLD1 and SW1116 cells treated with or without sgRNA targeting HIF-1α were used for transwell invasion assays under hypoxia. (**P**) Cells in (**O**) were collected after incubation under hypoxia for the indicated time, and cell viability was assessed by trypan blue staining. (**Q**, **R**) The media of DLD1 cells in (**O**) were collected after incubation under hypoxia for the indicated time to detect the glucose (**Q**) and lactate levels (**R**). The lactate level of vector+sgCtrl group at 24 h was used as the standard for data normalization in (**R**). Data information: $n =$ two biological replicates for each group (**A**). Data are mean ± SD from three biological replicates (**B**, **D**, **E**, **J–R**), five biological replicates (**C**) and four biological replicates (**F–H**). Statistics: permutation test on enrichment score was performed following Subramanian algorithm (**A**); unpaired two-tailed student's t-test (**B**, **C**, **J**, **K**); one-way ANOVA (**G**, **H**, **L–O**); two-way ANOVA (**D**, **E**, **P–R**). Source data are available online for this figure.

 © The Author(s)

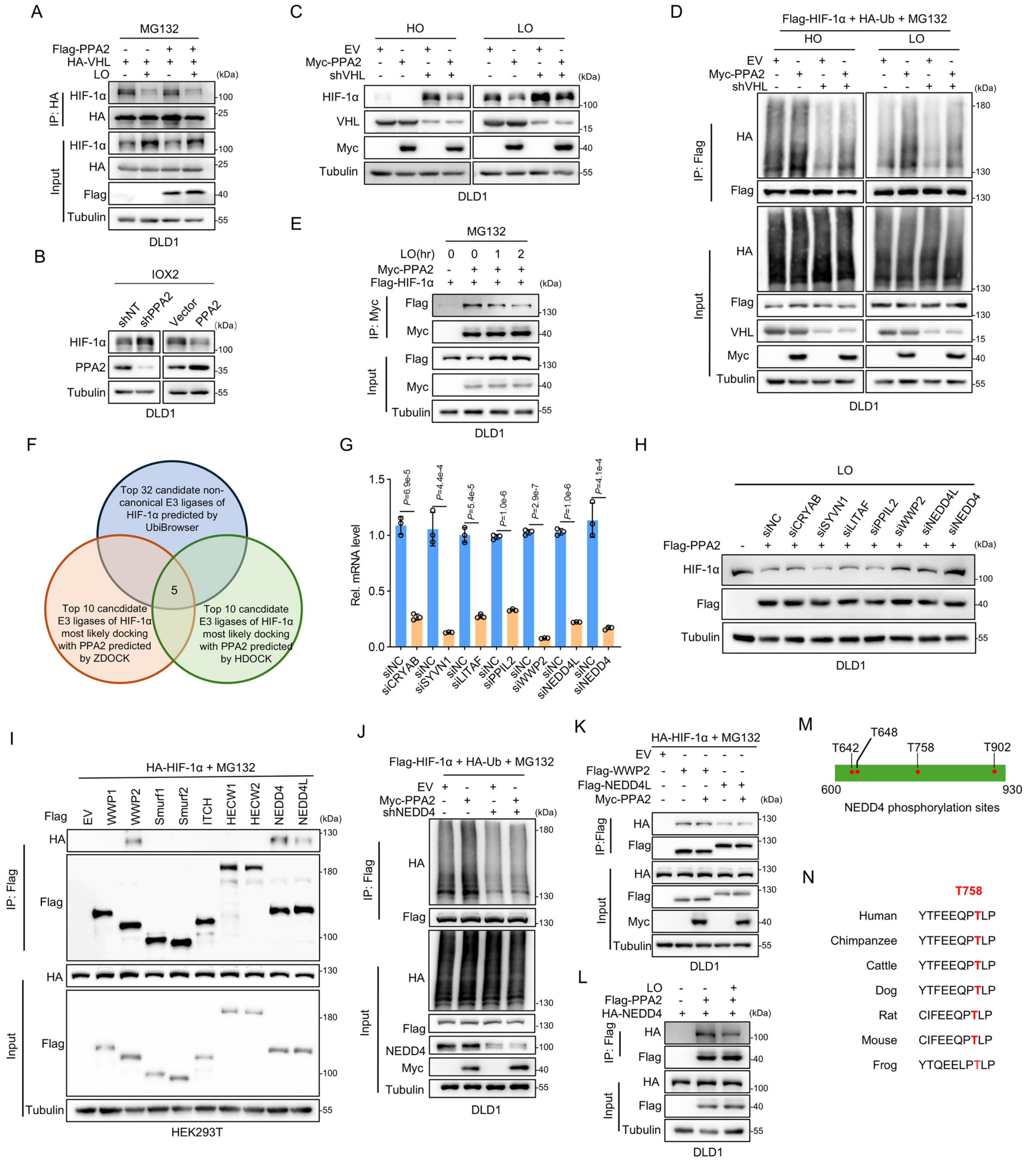
A
MG132
Flag-PPA2
HA-VHL
LO
(kDa)
IP: HA
HIF-1α
HA
Input
HIF-1α
HA
Flag
Tubulin
DLD1
B
IOX2
shNT
shPPA2
Vector
PPA2
HIF-1α
PPA2
Tubulin
DLD1
C
HO
LO
EV
Myc-PPA2
shVHL
HIF-1α
VHL
Myc
Tubulin
DLD1
D
Flag-HIF-1α + HA-Ub + MG132
HO
LO
EV
Myc-PPA2
shVHL
IP: Flag
HA
Flag
Input
HA
Flag
VHL
Myc
Tubulin
DLD1
E
MG132
LO(hr) 0 0 1 2
Myc-PPA2
Flag-HIF-1α
IP: Myc
Flag
Myc
Input
Flag
Myc
Tubulin
DLD1
F
Top 32 candidate non-canonical E3 ligases of HIF-1α predicted by UbiBrowser
5
Top 10 cancdidate E3 ligases of HIF-1α most likely docking with PPA2 predicted by ZDOCK
Top 10 cancdidate E3 ligases of HIF-1α most likely docking with PPA2 predicted by HDOCK
G
Rel. mRNA level
P=6.9e-5
P=4.4e-4
P=5.4e-5
P=1.0e-6
P=2.9e-7
P=1.0e-6
P=4.1e-4
siNC
siCRYAB
siSYVN1
siLITAF
siPPIL2
siWWP2
siNEDD4L
siNEDD4
H
LO
Flag-PPA2
HIF-1α
Flag
Tubulin
DLD1
I
HA-HIF-1α + MG132
Flag
EV
WWP1
WWP2
Smurf1
Smurf2
ITCH
HECW1
HECW2
NEDD4
NEDD4L
IP: Flag
HA
Flag
Input
HA
Flag
Tubulin
HEK293T
J
Flag-HIF-1α + HA-Ub + MG132
EV
Myc-PPA2
shNEDD4
IP: Flag
HA
Flag
Input
HA
Flag
NEDD4
Myc
Tubulin
DLD1
K
HA-HIF-1α + MG132
EV
Flag-WWP2
Flag-NEDD4L
Myc-PPA2
IP:Flag
HA
Flag
Input
HA
Flag
Myc
Tubulin
DLD1
L
LO
Flag-PPA2
HA-NEDD4
IP: Flag
HA
Flag
Input
HA
Flag
Tubulin
DLD1
M
T642
T648
T758
T902
600
930
NEDD4 phosphorylation sites
N
T758
Human YTFEEQPTLP
Chimpanzee YTFEEQPTLP
Cattle YTFEEQPTLP
Dog YTFEEQPTLP
Rat CIFEEQPTLP
Mouse CIFEEQPTLP
Frog YTQEELPTLP

Figure EV4. PPA2 recruits and activates NEDD4 for ubiquitin-mediated proteasomal degradation of HIF-1α.

(**A**) DLD1 cells transfected with HA-VHL and either empty vector (EV) or Flag-PPA2 plasmids. (**B**) The indicated DLD1 cells were treated with 40 μM IOX2 for 2 h. (**C**) DLD1 cells stably expressing shNT or shVHL were transfected with either EV or Myc-PPA2 plasmids. (**D**) DLD1 cells stably expressing shNT or shVHL were transfected with Flag-HIF-1α, HA-Ub and either EV or Myc-PPA2 plasmids. (**E**) DLD1 cells transfected with Flag-HIF-1α and either EV or Myc-PPA2 plasmids were incubated under hypoxia for the indicated time. (**F**) The candidate E3 ligases predicted by the UbiBrowser database were subjected to molecular docking analyses with PPA2 via ZDOCK and HDOCK, and the top 5 were sorted by the docking score. (**G**) PPA2-overexpressed DLD1 cells were transfected with siNC, siCRYAB, siSYVN1, siLITAF, siPPIL2, siWWP2, siNEDD4L, or siNEDD4 respectively, and the knockdown efficiencies were validated by qRT-PCR. (**H**) DLD1 cells overexpressing vector or Flag-PPA2 were transfected with siNC, siCRYAB, siSYVN1, siLITAF, siPPIL2, siWWP2, siNEDD4L, or siNEDD4 respectively. (**I**) DLD1 cells were transfected with HA-HIF-1α and EV, Flag-WWP1, WWP2, Smurf1, Smurf2, ITCH, HECW1, HECW2, NEDD4, or NEDD4L plasmids, respectively. (**J**) DLD1 cells stably expressing shNT or shNEDD4 were transfected with Flag-HIF-1α, HA-Ub and either EV or Myc-PPA2 plasmids. (**K**) DLD1 cells transfected with HA-HIF-1α and along with either EV, Flag-WWP2, Flag-NEDD4L, or Myc-PPA2 plasmids. (**L**) DLD1 cells were transfected with HA-NEDD4 and either EV or Flag-PPA2 plasmids. (**M**) Schematic diagram of potential threonine phosphorylation residues within 600-930AA of NEDD4 from PhosphoSitePlus. (**N**) Sequence alignment of NEDD4 T758 peptides in the indicated species. (**A–E**, **H–L**) IB analyses and IP were performed using the indicated antibodies. Six hours of MG132 (5 μM) treatment was administrated as indicated. Hypoxic stress was administrated in the last 2 h before the collection of cells as indicated (unless the hypoxia treatment time was specified). Data information: Data are mean ± SD from three biological replicates (**G**). Statistics: unpaired two-tailed student's t-test (**G**). Source data are available online for this figure.

 © The Author(s)

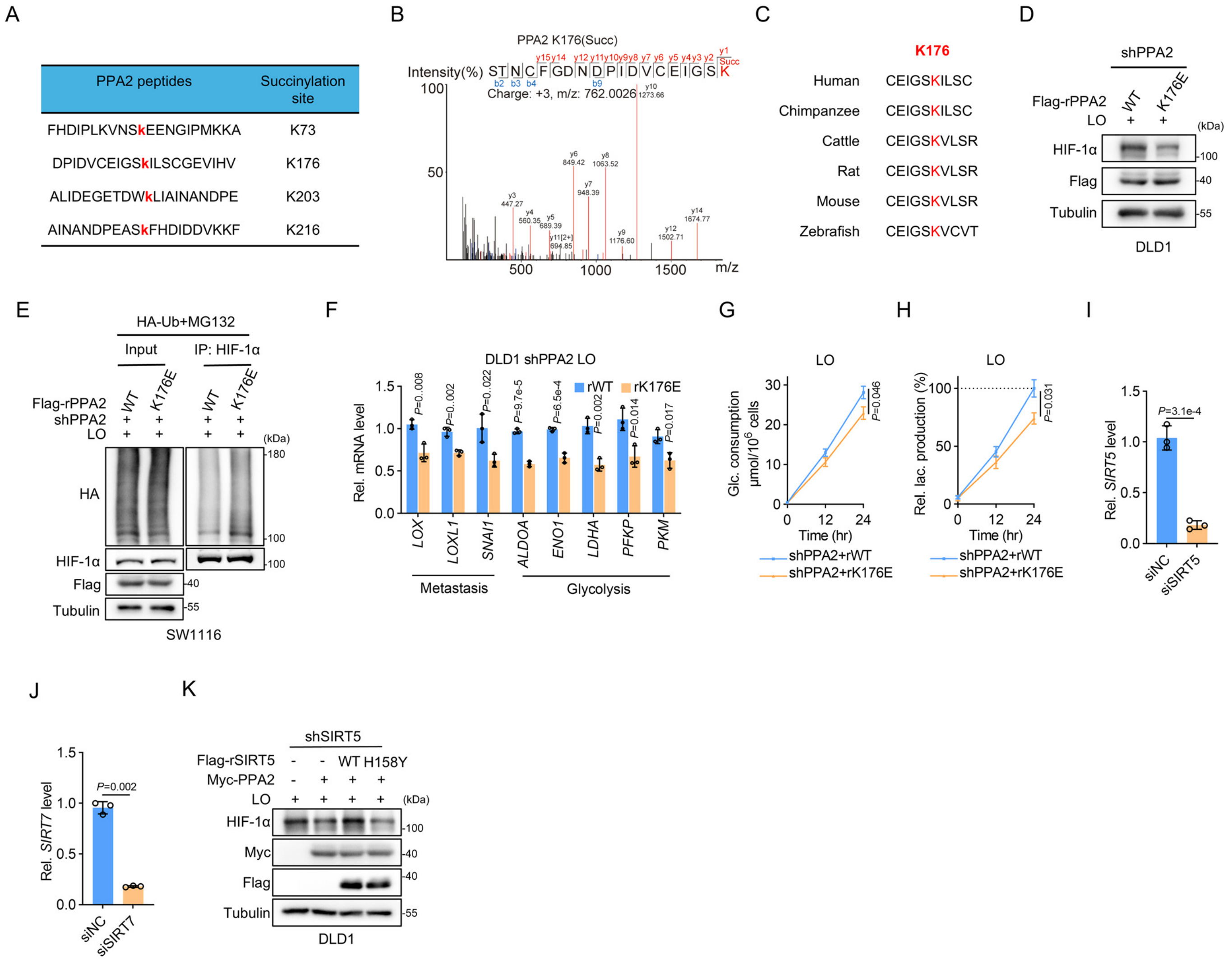

Figure EV5. SIRT5-mediated PPA2 desuccinylation at K176 impairs PPA2 function.

(**A**) Hypoxia induced desuccinylation of PPA2 was detected by LC-MS analysis at four lysine residues. (**B**) The LC-MS spectrum of hypoxia-induced modified K176 of PPA2 is shown. (**C**) Sequence alignment of PPA2 K176 peptides in the indicated species. (**D**) Endogenous PPA2-depleted DLD1 cells were transfected with Flag-rPPA2 WT or K176E mutant plasmids. (**E**) Endogenous PPA2-depleted SW1116 cells were transfected with HA-Ub and either Flag-rPPA2 WT or K176E mutant plasmids. (**F**) Endogenous PPA2-depleted DLD1 cells transfected with Flag-rPPA2 WT or K176E plasmids were incubated under hypoxia for 12 h, and were collected to detect the mRNA level of HIF-1α target genes. (**G**, **H**) The media of DLD1 cells in (**F**) were collected after incubation under hypoxia for the indicated time to detect the glucose (**G**) and lactate levels (**H**). The lactate level of shPPA2+rWT group at 24 h was used as the standard for data normalization in (**H**). (**I**, **J**) The knockdown efficiency of siSIRT5 (**I**) and siSIRT7 (**J**) in DLD1 cells was confirmed by qRT-PCR. (**K**) Endogenous SIRT5-depleted DLD1 cells were transfected with Flag-rSIRT5 WT or H158Y mutant and either EV or Myc-PPA2 plasmids. (**D**, **E**, **K**) IB analysis and IP were performed using the indicated antibodies. Six hours of MG132 (5 μM) treatment was administrated as indicated. Hypoxic stress was administrated in the last 2 h before collection of cells as indicated. Data information: Data are mean ± SD from three biological replicates (**F**–**J**). Statistics: unpaired two-tailed student's t-test (**F**, **I**, **J**); two-way ANOVA (**G**, **H**). Source data are available online for this figure.
